# Rapid molecular diversification and homogenization of clustered major ampullate silk genes in *Argiope* garden spiders

**DOI:** 10.1371/journal.pgen.1010537

**Published:** 2022-12-12

**Authors:** Richard H. Baker, André Corvelo, Cheryl Y. Hayashi

**Affiliations:** 1 Division of Invertebrate Zoology and Institute for Comparative Genomics, American Museum of Natural History, New York, New York, United States of America; 2 New York Genome Center, New York, New York, United States of America; University of North Carolina at Charlotte, UNITED STATES

## Abstract

The evolutionary diversification of orb-web weaving spiders is closely tied to the mechanical performance of dragline silk. This proteinaceous fiber provides the primary structural framework of orb web architecture, and its extraordinary toughness allows these structures to absorb the high energy of aerial prey impact. The dominant model of dragline silk molecular structure involves the combined function of two highly repetitive, spider-specific, silk genes (spidroins)—MaSp1 and MaSp2. Recent genomic studies, however, have suggested this framework is overly simplistic, and our understanding of how MaSp genes evolve is limited. Here we present a comprehensive analysis of MaSp structural and evolutionary diversity across species of *Argiope* (garden spiders). This genomic analysis reveals the largest catalog of MaSp genes found in any spider, driven largely by an expansion of MaSp2 genes. The rapid diversification of *Argiope* MaSp genes, located primarily in a single genomic cluster, is associated with profound changes in silk gene structure. MaSp2 genes, in particular, have evolved complex hierarchically organized repeat units (ensemble repeats) delineated by novel introns that exhibit remarkable evolutionary dynamics. These repetitive introns have arisen independently within the genus, are highly homogenized within a gene, but diverge rapidly between genes. In some cases, these iterated introns are organized in an alternating structure in which every other intron is nearly identical in sequence. We hypothesize that this intron structure has evolved to facilitate homogenization of the coding sequence. We also find evidence of intergenic gene conversion and identify a more diverse array of stereotypical amino acid repeats than previously recognized. Overall, the extreme diversification found among MaSp genes requires changes in the structure-function model of dragline silk performance that focuses on the differential use and interaction among various MaSp paralogs as well as the impact of ensemble repeat structure and different amino acid motifs on mechanical behavior.

## Author summary

Spider silk is renowned as one of the toughest natural or man-made materials. There are several different types of silk produced by orb-weaving spiders that serve different functions during a spider’s lifetime. Dragline silk, which provides the primary frame of an orb-web, must withstand the impact of flying prey without breaking or catapulting the insect out of the capture web. Dragline silk fibers are composed primarily of large proteins generated from a specific class of genes, the major ampullate spidroins (MaSps), but our understanding of the diversity and pattern of evolutionary change of these genes is limited. In this study, we provide the first detailed comparative examination of MaSp evolution across multiple species (gardens spiders in the genus *Argiope*) that focuses on multiple layers of genetic diversity. We find widespread divergence in copy number and sequence among the *Argiope* MaSp genes which indicates that new gene copies are often created and lost within the genome and can rapidly acquire new genetic features. Some of these features, such as nearly identical, regularly spaced introns and hierarchically organized protein units, are extremely unusual for any animal gene and provide valuable clues about how dragline silk achieves its remarkable strength and extensibility.

## Introduction

Silk production represents a key adaptation critical to the evolutionary success of spiders and is characterized by abundant variation in form and function throughout the order Araneae [1–3]. Silk fibers and secretions are utilized in numerous ecological roles in a spider’s life cycle including shelter, locomotion, predation, mating and egg protection, and achieve their greatest diversity in orb web spiders which have evolved seven different silk types generated from a suite of specialized glands [4–6]. This morphological diversification is mirrored at the genetic level as each silk type is comprised of distinct structural proteins (i.e., spidroins) that are expressed primarily in specific silk glands and whose molecular structure is specialized for that silk-type function. Dragline silk, produced in the major ampullate gland, is the most well studied of these various silks due to it widespread taxonomic distribution, functional significance and noteworthy mechanical properties [7]. Most spider species utilize this silk fiber as lifelines that are laid down during movement and allow a spider to rapidly descend from a web or other substrate. In addition, dragline silk serves as the primary frame of orb web architecture. The combination of high strength and extensibility associated with this silk places it among the most high-performing materials and has made it the subject of extensive recombinant biomimetic research [8–10].

Dragline silk has been understood to be composed primarily of two spidroins, MaSp1 and MaSp2, that have similar but distinct evolutionary histories and structural properties. Nearly all spidroins are encoded by large genes that belong to a single gene family that has undergone substantial diversification throughout the evolutionary history of spiders [11–14]. Spidroins share a common structure characterized by short conserved N- and C-terminal regions flanking a long repetitive region in which repeat units are often highly homogenized [15,16]. The repetitive units of MaSp proteins are comprised of separate poly-alanine (poly-A) and glycine-rich regions that provide contrasting structural properties [17,18]. Within the dragline fiber, poly-A regions align with each other to form β-sheet nanocrystal structures that provide strength and rigidity. Alternatively, the glycine-rich regions form a more amorphous matrix that confers enhanced extensibility [17,19,20]. Both MaSp genes have similar poly-A regions but the glycine-rich regions differ in the abundance of stereotypical motifs. MaSp1 contains an abundance of GGX motifs (the X position is variable but biased towards a limited set of amino acids), while GPGXX and QQ motifs are prominent in MaSp2 [21–23]. The abundance of proline in MaSp2 has a substantial impact on the mechanical properties of silk fibers as numerous studies have demonstrated a relationship between the percentage of proline content and extensibility [24–28]. Therefore, given that orb-weaver dragline silk is primarily composed of MaSp1 and MaSp2, the specific ratio of each protein can impact the relative strength and extensibility, and hence toughness, of fibers [24,28–31].

The mechanical properties of dragline fibers exhibit abundant inter- and intraspecific variation. In fact, under some conditions, the variation found among fibers spun from an individual spider is comparable to that measured across evolutionarily divergent species [32–34]. Protein sequence, spinning dynamics (i.e., how the silk is converted to a solid fiber from a liquid dope) and ecological factors (e.g., diet, humidity) all impact dragline variation [1,29,35], but the relative importance of each category is incompletely understood. In addition, the numerous factors affecting silk performance have made it difficult to compare silks across species in a standardized format with several studies recording low phylogenetic signal in silk properties [36–39]. Recently, the experimental use of ‘supercontraction’ has facilitated comparative analyses of different silks. Supercontraction occurs when silk fibers are exposed to water, breaking bonds created by the spinning process and returning the silk to a ‘ground state’. Several studies employing this approach [2,37,40,41] have examined silk property differences across a range of taxonomic distances and found a general trend toward increased extensibility and toughness of dragline silk in derived orb-weavers. One potential mechanism driving this pattern is an increase in the proportion of MaSp2 proteins in dragline silk. Surveys of spidroin amino acid motif representation throughout spiders [12,23,42–44] indicate that dragline silk in basal Araneomorphae taxa have sequence characteristics more similar to MaSp1 and that the GPGXX and QQ motifs found in MaSp2 arose later in spider evolution. While spiders from the retrolateral tibial apophysis (RTA) clade, Uloboridae, Theridiidae, and Nephilinae all exhibit some evidence of MaSp2 expression, derived Araneidae spiders have substantially increased their investment in this silk protein. These orb-weavers exhibit the highest proportion of proline in dragline silk which reflects an increased use of MaSp2 and likely contributes to the enhanced extensibility of dragline fibers in this group [26,28,29,42].

Although amino acid repeat sequences have been documented for several spider species [12,14,45,46] our understanding of the direct impact of protein sequence and structure on spidroin functional performance has been limited by a lack of full-length sequences for these genes. Due to their large size and homogenized repeat structure, spidroins are especially difficult to sequence in entirety. Ayoub et al. [47] published the first full length MaSp genes from the black widow, *Latrodectus hesperus*, demonstrating that the standard repeat units that include both a glycine-rich and poly-alanine region were organized into a higher-order repeat structure composed of several variable smaller units in a stereotypical pattern. Full-length MaSp1 and MaSp2 sequences were also presented for *Argiope bruennichi* [48] and revealed similar higher-order organization. The advent of long-read sequencing, in both genomic and transcriptomic studies, has dramatically enhanced our ability to accurately describe spidroin copy number variation and protein organization. Several recent studies [49–56] have utilized this technology to describe a complete or partial catalogue of full-length spidroins in individual species. Overall, they have demonstrated that MaSp gene diversity is substantially more extensive than previously recognized. For instance, the *Trichonephila clavipes* genome contains ten distinct MaSp genes [49,51,57]. Similarly, new MaSp genes with divergent molecular characteristics relative to MaSp1 and MaSp2 have been identified. Kono et al. [50,51] described the full-length sequence for a new MaSp paralogue [45], called MaSp3, with novel amino acid motifs that arose with the Araneidae, and Garb et al. [55] identified another novel MaSp paralogue, MaSp4, that may be a critical component of the extreme dragline mechanical properties found in Darwin’s bark spider, *Caerostris darwini*.

Despite recent improvements in spidroin genomic assembly, no study has examined the pattern of molecular variation across multiple full-length spidroins in a comparative framework, and here we present such an analysis of the MaSp gene complex in three species of *Argiope* garden spiders. Spiders in this genus are well known for producing large orb webs, often decorated with an elaborate stabilimentum [58], that are commonly spun in open environments to capture large diurnal insects such as grasshoppers and bees. The mechanical properties of *Argiope* dragline silk have been the subject of several studies [2,30,37,41] and been shown to exhibit high strength and extensibility. In fact, because of its notable mechanical properties, *A*. *aurantia* was chosen as the reference species for the “Spider Silk Standardization Initiative” [40] which provides a framework for comparing the variation in dragline silk properties across all species under standardized environmental conditions. Despite the generally high performance of *Argiope* dragline fibers, substantial mechanical variation exists among different species within the genus [39] making it an excellent system for studying the evolutionary diversification of spidroins. Our genomic analysis identifies a large, phylogenetically conserved, cluster of genes containing up to 13 representative MaSp genes that have undergone extensive diversification within the genus. MaSp2 genes, in particular, exhibit the most substantial variation at multiple levels—paralog diversity, gene structure and motif representation—that is driven by a complex suite of molecular processes likely involving selection, homogenization and intergenic gene conversion.

## Results

### Abundant gene duplication produced the MaSp genomic cluster

For all three *Argiope* species, separate genomes were produced using both 10X Chromium and Oxford Nanopore Technologies (ONT). 10X assembly of Illumina short reads was conducted with Supernova v2.0.1 combined with a custom protocol that uses linked read information to identify genomic contigs adjacent to each other (see Material and Methods for more details). The ONT assemblies first employed a long read self-correction step, followed by assembly of the corrected reads and short read error correction of frameshifts for spidroin contigs.

**Fig 1:**
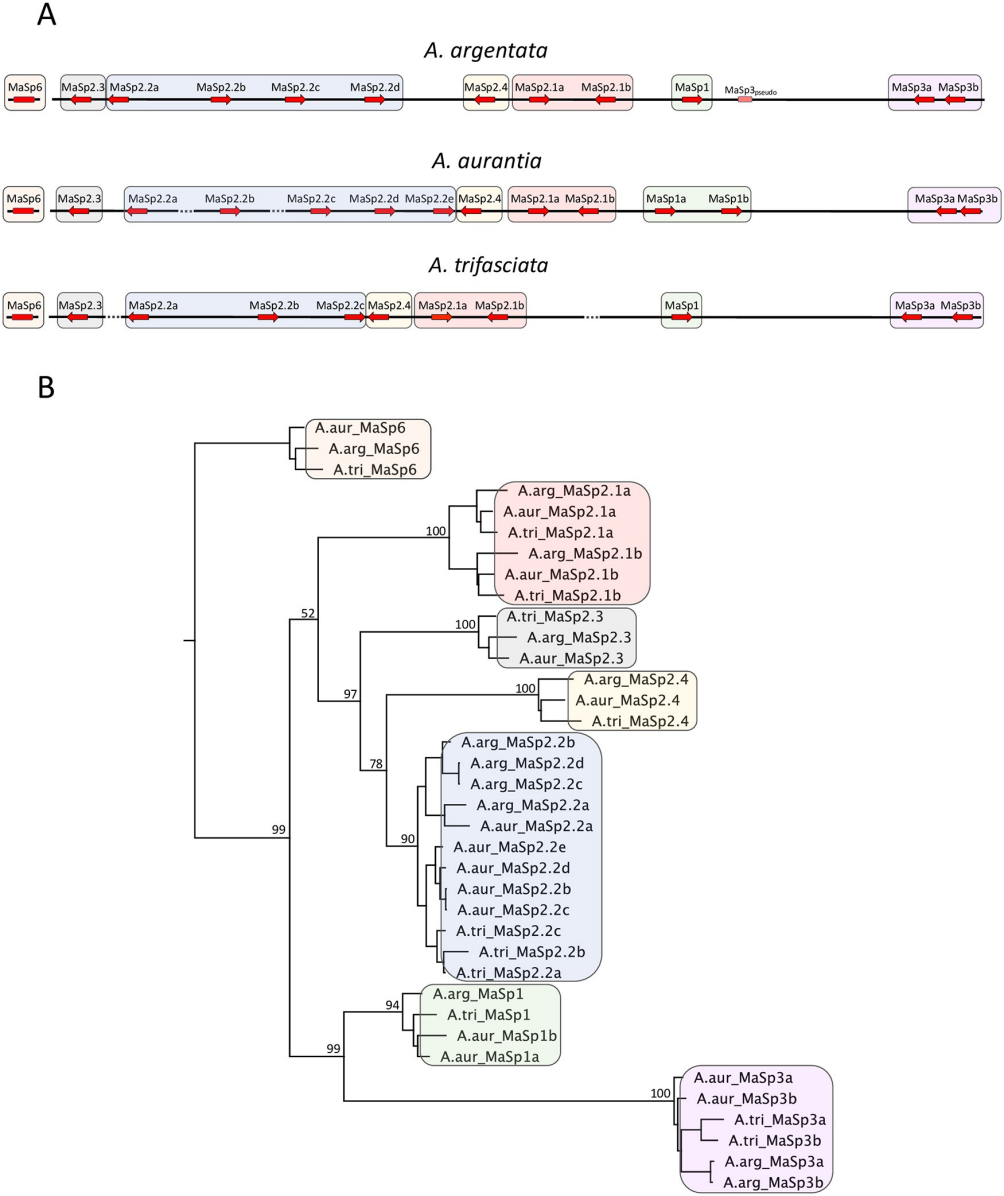
Organization and phylogenetic relationships of MaSp genes in *Argiope*. A) Schematic depiction of the genomic cluster containing most of the MaSp paralogs in each *Argiope* species (MaSp6 is located in a separate region in each species). The relative directionality of transcription is indicated by arrows. Dotted line regions between genes indicate gaps in the assembly where there is no direct support for contiguity between genes. The size of the cluster differs for each species and is approximately 1–1.5 Mb in size (see S1 Fig for additional details). The numbers associated with each MaSp gene name reflect putative homology relationships, while the letters reflect genomic location within each group. B) Phylogenetic relationships among all gene copies based on the concatenated N- and C-terminal nucleotide sequences for each gene. Bootstrap values provided for nodes defining relationships among primary MaSp clades. The tree was rooted with MaSp6 because this gene is not a member of the MaSp cluster.

Both the 10X and ONT genome assemblies of each *Argiope* species produced genomes roughly 1.8 GB in size. Contig N50 was slightly larger for the 10X assemblies than the ONT assemblies (S1 Table) but, because the 10X genomes are constructed entirely from short reads, most spidroins were not assembled with these data (N- and C-termini regions were separated by ambiguous bases) whereas the ONT genome assemblies reconstructed most spidroins in their entirety (see S2 Table for details of long read coverage for each spidroin). Blast annotation of these spidroins reveal 10–14 MaSp genes within each *Argiope* genome, all but one of which are co-localized within a genomic region containing almost exclusively these genes. No single assembly produced a contig containing all genes from this cluster, but the 10X and ONT MaSp-containing clusters for *A*. *argentata* had sufficient overlap to reconstruct the entire cluster (S1 Fig). Both *A*. *aurantia* and *A*. *trifasciata* have two points within the complex where there is no direct genomic support linking contigs with different MaSp genes together (Figs 1, S1). Given the strong synteny in MaSp order and orientation across species at all other regions of the assembly it is likely that these points of assembly ambiguity correspond to the pattern found in the other species.

The diversity of MaSp genes found within *Argiope* is substantially greater than what has been described in any other species. The gene set for these three *Argiope* species comprises seven distinct groups, generally arranged adjacent to one another in a genomic cluster, and four of these groups contain multiple paralogs for some species (Fig 1). Members of one clade, MaSp6, are not located in this cluster. The majority of genes belong to the well-described MaSp gene types—MaSp1, 2 and 3—but there are a few other types—MaSp2.3,2.4 and 6—that don’t easily fit into these categories but have clear homology to MaSp genes (Fig 1). The MaSp2 genes contain the greatest diversity, with 7–9 paralogs per species split into two well-defined clades, MaSp2.1 and MaSp2.2, that contain canonical MaSp genes with distinct poly-A regions and two other clades with MaSp2 genes (2.3 and 2.4) that are smaller in size and lack poly-A regions. MaSp1 and MaSp3 genes form a monophyletic clade, with two copies of each gene in some species. *A*. *argentata* possesses a third MaSp3 paralog though this gene is truncated (2049 aa) and contains a stop codon so it is likely a pseudogene.

### MaSp2 gene structure evolves rapidly

**Fig 2:**
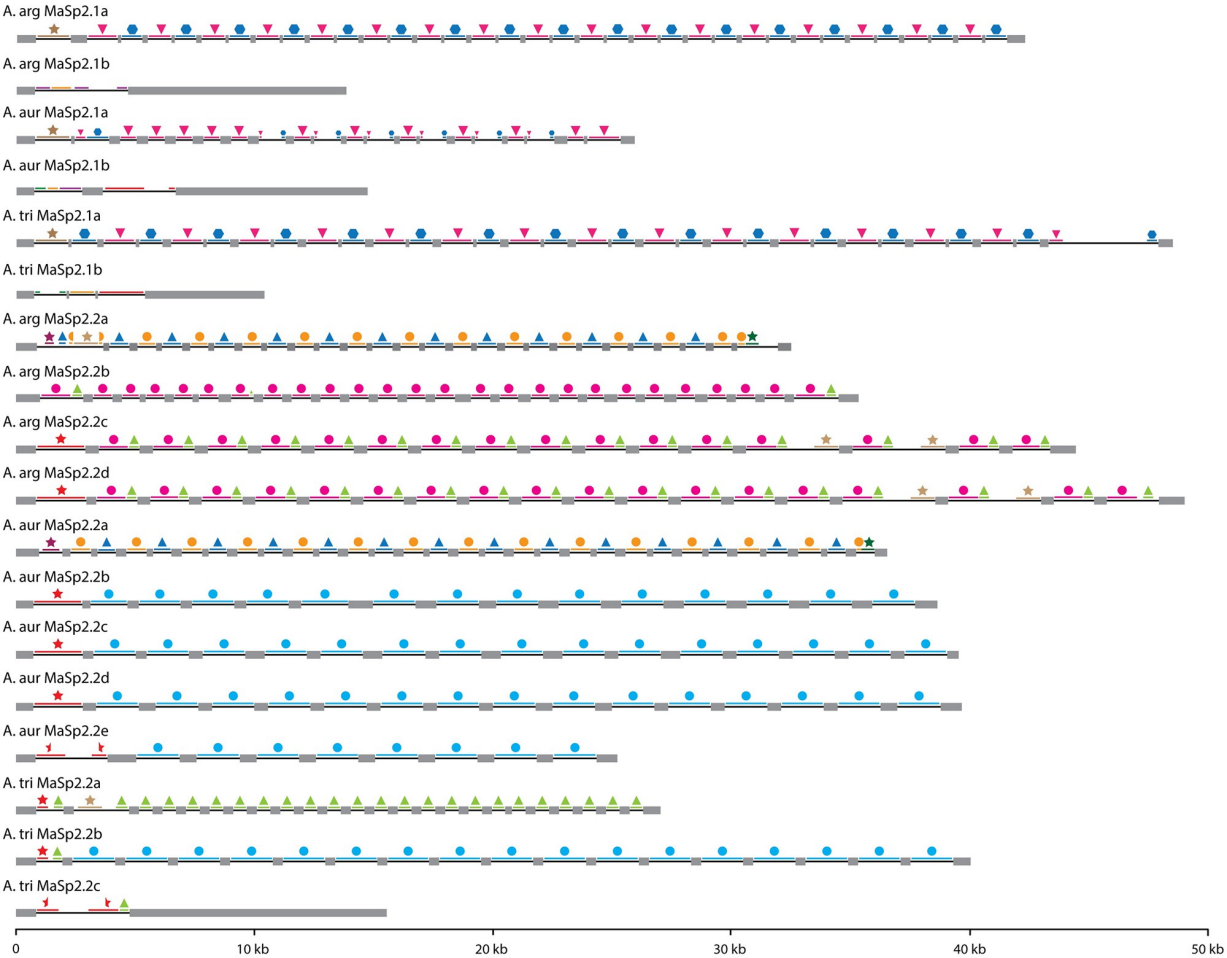
MaSp2.1 and MaSp2.2 gene structure. Intron/exon structure and homology relationships among introns is depicted. The exons are represented by gray rectangles and the introns by black lines. The colored lines and shape combinations above each intron indicate regions of sequence similarity shared among genes (each unique combination of color and shape represents a separate homology group).

**Fig 3:**
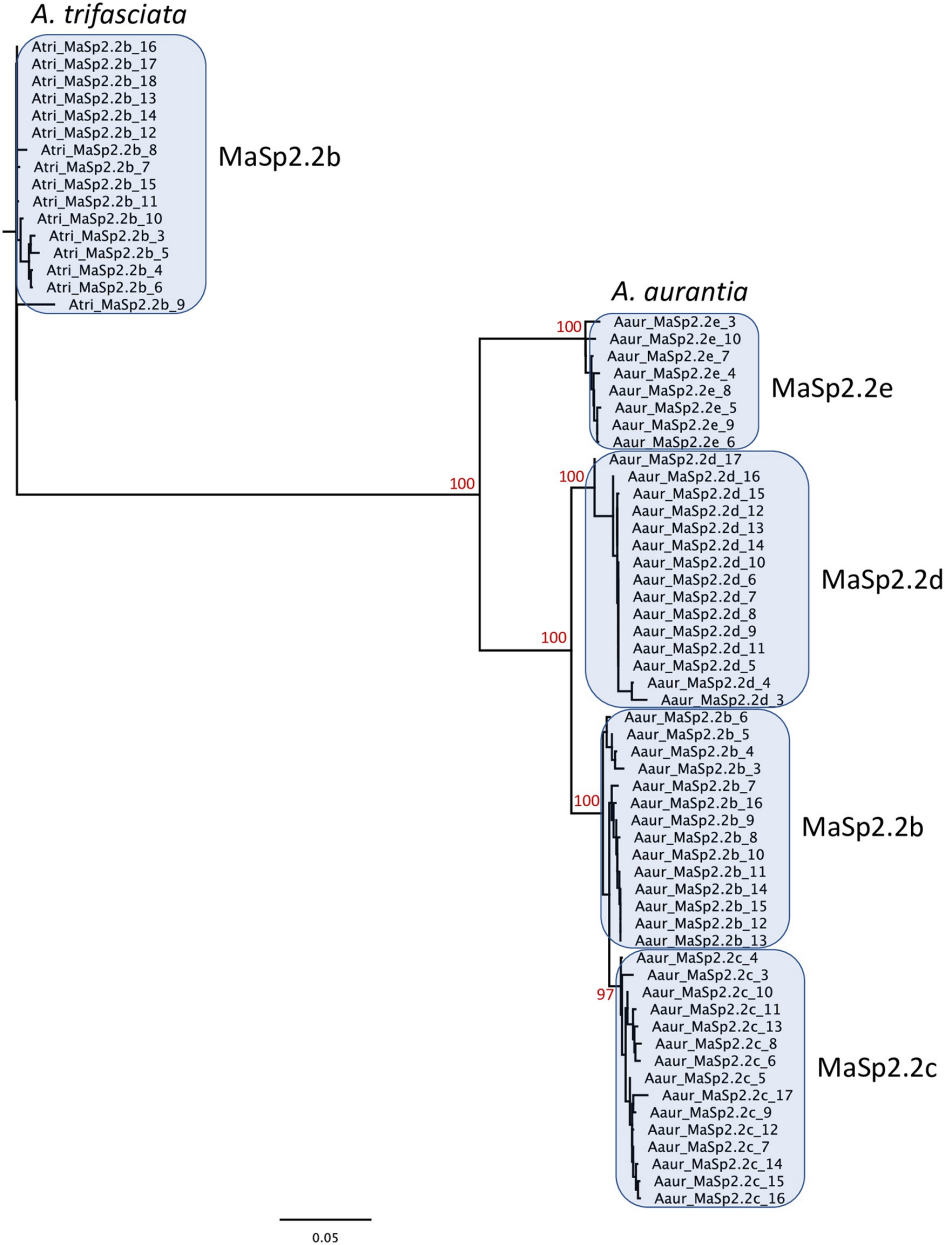
Sequence similarity among repetitive introns. Phylogenetic relationships among all the introns that belong to a specific homology group (introns of *A*. *aurantia MaSp2*.*2b*, *MaSp2*.*2c*, *MaSp2*.*2d* and *MaSp2*.*2e*, and *A*. *trifasciata MaSp2*.*2b*). Generally, introns from a given gene belong to a strongly supported clade (bootstrap values provided at the nodes) indicating strong homogenization of these sequences.

While most of the MaSp genes are expressed as a single long exon, the MaSp2 genes contain numerous exons and, in many cases, an extraordinary pattern of intron variation and homogenization. *MaSp2*.*2c* in *A*. *trifasciata* and all of the *MaSp2*.*1b* genes have one to three large introns in the 5’ region of the coding sequence and no other introns, but the other MaSp2s are organized in a highly repetitive exon/intron pattern in which nearly identical small introns are interspersed between similarly sized exons (Fig 2). Strikingly, some MaSp2 genes (*MaSp2*.*2a* in *A*. *argentata* and *A*. *aurantia* and all *MaSp2*.*1a* genes) exhibit an alternating pattern of intron types in which every other intron is similar in sequence (Fig 2). The exons of these genes are also organized in an alternating pattern such that two exons and two introns combined represent a core unit that is repeated throughout the sequence. The other MaSp2 genes contain a single repeated intron type (Fig 2). The iterated introns are highly homogenized in all genes (Figs 3, S2). The average pairwise similarity across all introns (excluding the first and last intron) within a given gene is 97.6% (range: 94.3–99.7%; S3 Table).

In addition to the high intronic sequence identity within a gene, there is a pattern of intron similarity across MaSp2 genes suggestive of shared evolutionary history or intergenic gene conversion. Within the MaSp2.2 genes there are 3 primary intron types whose grouping is largely consistent with the relationships among the termini sequences (Fig 1). *MaSp2*.*2a* of *A*. *argentata* and *A*. *aurantia* are clearly distinct from the other MaSp2.2 and the termini sequence also place these genes as the most divergent clade within the gene cluster. The other three MaSp2.2 genes of *A*. *argentata* share intron homology with each other and some similarity to the intron found in *MaSp2*.*2a* of *A*. *trifasciata*. Finally, introns from the remaining MaSp2.2 genes of *A*. *aurantia* and *MaSp2*.*2b* in *A*. *trifasciata* exhibit strong similarity (Fig 2). Overall, for these intron types, the level of similarity is greater within than between genes as introns from the same gene generally group together in phylogenetic analysis (Figs 3, S2). For the MaSp2.1 genes with repetitive introns, there is also strong identity both within a gene (S3 Table, Fig 2) and between species for the two intron types (S2 Fig). *MaSp2*.*1a* in *A*. *aurantia*, however, is enigmatic in that it contains a single repetitive intron in the 5’ half of the gene but a two-intron alternating pattern in the 3’ half of the genes (Fig 2). Given the strong similarity in intron sequence and organization between *A*. *argentata* and *A*. *trifasciata*, *A*. *aurantia MaSp2*.*1a* may be in the process of an evolutionary transition from a two intron to a one intron organization. All MaSp2.3 genes contain a single large intron that comprises between 46% (*A*. *trifasciata*) and 74.8% (*A*. *argentata*) of the gene.

### MaSp2 repeat organization is complex

#### Ensemble repeat organization

**Fig 4:**
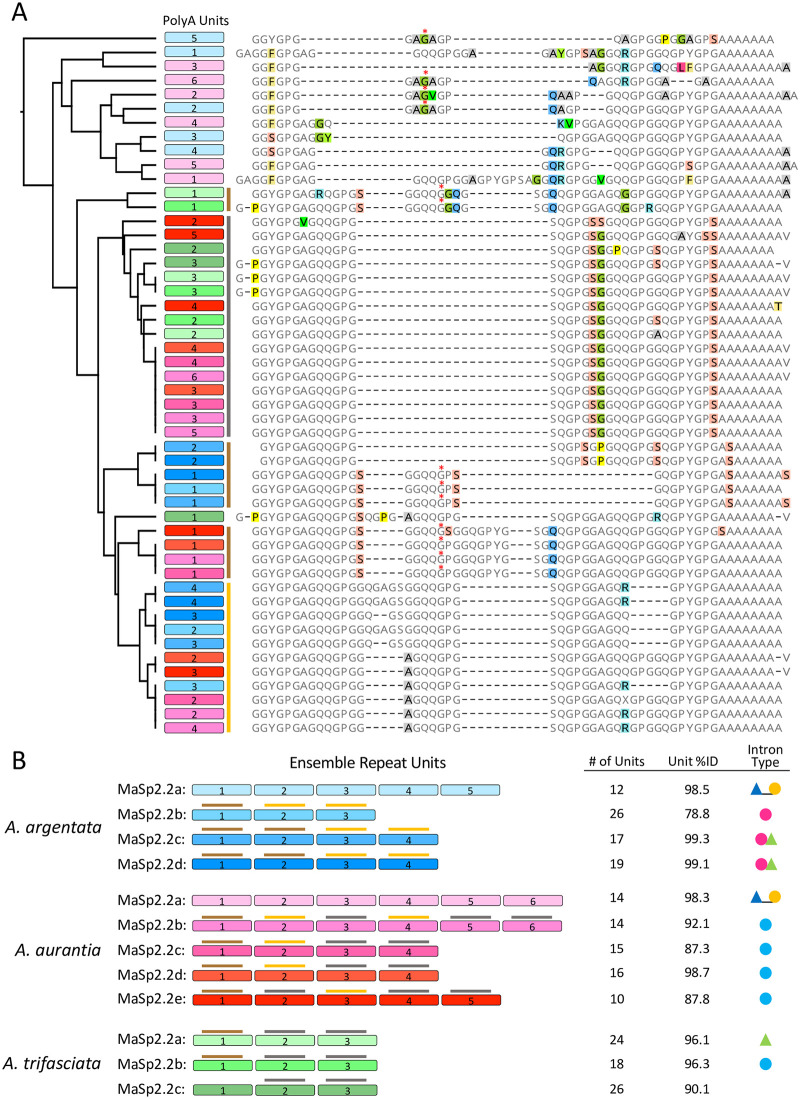
Variation in MaSp2.2 repeat structure. A) Alignment of the consensus poly-A units for each of the 12 MaSp2.2 genes. These amino acid sequences are generated by taking the consensus of all homologous poly-A units across all ensemble repeats within a given gene. Colored bars to the right of the poly-A boxes indicate the three poly-A types as determined by a phylogenetic analysis of nucleotide sequence (S7 Fig; e.g., the yellow bar refers to the consensus poly-A units defined by the yellow clade of S7 Fig). Red asterisks above the amino acid residues in some sequences show the intron locations. B) Depiction of the standard ensemble repeat unit organization for each MaSp2.2 paralog. The colors and numbers for each poly-A box correspond to those used in the protein alignment (A). The colored bars above some of the units indicate the poly-A type (S7 Fig). The total number of ensemble repeat units in each gene, the average percent identity among each unit within a gene and the intron type (colored shapes that correspond to those in Fig 2) are provided for each gene. Shapes separated by an underscore for intron type indicates that each alternating intron belongs to a different homology group.

MaSp2 genes are organized in a stereotypical pattern comprised of a core unit (hereby termed a ‘poly-A unit’) containing both a glycine-rich region (with characteristic motifs such as GPGXX and QQ) and a poly-alanine section. These poly-A units may be organized into larger ensemble units that repeat throughout the gene [47]. Reconstructing several full-length MaSp2 genes for multiple species provides an opportunity to examine the pattern of variation in ensemble, poly-A and motif identity and organization. As with the MaSp2 intron sequences, *Argiope* MaSp2 coding sequences are highly homogenized, more so than in the MaSp2 from any other species sequenced to date, but with substantial variation between genes, particularly in ensemble organization. For all MaSp2 genes with repetitive introns, the ensemble repeat unit is demarcated by an exon or pair of exons (when intron types alternate). There is some variation in the size of these ensemble units (in terms of the number of poly-A units contained within) among different MaSp2 paralogs but, generally, this organization is consistent within a gene. Overall, excluding the MaSp2.1b genes which don’t contain repetitive introns or consistent ensemble repeats, 83.5% of all ensemble units in the MaSp2 genes contain the same number of poly-A units as the most common ensemble unit within that gene. Fig 4 summarizes the variation found among ensemble and poly-A units across all MaSp2.2 paralogs. In this depiction, each poly-A unit represents the consensus sequence of all homologous poly-A units across all ensemble repeats within a given gene. Ensemble units range in size from 3 to 6 poly-A units and range in occurrence from 10 ensemble units within a gene (*A*. *aurantia MaSp2*.*2e*) to 26 units per gene (*A*. *argentata MaSp2*.*2b* and *A*. *trifasciata MaSp2*.*2c*) (Fig 4).

Measurement of protein similarity across ensemble units belonging to the same gene indicates extreme homogenization, with an average identity of 93.5% (range: 78.8–99.3%) for all the MaSp2.2 paralogues (Fig 4). MaSp2.1a genes exhibit similar levels of ensemble unit homogenization. In fact, *A*. *argentata* MaSp2.1a has the most extreme homogenization of any MaSp2 paralog with perfect identity among all ensemble units (each with three poly-A units) and *A*. *trifasciata* MaSp2.1a (with four poly-A units) is second at 99.7%. *A*. *aurantia* MaSp2.1a ensemble unit structure correlates with the intron/exon configuration within the gene. The 5’ half of the gene (containing a single repetitive intron, Fig 2) encodes an ensemble unit comprised of four poly-A units while the 3’ half of the gene (with alternating homologous introns, Fig 2) codes for a different ensemble unit with three poly-A units in most cases. While MaSp2.1b genes are not organized into homogenized ensemble units, the poly-A units from a given gene are generally more similar to each other than to the units belonging to the other two species (S3 Fig).

### Motif representation and amino acid composition

In terms of sequence composition across different poly-A units within and between paralogs (Fig 4), there is substantial variation in the size of the units with the largest unit (58 aa, the poly-A unit 1 of MaSp2.2b, c, d and e of *A*. *aurantia*) being nearly twice the size of the smallest unit (31 aa, *A*. *aurantia* MaSp2.2a unit 3). The protein variation is less dramatic as only 19 of the aligned 70 aa sites exhibit variants in which at least three sequences (out of 50) contain a variant amino acid (Fig 4A). As with the termini and intron sequence, the poly-A units of MaSp2.2a of *A*. *argentata* and *A*. *aurantia* are divergent from the other copies (Fig 4). Furthermore, the location of the intron in most of the MaSp2.2 paralogs occurs in a homologous region (within a glycine codon following a QQ motif in the poly-A unit 1 of each ensemble repeat) but the intron location for MaSp2.2a of *A*. *argentata* and *A*. *aurantia* exhibits no clear homology to the other sequences (Fig 4). Amino acid composition also distinguishes *A*. *argentata* and *A*. *aurantia* MaSp2.2a from the other paralogs. These genes have higher proportions of alanine and phenylalanine, and reduced serine and glutamine (S4 Fig; S4 Table). The elevated alanine results largely from smaller glycine-rich regions (an average of 28.3 aa compared to 38.9 aa in the other genes) such that the poly-A regions comprise a larger percentage of the total repetitive sequence. Overall, however, the amino acid composition among the MaSp2.2 copies is relatively stable (S4 Fig; S4 Table), with glycine content ranging from 37.1–40.8% and proline content between 12.7–15.9%. The MaSp2.1 genes have a similar composition to the MaSp2.2 copies except for an increased serine proportion (and concomitant reduced glutamine proportion) in the MaSp2.1b genes (S4 Fig; S4 Table).

The occurrence of stereotypical motifs (GPGXX, GGX and QQ) in the MaSp2 genes follows a pattern consistent with the amino acid content. Given the relatively high proline content in all of these genes, the presence of GPGXX is common, from 40–65% in all genes, while GGX motifs constitute approximately 20% of the protein sequence (S5 Fig; S4 Table). QQ motifs represent roughly 10% of the repetitive protein sequence but were greatly reduced in the few paralogs (*A*. *argentata* and *A*. *aurantia* MaSp2.2a and the MaSp2.1b genes) that have low glutamine content (S5 Fig; S4 Table). Despite the relative abundance of these motifs, they were not identified as the short sequences most highly overrepresented in the MaSp2.2 genes. Rather, GQQGPG, YGPG and QGP had the highest repeat occurrences relative to motifs of similar size. Among the 50 MaSp2.2 consensus poly-A units (Fig 4) GQQGPG was found 92 times and occurred three or more times in six of the poly-A units (S6 Fig; S5 Table). Given that this unambiguous motif involves the presence of amino acid residues before GPG (rather than after GPG in the case of GPGXX), we directly compared the total number of QQGPG motifs found within the MaSp2.2 gene relative to the most abundant unambiguous motif satisfying GPGXX. This motif, GPGAG, occurs approximately half as often as QQGPG (780 vs 1567), further supporting the potential significance of the GQQGPG motif. *A*. *aurantia* has the highest proportion of GQQGPG per poly-A unit (2.26 vs 1.88 in *A*. *trifasciata* and 1.44 in *A*. *argentata*). YGPG was the next most overrepresented motif and occupies a consistent location within the poly-A units. Allowing for slight variation in YGPG (an F in the first position and S in the last position), it is clear this motif is a highly conserved element occurring on both sides of the poly-A region (S6 Fig). The MaSp2.1 genes exhibit less complexity in stereotypical motifs, with GPG, GPS and GQG comprising the most overrepresented sequences (S5 Table).

We also examined homology among different poly-A units in order to understand the relationship among ensemble units across species and how variation in ensemble units has evolved. Given their high paralog diversity, we focused on the MaSp2.2 genes excluding the more divergent copies, MaSp2.2a of *A*. *argentata* and *A*. *aurantia*. The consensus nucleotide sequence of the ensemble repeat units of each gene was determined and then split into its component poly-A units. All consensus poly-A units were aligned, accounting for their amino acid translation, and a maximum likelihood (ML) tree reveals structure differentiating three primary clades of poly-A units (colored yellow, brown and gray; S7 Fig), supported by moderate bootstrap values (66 and 87). The distribution of the three poly-A types within each ensemble unit is shown in Fig 4 and provides a more detailed view of the homology across paralogs. The *A*. *aurantia* genes contain all three poly-A types while the genes for the other two species are comprised of just two types (but a different pair for each species), suggesting there were gains or losses of poly-A types within the gene family. For instance, variation in ensemble unit size across the *A*. *aurantia* copies is caused by the gain or loss of both the yellow and gray poly-A types. Comparison of every exon within these four *A*. *aurantia* genes (S8 Fig) further supports the interpretation that changes in the number of poly-A units is the primary mechanism driving ensemble unit variation. Among 225 variable aa sites (out of 350) in this alignment, 17 sites (7.56%) are amino acid differences, 45 (20%) are small indels, and 163 (72.45%) represent gain/loss of entire poly-A units.

### MaSp2 flanking sequences suggest frequent intergenic conversion

**Fig 5:**
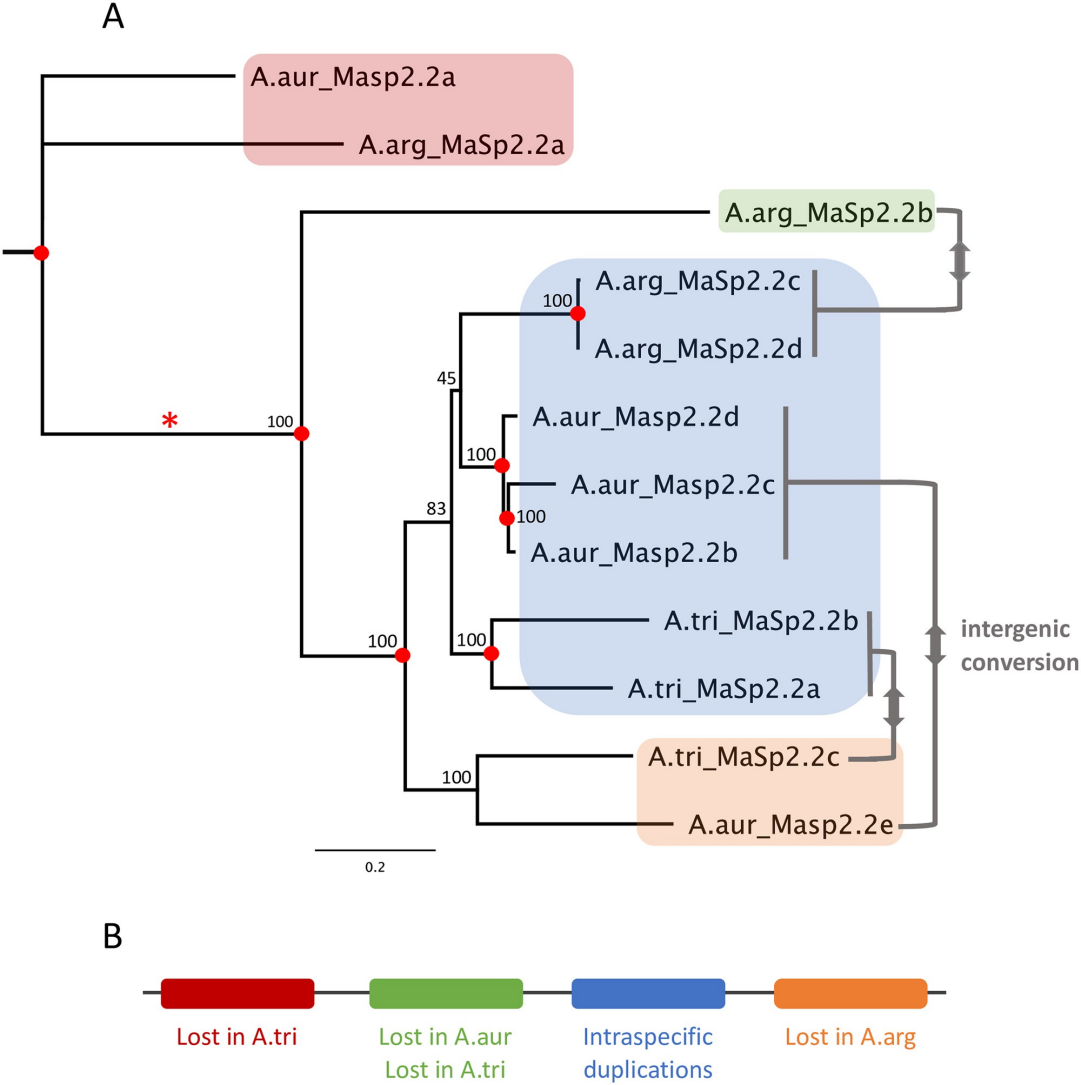
Gene gain/loss and intergenic conversion for *Argiope* MaSp2.2 genes. A) Phylogenetic relationships among MaSp2.2 genes based on flanking sequence. Approximately 1000 bp of sequence data directly upstream and downstream of the protein-coding sequence for each gene were concatenated and aligned to produce this topology. Nodes provide bootstrap support and duplication events (red circles). Asterisk indicates node discussed in Results. Putative intergenic gene conversion events for repetitive regions (gray bars) are summarized (see text for details). B) Putative ancestral gene organization for original genes from the four primary clades and gene gains or losses summarized for each taxon in each clade.

Because spidroin coding sequences are subject to numerous evolutionary forces (e.g., concerted evolution, replication slippage and selection) that obscure patterns of homology, examination of non-coding DNA that flanks these genes provides a valuable resource for understanding their evolutionary history. Phylogenetic analysis of a concatenated matrix of approximately 1000 bp each of sequence directly upstream and downstream of each MaSp2.2 gene produced a topology (Fig 5) divergent from results based on other data. Contrary to the signal provided by the terminal (Fig 1), intron (Fig 2) and exon (Figs 4, S7) sequences, *MaSp2*.*2b* in *A*. *argentata* does not group with *MaSp2*.*2c* and *MaSp2*.*2d* from this species and *MaSp2*.*2e* in *A*. *aurantia* does not group with *MaSp2*.*2b*, *MaSp2*.*2c* and *MaSp2*.*2d* from this species. Instead, *A*. *argentata MaSp2*.*2b* is in its own lineage while *MaSp2*.*2c* and *MaSp2*.*2d* are part of a strongly supported clade containing most of the MaSp2.2 genes from the other two species (Fig 5). In addition, *A*. *aurantia MaSp2*.*2e* and *A*. *trifasciata MaSp2*.*2c* form their own strongly supported clade separate from the other genes (Fig 5).

This topology suggests the presence of four primary gene groups within the MaSp2.2 cluster that have a complicated history of duplication, gene loss and intergenic conversion (Fig 5). The first group includes the *MaSp2*.*2a* genes from *A*. *argentata* and *A*. *aurantia*. Given the presence of paralogs from all three species in more derived clades, the origin of these MaSp2.2a genes occurred prior to the divergence of the three species, suggesting there was a loss of this gene in *A*. *trifasciata*. Incomplete species representation in both the second group, which includes just *A*. *argentata MaSp2*.*2b*, and the third group, comprised of *A*. *aurantia MaSp2*.*2e* and *A*. *trifasciata MaSp2*.*2c*, also implies gene losses in these clades. Finally, the fourth group resulted from a duplication prior to the species divergence but also involved independent within-species duplications in each species. More significantly, the strong similarity in sequence and gene organization among genes from the same species in the different groups suggests that several gene conversion events occurred between genes within a species that homogenized differences among paralogous copies (Fig 5). For instance, in *A*. *argentata*, *MaSp2*.*2b* has similar terminal (Fig 1), intron (Fig 2) and exon (Figs 4, S7) sequence to its adjacent paralogs, *MaSp2*.*2c* and *MaSp2*.*2d*, but the tree of non-coding flanking regions suggests this similarity is not the result of shared evolutionary history. Instead, if the flanking sequence topology is correct, the similarity in gene sequence within this species likely arose through conversion between *MaSp2*.*2b* and the other two genes. The directionality of the putative intergenic conversion events cannot be inferred from the tree, but the topology implies at least one instance of intronic conversion. The intron sequences present at the node uniting all taxa except the *MaSp2*.*2a* genes from *A*. *argentata* and *A*. *aurantia* (marked by a red asterisk in Fig 5) likely represented either the most common *A*. *aurantia* intron sequence (blue circle in Fig 2) or the most common *A*. *argentata* intron sequence (magenta circle in Fig 2). In the first case, the *A*. *argentata* intron sequence has converted between paralogs while in the second case, the *A*. *aurantia* intron sequence has converted between *MaSp2*.*2e* and the other four paralogs.

### MaSp1 and MaSp3 exhibit similar evolutionary dynamics

Relative to MaSp2, the repeat structure in MaSp1 and MaSp3, at all hierarchical levels, generally exhibits less diversity and complexity. Genes from both complexes contain two primary poly-A units that are usually organized into small ensemble units comprised of one of each poly-A unit type (S9 Fig), a pattern also found in *A*. *bruennichi* [48] and *Araneus ventricosus* [50], but, in some instances, that combine into larger units (S9 Fig; S6 Table). In *A*. *argentata* MaSp1, the poly-A pair has duplicated so that each ensemble unit contains two type1 and two type2 units in alternating placement, while several of the *A*. *aurantia* MaSp1a ensemble units contain an additional type1 poly-A unit (S9 Fig; S6 Table). Three of the MaSp3 genes also deviate from the most common simple organization. *A*. *aurantia* MaSp3b contains an ensemble unit with six poly-A units consisting of alternating type1 and type2 variants (S9 Fig; S6 Table). *A*. *trifasciata* MaSp3a is composed primarily of type1 units but has a type2 unit that occurs every 9–12 units suggesting the presence of the type2 unit may demark an ensemble unit. *A*. *trifasciata* MaSp3b has the most complex ensemble unit structure with two type1 and type2 variants organized in a 7-unit repeat (S10 Fig).

Consistent with other studies examining MaSp1 protein sequence [12,31,46,59], the *Argiope* copies are abundant in glycine and alanine but lack proline (S11 Fig). GGX motifs comprise, on average, 44.2% of the repetitive region, but GPGXX and QQ motifs are absent (S4 Table). GAG (24 additional occurrences) and QGG (8) are the most overrepresented motifs within the poly-A units. Overall, the MaSp1 poly-A units are highly conserved. For each type, there are few variable sites within *Argiope* and only a single amino acid difference between the *Araneus ventricosus* and the *Argiope* consensus sequences (S9 Fig). MaSp3 genes are intermediate between MaSp1 and MaSp2 in amino acid composition as they contain moderate proline abundance, and hence GPGXX motifs, but QQ motifs occur rarely (S4 Table). Arginine, serine and aspartic acid are found more frequently in MaSp3 than MaSp1 or MaSp2 (S4 Table), with a double serine (SS) often associated with the poly-A region (S9 Fig). GPG, GSG and GYG are the most overrepresented motifs within MaSp3, but each only occurs five additional times. The MaSp3 genes are also distinguished from MaSp1 and MaSp2 by the presence of a linker region within the repetitive sequence that occurs one or two times and has the consensus sequence KEIIKKIIVHRR (S10 Fig, S2 File). Analysis of the non-coding flanking sequences for the MaSp3 genes is more consistent with independent duplications of this gene in each species rather than an ancestral duplication as the two copies from the same species are monophyletic for both *A*. *argentata* and *A*. *aurantia* (S12 Fig). However, the lack of a sister relationship for the flanking regions of the two *A*. *trifasciata* paralogs suggests these genes may have a more complicated history.

### Non-canonical MaSps

Each *Argiope* genome contains three additional genes that exhibit homology to MaSp genes in the termini regions (Fig 1) but lack the stereotypical repeat structure of MaSp genes. These smaller genes (MaSp2.3, MaSp2.4 and MaSp6) do not contain poly-A regions and have more varied amino acid composition. The amino acids alanine, glutamine, glycine and proline comprise, on average, 82.6% of all archetypal MaSp1, 2 and 3 genes but represent 50.2% of MaSp2.3, 25.3% of MaSp2.4 and 40.1% of MaSp6. MaSp2.3 in *A*. *argentata* is roughly half the size of the MaSp2.3 genes in the other two species (904 aa vs. 1622 aa and 1713 aa) and contains a stop codon approximately 100 bp after the end of the conserved spidroin N-terminal region, suggesting this gene may be undergoing pseudogenization. Despite lacking poly-A regions, MaSp2.3 and MaSp6 possess repeat units that are similar across species but are not as strongly homogenized as the ensemble units in most of the other MaSp genes (S13 and S14 Figs). MaSp2.3 has an average within-species unit identity of 71.6% (*A*. *argentata*: 65.3%, *A*. *aurantia*: 73.5%, *A*. *trifasciata*: 75.9%), and MaSp6 has an average within-species unit identity of 79.0% (*A*. *argentata*: 66.7%, *A*. *aurantia*: 85.2%, *A*. *trifasciata*: 85.2%). MaSp2.4 contains no obvious repeat structure (S15 Fig).

### MaSp expression analysis

**Fig 6:**
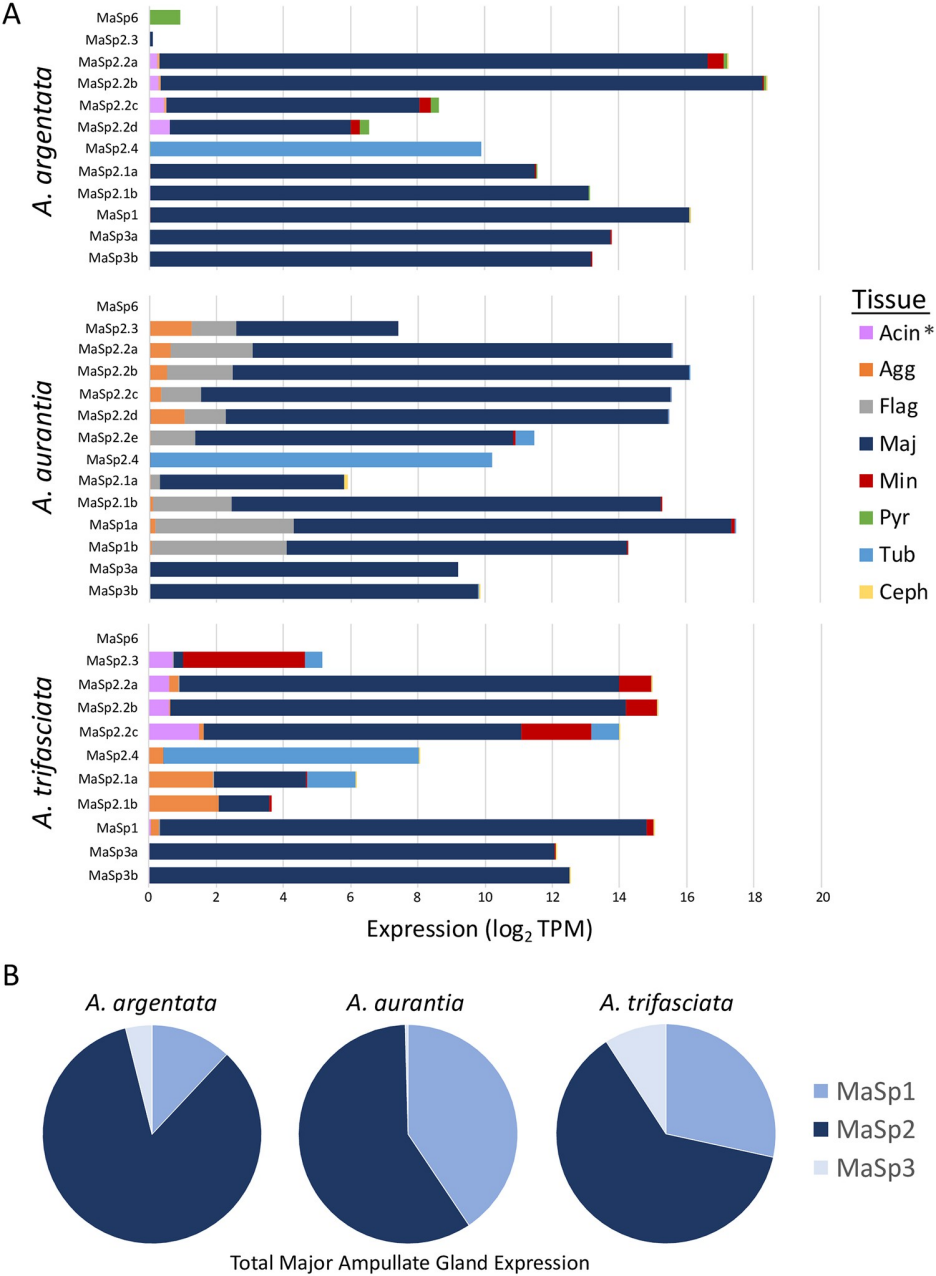
MaSp Gene Expression. A) Proportional gene expression in different tissues for each gene. Tissue abbreviations: Acin–aciniform gland, Agg–aggregate gland, Flag–flagelliform gland, Maj–major ampullate gland, Min–minor ampullate gland, Pyr–pyriform gland, Tub–tubuliform gland, Ceph–cephalothorax. Asterisk at Acin indicates that, for *A*. *aurantia* and *A*. *trifasciata*, this tissue is a combination of the aciniform and pyriform glands. B) Proportional gene expression summed across all paralogs of the three primary poly-A MaSp genes.

Most MaSp paralogs exhibit high expression levels within the major ampullate gland suggesting that they are likely to be functional (Fig 6a; S7 Table). The MaSp genes are the dominant transcripts within the major ampullate gland, representing approximately half of all expression within this tissue (62.3% of total transcripts per million (TPM) in *A*. *argentata*, 51.8% in *A*. *aurantia* and 48.4% in *A*. *trifasciata*). Of the three primary poly-A MaSp types, MaSp2 paralogs comprise the majority of total expression (Fig 6b). Minor differences exist among species in the relative expression for the different MaSp types and the extent of expression in silk glands other than the major ampullate gland. For example, several of the *A*. *aurantia* MaSp genes exhibit expression in the flagelliform gland while the MaSp2.1 genes in *A*. *trifasciata* have higher total expression in glands other than the major ampullate (Fig 6a). Both MaSp2.4 and MaSp6 appear to have shifted their function outside of the major ampullate gland as MaSp2.4 is expressed primarily in the tubuliform gland in all three species and MaSp6 is expressed just in the pyriform gland in *A*. *argentata* (pyriform gland expression was not measured in isolation for *A*. *aurantia* or *A*. *trifasciata*). In *A*. *argentata*, the two putative pseudogenes that are truncated and possess a stop codon, MaSp2.3 and a third MaSp3 paralog, exhibit either minimal (MaSp2.3) or no (MaSp3) gene expression (S7 Table).

### MaSp evolution within the Araneoidea

**Fig 7:**
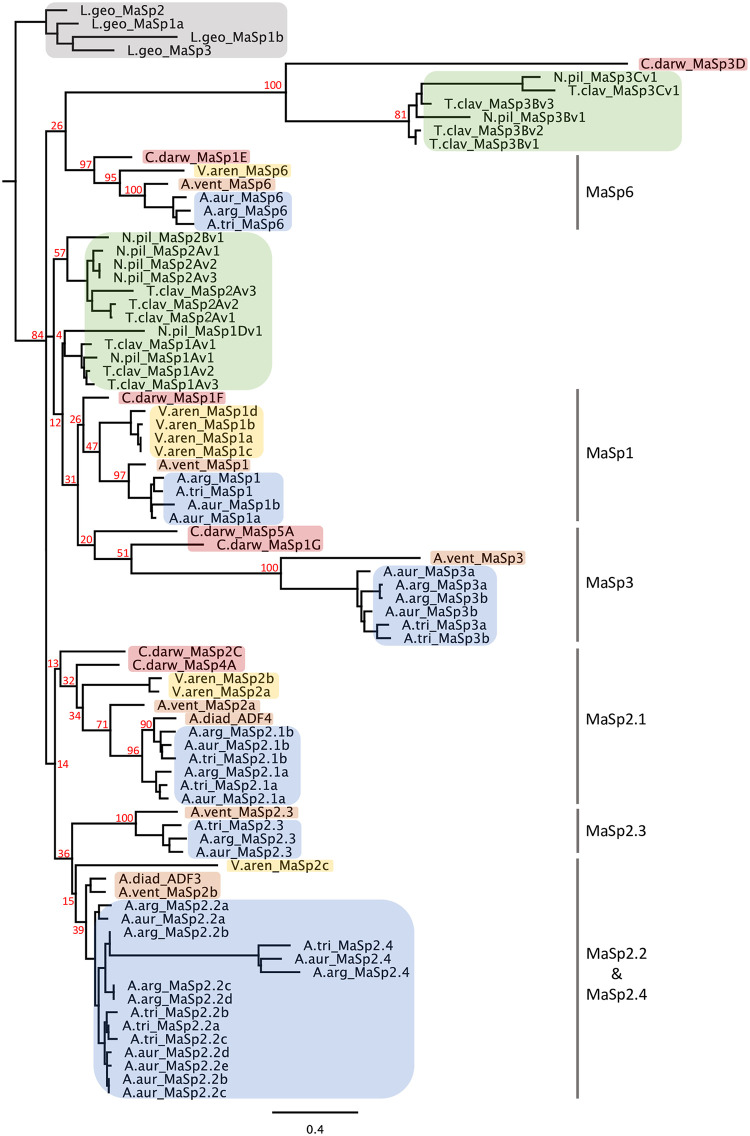
Phylogenetic relationship among MaSp genes within the Araneoidea. A ML tree of the concatenated protein sequences from the N- and C- terminal regions. Clade shading highlight different genera or families. A.diad–*Araneus diadematus*, A.vent–*Araneus ventricosus*, A.arg–*Argiope argentata*, A.aur–*Argiope aurantia*, A.tri–*Argiope trifasciata*, C.darw–*Caerostris darwini*, L.geo–*Latrodectus geometricus*, N.pil–*Nephila pilipes*, T.clav–*Trichonephila clavipes*, V.aren–*Verrucosa arenata*. Bootstrap values provided for most nodes. See S16 Fig for similar ML tree of Araneidae taxa based on N- plus C-termini DNA sequences.

To further explore the evolutionary diversification of the *Argiope* MaSp complex we examined the phylogenetic placement of these genes relative to other Araneoidea species that have been comprehensively sampled at the genomic level using both protein (Fig 7) and nucleotide (S16 Fig) sequences. As with previous analyses of spidroin termini sequences, paralogous MaSp copies from the same species often group together relative to spidroins from distantly related species. For example, all MaSp copies from the brown widow *Latrodectus geometricus* form a monophyletic group (Fig 7), a pattern that likely reflects intergenic gene conversion between the termini regions of paralogous copies [13,59,60], although convergent selection and recurring gene duplication may also play a role. The MaSp genes from araneid species exhibit relationships that are more consistent with species relationships [61]. Both trees (Figs 7, S16) place the origin of the two primary MaSp2 types (2.1 and 2.2) near the base of the araneid lineage as the node uniting these clades also includes genes belonging to *C*. *darwini* and *V*. *arenata*, which occupy a basal position in the family [61]. As with the analysis of *Argiope* paralogs alone (Fig 1), the topologies suggest MaSp3 arose from a duplication of MaSp1 but support for this relationship is weak (bootstrap values of 31% in Fig 7 and 39% in S16 Fig). Kono et al. [50,51] found all MaSp3 from the Nephilinae and other Araneidae were monophyletic but their analysis was based on 100 aa from the N-termini while our tree is derived from 250 aa from both termini. In addition, *C*. *darwini* possesses paralogs associated with both the Nephilinae MaSp3 clade and the other Araneidae MaSp3 clade supporting their potential independent origin. However, the amino acid structure of the MaSp3 repetitive regions in both the Nephilinae and other Araneidae have a similar and distinctive composition (high frequency of D and R) so it is possible gene conversion has occurred between the MaSp1 and MaSp3 genes at some point within the Araneidae. *C*. *darwini* MaSp4 is part of the MaSp 2.1 clade with moderate support (bootstraps 32% and 81%, Figs 7 and S16) but does share repetitive sequence similarities, such as a GPGPQ motif and a valine/serine-rich region [52,55], with *Argiope* MaSp2.3 (S14 Fig). As with the *Argiope* MaSp2.1b genes, *C*. *darwini* MaSp2 genes and *V*. *arenata* genes that are part of the MaSp2.1 clade (Fig 7) lack QQ motifs, suggesting that the absence of QQ may be the ancestral state for this clade. This pattern, combined with the presence of QQ motifs in *Latrodectus* and Nephilinae MaSp2 genes, suggests the evolution of QQ motifs in *Argiope* MaSp2.1a genes likely represents an independent and convergent acquisition of this molecular feature.

## Discussion

Dragline silk is a remarkable biomaterial essential to the evolutionary success of spiders and a paradigm for diverse man-made applications. Understanding the molecular and mechanical properties that provide dragline silk with its extraordinary combination of strength and extensibility has been the focus of substantial research [18,62]. Numerous studies have stressed the importance of silk’s hierarchical structure, oriented from amino acid motifs through nanocomposite networks to silk fibril interactions, in providing its noteworthy functional characteristics [63–67]. The predominant model of dragline silk performance and evolution [16,28,30,31,68] involves the combined functionality of two MaSp spidroins—MaSp1 and MaSp2—with each spidroin type providing different mechanical properties. In general, MaSp1 contributes to fiber strength while MaSp2 enhances extensibility and the performance of an individual dragline silk is determined, in part, by the relative proportion of each of these proteins in a given fiber. Recent genomic studies, however, have revealed substantially more genetic diversity, both in terms of copy number variation within the MaSp1 and MaSp2 archetypes and the presence of additional MaSp paralogs not accounted for by the traditional MaSp1-MaSp2 model [50–52,55]. It is likely that this increased molecular complexity impacts dragline function. In our survey of closely related *Argiope* species, we provide the most detailed comparative analysis of MaSp diversity and evolution to date and reveal a highly complex hierarchical pattern of molecular variation involving paralogous variants, diverse repeat unit structure and motif representation. This new understanding of MaSp genetic diversity requires a fundamental remodeling of the structure-function relationships that characterize dragline silk performance.

### MaSp gene diversity is shaped by complex evolutionary mechanisms

*Argiope* spiders exhibit high levels of variation in dragline silk performance profiles within the genus. In a study sampling several groups of spiders, Blackledge et al. [41] noted that variation in stress-strain curves within the genus spanned the range found within all other Araneoidea and RTA-clade spiders tested. For example, *A*. *aurantia* dragline silk is nearly three times tougher than *A*. *argentata* [40] and *A*. *lobata* exhibits mechanical performance closer to *Trichonephila* than to other *Argiope* species [37]. Here, we show that this diversity is mirrored at the genetic level with the three *Argiope* species examined in our study exhibiting the largest catalog of MaSp genes found in any spider species, all but of one of which are clustered in a single genomic region. While no single genome assembly reconstructed the entire cluster, scaffolds from the ONT and 10X assemblies in *A*. *argentata* could be merged to produce a single region spanning the entire cluster. Combination of the ONT and 10X scaffolds in *A*. *aurantia* and *A*. *trifasciata* yielded two breakpoints each that separated genes in the cluster, but all syntenic relationships were consistent with the organization in *A*. *argentata*. The MaSp gene cluster was also reconstructed in a genomic assembly of *Argiope bruennichi* [54], suggesting it is conserved in the genus. In addition, the recent genomic assembly of *C*. *darwini* includes a scaffold (Genbank accession BPLQ01015719.1) that contains representatives of MaSp1, MaSp2 and MaSp3 in an arrangement consistent with the *Argiope* organization, suggesting this MaSp cluster is ancestral for Araneidae, or perhaps an even larger clade of Araneae.

Comparison across multiple species indicates the MaSp genes in *Argiope* have a complex evolutionary history characterized by the prevalence of a birth-death mechanism involving frequent duplications and sequence divergence [69], intense intragenic homogenization, with occasional gene conversion and gene-loss events. Overall, the set of *Argiope* MaSp genes can be divided into seven distinct evolutionary lineages with several of these sub-groups containing *Argiope*-specific paralogs (Fig 1). MaSp2 genes have undergone the most extensive diversification with two main clades (genes containing poly-A units) that appear to have arisen with the Araneidae (Fig 7) but have reached their greatest diversity within *Argiope*. *A*. *aurantia* possesses seven of these MaSp2 paralogs, all of which exhibit high level of gene expression within the major ampullate gland (Fig 6). Similarly, both MaSp1 (in *A*. *aurantia*) and MaSp3 (in all three species) have experienced recent species-specific duplications. In *A*. *argentata*, the presence of a putative MaSp3 pseudogene and the likelihood that MaSp2.3 is also non-functional further highlight the high rate of gene turnover in this genus. The Araneoidea termini tree does not provide a clear picture of the pattern of diversification among the major MaSp types within this lineage, as there is low support for basal nodes, but does suggest the potential independent evolution of MaSp3 genes (Fig 7).

Within *Argiope*, the close similarity among termini regions of some MaSp2 paralogs within a species may not reflect recent common ancestry but, rather, intergenic conversion events. Phylogenetic signal in the flanking sequences strongly supports four primary MaSp2.2 gene clades that have each undergone some gene loss and/or gene conversion. Given the limited sampling of *Argiope* species, establishing the directionality of the conversion events is not possible, but the topology in Fig 5 implies not only the conversion of N- and C-terminal regions, but at least one conversion of intronic sequence. While concerted evolution has been implicated in previous studies of spidroin termini evolution [13,59,60,70], this is the strongest evidence thus far of intergenic conversion events impacting the repetitive regions. Understanding whether this within-species conversion is selected for because it improves fiber assembly and function when multiple spidroin proteins are integrated into a single fiber [71–74] or is the random outcome of frequent recombination within the cluster requires additional sampling and experimentation. In addition, it is important to note that the complex set of factors affecting MaSp sequence diversity (e.g., intergenic conversion, homogenization and selection) may distort the phylogenetic signal provided by various regions of these genes. Therefore, additional interspecific and intraspecific sampling is required to verify the patterns suggested by this analysis.

While the overall diversification of silk genes is well documented and clade-specific spidroin gains and losses have been found in other spider groups [51,52,56,60,70], the evolution of MaSp paralogs in *Argiope* signifies one of the most extreme cases of spidroin proliferation. What evolutionary forces are potentially responsible for this diversity? The fixation of gene duplicates occurs through drift or positive selection [75,76] and changes in gene expression levels associated with increased copy number is an immediate potential benefit responsible for duplicate fixation [77]. While buffering mechanisms often limit the change in expression levels that might be expected to result from having two gene copies, increasing dosage effects following duplication have been demonstrated in several studies [78–80]. In spiders, silk use clearly plays a central ecological role in their evolutionary success and requires high and continuous levels of protein synthesis. Spidroins are consistently among the most highly expressed genes in a given silk gland [49,50,52,81] and represent the majority of gene expression in the major ampullate gland of *Argiope*. Therefore, it is likely that selection favors genetic and cellular mechanisms that facilitate abundant gene expression, and increased MaSp copy numbers may be one pathway for achieving this goal. It is noteworthy that all of the recent MaSp paralogs in *Argiope* are organized in a tandem configuration because two recent studies [82,83] have found that tandem orientation boosts gene expression levels beyond the expected two-fold increase. However, why selection for increased gene expression might be greater in *Argiope* than other spiders is unclear. Previous studies have found that large body size, as found in *Argiope*, has evolved independently several times within orb-web weaving spiders, and larger webs have greater stopping potential, often with enhanced silk properties [84,85]. Producing enough silk for the construction of a large web within a short period of time may select for increased levels of gene expression. Within the limited sampling provided by this study, the largest spider, *A*. *aurantia*, has the most gene copies. Future studies will be required to determine any phylogenetic correlation between body size and MaSp copy number.

While selection for increased gene expression may drive the initial fixation of duplicate gene copies, the long-term maintenance and divergence of paralogs imply shifts in the functional roles of these genes. The functional differences between MaSp1 and MaSp2 are well established [18,30,62] and the increased use of MaSp2 is critical to the evolutionary success of orb-weavers [2,42,84]. Additional MaSp genes that likely impact fiber performance, such as MaSp3, have arisen within the Araneidae [50,52,55]. In this study, we show that the major MaSp2 lineages (MaSp2.1 and MaSp 2.2) appear to have diverged early in Araneidae evolution (Figs 6, S16) before undergoing additional duplications within *Argiope* that entail dramatic shifts in amino acid composition and motif representation. For instance, the amino acid composition variation between the two MaSp2.1 clades, and between the MaSp2.2a genes in *A*. *argentata* and *A*. *aurantia* and the other MaSp2.2 paralogs (Figs 3, S5 and S6) suggests these genes exhibit some tensile property differences. Functional benefits may arise from the presence of heterogeneity in silk fiber assembly that is tied to gene copy variation. A recent study [86] examining the mechanical properties of recombinant silk derived from the MaSp genes ADF3 and ADF4 in *Araneus diadematus* found that samples containing heterodimeric constructs (ADF3 and ADF4 repeat regions linked via C-termini) exhibited improved performance relative to homodimeric samples. ADF3 belongs to the MaSp2.2 clade and ADF4 is a MaSp2.1 paralog (specifically MaSp2.1b, Fig 7), indicating that despite their evolutionary divergence these proteins can interact to form complexes with emergent properties. Just like MaSp1 and MaSp2 proteins combine to produce a tougher fiber, different combinations of MaSp2 proteins that have molecular and performance differences may allow spiders to fine-tune the mechanical properties of dragline silk to match specific needs [33]. It is possible that, for *Argiope*, the divergent copies within each MaSp2 clade (MaSp2.1a and MaSp2.1b on the one hand and MaSp2.2a and MaSp2.2b-e on the other hand) have evolved as partners in heterodimeric complexes that can form easily due to termini sequence similarities and provide enhanced functional capabilities.

Similarly, at a more coarse-grained scale, the value of gene copy diversity may be related to benefits associated with heterogeneity in fibril structure and interactions. A silk fiber is composed of numerous fibrils running lengthwise along the fiber core [63,64,68]. These fibrils are not uniform and smooth but are characterized by an irregular globular surface structure, such that there is an interlocking friction among adjacent fibrils that increases fiber strength by reducing slippage and shearing in the latter stages of stress [63,65,87,88]. The mechanisms controlling the globular morphology of fibrils is not well understood, and variation in spinning behavior has been suggested as a potential factor [63]. While speculative at this point, variation in the molecular structures that comprise individual fibrils and fibril sets may also impact fibril surface heterogeneity. If this is the case, we would expect species that have a larger suite of MaSp gene copies that are integrated in a given dragline fiber type to exhibit higher fibril heterogeneity and for this increased heterogeneity to result in stronger silk fibers.

Ultimately, selection for increased MaSp diversity is expected to be tied to ecological and environmental variation. Intraspecific plasticity in dragline silk properties, ranging from difference among populations to temporal effects, has been documented in numerous studies [1,29,33,89–91]. At the molecular level, this variation is often associated with differences in the amino acid composition of dragline fibers [91,92]. Nutrition is the environmental variable that has received the most attention. Natural dietary variation or diet manipulation are often associated with changes in silk production, usually resulting in the down regulation of MaSp2 under low nutrient conditions because this spidroin type is thought to be more energetically costly than MaSp1 due to its high proline content [89–95]. Seasonality [93], humidity [96] and life stage [97] are additional factors that have been shown to be associated with variation in silk fiber properties or web building behavior. MaSp silk fibers are used in a range of roles such as the dragline, the web frame, the web radii and ballooning, but compositional differences among these utilities have not been investigated. These spidroins may also be incorporated, to some extent, into non-canonical roles (e.g., prey wrapping), and gene expression analyses from this study (Fig 6) and others [81,98] indicate MaSp expression is not limited to the major ampullate gland. Overall, selection is likely to favor the use of different MaSp types for different functional roles as well as the flexibility to alter silk fiber composition depending on specific ecological conditions. Future work will need to identify how the various *Argiope* MaSp spidroins are incorporated into different types of major ampullate silk fibers and how this genetic composition varies with divergent ecological uses, environmental conditions, and/or physiological variation that is specific to spiders in this genus.

### *Argiope* MaSp repeat organization has independently evolved hierarchical structure

Proteins that contain domains organized in a repetitive sequence are widespread throughout eukaryotes and carry out numerous functional roles [99–102]. The evolution of these proteins is often shaped by complex genetic mechanisms such as concerted evolution [69,103,104]. Spidroins are among the most repetitive proteins found in nature. Even for highly repetitive proteins, it is rare for the repeat region to comprise nearly all of the amino acid sequence [100], as is the case for spidroins. Similar to some other repetitive proteins [105–109], spidroin repetitive sequence is often organized in a hierarchical fashion with smaller repeats nested within a larger repeat unit [47,48,53,110]. In addition, spidroins generally exhibit extreme homogenization across repeat units [15,46,47,111–113]. Homogenization, however, is not a common attribute of repetitive proteins [99,101], so its ubiquitous presence in spidroins suggests that selection is shaping this organization to some degree. Analysis of spidroin repetitive structure has focused largely on the occurrence and distribution of small motifs, such as GGX and GPGXX [16,21–23], but the pattern of variation within the *Argiope* MaSp genes, particularly the diversification of the MaSp2 genes, highlights the potential significance of larger repetitive units. Overall, MaSp diversity in *Argiope* is characterized by remarkable hierarchical repeat organization and homogenization, suggesting these features may represent essential characteristics of dragline function in this genus.

In the MaSp2 genes of *Argiope*, this pattern is driven by a stereotypical, but rapidly evolving, intron-exon organization that arose within or just before the origin of this genus. All the MaSp2.1a paralogs and all but one of the MaSp2.2 paralogs have a gene structure characterized by repetitive exon-intron pairs in which the larger repetitive unit (i.e., ensemble repeat) is defined by one or two of these exons. Furthermore, this gene structure appears to have evolved independently in the two primary MaSp2 clades as none of the MaSp2 genes belonging to other Araneidae species contain repetitive introns (Fig 7). Despite this common organization, there is substantial diversity in ensemble repeat composition among different MaSp genes and, in some cases, within a gene. The relationship between exon-intron structure and ensemble repeat composition is most evident in *A*. *aurantia MaSp2*.*1a*, in which the gene is split into two regions with different intron types and ensemble repeat units associated with 5’ and 3’ regions of the gene. MaSp1 and MaSp3 also exhibit ensemble repeat structure, generally involving some combination of two primary poly-A types (S9 Fig), but without the presence of introns. Similar to the MaSp2 genes, variation in ensemble repeat structure can evolve rapidly. For example, *MaSp3a* and *MaSp3b* in *A*. *aurantia* are recent intraspecific duplicates and have substantially diverged ensemble repeat organization (composed of a two poly-A unit vs a six poly-A unit). The exact mechanisms, such as slipped strand mispairing or unequal crossing over, driving this ensemble repeat diversity, as well as the functional consequences of ensemble repeat structure to mechanical performance, are unclear at this time, but similar functional diversity of ensemble repeat structure has been found in mammalian genes [105]. The independent acquisition, and subsequent divergence, of MaSp genes with introns defining ensemble repeat boundaries, combined with their abundant gene expression, suggests this type of protein organization has adaptive significance. The vast majority of recombinant silk experiments have focused on the performance capabilities of a single poly-A unit [10,30,74,114–117], so future work should explore the mechanical properties of more complex repetitive units involving heterogeneous poly-A units. It will also be critical to survey additional araneid species to understand the full diversity and pattern of evolution in ensemble repeat structure.

Regardless of the adaptive significance of ensemble repeat structure, the acquisition of introns appears to impact the homogenization of these large repeat units within MaSp genes. Overall, these genes exhibit a remarkable pattern of intron diversity. The regular occurrence of introns that delineate repetitive protein-coding units has been found in several proteins [105,107,109,118,119]. Their presence often facilitates repeat unit duplication within a gene [119] and, in some cases, these introns are homogenized [120,121]. Repetitive, homogenized introns have been described in flagelliform spidroins [15], but *Argiope* is the first taxon in which repetitive introns in MaSp genes were found [122]. In addition, we are not aware of another example in any eukaryotic gene where two types of homogenized introns are organized in an alternating pattern as in the MaSp2.1a and MaSp2.2a genes. The MaSp2 introns also experience rapid turnover with minimal disruption of exon homology. For instance, in the *A*. *aurantia* and *A*. *argentata* MaSp2 genes with a single intron type, the poly-A units that contain the intron exhibit strong amino acid similarity between species (Fig 4) despite having highly divergent introns (Fig 2).

Relative to other araneid MaSp2 genes, the presence of introns in *Argiope* is associated with an increase in the stereotypical structure and homogenization of ensemble repeat units. For the intronless MaSp2 genes belonging to *Araneus ventricosus* and *V*. *arenata*, as well as the *Argiope* MaSp2.1b genes that have just one to three non-repetitive introns (Fig 2), there is little to no discernible regularity in the poly-A unit size or periodicity. As such, repetitive introns may be a mechanism that facilitates strong homogenization within a gene by limiting the pattern of recombination to specific regions of homologous sequence that promote unequal crossing over and increase inter-unit identity [121]. While most other spidroins, such as tubuliform and aciniform spidroins, maintain highly homogenized repetitive sequence without having introns, their repeats are often larger and more complex with greater amino acid diversity [70,111–113]. MaSp genes, like flagelliform spidroins, have relatively small repeat units dominated by codons for only a few amino acids (A, G, P, and Q). Under these conditions, replication slippage may be common, and unequal crossing over facilitated by repetitive introns, in combination with selection, might purge slippage mutations and homogenize the repeat units. Occasionally the mutational diversity driven by replication slippage may create a poly-A variant with a favorable characteristic, and this new unit could be incorporated into the ensemble repeats. If identical introns facilitate the homogenization of coding sequence within a given MaSp gene, they also provide an opportunity for intergenic exchange if there are multiple paralogs with similar introns. However, once these paralogs evolve functional differences that are critical to performance, intergenic exchange is likely to have negative fitness consequences, and selection will favor a rapid turnover in the intron sequences of one or both paralogs to minimize genetic exchange [123,124]. This process may explain the extreme sequence divergence found between introns of the *Argiope* MaSp2 gene copies. It must be noted, however, that some MaSp1 and MaSp3 genes possess strongly homogenized ensemble repeat units without introns. For these genes, it is possible that the relatively high divergence between the two primary poly-A types (S9 Fig) constrains recombination in a manner similar to repetitive introns. MaSp2 may also have evolved introns to allow for alternatively spliced transcripts, but this would likely only affect the length of a given protein, and not the structure, since all ensemble repeat units in a particular gene are essentially identical.

### *Argiope* MaSp motif composition is more varied than the classic MaSp1-MaSp2 model

At the molecular level, dragline silk is characterized by a distinct dichotomous organization, linked β-sheet nanocrystals surrounded by a coiled amorphous region, that is central to its mechanical performance. The crystalline structure primarily confers strength to the fiber and is encoded by stacked poly-alanine motifs while the amorphous regions provide extensibility and are determined by the glycine-rich sections of the protein [2,17,18]. Dragline silk evolution within the Entelegynae is marked by a dramatic increase in extensibility, likely driven by the increased incorporation of MaSp2 spidroins into the dragline fiber of Araneidae spiders [2,42,44]. For example, MaSp2 has been estimated to comprise approximately 20% of dragline fiber in *T*. *clavipes* [125], compared to nearly 40% in *A*. *bruennichi* [48]. The abundance of proline residues in MaSp2 proteins impacts dragline extensibility by increasing the secondary structure disorder in the amorphous region [26–28]. Several recent studies [23,96,126], however, have highlighted the role that other amino acids, such a glutamine and tyrosine, may play in dragline mechanical properties. Similar to results found for paralog and repeat unit diversity, motif composition within *Argiope* MaSp genes exhibits widespread variation, and dragline protein evolution in this group is characterized largely by change in the glycine-rich, amorphous region, with the recently diversified MaSp2.2 gene having the most complex motif structure.

Recent comparisons among Entelegynae spiders [32,127] indicate that *A*. *aurantia* has the lowest degree of crystallinity (the ratio between crystalline and amorphous phases) that results in elevated levels of nanostructure disorder. As a result, *A*. *aurantia* exhibits extreme supercontraction and therefore was chosen as the reference taxon for the “Spider Silk Standardization Initiative” [40]. Proline content is high in all *Argiope* MaSp2 paralogs and, given the large number of these genes in each species and their relatively high levels of expression, it is likely the overall proline content in *Argiope* dragline fibers is high relative to other species that possess fewer MaSp2 paralogs. It should be noted however that protein and amino acid content of fibers were not directly measured in this study and have not been quantified in any species with reference to the full suite of MaSp paralogs. The presence of proline residues within MaSp2 proteins has generally been identified and analyzed within the context of GPGXX motifs [16,21]. While this motif is common within all MaSp2 paralogs, analysis of overrepresented k-mers suggests a slight variant on this motif, GQQGPG, is more pronounced and may also have functional significance. In direct comparison of 5-mers, QQGPG occurs nearly twice as often in the *Argiope* MaSp2.2 as the most common GPGXX motif. The GQQGPG motif is not limited to *Argiope* but is found consistently in other MaSp2 genes. In a survey of repetitive sequence in Entelegynae taxa, Malay et al. [23] reported GQQGPG in the MaSp2 sequences from five genera that were examined (*Araneus*, *Argiope*, *Gasteracantha*, *Parawixia*, and *Tetragnatha*). It occurs an average of 87 times in each of the MaSp2 variants of *N*. *pilipes* and *T*. *clavipes* [51] and is present in some MaSp repeat sequence of *Uloborus diversus*, a distantly related cribellate orb-weaver [128]. Given its prominence in *Argiope* and widespread taxonomic distribution, GQQGPG may represent a central functional motif whose expansion is tied to changes in initial fiber crystallinity and disorder. Within *Argiope*, *A*. *aurantia* possesses the highest occurrence of GQQGPG, so it will be important for future experiments to assess to what extent the elevated disorder, extensibility, and supercontraction in this species results from this amino acid feature.

Though not normally discussed in combination with GPG, the QQ motif contained within GQQGPG has also been identified as a core amino acid feature of MaSp2 genes that was present in the common ancestor of the RTA clade and orb-weaving spiders [23,43]. It has been hypothesized that the function of this motif is related to intermolecular hydrogen bonding [23] and protein aggregation [129], but direct experimental evidence regarding its effects is lacking. Results from this study suggest a more complex relationship with MaSp2 protein structure than previously thought as several MaSp paralogs have lost the motif completely. The majority of paralogs in the Araneidae MaSp2.1 clade (Figs 7, S16) do not possess a QQ motif. Given its presence in the Theridiidae and Nephilinae, it is likely the motif was lost when the MaSp2.1 clade first arose within the Araneidae and then regained in some MaSp2.1 genes (*Argiope* MaSp2.1a and *Araneus ventricosus* MaSp2a). The lost glutamine residues in *Argiope* MaSp2.1b genes appear to be replaced largely by serine residues (S4 Fig) and the two *V*. *arenata* MaSp2.1 sequences (Fig 7) that lack QQ repeats also have a high serine composition (11.6% and 9.1%). A similar trade-off between glycine and serine was found across multiple spidroins spanning the Araneae [11]. Overall, given the dramatic shift in QQ motif representation within MaSp2 evolutionary history, it will be critical for future studies to isolate the functional properties of this motif.

Finally, tyrosine has also been shown to play a critical role in silk extensibility and supercontraction [96,126], and the second most over-represented k-mer amongst the *Argiope* MaSp2.2 genes was the motif YGPG (and its variant YGPS) that is consistently positioned on either side of the poly-A unit (S6 Fig). The association of tyrosine and GPG is common throughout the Araneoidea. In the Theridiidae and Nephilinae the majority of tyrosine residues are adjacent to a GP(G/S) motif (*L*. *hesperus*– 64.25%, *N*. *pilipes*– 51.49% and *T*. *clavipes*– 78.21%). This proportion increases to over 95% in two of the *V*. *arenata* MaSp2 genes and both of the *A*. *ventricosus* MaSp2 genes. Recent experiments examining changes in the microstructure of dragline silk fibers under stress suggest an increase in the crystalline phase as the fiber stretches [127,130]. This transformation does not appear to be tied to the poly-A nanocrystal sheets but results from the formation of new crystalline structures that provide additional strength during the latter stages of strain. The molecular mechanism responsible for this functional characteristic is unknown but Pérez-Rigueiro et al. [127] hypothesized that polyglycine II nanocrystals, tied to the motifs GGX and GPG, are the source of this increased crystallinity. Given the association of tyrosines to GPG and their proximity to the poly-A region it is tempting to speculate that this motif may play a role. It is also noteworthy that the functional significance of polyglycine II nanocrystals was first identified in flagelliform silk fibers [131] and the association between tyrosine and GPG is dominant in these genes. In the published flagelliform protein sequences for *N*. *pilipes*, *T*. *clavipes*, *A*. *ventricosus* and *A*. *bruennichi* 90% of all tyrosine residues precede GPG (range: 77.3%– 97.4%). As such, the functional significance of the YGPG motif may span multiple spidroin types and be integral to both supercontraction and a second crystalline phase in dragline fiber.

## Conclusions

The structure-function relationship defining dragline silk mechanical properties has been the subject of abundant research [2–5,9,10,18,20], but placing this knowledge within an evolutionary context has been limited by the lack of full length MaSp genes for closely related species. In this study, we provide a detailed examination of molecular variation across numerous aspects of MaSp gene composition including copy number, gene organization and flanking, terminal and repeat sequence. These comparisons highlight the abundant genetic diversity associated with MaSp genes, particularly in *Argiope*, and the varied evolutionary processes driving this diversity. Specifically, we find 1) the largest diversity of MaSp genes found in any spider. This gene expansion highlights the importance of MaSp2 evolution within orb-weaving spiders and the frequency of gene turnover for MaSp paralogs. We identify two primary clades of MaSp2 genes, MaSp2.1 and MaSp2.2, whose divergence occurred early in araneid evolution and that likely represent distinct and interacting components of dragline fibers. 2) A remarkable pattern of exon-intron structure that has evolved independently twice within the genus and rapidly changes structure and sequence. This gene organization appears to influence repetitive unit homogenization and ensemble repeat structure. Furthermore, the diversity and complexity of ensemble repeats in these spiders suggests they may influence dragline mechanical performance. 3) Multiple occurrences of intergenic gene conversion between silk gene paralogs and the first evidence that this process may influence repetitive gene composition. 4) Motif composition that suggests the evolution and function of the amorphous glycine-rich region of MaSp2 involves more than the distribution of GPGXX.

## Materials and methods

### Specimen collection and tissue dissection

Individual spiders were collected at the following locations: *A*. *argentata*—San Diego, CA in 2017, *A*. *aurantia*—near Savannah, GA in 2018, *A*. *trifasciata*—San Diego, CA in 2017. In addition to the three focal species, we sequenced genomes for the cob-web spider *Latrodectus geometricus* and the basal araneid [61] *Verrucosa arenata*, to provide additional outgroup taxa for phylogenetic analysis of *Argiope* spidroins. *L*. *geometricus* spiders were collected near Riverside, CA in 2018 and *V*. *arenata* spiders were collected in Lexington, VA in 2019. Mature spiders were housed at the American Museum of Natural History (AMNH) in ambient conditions until dissection. Spiders used for molecular work were humanely euthanized with carbon dioxide and then dissected for individual silk glands. Dissected tissue was flash frozen in liquid nitrogen and stored at -80°C until processed for DNA or RNA extraction.

### Genome sequencing and assembly

Genomic sequencing and assemblies for all three *Argiope* species in this study were conducted using both 10X Genomics Chromium and Oxford Nanopore PromethION technologies. *L*. *geometricus* was sequenced using 10X technology and *V*. *arenata* with PromethION reads. All genomic preps were conducted on dissected silk gland tissue from a single individual using the Gentra Puregene tissue kit (Qiagen cat 158667). DNA samples to be sequenced on the PromethION system were treated with the Circulomics short read eliminator kit (PacBio cat SS-100-101-01).

### 10X Chromium Genomics (10X)

Approximately 1 ug of DNA for each *Argiope* species, as well as for *L*. *geometricus*, was sent to the New York Genome Center (NYGC) where it was prepped with the Chromium Genome linked read kit (10X Genomics) and paired-end sequenced (150 bp) on a lane of an Illumina HiSeqX machine, producing 868.65 M reads for *A*. *argentata*, 800.00 M reads for *A*. *aurantia*, 837.23 M reads for *A*. *trifasciata* and 1500.05 M reads for *L*. *geometricus*. These reads were assembled at NYGC using Supernova v2.0.1 [132]. Assembly statistics for the resultant draft genomes are presented in S1 Table.

Given the barcoding of large molecules utilized in the 10X technology, we developed a protocol that uses this linked read information to identify genomic contigs that share barcodes and, therefore, are likely to be close to each other in the genome despite separate assembly. For each species, raw 10X read pairs were processed with the 10X Long Ranger v2.2.2 [133] BASIC pipeline that performs basic read and barcode processing including read trimming, barcode error correction and barcode whitelisting, prior to attaching the processed barcode sequences to each read as SAM tags in the description field of the FASTQ headers. Barcoded reads were then screened for duplicates, which were removed, and adapter sequences and low-quality bases were trimmed using cutadapt v.1.13 [134]. Finally, read pairs for which both ends were at least 50bp long after this process were further screened for spiked-in PhiX using the GEM mapper [135]. The resulting read sets were then mapped in paired-end mode against the corresponding species draft genomes with BWA-MEM v0.7.15 [136], ensuring that the barcode tags were appended to each SAM record by using the “-C” flag. SAM records were then coordinate sorted using the Picard toolkit v1.122 (https://broadinstitute.github.io/picard) and accordingly indexed. From the barcoded read alignments, molecules were defined based on clusters of proximal reads sharing the same barcode, using a maximum intra-molecule read alignment distance of 50kb, with the start and end coordinates of each molecule corresponding to the alignment start and alignment end of the left-most and the right-most reads in each cluster, respectively. The genome was then partitioned into segments in such a way that all bases from a given segment are overlapped by exactly the same molecules. For each segment, the list of molecule barcodes was retrieved, allowing to compute barcode similarity between any two query and target regions of the genome. Based on this information, we were able to infer genomic proximity and orientation between different contigs in the draft assemblies.

### Oxford Nanopore PromethION Technologies (ONT)

Approximately 5 ug of DNA for each species was sent to the DNA Technologies Core at UC Davis for long-read sequencing. *A*. *trifasciata* was sequenced on a single PromethION flowcell, producing a total of 62 GB of sequence. *A*. *argentata*, *A*. *aurantia* and *V*. *arenata* were all sequenced on two flowcells, producing a total of 91 GB (*A*. *argentata*), 86 GB (*A*. *aurantia*) and 79 GB (*V*. *arenata*) of data. For genome assembly, all reads for each species were first corrected using the long read self-correction step in Canu v2.1.1 [137] with default parameters except correctedErrorRate = 0.105. Corrected reads were then assembled using Flye v2.8 [138] with default parameters. All three *Argiope* assemblies produced genomes approximately 1.8 GB in size with N50 values ranging from 2.46 GB to .937 GB (S1 Table). The *V*. *arenata* assembly was slightly larger and more fragmented than the *Argiope* genomes (S1 Table). Genomic DNA used for ONT sequencing was also submitted to Novogene (Sacramento CA, USA) for library preparation and short read sequencing. Approximately 60 GB of 150 bp paired-end reads were generated from each sample for use in error correction.

### RNA sequencing and gene expression analysis

RNA-seq libraries, two or three replicates each, were constructed for up to eight tissues—aciniform, aggregate, flagelliform, major ampullate, minor ampullate, pyriform and tubuliform silk glands, and cephalothorax—in each *Argiope* species. For *A*. *aurantia* and *A*. *trifasciata*, the pyriform and aciniform glands were combined into a single library because the pyriform glands are challenging to isolate in dissections due to their small size. For *A*. *argentata*, anterior and posterior regions of the aggregate gland were dissected and sequenced separately but expression levels were averaged between the two sections for a single aggregate gland score. Similarly, in *A*. *aurantia*, the lateral and medial sections of the aciniform gland were dissected and sequenced separately but combined into a single aciniform gland score. RNA samples were prepped using the PureLink RNA Kit (Invitrogen) with bead mill homogenization and Trizol isolation and submitted to Novogene for library preparation and Illumina sequencing, with approximately 20 million 150 bp paired-end reads generated for each sample. All RNA-seq reads were trimmed of low-quality base pairs with Trimmomatic [139] and combined into a single transcriptome assembly using Trinity v2.9 [140]. Resulting contigs were blasted (blastx) against a custom arthropod protein database and TPM expression values for each library were calculated for all genes using RSEM [141]. Any gene without a TPM value greater than one in at least one library was excluded from the transcriptome set. Representative transcripts for each protein coding gene, including a single transcript for each spidroin N- and C-termini, were selected as part of the core transcriptome set for genome annotation. This transcript set was aligned to the ONT genome with gmap [142] to generate a .gff file that was used to calculate expression values for all gene regions using STAR v2.7 [143]. Proportional expression in each tissue relative to the total was calculated based on the ratio of tissue TPM to summed (across all tissues) TPM using non log-transformed TPM values and then applied to the log-transformed summed TPM score.

### Spidroin annotation and sequence analysis

To identify all potential MaSp gene copies, genomic contigs and scaffolds for each assembly were blasted against a custom database of published spidroin N- and C-termini sequences. For the 10X genomes, the contig nucleotide sequences adjacent to these hits were examined for MaSp repetitive DNA but, for nearly all hits, the assembled sequence was limited primarily to terminal regions with little to no repetitive sequence. The ONT assemblies, on the other hand, reconstructed full-length spidroins in the majority of cases. Therefore, all ONT contigs that had a spidroin hit were extracted from the genome and subjected to three rounds of short read error correction using minimap2 v2.17 [144] to align the short reads and Pilon v1.23 [145] to correct the sequences. To prevent artificial homogenization of the repeat nucleotides due to reads from dominant repeat sections aligning to and correcting variant repeat sections, Pilon only corrected frameshifts (with ‘—fix indels’ command) not basepairs. Intron/exon boundaries were identified by aligning RNA-seq reads generated from major ampullate tissue (see above) to the spidroin contigs with minimap2 [144] and viewing shifts in read mapping distribution in IGV [146]. Long reads were also mapped to contigs containing MaSp genes using minimap2 v2.17 [144].

### Motif identification and repeat analysis

Certain stereotypical motifs, such as GGX and GPGXX, have long been recognized as components of MaSp protein structure. In order to identify additional amino acid (aa) repeat motifs that populate these proteins and may be of functional significance, we searched consensus poly-A units (a sequence stretch comprised of one glycine-rich region and one poly-alanine motif) from paralogs belonging to a specific clade (e.g., all *Argiope* MaSp2.2 genes) for k-mers that were overrepresented relative to other k-mers of similar size. We scored the occurrence of all k-mers of size 3–10 aa across a given paralog group using a Teiresias algorithm (teiresias_v0.9.1 available https://cm.jefferson.edu/data-tools-downloads/teiresias/). As we were primarily interested in motifs that are found multiple times in a sequence rather than regions that are highly conserved across all sequences, we excluded any k-mers that occurred no more than once in all paralog sequences and scored k-mers relative to their multiple occurrences in a sequence. For the k-mers that occurred more than once in at least one sequence, we scored their relative overrepresentation as the total additional occurrences (i.e., the number of occurrences beyond the first one) of that k-mer divided by the total additional occurrences of all k-mers of that size. K-mers across all size ranges were compared to each other to determine the most overrepresented k-mer. That k-mer was then masked from all the paralog sequences and k-mer overrepresentation scoring was run again. The process was repeated until there was no k-mer that appeared more than 5 times.

### Phylogenetic analysis

Several phylogenetic trees were produced for termini, flanking, and repeat region sequences. Termini sequences consisted of the conserved spidroin amino- and carboxy-terminal regions that are hallmarks of the spidroin family [13]. Flanking sequences consisted of ~1000 bp upstream of the first Methionine codon and ~1000 bp downstream of the stop codon. Both nucleotide alignments (translated alignments for protein coding regions and standard alignments for flanking regions) and protein alignments were generated using MAFFT [147]. Maximum likelihood trees were calculated with PhyML [148] using the models GTR+I+G (nucleotides) and LG+I+G (amino acids), generally with 1000 bootstrap replicates.

## Supporting information

S1 Fig MaSp cluster genomic assembly organization. Location of genomic contigs containing the MaSp cluster genes for the 10X and ONT assemblies is indicated. Blue dots in between 10X contigs indicate regions where the two contigs were inferred to be adjacent via shared barcode information (see Materials and Methods). Dotted line regions between genes for *A*. *aurantia* and *A*. *trifasciata* indicate gaps in the assembly where there is no direct support for contiguity between genes. Scale bars provided for each species.
(TIF)

S2 Fig Phylogenetic analysis of MaSp2.2 and MaSp2.1 introns. The colored shapes correspond to those used in Fig 2. The taxa-gene numbers indicate the order of the intron in the gene (e.g., Aaur_MaSp2.2d_8 is the 8^th^ intron).
(TIF)

S3 Fig Phylogenetic relationships among the *Argiope* MaSp2.1b poly-A unit protein sequences. Colors highlight clades and lineages from different species.
(TIF)

S4 Fig Amino acid composition of repetitive regions of the *Argiope* MaSp2 genes. (TIF)

S5 Fig Motif frequency within repetitive regions of the *Argiope* MaSp2 genes. Bars represent the total proportion of nucleotides with the repetitive region of each gene that are represented by a given motif.
(TIF)

S6 Fig Occurrences of over-represented k-mers within the consensus poly-A sequences of the MaSp2.2 genes. Sequence names correspond to those used in Fig 4.
(TIF)

S7 Fig Phylogenetic relationships among the *Argiope* MaSp2.2 consensus poly-A protein sequences. Two genes, *A*. *argentata MaSp2*.*2b* and *A*. *aurantia MaSp2*.*2c*, had a variant ensemble repeat (the *A*. *argentata* variant contains five poly-A units and the *A*. *aurantia* variant contains six poly-A units) that occurred more than four times within the gene so the consensus sequence for both the common ensemble unit (Conseq1) and the variant ensemble unit (Conseq2) was determined and analyzed separately. The first poly-A unit of the *A*. *trifasciata* MaSp2.2c gene (the one MaSp2.2 gene lacking a repetitive intron) had ambiguous signal and was excluded from the analysis. Three primary clades are highlighted. Clade colors correspond to the poly-A unit colors presented in Figs 4 and S8.
(TIF)

S8 Fig Variation in MaSp2 repeat structure. Alignment (A) and phylogenetic relationships (B) among the *A*. *aurantia* MaSp2.2 exons (not including MaSp2.2a). Poly-A unit types within the exons are represented by different colors that correspond to colors presented in Figs 4 and S7. Numbers at nodes are bootstrap values.
(TIF)

S9 Fig MaSp1 and MaSp3 repeat structure. Alignment and phylogenetic relationships among consensus poly-A units of MaSp1 (A) and MaSp3 (B) for *Argiope* species and *Araneus ventricosus*. C) Phylogenetic analysis of combined protein matrix of MaSp1 and MaSp 3 consensus poly-A units. Given the close phylogenetic relationship between MaSp1 and MaSp3 genes (Fig 1) and the similarity in their ensemble repeat structures, we wanted to assess if there was shared homology between the poly-A types for each gene. However, the tree does not support this hypothesis as all the MaSp1 poly-A units group in one clade and all but one of the MaSp3 poly-A units group in a separate clade. Bootstrap value separating the two primary clades presented.
(TIF)

S10 Fig *A*. *trifasciata* MaSp3b repeat structure. Phylogenetic relationships (A) and repeat arrangement (B) among poly-A units. The taxa-gene numbers indicate the order of the poly-A unit in the gene. Colored blocks in the gene schematic correspond to poly-A units that belong to the same-colored grouping on the phylogeny (bootstrap values provide for these 4 nodes). B) Ensemble repeats with stereotypical structure indicated by red lines above several seven-block sections. N and C blocks at each end of the gene indicate the N- and C-terminal regions, and the thick line after the first eight colored blocks indicates linker sequence (see Results for description).
(TIF)

S11 Fig Amino acid composition of repetitive regions of the *Argiope* MaSp1 and MaSp3 genes. In the legend, ‘O’ indicates other amino acids not listed in legend.
(TIF)

S12 Fig MaSp3 flanking tree. Phylogenetic tree of flanking sequence (approximately 1000 bp of upstream and downstream nucleotide data combined) for the MaSp3 genes. Bootstrap values provided above the nodes.
(TIF)

S13 Fig Alignment and phylogenetic relationships among repeat units in *Argiope* MaSp2.3. Colored amino acids indicate variable sites relative to the consensus sequence.
(TIF)

S14 Fig Alignment and phylogenetic relationships among repeat units in *Argiope* MaSp6. Colored amino acids indicate variable sites relative to the consensus sequence.
(TIF)

S15 Fig Alignment of MaSp2.4 amino acid sequences among three *Argiope* species. (TIF)

S16 Fig Phylogeny of MaSp paralogs in the Araneidae. Derived from the concatenated nucleotide sequence of N- and C-terminal regions. Clade shading highlight different genera. Bootstrap values provided for nodes at generic level and above. A.diad–*Araneus diadematus*, A.vent–*Araneus ventricosus*, A.arg–*Argiope argentata*, A.aur–*Argiope aurantia*, A.tri–*Argiope trifasciata*, C.darw–*Caerostris darwini*, V.aren–*Verrucosa arenata*.
(TIF)

S1 Table Sequencing and genome assembly statistics. (XLSX)

S2 Table Long read coverage of assembled MaSp genes. For each MaSp gene, the average length of the ONT reads that map to that location is provided as well as the total basepair coverage of all ONT reads across the length of each gene. Species names abbreviated as A.arg–*Argiope argentata*, A.aur–*Argiope aurantia*, A.tri–*Argiope trifasciata*.
(XLSX)

S3 Table MaSp intron homogenization. Average pairwise sequence similarity among all repetitive introns that belong to a homology group for each gene. For genes with alternating introns the pairwise sequence similarity is calculated separately for first and second type.
(XLSX)

S4 Table Motif and amino acid representation for MaSp genes. Calculated based on the repeat regions (termini excluded) of all *Argiope* poly-A MaSp genes.
(XLSX)

S5 Table Measurement of k-mer overrepresentation within each MaSp gene group. The four gene groups are MaSp1, MaSp2.1, MaSp2.2 and MaSp3 (results in different tabs). ’Occurrence’ indicates the total number of times the k-mer is found across all consensus poly-A units. ’Add_occ’ indicates the number of occurrences within the sequences that represent multiple occurrences. ’Total kmer Add_Occ’ is the sum of all additional occurrences for k-mers of that length. ’Rel. Add_Occur’ is calculated by dividing ’Add_occ’ by ’Total kmer Add_Occ’.
(XLSX)

S6 Table Ensemble repeat structure of MaSp1 and MaSp3 genes. ’ER size’ indicates the most common ER in terms of number of poly-A units. ’ER regularity’ provides the proportion of total ER units that have the most common size (e.g., a value of 1 means all ER units are the same size). ’Ave %_ID’ indicates the average percent amino acid identity for homologous poly-A units across all ER units.’% Repeat’ indicates the percentage of the total protein occupied by stereotypical ER units.
(XLSX)

S7 Table MaSp replicate gene expression. TPM values of tissue replicates for all MaSp genes. Each species’ values are presented in a separate tab.
(XLSX)

S1 File Amino acid translations of all *Argiope* MaSp gene coding regions. (DOCX)

S2 File MaSp repeat structure. Poly-A containing *Argiope* MaSp proteins broken down by poly-A units. In most genes, ensemble repeats are evident as a repeating set of poly-A units of stereotypical size. Linker regions in MaSp3 genes presented as lowercase residues.
(DOCX)

S3 File Alignments of sequence data used to generate trees in Figures. (TXT)
