# Supplementary figures and images for: Rapid molecular diversification and homogenization of clustered major ampullate silk genes in Argiope garden spiders
Source: PLoS Genet. 2022 Dec 12;18(12):e1010537. doi: 10.1371/journal.pgen.1010537 (PMC9779670; doi:10.1371/journal.pgen.1010537)

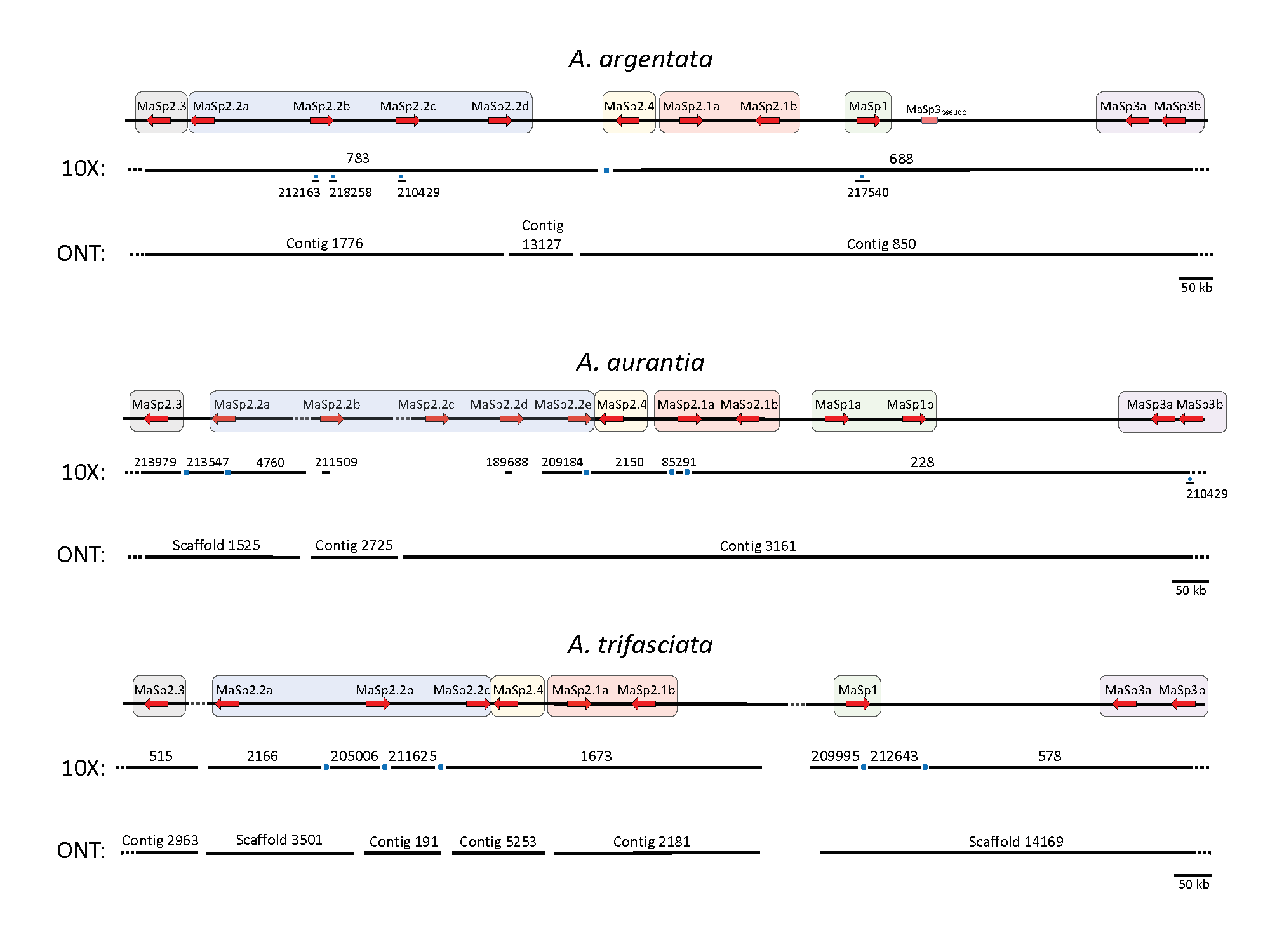

Supplement: S1 Fig — Location of genomic contigs containing the MaSp cluster genes for the 10X and ONT assemblies is indicated. Blue dots in between 10X contigs indicate regions where the two contigs were inferred to be adjacent via shared barcode information (see Materials and Methods). Dotted line regions between genes for A. aurantia and A. trifasciata indicate gaps in the assembly where there is no direct support for contiguity between genes. Scale bars provided for each species. (TIF) [file pgen.1010537.s001.tif]

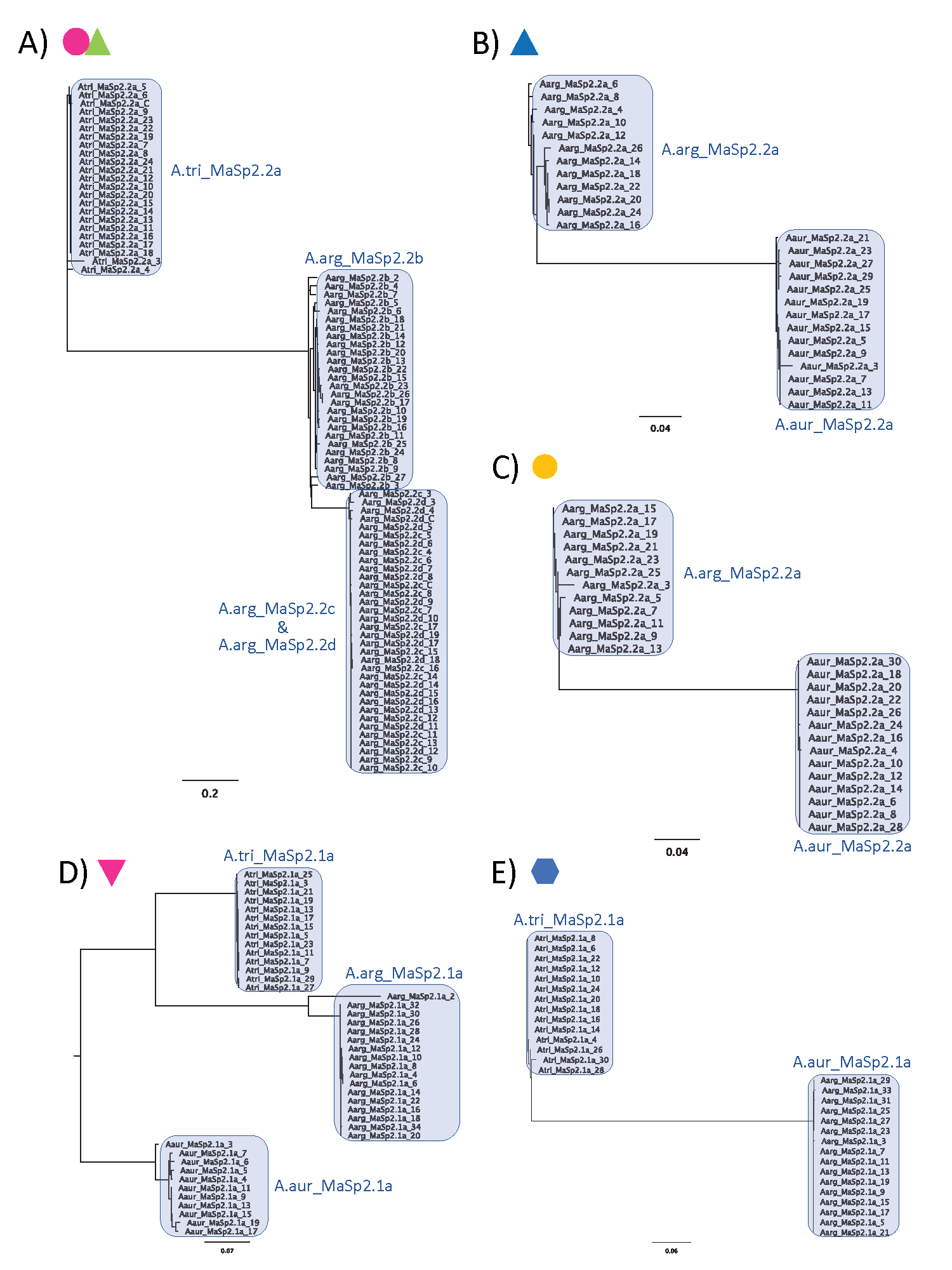

Supplement: S2 Fig — The colored shapes correspond to those used in Fig 2. The taxa-gene numbers indicate the order of the intron in the gene (e.g., Aaur_MaSp2.2d_8 is the 8th intron). (TIF) [file pgen.1010537.s002.tif]

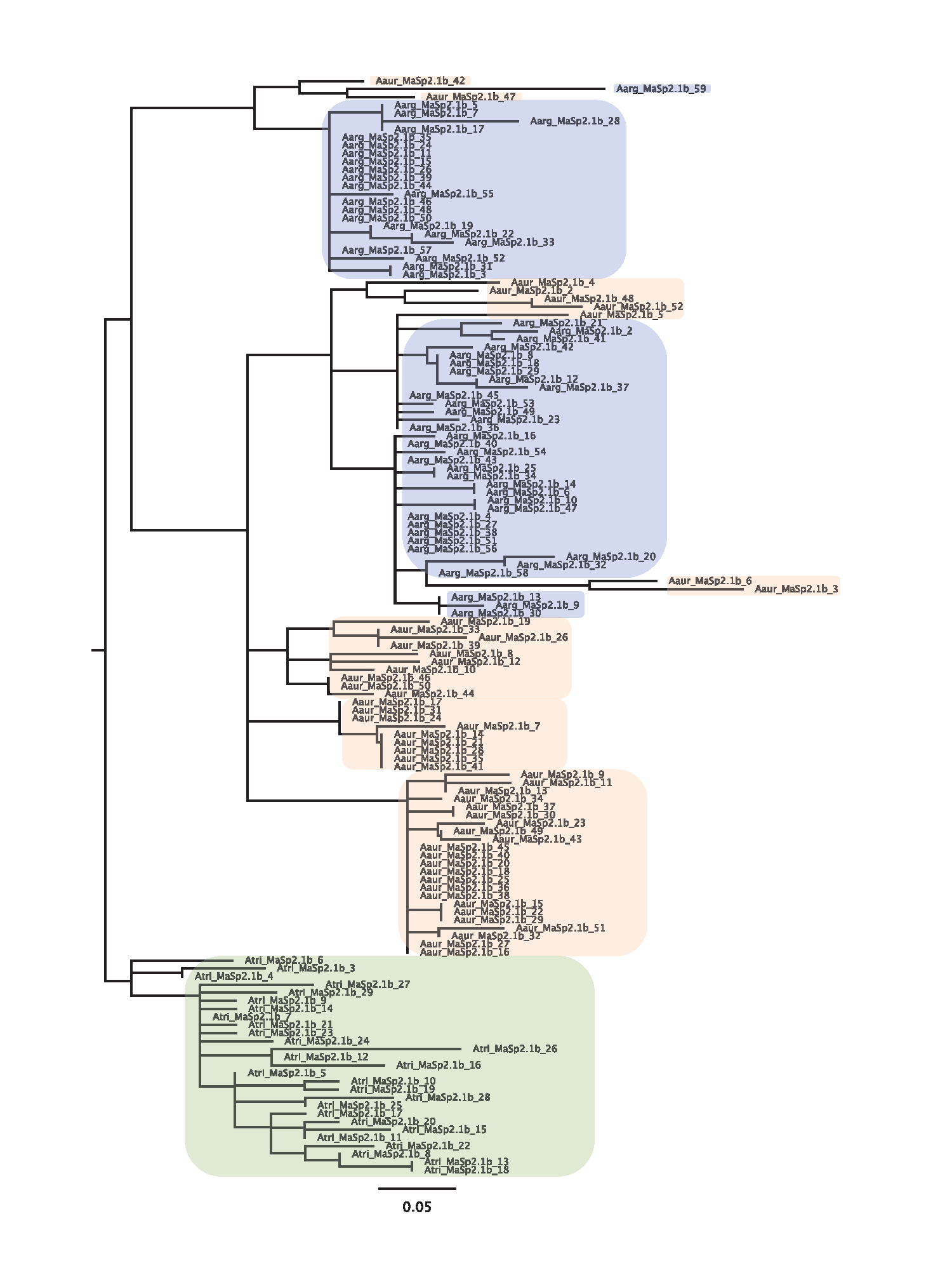

Supplement: S3 Fig — Colors highlight clades and lineages from different species. (TIF) [file pgen.1010537.s003.tif]

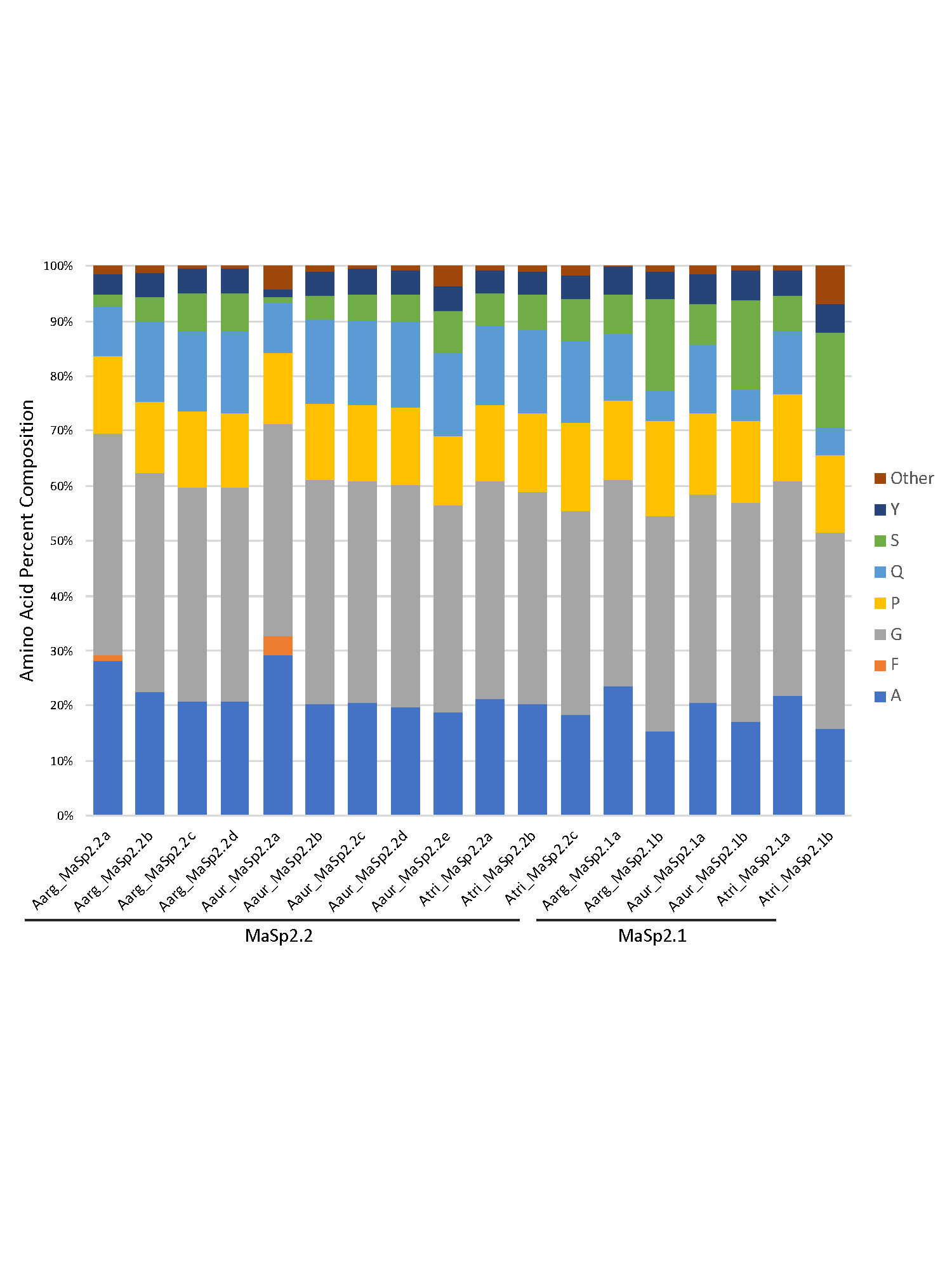

Supplement: S4 Fig — (TIF) [file pgen.1010537.s004.tif]

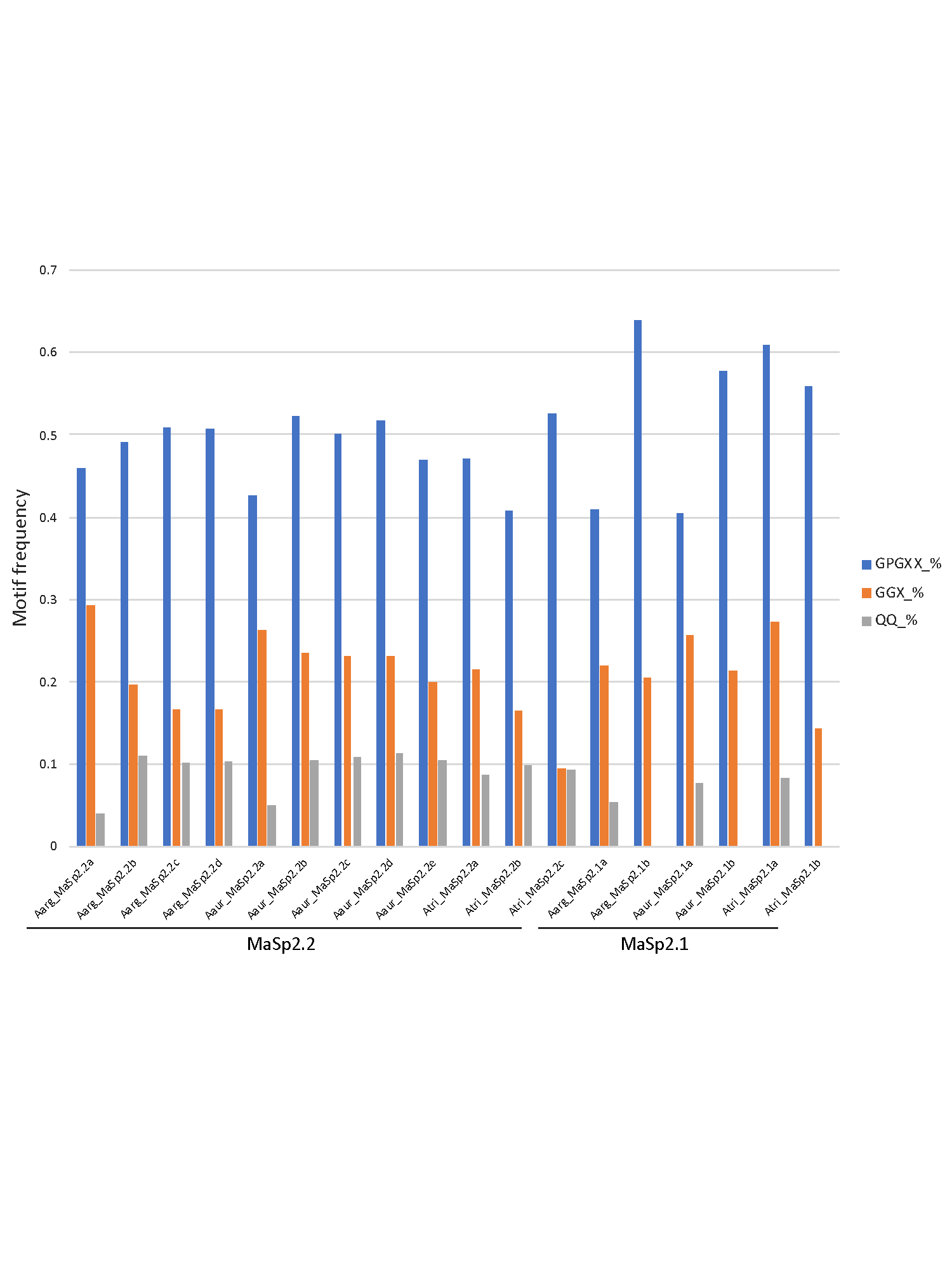

Supplement: S5 Fig — Bars represent the total proportion of nucleotides with the repetitive region of each gene that are represented by a given motif. (TIF) [file pgen.1010537.s005.tif]

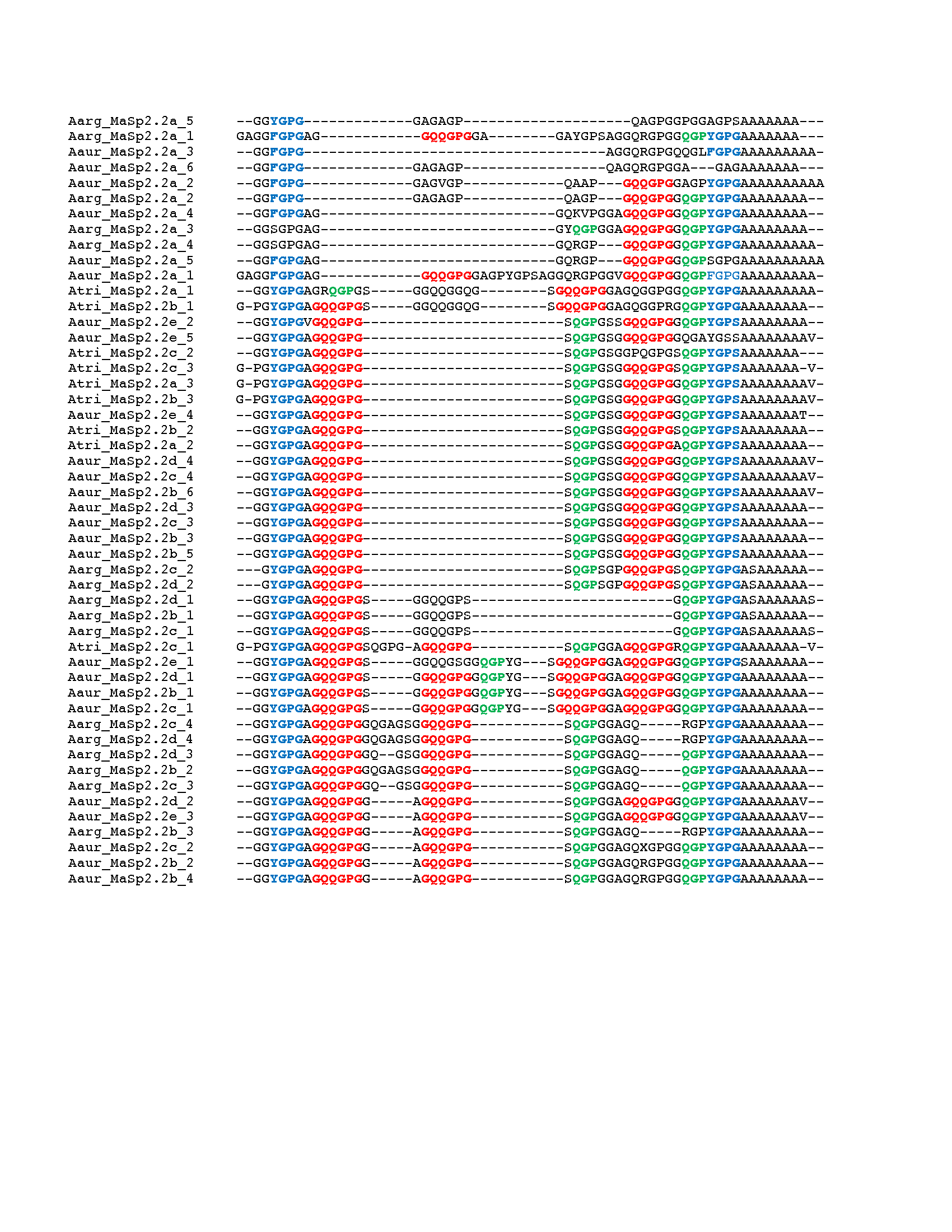

Supplement: S6 Fig — Sequence names correspond to those used in Fig 4. (TIF) [file pgen.1010537.s006.tif]

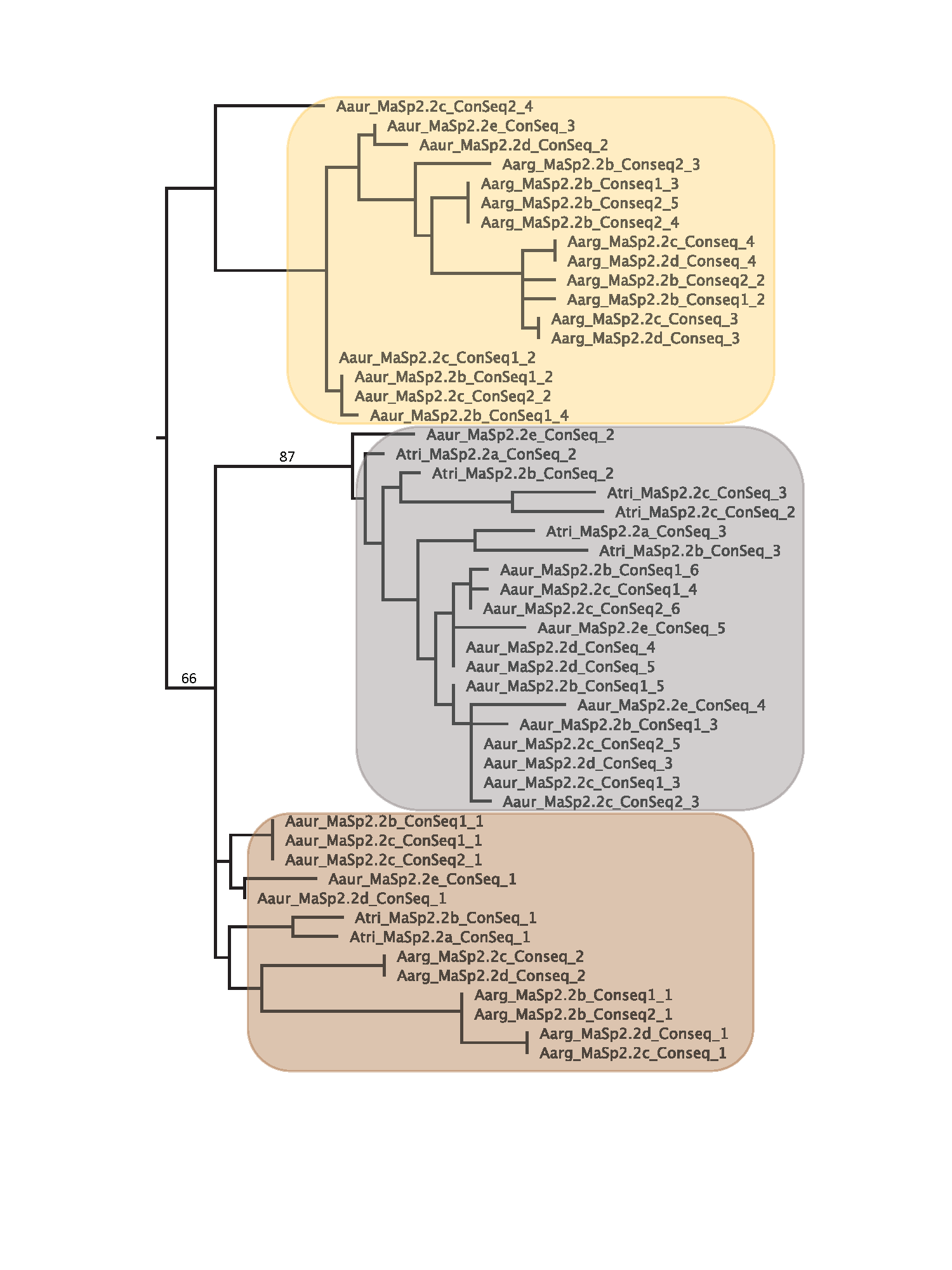

Supplement: S7 Fig — Two genes, A. argentata MaSp2.2b and A. aurantia MaSp2.2c, had a variant ensemble repeat (the A. argentata variant contains five poly-A units and the A. aurantia variant contains six poly-A units) that occurred more than four times within the gene so the consensus sequence for both the common ensemble unit (Conseq1) and the variant ensemble unit (Conseq2) was determined and analyzed separately. The first poly-A unit of the A. trifasciata MaSp2.2c gene (the one MaSp2.2 gene lacking a repetitive intron) had ambiguous signal and was excluded from the analysis. Three primary clades are highlighted. Clade colors correspond to the poly-A unit colors presented in Figs 4 and S8. (TIF) [file pgen.1010537.s007.tif]

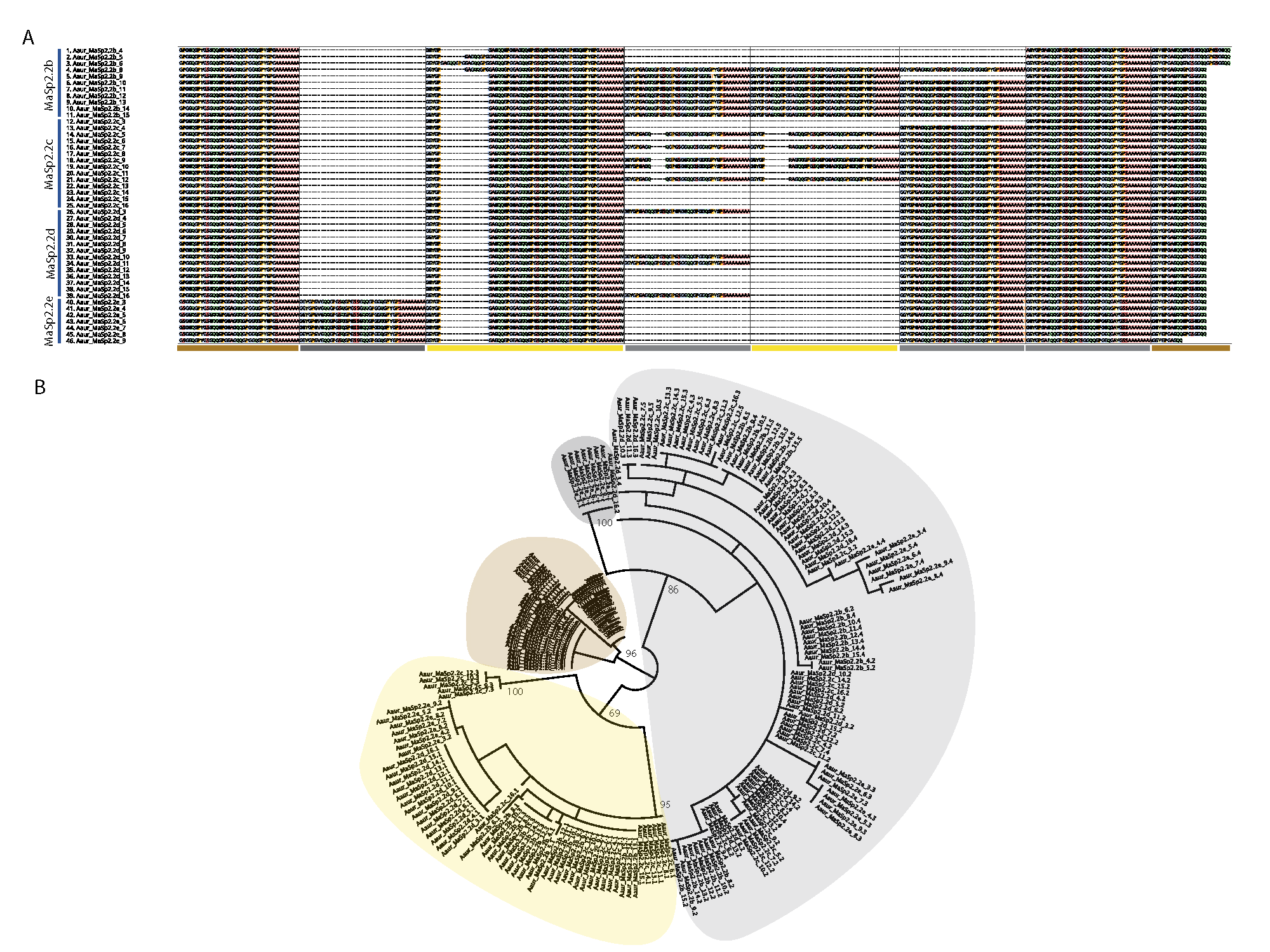

Supplement: S8 Fig — Alignment (A) and phylogenetic relationships (B) among the A. aurantia MaSp2.2 exons (not including MaSp2.2a). Poly-A unit types within the exons are represented by different colors that correspond to colors presented in Figs 4 and S7. Numbers at nodes are bootstrap values. (TIF) [file pgen.1010537.s008.tif]

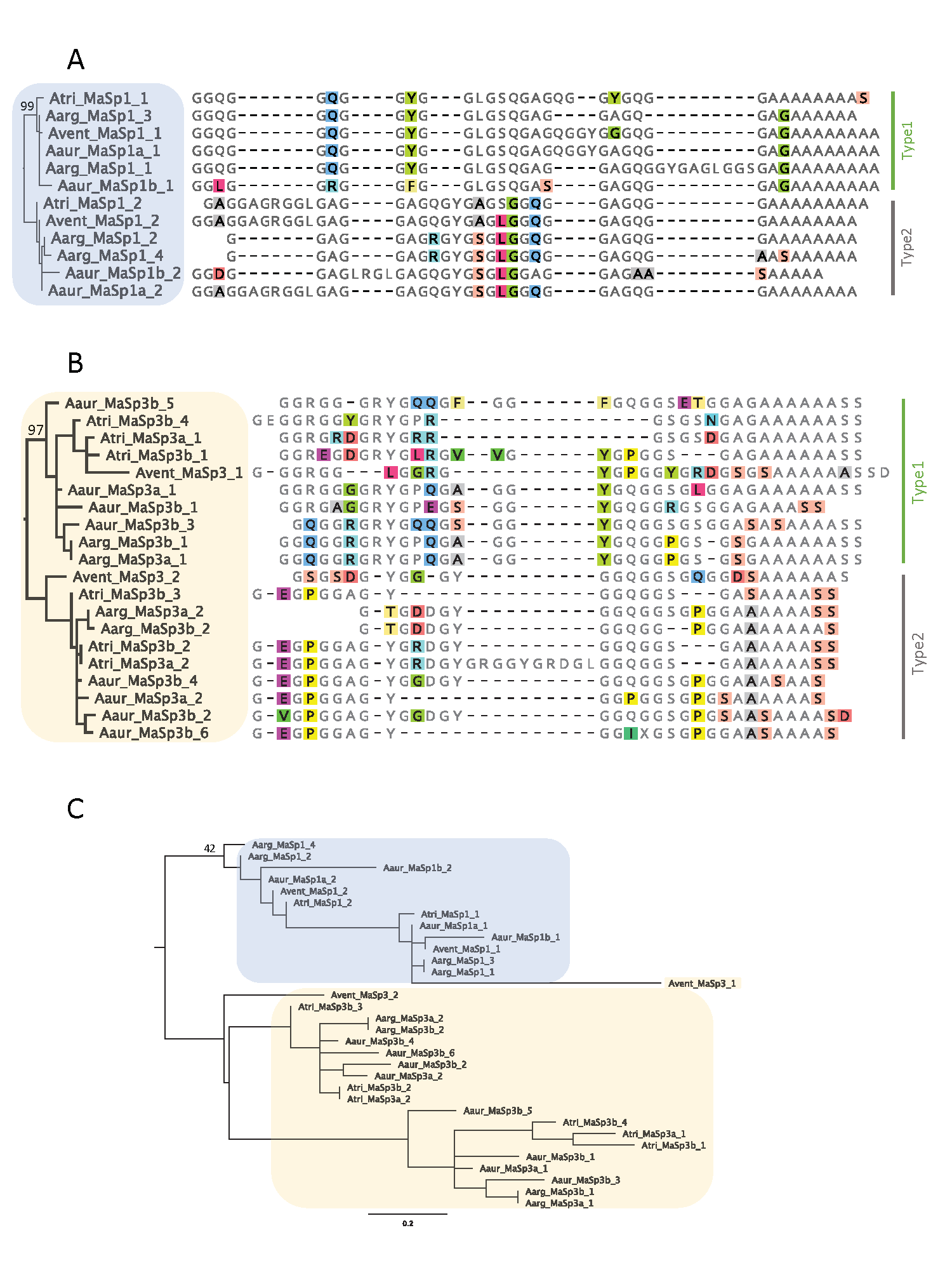

Supplement: S9 Fig — Alignment and phylogenetic relationships among consensus poly-A units of MaSp1 (A) and MaSp3 (B) for Argiope species and Araneus ventricosus. C) Phylogenetic analysis of combined protein matrix of MaSp1 and MaSp 3 consensus poly-A units. Given the close phylogenetic relationship between MaSp1 and MaSp3 genes (Fig 1) and the similarity in their ensemble repeat structures, we wanted to assess if there was shared homology between the poly-A types for each gene. However, the tree does not support this hypothesis as all the MaSp1 poly-A units group in one clade and all but one of the MaSp3 poly-A units group in a separate clade. Bootstrap value separating the two primary clades presented. (TIF) [file pgen.1010537.s009.tif]

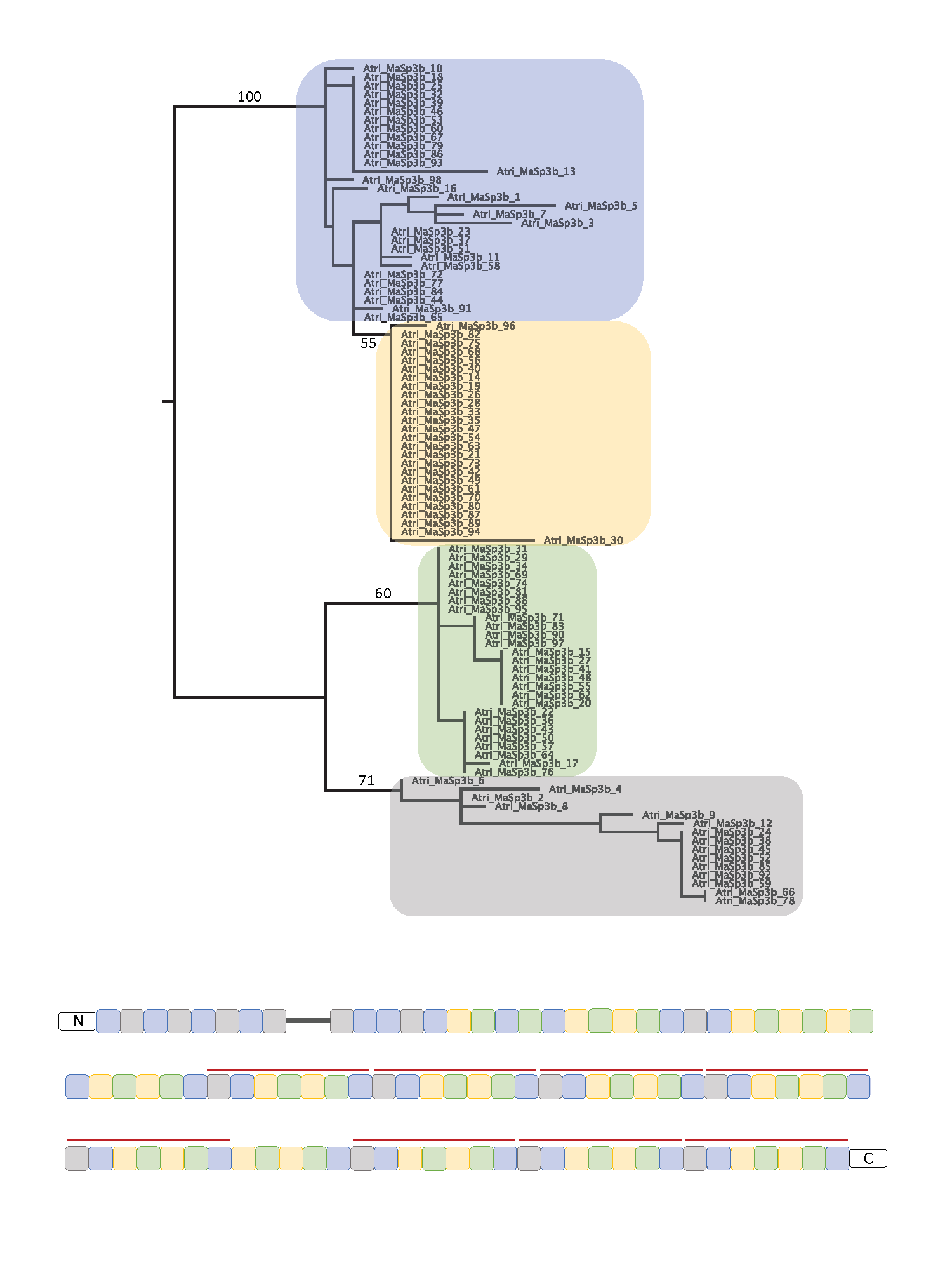

Supplement: S10 Fig — Phylogenetic relationships (A) and repeat arrangement (B) among poly-A units. The taxa-gene numbers indicate the order of the poly-A unit in the gene. Colored blocks in the gene schematic correspond to poly-A units that belong to the same-colored grouping on the phylogeny (bootstrap values provide for these 4 nodes). B) Ensemble repeats with stereotypical structure indicated by red lines above several seven-block sections. N and C blocks at each end of the gene indicate the N- and C-terminal regions, and the thick line after the first eight colored blocks indicates linker sequence (see Results for description). (TIF) [file pgen.1010537.s010.tif]

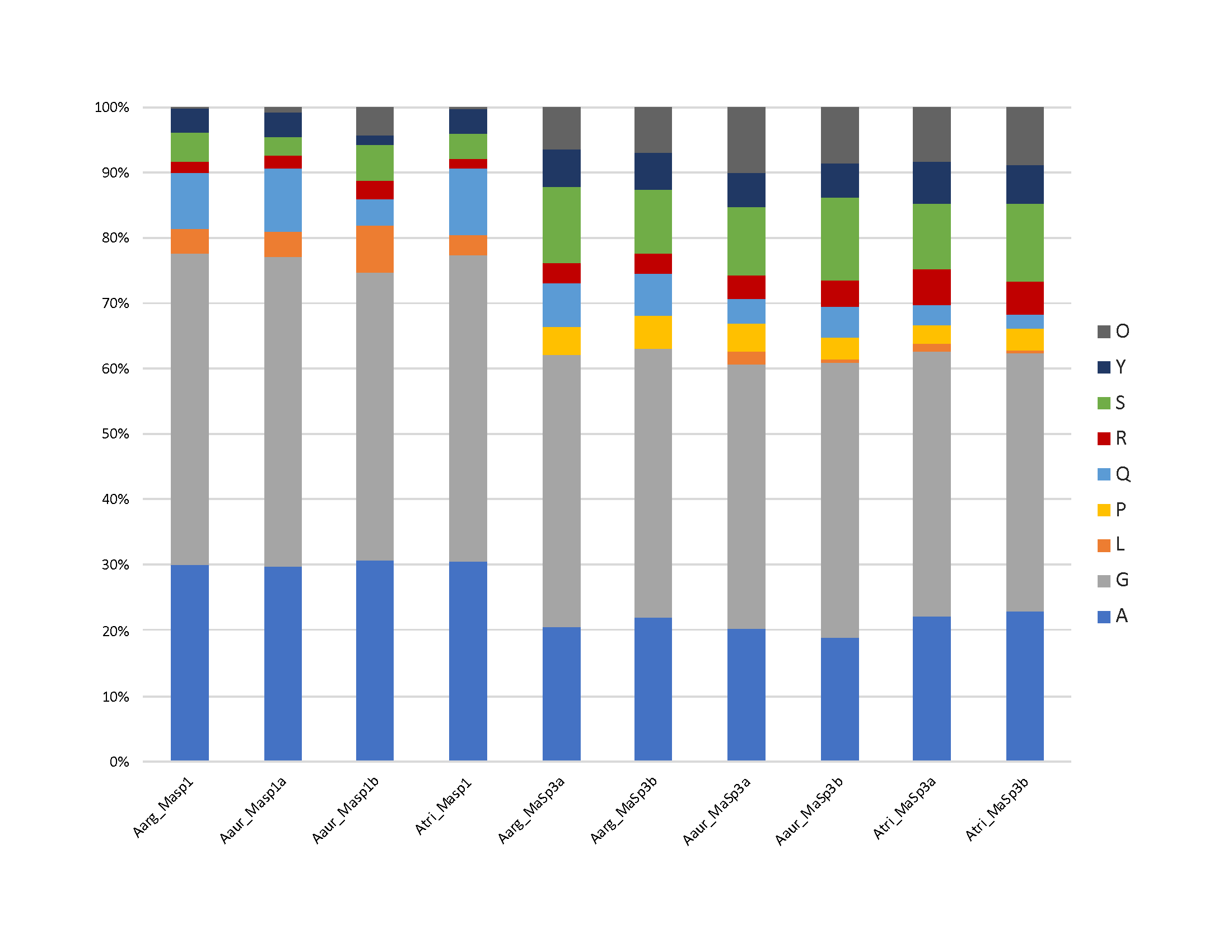

Supplement: S11 Fig — In the legend, ‘O’ indicates other amino acids not listed in legend. (TIF) [file pgen.1010537.s011.tif]

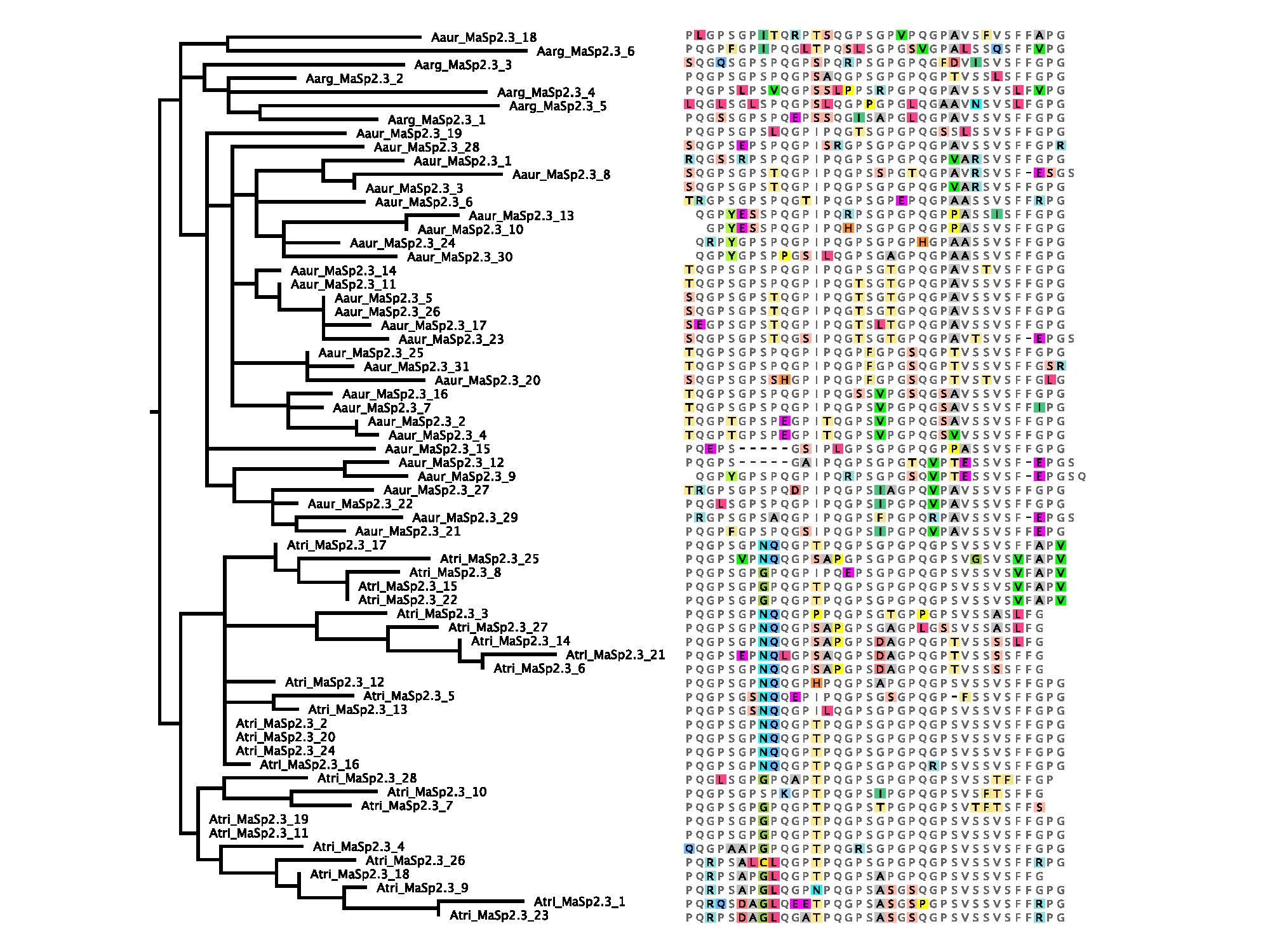

Supplement: S13 Fig — Colored amino acids indicate variable sites relative to the consensus sequence. (TIF) [file pgen.1010537.s013.tif]

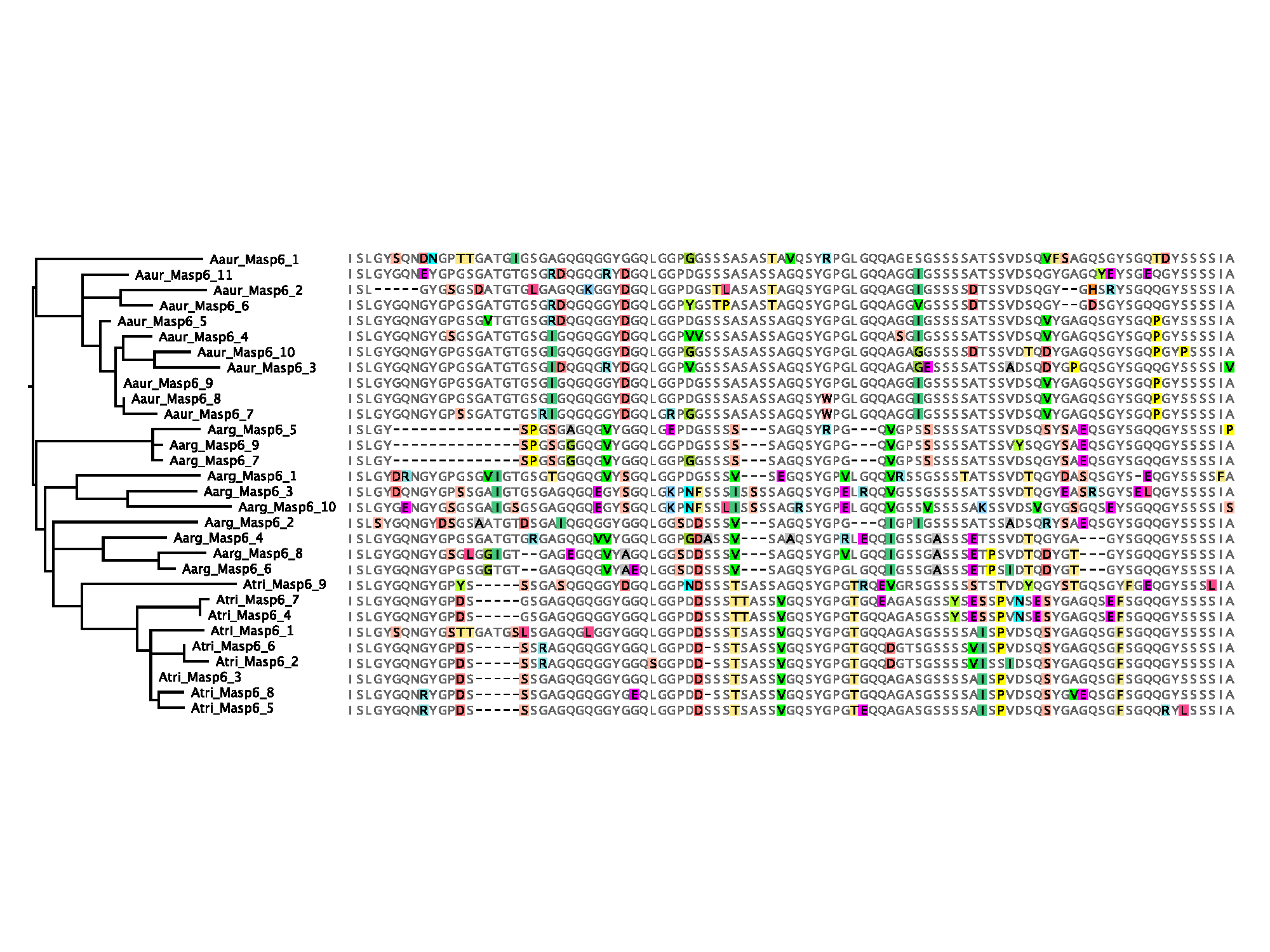

Supplement: S14 Fig — Colored amino acids indicate variable sites relative to the consensus sequence. (TIF) [file pgen.1010537.s014.tif]

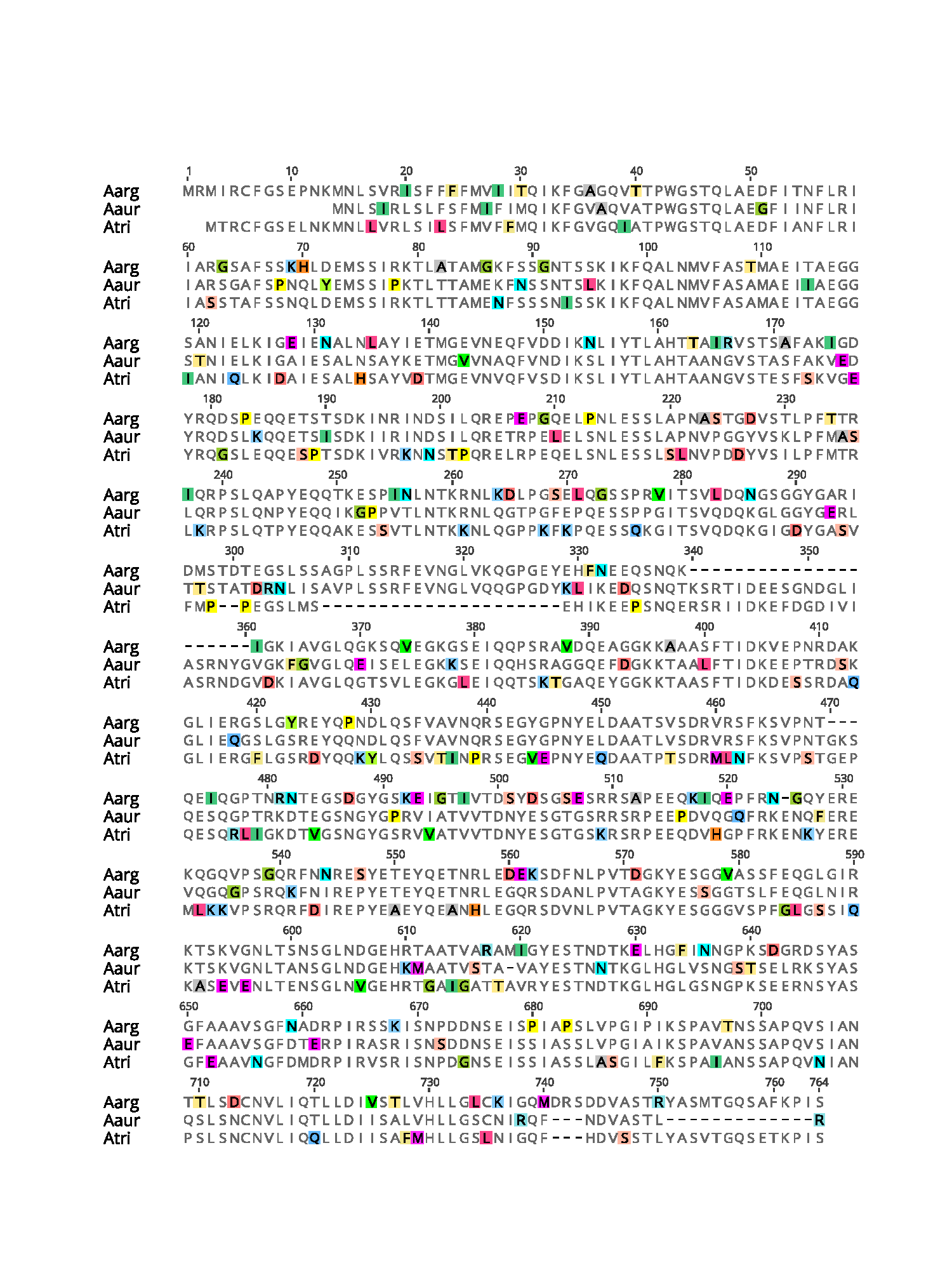

Supplement: S15 Fig — (TIF) [file pgen.1010537.s015.tif]

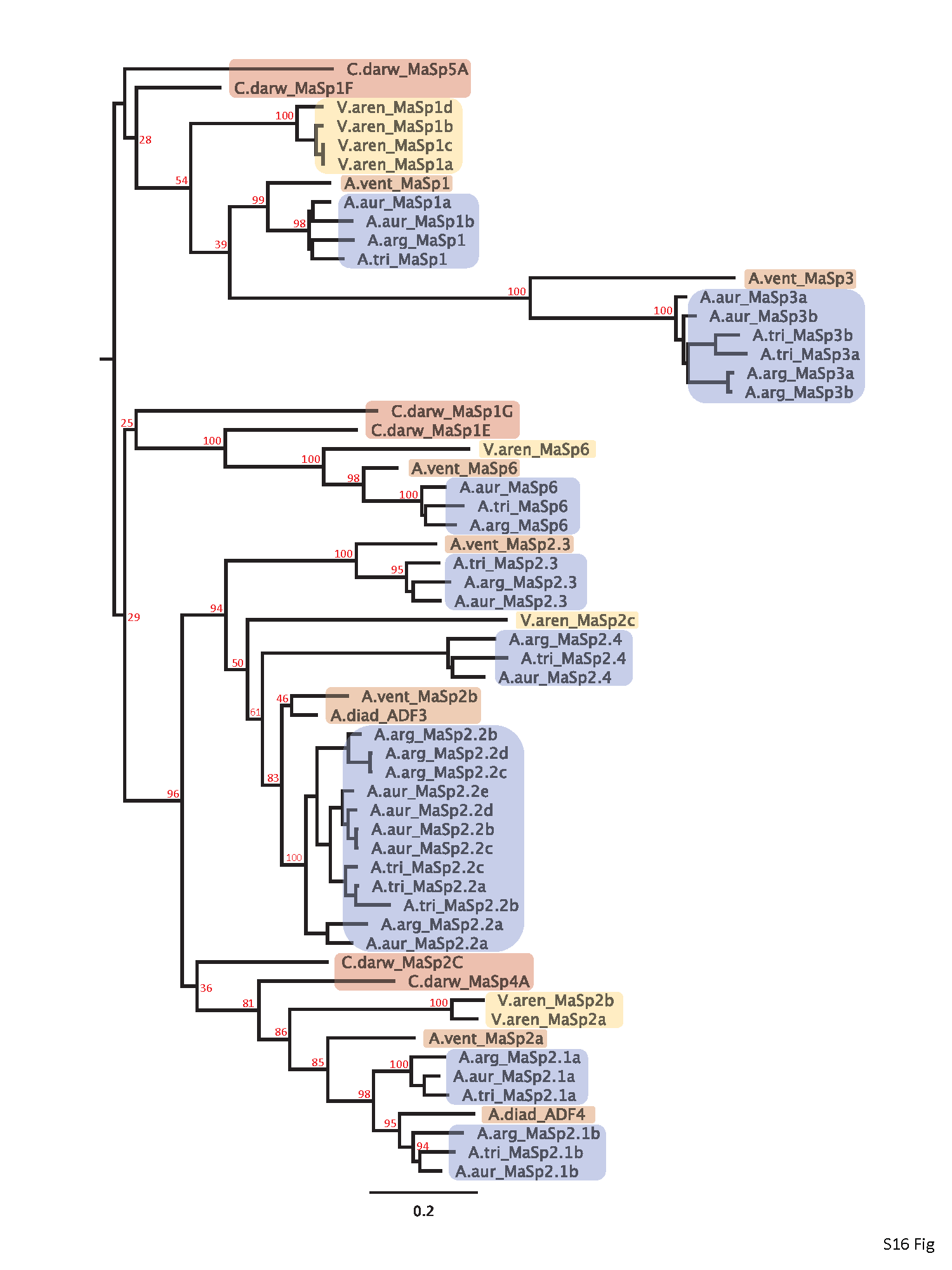

Supplement: S16 Fig — Derived from the concatenated nucleotide sequence of N- and C-terminal regions. Clade shading highlight different genera. Bootstrap values provided for nodes at generic level and above. A.diad–Araneus diadematus, A.vent–Araneus ventricosus, A.arg–Argiope argentata, A.aur–Argiope aurantia, A.tri–Argiope trifasciata, C.darw–Caerostris darwini, V.aren–Verrucosa arenata. (TIF) [file pgen.1010537.s016.tif]
