# Supplementary material for: Rapid molecular diversification and homogenization of clustered major ampullate silk genes in Argiope garden spiders
Source: PLoS Genet. 2022 Dec 12;18(12):e1010537. doi: 10.1371/journal.pgen.1010537 (PMC9779670; doi:10.1371/journal.pgen.1010537)
Supplement: S1 File — (DOCX) [file pgen.1010537.s024.docx]

>Aarg_Masp1

MIWTTRLALSILLVICSQSIFALGQSPWQSASMAESFMTSFSNALGQSQAFTDEQMDDIDTIAASIKMGVDKMERSGKTSQNKLQAMNMAFASAVAEIAISEGGGQSAQVKTNAIADALASAFLQTTGVVNGQFINEIRGLISMFAQANSISSSSASASASAGGAGGYGSQASGAAAATNGGYGQGSSQGSQAYGAGAPGAGPKIQSTQGQGQNSYQYSISVRSQSGSQGTIGGQQGGQGGYSSQGAGGADQGGYGGQGRAGAAAAAAAAGGAGQGYGSGLGSQGGTGQGGANAAAAAAAGGQGGQGGYGGLGSQGAGGAGQGGYGAGLGGSGAGAAAAAAAAGGAGGAGRGYGSGLGGQGGAGQGGAAAAAAAAGGQGGQGGYGGLGSQGAGGAGQGAGAAAAAAGGAGGAGRGYGSGLGGQGGAGQGGAAAAAAAAGGQGGQGGYGGLGSQGAGGAGQGGYGAGLGGSGAGAAAAAAAAGGAGGAGRGYGSGLGGQGGAGQGGAAAAAAAAGGQGGQGGYGGLGSQGAGGAGQGAGAAAAAAGGAGGAGRGYGSGLGGQGGAGQGAASAAAAGGQGGQGGYGGLGSQGAGGAGQGGYGAGLGGSGSGAAAAAAAAGGAGGAGRGYGSGLGGQGGAGQGGAAAAAAAAGGQGGQGGYGGLGSQGAGGAGQGAGAAAAAAGGAGGAGRGYGSGLGGQGGAGQGAASAAAAGGQGGQGGYGGLGSQGAGGAGQGGYGAGLGGSGSGAAAAAAAAGGAGGAGRGYGSGLGGQGGAGQGGAASAAAAAGGQGGQGGYGGLGSQGAGGAGQGAGAAAAAAAAGGAGGAGRGYGSGLGSQGGAGQGAASAAAAGGQGGQGGYGGLGSQGAGGAGQGGYGAGLGGSGSGSGAAAAAAAAGGAGGAGRGYGSGLGSQGGAGQGGAAAAAAAAAGGQGGQGGYGGLGSQGAGGAGQGGYGAGLGGSGAGAAAAAAAAGGAGGAGRGYGSGLGGQGGAGQGGAAGAAAAGGQGGQGGYGGLGSQGAGGAGQGAGAAAAAAGGAGGAGRGYGSGLGGQGGAGQGAASAAAAGGQGGQGGYGGLGSQGAGGAGQGGYGAGLGGSGSGSGAAAAAAAAGGAGGAGRGYGSGLGGQGGAGQGGAAAAAAAAGGQGGQGGYGGLGSQGAGGAGQGAGAAAATAGGAGGAGRGYGSGLGGQGGAGQGGAAAAAAAAGGQGGQGGYGGLGSQGAGGAGQGAGAAAAAAGGAGGAGRGYGSGLGGQGGAGQGAASAAAAGGQGGQGGYGGLGSQGAGGAGQGGYGAGLGGSGSGAAAAAAAAGGAGGAGRGYGSGLGGQGGAGQGGAAAAAAAAGGQGGQGGYGGLGSQGAGGAGQGAGAAAAAAGGAGGAGRGYGSGLGGQGGAGQGAASAAAAGGQGGQGGYGGLGSQGAGGAGQGGYGAGLGGSGSGAAAAAAAAGGAGGAGRGYGSGLGGQGGAGQGGAAAAAAAAGGQGGQGGYGGLGSQGAGGAGQGAGAAAAAAAAGGAGGAGRGYGSGLGGQGGAGQGAASAAAAGGQGGQGGYGGLGSQGAGGAGQGGYGAGLGGSGSGAAAAAAAAGGAGGAGRGYGSGLGGQGGAGQGGAAAAAAAAGGQGGQGGYGGLGSQGAGGAGQGAGAAAAAAAGGAGGAGRGYGSGLGGQGGAGQGAASAAAAGGQGGQGGYGGLGSQGAGGAGQGGYGAGLGGSGAGAAAAAAAAGGAGGAGRGYGSGLGGQGGAGQGGAAAAAAAAGGQGGQGGYGGLGSQGAGGAGQGAGAAAAAAGGAGGAGRGYGSGLGGQGGAGQGAASAAAAGGQGGQGGYGGLGSQGAGGAGQGGYGAGLGGSGAGAAAAAAAAGGAGGAGRGYGSGLGGQGGAGQGGAAAAAAAAGGQGGQGGYGGLGSQGAGGAGQGAGAAAAAAGGAGGAGRGYGAGLGGSGAGAAAAAAAAGGAGGAGRGYGSGLGGQGGAGQGAASAAAAGGQGGQGGYGGLGSQGAGGAGQGGYGAGLGGSGAGAAAAAAAAGGAGGAGRGYGSGLGGQGGAGQGGAAAAAAAAGGQGGQGGYGGLGSQGAGGAGQGAGAAAAAAGGAGGAGRGYGSGLGGQGGAGQGAASAAAAGGQGGQGGYGGLGSQGAGGAGQGGYGAGLGGSGAGAAAAAAAAGGAGGAGRGYGSGLGGQGGAGQGGAAAAAAAAGGQGGQGGYGGLGSQGAGGAGQGAGAAAAAAAGGAGGAGRGYGSGLGGQGGAGQGAASAAAAGGQGGQGGYGGLGSQGAGGAGQGGYGAGLGGSGAGAAAAAAAAGGAGGAGRGYGSGLGGQGGAGQGGAAAAAAAAGGQGGQGGYGGLGSQGAGGAGQGAGAAAAAAGGAGGAGRGYGSGLGGQGGAGQGAASAAAAGGQGGQGGYGGLGSQGAGGAGQGGYGAGLGGSGAGAAAAAAAAGGAGGAGRGYGSGLGGQGGAGQGGAAAAAAAAGGQGGQGGYGGLGSQGAGGAGQGAGAAAAAAGGAGGAGRGYGSGLGGQGGAGQGAASAAAAGGQGGQGGYGGLGSQGAGGAGQGGYGAGLGGSGAGAAAAAAAAGGAGGAGRGYGSGLGGQGGAGQGGAAAAAAAAGGQGGQGGYGGLGSQGAGGAGQGAGAAAAAAGGAGGAGRGYGSGLGGQGGAGQGAASAAAAGGQGGQGGYGGLGSQGAGGAGQGGYGAGLGGSGAGAAAAAAAAGGAGGAGRGYGSGLGGQGGAGQGGAAAAAAAAGGQGGQGGYGGLGSQGAGGAGQGAGAAAAAAGGAGGAGRGYGSGLGGQGGAGQGAASAAAAGGQGGQGGYGGLGSQGAGGAGQGGYGAGLGGSGAGAAAAAAAAGGAGGAGRGYGSGLGGQGGAGQGGAAAAAAAAGGQGGQGGYGGLGSQGAGGAGQGAGAAAAAAGGAGGAGRGYGSGLGGQGGAGQGAASAAAAGGQGGQGGYGGLGSQGAGGAGQGGYGAGLGGSGAGAAAAAAAAGGAGGAGRGYGSGLGGQGGAGQGGAAAAAAAAGGQGGQGGYGGLGSQGAGGAGQGAGAAAAAAGGAGGAGRGYGSGLGGQGGAGQGAASAAAAGGQGGQGGYGGLGSQGAGGAGQGGYGAGLGGSGAGAAAAAAAAGGAGGAGRGYGSGLGGQGGAGQGGAAAAAAAAGGQGGQGGYGGLGSQGAGGAGQGAGAAAAAAGGAGGAGRGYGSGLGGQGGAGQGAASAAAAGGQGGQGGYGGLGSQGAGGAGQGGYGAGLXGSGAGAAAAAAAAGGAGGAGRGYGSGLGGQGGAGQGGAAAAAAAAGGQGGQGGYGGLGSQGAGGAGQGAGAAAAAAGGAGGAGRGYGSGLGGQGGAGQGAASAAAAGGQGGQGGYGGLGSQGAGGAGQGGYGAGLXGSGAGAAAAAAAAGGAGGAGRGYGSGLGGQGGAGQGGAAAAAAAAGGQGGQGGYGGLGSQGAGGAGQGAGAAAAAAGGAGGAGRGYGSGLGGQGGAGQGAAAAAAAGGQGGQGGYGGLGSQGAGGAGQGGYGAGLGGSGAGAAAAAAAAGGAGGAGRGYGSGLGGQGGAGQGGAAAAAAAAGGQGGQGGYGGLGSQGAGGAGQGAGAAAAAAAAGGAGGAGRGYGSGLGDXGGAGQGGGAAAAAGGQGGQGGYGGLGSQGAGQGGYGSGSYSGQQSGAASVAAASAAASRLSSPGAASRVSSAVTSLVSSGGPTNGAALSNTISNVVSQISASNPGLSGCDVLVQALLEIVSALVHILGSANIGQVNSNSAGRSASLVGQSVYQALS

>Aaur_Masp1a

MNWTTRLALSVLVVICSQSIFALGQSPWQSASMAESFMTYFSAALGQSGAFTNEQMDDIDTIATSIKMGVDKMERSGKTSQNKLQAMNMAFASAVAEIAIAEGGGQSAQVKTNAIADALASAFLQTTGVVNRQFINEIRGLISMFAQANSISSSSGYASASAEAAAGSAGGAGQGYGAGLGGQGGAGQGGAAAAAAAAGGQGGQGGYGGLGSQGAGQGGYGAGQGGAGAAAAAAAAGGAGGAGRGGLGAGGGGQGYGSGLGGQGGAGQGGAAAAAAAAGGQGGQGGYGGLGSQGAGQGGYGAGQGGAGAAAAAAAAGGAGGAGRGGLGAGGAGQGYGSGLGGQGGAGQGGAAAAAAAAGGQGGQGGYGGLGSQGAGQGGAGAAAAAAAAGGAGGAGRGGLGAGGAGQGYGSGLGGQGGAGGGAAAAAAAAGGQGGQGGYGGLGSQGASQGGAGRGAAAAAAAAGGQGGQGGYGGLGSQGAGQGGYGAGQGGAGAAAAAAAAGGTGGAGRGGLGAGGAGQGYGSGLGGQGGAGQGGAAAAAAAAGGQGGLGGYGGLGSQGAGQGGYGAGQGGAGAAAAAAAAGGAGGAGRGGLGAGGAGQGYGSGLGGQGGAGQGGAAAAAAAAGSQGGQGGYGGLGSQGAGQGGYGAGQGGAGAAAAAAAAGGAGRGGLGAGGAGQGYGSGLGGQGGAGGGAAAAAAAAGGQGGQGGYGGLGSQSAGQVGAGRGAAAAAAAAGGQGGQGGYGGLGSQGAGQGGYGAGQGGAGAAAAAAAAGGAGGAGRGGLGAGGAGQGYGSGLGGQGGAGQGGAAAAAAAAGGQGGQGGYGGLGSQGAGQGGYGAGQRGAGAAAAAAAAGGAGGAGRGGLGAGGAGQGYGSGLGGQGGAGQGGAAAAAAAAGDQGGQGGYGGLGSQGAGPGGYGAGQGGAGAAAAAAAAGGAGRGGLGAGGAGQGYGSGLGGQGGAGQGGAAAAAAASGGQGGQGGYGGLGSQGAGQGGYGAGAAAAAAAAGGAGRGGLGAGGAGQGYGSGLGGQGGARGGAAAAAAAASGQGGQGGYGGLGSQGAGQGGAGRGASAAAAAAGGQGGQGGYGGLGSQGAGQGGYGAGQGGAGAAAAAAADGGSGGAGRGGLGAGGAGRYGSGLGGQGGDGQGGAAAAAAAAAGGQSGQGGYGGLGSQGAGQGGYGAGQGGAGAAAAAAAAGGAGGAGRGGLGAGGAGQGYGSRLGGQGRAGQGGAAAAAAAAGGQGGQGGYGGLGSQGAGQGGYGAGQGGAGAAAAAAAAGAAGGAGRGGLGAGGAGQGYGSGLGGQGGAGQGGAAAAAAAAGGQGGQGGYGGLGSQGAGQGGYGAGQGGAGAAAAAAAAGGAGGAGRGGLGAGGAGRYGSGLGGQGGDGQGGAAAAAAAAAGGQGGYGGLGSQGAGQGGAGRGAAAAAAAAGGQGGQGGYGGLGSQGAGQGGYGAGQGGAGAAAAAAAAGGAGGAGRGGLGAGGAGQGYGSGLGGQGGAGGGAAAAAAAAGGQGGQGGYGGLGSQGAGQGGAGRGASAAAAAAGGQGGQGGYGRLGSQGAGQGGYGAGQGGAGAAAAAAGGAGGAGRGGLGAGGAGRYGSGLGGQGGDGQGGAAAAAAAAAGGQGGYGGLGSQGVGQGGAGRGAAAAAAAAGGQGGQGGYGGLGSQGAGQGGYGAGQGGAGAAAAAAAAGGAGGAGRGGLGAGGAGRYGSGLGGQGGAGQGGAAAAAAAAGGQGGQGGYGGLGSQGAGQGGYGAGAAAAAAAAGGAGGAGRGGLGAGGAGQGYGSGLGGQGGAGQGGAAAAAAAAGGQGGQGGYGGLGSQGAGQGGYGAGQGGAGAAAAAAAAGGAGGAGRGGLGAGGAGRYGSGLGGQGGDGQGGAAAAAAAAAGGQGGQGGYGGLGSQGAGQGGAGRGAAAAAAAAGGQGGQGGYGGLGSQGAGQGGYGAGQGGAGAAAAAAAAGGAGGAGRGGIGAGGAGRYGSGLGGQGGAGQGGAAAAAAAAGGQGGQGGYGGLGSQGAGQGGYGAGAAAAAAAAGGAGGAGRGGLGAGGAGQGYGSGLGGQGGAGQGGAAAAAAAAGGQGGQGGYGGLGSQGAGQGGYGAGQGGAGAAAAAAAAGGAGGAGRGGLGAGGAGQGYGSGLGGQGGAGQGGAAAAAAAAAGGQGGQGGYGGLGSQGAGQGGYGAGQGGAGAAAAAGGAGGAGRGGLGAGGAGQGYGSGLGGQGGAGQGGAAAAAAAAGGQGGQGGYGGLGSEGAGQGGYGAGQGGAGAAAAAAAAGGAGGAGRGGLGAGGAGQGYGSGLGGQGGAGQGGAAAAAAAAGGQGGQGGYGGLGSQGAGQGGAGRGAAAAAAAAGGQGGQGGYGGLGSQGAGQGGYGAGQGGAGAAAAAAAAGGAGGAGRGGLGAGGAGQGYGSGLGGQGGAGQGGAAAAAAAAGGQGGQGGYGGLGSQGAGQGGYGAGQGGAGAAAAAATAGGAGGAGRGGLGAGGAGQGYGSGLGGQGGAGQGGAAAAAAAAGGQGGQGGYGGLGSQGAGQGGYGAGAAAAAAAAGGAGGAGRGGLGAGGAGQGYGSGLGGQGGAGQGGAAAAAAAAGGQGGQGGYGGLGSQGAGQGGYGAGQGGAGAAAAAAAAGGAGGAGRGGLGAGGAGRYGSGLGGQGGDGQGGAAAAAAAAAGGQGGQGGYGGLGSQGVGRDAAAAAAAAGGQGGQGGYGGLGSQGAGQGGYGAGQGGAGAAAAAAAAGGAGGAGRGGLGAGGAGQGYGSGLGGQGGAGQGGAAAAAAAAAGGQGGQGGYGGLGSQGAGQGGYGVGQRGAGAAAAAAAAGGAGGAGRGGLGAGGAGQVYGSGLGGQGGAGQGGAATAAAAAGGQGGQGGYGGLGSEGAGQGGYGAGQGGAGAAAAAAAAGGAGGAGRGGLGAGGAGQGYGSGLGGQGGAGQGGAAAAAAAAGGQGGQGGYGGLGSQGAGQGGAGRGAAAAAAAAGGQGGQGGYGGLGSQGAVQGGAGRGAAAAAAAAGGQGGQGGYGGLGSQGAGQGGAGRGAAAAAAAAGGQGGQGGYGGLGSEGAGQGGAGRGAAAAAAAAGGQGGQGGYGGLGSQGAGQGGYGAGQGGARAAAAAAAAGGAGGAGRGGLGAGGAGQGYGSGLGGQGGAGQGGAAAAAAAAAAGGQGGQGGYGGLGSQGAGQGGYGAGQGGAGAAAAAATAGGAGGAGRGGLGAGGAGQGYGSGLGGQGGAGQGGAAAAAAAAGGQGGQGGFGRFSSQEAGQGAYGGGAYSGQQGAAASVSAASAAASRLSSPGAASRVSSAVTSLVSSGGPTNPAALSNTISNVVSQISESNPGLSGCDVLVQALLELVSALVHILGSANIGQVNSSAAGQSASLVRQSVYQALS

>Aaur_Masp1b

MIWTVRFSLSLLIVICSQSIFALGQSPWQSASMAESFMTYFSAALGQSGAFTNEQMDDIDTIASSIKMGVDKMERSGKTSQNKLQAMNMAFASAVADIAIAEGGGQSAQIKTNAIADALASAFLQTTGTVNNQFINEIRGLISMFAQANSISSSSASASESTAAAAGGPGGAGQGYGSGLGGAGGVGAASAAAAAGGLGGRGGFGGLGSQGVGGAGQGAGAAAAAAAAGGDGGAGLRGLGAGQGYGSGLGGAGGAGAASAAAAAGGLGGRGGFGGLGSQGASGAGQGGAGAAAAAAAAGGDGGAGLRGLGAGQGYGSGLGGAGGAGAASAAAAAGGLGGRGGFGGLGSQGVGGAGQGAGAAAAAAAAGGDGGAGLRGLGAGQGYGSGLXGAGAGAASAAAAAGGLGGRGGFGGLGSQGVGGAGQGGAGAAAAAAAAGGDGGAGLRGLGAGQGYGSGLGGAGGAGAASAAAAAGGVGGRGGFGGLGSQGASGAGQGGAGAASAAAAAGGLGGRGGFGGLGSQGASGAGQGGAGAAAAAAAAGGDGGAGLRGLGAGQGYGSGLGGAGGAGAASAAAAAGGLGGRGGFGGLGSQGASGAGQGAGAAAAAAAAGGDGGAGLRGLGAGQGYGSGLGGAGGAGAASAAAAAGGLGGRGGFGGLGSQGVGGAGQGGAGAAAAAAAAGGDGGAGLRGLGAGQGYGSGLGGAGGAGAASAAAAAGGLGGRGGFGGLGSQGASGAGQGGAGAAAAAAAAGGDGGAGLRGLGAGQGYGSGLGGAGGAGAASAAAAAAGGDGGAGLRGLGAGQGYGSGLGGAGGAGAASAAAAAGGLGGRGGFGGLGSQGVGGAGQGAGAAAAAAAAGGDGGAGLRGLGAGQGYGSGLGGAGGAGAASAAAAAGGLGGRGGFGGLGSQGASGAGQGGAGAAAAAAAAGGDGGAGLRGLGAGQGYGSGLGGAGGAGAASAAAAAGGLGGRGGFGGLGSQGASGAGQGGAGAAAAAAAAGGDGGAGLRGLGAGQGYGSGLGGAGGAGAASAAAAAGGLGGRGGFGGLGSQGASGAGQGGAGAAAAAAAAGGDGGAGLRGLGAGQGYGSGLGGAGGAGAASAAAAAGGLGGRGGFGGLGSQGASGAGQGGAGAAAAAAAAGGDGGAGLRGLGAGQGYGSGLGGAGGAGAASAAAAAGGLGGRGGFGGLGSQGVGGAGQGAGAAAAAAAAGGDGGAGLRGLGAGQGYGSGLGGAGGAGAASAAAAAGGLGGRGGFGGLGSQGVGGAGQGAGAAAAAAAAGGDGGAGLRGLGAGQGYGSGLGGAGGAGAASAAAAAGGLGGRGGFGGLGSQGASGAGQGGAGAAAAAAAAGGDGGAGLRGLGAGQGYGSGLGGAGGAGAASAAAAAGGLGGRGGFGGLGSQGASGAGQGGAGAAAAAAAAGGDGGAGLRGLGAGQGYGSGLGGAGGAGAASAAAAAGGLGGRGGFGGLGSQGASGAGQGAGAAAAAAAAGGDGGAGLRGLGAGQGYGSGLGGAGGAGAASAAAAAGGLGGRGGFGGLGSQGASGAGQGGAGAAAAAAAAGGDGGAGLRGLGAGQGYGSGLGGAGAASAAAAAGGLGGRGGFGGLGSQGASGAGQGGAGAAAAAAAAGGDGGAGLRGLGAGQGYGSGLGGAGGAGAASAAAAAGGLGGRGGFGGLGSQGASGAGQGGAGAAAAAAAAGGDGGAGLRGLGAGQGYGSGLGGAGGAGAASAAAAAGGLGGRGGFGGLGSQGVGGAGQGAGAAAAAAAAGGDGGAGLRGLGAGQGYGSGLGGAGGAGAASAAAAAGGLGGRGGFGGLGSQGASGAGQGGAGAASAAAAAGGVGGRGGFGGLGSEGASGAGQGGAGAAAAAAAAGGDGGAGLRGLGAGQGYGSGLGGAGGAGAASAAAAAGGLGGRGGFGGLGSQGVGGAGQGGAGAAAAAAAAGGDGGAGLRGLGAGQGYGSGLGGAGGAGAASAAAAAAGGDGGAGLRGLGAGQGYGSGLGGAGGAGAASAAAAAGGLGGRGGFGGLGSQGVGGAGQGAGAAAAAAAAGGDGGAGLRGLGAGQGYGSGLGGAGAASAAAAAGGLGGRGGFGGLGSQGASGAGQGGAGAAAAAAAAGGDGGAGLRGLGAGQGYGSGLGGAGGAGAAAAAAAAGGDGGAGLRGLGAGQGYGSGLGGAGGAGAASAAAAAGGLGGRGGFGGLGSQGASGAGQGAGAAAAAAAAGGDGGAGLRGLGAGQGYGSGLGGAGGAGAASAAAAAGGLGGRGGFGGLGSQGASGAGQGGAGAAAAAAAAGGDGGAGLRGLGAGQGYGSGLGGAGGAGAASAAAAAGGLGGRGGFGGLGSQGASGAGQGGAGAAAAAAAAGGDGGAGLRGLGAGQGYGSGLGGAGGAGAASAAAAAGGLGGRGGFGGLGSQGASGAGQGGAGAAAAAAAAGGDGGAGLRGLGAGQGYGSGLGGAGGAGAASAAAAAGGLGGRGGFGGLGSQGVGGAGQGAGAAAAAAAAGGDGGAGLRGLGAGQGYGSGLGGAGAASAAAAAGGLGGRGGFGGLGSQGVGGAGQGGAGAAAAAAAAGGDGGAGLRGLGAGQGYGSGLGGAGGAGAAGAAAAAGGLGGRGGFGGLGSQGVGSAGQGAGAAAAAAAAGGDGGTGLRGLGAGKGYGAGLGGAGGAGAASAAAAAAGGDGGAGLRGLGAGQGYGSGLGGAGGAGAASAAAAAGGLGGRGGFGGLGSQGVGGAGQGAGAAAAAAAAGGDGSAGLRGLDAIQGYGSGLGGAGAASAAAAAGGLGGRGGFGGLGSQGASGAGQGGAGAAAAAAAAGGDGGAGLRGLGAGQGYGSGLGGAGGAGAASAAAAAAGGDGGTGLRGLGAGQGYGSGLGGAGGAGAASVAAAAGGVGGRGGFGGLGSQGASGAGQGGAGAAAAAAAAGGDGGAGLRGLGAGKGYGSGLGGAGGAGAASAAAAAAGGDGSAGLRGLDAIQGYGSGLGGAGAASAAAAAGGLGGRGGFGGLGSQGASGAGQGGAAAAAAAGGDSGAGLRGLGAGQGYGSGLGGAGGAGAASAAAAAGGLGGRGGFGGLGSQGVGGAGKGAGAAAAAAAAGGDGGAGLRGLGAGQGYGSGLGXAGGAGAASAAAAAGGLGGRGGFGGLGSQGVGGAGQGAGAAAAAAAAGGDGGAGLRGLGAGQGYGSGLGGAGGAGAASAAAAAGGLGGRGGFGGLGSQGVGGAGQGAGAAAAAAAAGGDGGAGLRGLGAGQGYGSGLGGAGGAGAASAAAAAAGGDGSAGLRGLGAGQGYGSGLGGAGGAGAASAAAAAGGLGGRGGFGGLGSQGVGSAGQGAGAAAAAAAAGGDGGAGLRGLGAGQGYGSGLLGAGAASAAAAAGGLGGRGGFGGLGSQGASGAGQGGAGAAAAAAAAGGDGGAGLRGLGAGQGYGSGLGGAGGAGAASAAAAAGGLEGRGGFGGLGSQGVGGAVQGGAGASASAAASGGYGGLGSELEVQGAYRSGAYSGQQSAAVSVAAVSAAASRLSSPNAASRISSAVTSLISGGGPTNLAALSNTFSNVVYQISVSNPGLSGCDVLVQALLELVSALVHILGSAIIGHVNSSAAGETAALVGQSVYQAFS

>Atri_Masp1

MIWTTRLALSILVVICSQSIFAQGQSPWQSASMAESFMTYFSAALGQSGAFTNEQMDDIDTIATSIKMGVDKMERSGKTSLNKLQAMNMAFASAVAEIAISEGGGQSAQVKTNAIADALASAFLQTTGVVNRQFINEIRGLISMFAQANSISSSSASASASAAGAGGYGSQGSGAAVSAGGGYGQGTSQGSQAYGAGASGAGPQTQSTQGQGQSSYQYSISVSSQGGYGGLGSQGAGQGGYGGGQGGAAAAAVAASGASGAGQGGLGAGGAGQGYGAGLGGQGGAGRGGAAAAAAAAAGGQGGQGGYGGLGSQGAGQGGYGQGGAAAAAAAASGAGGAGRGGLGAGGAGQGYGAGLGGQGGAGQGGAAAAAAAAAGGQGGQGGYGGLGSQGAGQGGYGQGGAAAAAAAASGAGGAGRGGLGAGGAGQGYGAGSGGQGGAGQGGAAAAAAAAAGGXGGLGGYGGLGSQGAGQGGYGQGGAAAAAAAASGAGGAGRGGLGAGGAGQGYGAGSGGQGGAGQGGAAAAAAAAAGGQGGQGGYGGLGSQGAGQGGYGQGGAAAAAAAASGSGGAGRGGLGAGGAGQGYGAGSGGQGGAGQGGAAAAAAAGGQGGQGGYGGLGSQGAGQGGYGQGGAAAAAAAASGSGGAGRGGLGAGGAGQGYGAGLGGQGGAGQGGAAAAAAAAAGGQGGQGGYGGLGSQGAGQGGYGQGGAAAASAAASGAGGAGRGGLGAGGAGQGYGAGSGGQGGAGQGGAAAAAAAGGQGGQGGYGGLGSQGAGQGGYGQGGAAAAAAAASGAGGAGRGGLGAGGAGQGYGAGLGGQGGAGQGGAAAAAAAAAGGQGGQGGYGGLGSQGAGQGGYGQGGAAAAAAAASGAGGAGRGGLGAGGAGQGYGAGSGGQGGAGQGGAAAAAAAGGQGGQGGYGGLGSQGAGQGGYGQGGAAAAAAAASGAGGAGRGGLGAGGAGQGYGAGSGGQGGAGQGGAAAAAAAAAGGQGGQGGYGGLGSQGAGQGGYGQGGAAAAAAAASGAGGAGRGGLGAGGAGQGYGAGSGGQGGAGQGGAAAAAAAGGQGGQGGYGGLGSQGAGQGGYGQGGAAAAAAAASGAGGAGRGGLGAGGAGQGYGAGSGGQGGAGQGGAAAAAAAAAGGQGGQGGYGGLGSQGAGQGGYGQGGAAAAAAAASGAGGAGRGGLGAGGAGQGYGAGLGGQGGAGQGGAAAAAAAAAGGQGGQGGYGGLGSQGAGQGGYGQGGAAAAAAAASGAGGAGRGGLGAGGAGQGYGAGSGGQGGAGQGGAAAAAAAAGGQGGQGGYGGLGSQGAGQGGYGQGGAAAAAAAASGAGGAGRGGLGAGGAGQGYGAGLGGQGGAGQGGAAAAAAAAAGGQGGQGGYGGLGSQGAGQGGYGQGGAAAAAAAASGAGGAGRGGLGAGGAGQGYGAGLGGQGGAGQGGAAAAAAAAAGGQGGQGGYGGLGSQGAGQGGYGQGGAAAAAAAASGAGGAGRGGLGAGGAGQGYGAGSGGQGGAGQGGAAAAAAAAAGGQGGQGGYGGLGSQGAGQGGYGQGGAAAAAAAASGAGGAGRGGLGAGGAGQGYGAGSGGQGGAGQGGAAAAAAAAAGGQGGQGGYGGLGSQGAGQGGYGQGGAAAAAAAASGAGGAGRGGLGAGGAGQGYGAGSGGQGGAGQGGAAAAAAAGGQGGQGGYGGLGSQGAGQGGYGQGGAAAAAAAASGAGGAGRGGLGAGGAGQGYGAGLGGQGGAGQGGAAAAAAAGGQGGQGGYGGLGSQGAGQGGYGQGGAAAAAAAASGAGGAGRGGLGAGGAGQGYGAGSGGQGGAGQGGAAAAAAAAGGQGGQGGYGGLGSQGAGQGGYGQGGAAAAAAAASGAGGAGRGGLGAGGAGQGYGAGSGGQGGAGQGGAAAAAAAAAGGQGGQGGYGGLGSQGAGQGGYGQGGAAAAAAAASGAGGAGRGGLGAGGAGQGYGAGSGGQGGAGQGGAAAAAAAAAGGQGGQGGYGGLGSQGAGQGGYGQGGAAAAAAAASGAGGAGRGGLGAGGAGQGYGAGSGGQGGAGQGGAAAAAAAAAGGQGGQGGYGGLGSQGAGQGGYGQGGAAAAAAAASGAGGAGRGGLGAGGAGQGYGAGSGGQGGAGQGGAAAAAAAAAGGQGGQGGYGGLGSQGAGQGGYGQGGAAAAAAAASGAGGAGRGGLGAGGAGQGYGAGSGGQGGAGQGGAAAAAAAAGGQGGQGGYGGLGSQGAGQGGYGQGGAAAAAAAASGAGGAGRGGLGAGGAGQGYGAGSGGQGGAGQGGAAAAAAAAAGGQGGQGGYGGLGSQGAGQGGYGQGGAAAAAAAASGAGGAGRGGLGAGGAGQGYGAGSGGQGGAGQGGAAAAAAAAAGGQGGQGGYGGLGSQGAGQGGYGQGGAAAAAAAASGAGGAGRGGLGAGGAGQGYGAGSGGQGGAGQGGAAAAAAAAAGGQGGQGGYGGLGSQGAGQGGYGQGGAAAAAAAASGAGGAGRGGLGAGGAGQGYGAGSGGQGGAGQGGAAAAAAAAAGGQGGQGGYGGLGSQGAGQGGYGQGGAAAAAAAASGAGGAGRGGLGAGGAGQGYGAGSGGQGGAGQGGAAAAAAAAAGGQGGQGGYGGLGSQGAGQGGYGQGGAAAAAAAASGAGGAGRGGLGAGGAGQGYGAGSGGQGGAGQGGAAAAAAAAAGGQGGQGGYGGLGSQGAGQGGYGQGGAAAAAAAASGAGGAGRGGLGAGGAGQGYGAGSGGQGGAGQGGAAAAAAAGGQGGQGGYGGLGSQGAGQGGYGQGGAAAAAAAASGAGGAGRGGLGAGGAGQGYGAGLGGQGGAGQGGAAAAAAAAAGGQGGQGGYGGLGSQGAGQGGYGQGGAAAAAAAASGAGGAGRGGLGAGGAGRGYGAGSGGQGGAGQGGAAAAAAAAAGGQGGQGGYGGLGSQGAGQGGYGQGGAAAAAAAASGAGGAGRGGLGAGGAGQGYGAGSGGQGGAGQGGAAAAAAAAAGGQGGQGGYGGLGSQGAGQGGYGQGGAAAAAAAASGAGGAGRGGLGAGGAGQGYGAGSGGQGGAGQGGAAAAAAAAAGGQGGQGGYGGLGSQGGYGQGGAAAAAAAASGAGGAGRGGLGAGGAGQGYGAGSGGQGGAGQGGAAAAAAAAAGGQGGQGGYGGLGSQGAGQGGYGQGGAAAAAAAASGAGGAGRGGLGAGGAGQGYGAGLGGQGGAGQGSAAAAAAAAAGGQGGQGGYGGLGSQGAGQGGYGQGGAAAAAAAASGAGGAGRGGLGAGGAGQGYGAGLGGQGGAGQGGAAAAAAAAAGGQGGQGGYGGLGSQGAGQGGYGQGGAAAAAAAASGAGGAGRGGLGAGGAGQGYGAGSGGQGGAGQGGAAAAAAAAAGGKGGQGGYGGLGSQGAGQGGYGQGGAAAAAAAASGAGGAGRGGLGAGGAGQGYGAGSGGQGGAGQGGAAAAAAAAAGGQGGQGGYGGLGSQGAGQGGYGQGGAAAAAAAASGAGGAGRGGLGAGGAGQGYGAGSGGQGGAGQGGAAAAAAAAAGGQGGQGGYGGLGSQGAGQGGYGQGGAAAAAAAASGAGGAGRGGLGAGGAGQGYGAGLGGQGGAGQGGAAAAAAAAAGGQGGQGGYGGLGSQGAGQGGYGQGGAAAAAAAASGAGGARRGGLGAGGAGQGYGAGLGGQGGAGQGGAAAAAAAAAGGQAGQGGYGGLGSQGAGQGGYGQGGAAAAAAAASGAGGARRGGLGAGGAGQGYGAGSGGQGGAGQGGAAAASAAAAGSQGGPGRYGGLGSQGSGQGGYGQGGAAAAAAAASGAGGAGRGGLGAGGAGQGYGAGSGGQGGAGQGGAAAAAAAAAGGQGGQGGYGGLGSQGAGQGGYGQGGAAAAAAAASGAGGAGRGGLGAGGAGQGYGAGLGGQGGAGQGGAAAAAAAGGKGGQGGYGGLGSQGAGQGGYGQGGAAAAAAAASGAGGAGQGGLGAGGAGQGYGAGSGGQGGAGQGGAAAAAAAAAGGQGGQGGYGGLGSQGAGQGGYGQGAAAAAAAASGAGGAGRGGLGAGGAGQGYGARLGSQGGAGQGGAAAAAAAGGQGGLGGYGGLGSQGSGQGGYGQGGAAATAAAASGAGGAGQGGLGAAGAGQGYGAGSGGQGGAGQGGAAAAAAAAAGGQGGQGGYGGLGSQGAGQGGYGQGGVAAAAAAASGAGGAGRGGLGAGGAGQVYGAVSGGQGGAGQGGEAAAAAAAAGGQGGQGGYGGLGSQGAGQGGYGQGGAAAAIAAASGAGGAGQGGLGAGGAGQGYGAGSGGQGGAGQGGAAAAAAAASGQGGQGGYGGLGSQGAGQGGYGQGGAAAAAASAGGQGGQGGYGGLGSQGAGQGGYGGGAFSGQQGGAASVATASAAASRLSSPGAASRVSSAVTSLVSSGGPTNSAALSNTISNVVSQISSSNPGLSGCDVLVQALLEIVSALVHILGSANIGQVNSSGVGRSASIVGQSINQAFS

>Aarg_MaSp2.1a

MSCPRLVLAFLALLSTHALFASAAGATPWSSPAMADSFMTNFINGIANSRAFTGGQIDDMTTIGDTMMDSVNRLASSGKISKSKLQALNMAFASSMAEIAATEEGGMSIGAKTSAIANALRGAFLQTTGYANEQFINEITSLVSMIAQANANSVSASASASAGGGGGGGYGGSSYGPSGYGPSQQQSSASSVSVSASAAGAGPRGQAPSGPAQQGPRGYGPSGPGGASAAAAAAASGPRGQGPYGPAQQGPGARGPSGPSQQGPGPQGPGGYGPSGPGSASAAAAAASTGGQGPSGQGQQGPGGYGQSGPGQQGPGGYGPSGPGSASAAAAAASAGGQGPSGQGQQGPGGYGQSGPGQQGPGGYGPSGPGSASAAAAAASAGGQGPSGQGQQGPGGYGQSGPGQQGPGGYGPSGPGSASAAAAAASAGGQGPSGQGQQGPGGYGQSGQGQRGPGGYGPSGPSGAAAAAASAGGQGPYGQGQQGPGQQGPGAGGYGPGGASAAAAAAAAGGPGGQGPYGPGQGAGGPYGPGAQGPGSQGPGGYGPSGPGSASAAAAAAAAGGQGPSGQGQQGPGGYGPSGPSGAAAAAAAAGGQGPYGQGQQGPGQQGPGAGGYGPGGASAAAAAAAAGGPGGQGPYGPGQGAGGPYGPGAQGPGSQGPGGYGPSGPGSASAAAAAAAAGGQGPSGQGQQGPGGYGPSGPSGAAAAAAAAGGQGPYGQGQQGPGQQGPGAGGYGPGGASAAAAAAAAGGPGGQGPYGPGQGAGGPYGPGAQGPGSQGPGGYGPSGPGSASAAAAAAAAGGQGPSGQGQQGPGGYGPSGPSGAAAAAAAAGGQGPYGQGQQGPGQQGPGAGGYGPGGASAAAAAAAAGGPGGQGPYGPGQGAGGPYGPGAQGPGSQGPGGYGPSGPGSASAAAAAAAAGGQGPSGQGQQGPGGYGPSGPSGAAAAAAAAGGQGPYGQGQQGPGQQGPGAGGYGPGGASAAAAAAAAGGPGGQGPYGPGQGAGGPYGPGAQGPGSQGPGGYGPSGPGSASAAAAAAAAGGQGPSGQGQQGPGGYGPSGPSGAAAAAAAAGGQGPYGQGQQGPGQQGPGAGGYGPGGASAAAAAAAAGGPGGQGPYGPGQGAGGPYGPGAQGPGSQGPGGYGPSGPGSASAAAAAAAAGGQGPSGQGQQGPGGYGPSGPSGAAAAAAAAGGQGPYGQGQQGPGQQGPGAGGYGPGGASAAAAAAAAGGPGGQGPYGPGQGAGGPYGPGAQGPGSQGPGGYGPSGPGSASAAAAAAAAGGQGPSGQGQQGPGGYGPSGPSGAAAAAAAAGGQGPYGQGQQGPGQQGPGAGGYGPGGASAAAAAAAAGGPGGQGPYGPGQGAGGPYGPGAQGPGSQGPGGYGPSGPGSASAAAAAAAAGGQGPSGQGQQGPGGYGPSGPSGAAAAAAAAGGQGPYGQGQQGPGQQGPGAGGYGPGGASAAAAAAAAGGPGGQGPYGPGQGAGGPYGPGAQGPGSQGPGGYGPSGPGSASAAAAAAAAGGQGPSGQGQQGPGGYGPSGPSGAAAAAAAAGGQGPYGQGQQGPGQQGPGAGGYGPGGASAAAAAAAAGGPGGQGPYGPGQGAGGPYGPGAQGPGSQGPGGYGPSGPGSASAAAAAAAAGGQGPSGQGQQGPGGYGPSGPSGAAAAAAAAGGQGPYGQGQQGPGQQGPGAGGYGPGGASAAAAAAAAGGPGGQGPYGPGQGAGGPYGPGAQGPGSQGPGGYGPSGPGSASAAAAAAAAGGQGPSGQGQQGPGGYGPSGPSGAAAAAAAAGGQGPYGQGQQGPGQQGPGAGGYGPGGASAAAAAAAAGGPGGQGPYGPGQGAGGPYGPGAQGPGSQGPGGYGPSGPGSASAAAAAAAAGGQGPSGQGQQGPGGYGPSGPSGAAAAAAAAGGQGPYGQGQQGPGQQGPGAGGYGPGGASAAAAAAAAGGPGGQGPYGPGQGAGGPYGPGAQGPGSQGPGGYGPSGPGSASAAAAAAAAGGQGPSGQGQQGPGGYGPSGPSGAAAAAAAAGGQGPYGQGQQGPGQQGPGAGGYGPGGASAAAAAAAAGGPGGQGPYGPGQGAGGPYGPGAQGPGSQGPGGYGPSGPGSASAAAAAAAAGGQGPSGQGQQGPGGYGPSGPSGAAAAAAAAGGQGPYGQGQQGPGQQGPGAGGYGPGGASAAAAAAAAGGPGGQGPYGPGQGAGGPYGPGAQGPGSQGPGGYGPSGPGSASAAAAAAAAGGQGPSGQGQQGPGGYGPSGPSGAAAAAAAAGGQGPYGQGQQGPGQQGPGAGGYGPGGASAAAAAAAAGGPGGQGPYGPGQGAGGPYGPGAQGPGSQGPGGYGPSGPGSASAAAAAAAAGGQGPSGQGQQGPGGYGPSGPSGAAAAAAAAGGQGPYGQGQQGPGQQGPGAGGYGPGGASAAAAAAAAGGPGGQGPYGPGQGAGGPYGPGAQGPGSQGPGGYGPSGPGSASAAAAAAAAGGQGPSGQGQQGPGGYGPSGPSGAAAAAAAAGGQGPYGQGQQGPGGYGPSGPSGAAASAAAAGGQGPYGQGQQGPGGYGPSGPSGAAAAAAAAGGQGPYGQGQQGPGGYGPSGPASGVSASVSSAASRLSSPAASSRVSSAVSTLASSGPSNAGVVSSALSNLVSQVSANHPGLSGCDVIVQALLELVSALVHILGSSSVGQVDYNGASYSAQSLGQAVAQALG

>Aarg_MaSp2.1b

MSCPRLVLAFLALLSTHALFASAAGATPWSTPALADSFMRSFIGGISSSGAFTGGQIDDMSTISDTMTDSVNRLASSGKISKSKLQALNMAFASSMAEIAATEEGGLSIGAKTSAIADALRGAFLQTTGYSNEQFINEITSLVSMIAQANANSVSASASASSGGGGGGGYGGSSYGPSSVSSASASASAAGAGAGPAQQGSGSYGPSGPGGYGPSGSSAAAAASGGQGPGNYGPSGSGGAGPSGPGGYGPGSQGSSGSGNQGPGAAASAAAAASGPGGYGPGSQGPSGPGGYGPGSQGPGGAGGYGPGSQGPGGSGAAAAAAAASSGPGGYGPGSQGPSGPGSQGSSGPGGASAAAAAASSGPGGYGPGSQGPSGPGGYGPGSQGPSGPGGYGPGSSGPGGYGPGSQGPGAASAAAAASGPGGYGPGSQGPSGAGSQGPSGPGSQGPSGPGGASAAAAAASSGPGGYGPGSQGPSGPGGYRPGSQGPSGPGGFGPGSSGPGGYGPGSQGPGAASAAAAASGPGGYGPGSQGPSGAGSQGPSGPGSQGPSGPGGASAAAAAASSGPGGYGPGSQGPSGLGGYGPGSQGPSGPGGYGPGSSGPGGAGGYGPGSQGPGGSGAAAAAAAASSGPGGYGPRSQGPSGPGSQGPSGPGGYGPGSSGPGGYGPGSQGPGAAPAAAAASGPGGYGPGSQGPGAASAAAAASGPGGYGPGSQGPSGPGSQGPSGPGGASAAAAAASSGPGGYGPGSQGPSGLGGYGPGSQGPSGPGGYGPGSSGPGGAGGYGPGSQGQGGSGAAAAAAAASSGPGGYGPRSQGPSGPGSQGPSGPGGYGPGSSGPGGYGPGSQGPGAASAAAAASGPGGYGPGSQGPGAASAAAAASGPGGYGPGSQGPSGPGSQGPSGPGGASAAAAAASSGPGGYGPGSQGPSAPGGYGPGSQGPSGPGGYGPGSSGPGGYGPGSQGPGAASAAAAASGPGGYGPGSQGPSGAGSQGPSGPGSQGPSGPGGASAAAAAASSGPGGYGPGSQGPSGLGGYGPGSQGPSGPGGYGPGSSGPGGAGGYGPGSQGPGGSGAAAAAAAASSGPGGYGPRSQGPSGPGSQGPSGPGGASAAAAAASSGPGGYGPGSQGSSGPGGYGPGSSGPGGYGPGSQGPGAAAAAAASSGPGGYGPGSQGPSGPGSQGPAGPGGYGPGSSGPGGAGGYGPGSQGPGGSGAAAAAAAASSGPGGYGPGSQGPSGPGSQGPSGPLSQGPSGPGSQGPSGPGGASAAAAAASSGPGGYGPGSQGPSGPGGYGPGSQGPSGPGGYGPGSQGPSGPGGYGPGPSGPGAAGGYGPGSQGPGGSGAAAAAAAASSGPGGYGPGSQGPSGPXSQGPSGPGGASAAAAAASSGPGGYGPGSQGPSGPGGYGPGNQGPSGPGGYGPGSSGPGGYGPGSQGPGAASAAAAASGPGGYGPGSQGPSGPGSQGPSGPGGASAAAAAASSGPGGYGPGSQGPSGPGGYGPGSQGPSGPGGYGPGSSGPGGYGPGSQGPGAASAAAAASGPGGYGPGSQGPSGAGIQGPSGPGIQGPSGPGGASAAAAAASSGPGGYGPGSQGPSGLGGYGPGSQGPSGPGGYGPGSSGPGGAGGYGPGSQGPGGSGAAAAAAAASSGPGGYGPRSQGPSGPGSQGPSGPGGYGPGSSGPGGYGPGSQGPGAASAAAAASGPGGYGPGSQGPSGPGGASAAAAAASSGPGGYGPGSQGSSGPGGYGPGSQGPSGPGGYGPGSSGPGGYGPGSQGPGAAAAAAASSGPGGYGAGSQGPSGPGSQGPSGPLSQGPSGPGSQGPSGPGGASAAAAAASSGPGGYGPGSQGPSGPGGYGPGNQGPSGPGGYGPGSSGPGGYGPGSQGPGAASAAAAASGPGGYGPGSQGPSGPGSQGPSGPGSQGPSGPGSQGPSGPGGASAAAAAASSGPGGYGPGSQGPSGPGGYGPGSQGPSGPGGYGPGSQGPSGPGGYGPGSSGPGGAGGYGPGSQGPGGSGAAAAAAAASSGPGGYGPGSQGPSGPGGYGPGSQGPSGPGGAGGYGPGSQGLGGSGAAAAAAAASSGPGGYGPGSQGPSGPGGYGPGSQGPSGPGGYGPGSSGPGGYGPGSQGPGAASAAAAASGPGGYGPGSQGPSGPGSQGPSGPGGASAAAAAASSGPGGYGPGSQGPSGPGSQGPSGPGGYGPGSSGPGGYGPGSQGPGAASAAAAASGPGGYGPGSQGPSGAGSQGPSGPGSQGPSGPGGYGPGSSGPGGAGGYGPGSQGPGGSGAAAAAAAASSGPGGYGPGSQGPSGPGGYGPGSSGPGGAGGYGPGSQGPGRSGAAAAAAAASSGPGGYGPGSQGPSGPGGYGPGSSGPGGYGPGSQGPGAASAAAAASGPGGYGPGSQGPSGPGSQGPSGPGGASAAAAAASSGPGGYGPGSQGPSGPGGYGPGSQGPSGPGGYGPGSSGPGGAGGYGPGSQGPGGSGAAAAAAAASSGPGGYGPGSQGPSGPGSQGPSGPGGASAAAAAASSGPGGYGPGSQGPSGPRGYGPGSQGPSGPGGNGPGSSGPGGYGPGSQGPGAASAAAAASGPGGYGPGSQGPSGPGSQGPSGPGGASAAAAAASSGPGGYGPGSQGPSGPGGYGPGSQGLSGPGGYGPGSSGPGGAGGYGPGSQGPGGSGAAAAAAAASSGPGGYGPGSQGPSGPGSQGPSGPGGASAAAAAASSGPGGYGPGSQGPSGPGGYGPGSQGPSGPGGYGPGSSGPGGYGPGSQGPGAASAAAAASGPVGYGPGSQGPSGPGSQGPSGPGGASAAAAAASSGPGGFGPGSQGPSGPGGYGPGSQGPSGPGGYGPGSSGPGGAGGYGPGSQGPGGSGAAAAAAAASSGPGGYGPGSQGPSGPGSQGPSGPGGYGPGSSGPVGYGPGSQGPGAASAAAAASGPGGYGPGSQGPSGPGSQGPSGPGGANAAAAAASSGPGGYGPGSQGPSGPGGYGPGSQGPSGPGGYGPGSSGPGGYGPGSQGPGAASAAAAASGPGGYGPGSQGPSGPGSQGPSGPGGASAAAAAASSGPGGYGPGSQGPSGPGGYGPGSQGPSGPGGYGPGSQGPSGPGGYGPGSSGPGGYGPGSQGPGAAAAAAAASGPGGYGPGSQGPSGPGGAGGNGPGSQGPGGSGGYGPGNQGPGGAGAAASAAASSGPSGPGGYRPGVQGPSNAGGYGSSAPASVSVAASRLSSPAASSRVSSAVTSLVSSGPTNGASVSGALNGLVSQISSSNPGLSGCDVLVQALLELVSALVAILGSASIGAVDYNSVGQTTQTISQYFS

>Aaur_MaSp2.1a

MSCPRLVLAFLALLSTNALFAAAAAATPWDSPALADSFMKSFMDGIGTSGAFTSSQIDDMSTIGDTMMDSVNRLASSGRISKSKLQALNMAFASSMAEIAATEEGGLSIGAKTSAIASALRGAFLQTTGYANEQFINEITSLINMIAQANVNAVSASASASAGGGYGAPAYGPSSYGPSQQQSSASSVSVSASAAGAGPRSQAPSRPAQQGPRGYGPSGPGGTAAASASAGGPGSQGPYGPGQQGPGPRGPSRPSQQGPGGYGPSGPGGASAAAAAAAAGGPGGQGPYGPGQQGPGAGPYGPGQQGPGQQGPGGYGPSGPGGAAAAAAAAAAGGPGGQGPSGPGQQGPGGYGPSGPSGASAAAAAAGGQGPYGQGQQGPRGYGPSGPGGTAAAAAAAGGPGGQGQYGPGQQGPGGYGSSGTGGASAAAAAAAAGGPGGQGPYGPGQQGPYGPGQQGPGGQGRGGYGPSGPGGASAAAAAAAAGGPGGQGQYGPGQQGPGGYGPSGPGGASAAAAASAAGGPGGQGPSGPGGQGPSGPGQQGPGGYGPSGPSGASAAAAVAGGQGPYGQGQQGPGGYGPSGPAGASAASAAAAAGGQGGQGPYGPGQQGPYGPGQQGPGGQGRGGYGPSGPGGASAAAAAAAAGGPGGRGEYGPGQQGPGGYGPSGPGGASAAAAASAAGGPGGQGPSGPGQQGPGGYGPSGPSGASAAAAVAGGQGPYGQGQQGPGGYGPSGPAGASAASAAAAAGGQGGQGPYGPGQQRPYGPGQQGPGGQGRGGYGPSGPGGASAAAAAAAAGGPGGQGQYGPGQQGPGGYGPSGPGGASAAAAASAAGGPGGQGPSGPGQQGPGGYGPSGPSGASAAAAVAGGQGPYGQGQQGPGGYGPSGPAGASAASAAAAAGGQGGQGPYGTGQQGPYGPGQQGPGGQGRGGYGPSGPGDASAAAAAAAAGGPGGQGQYGPGQQGPGGYGPSGPGGASAAAAASAAGGPGGQGPSGPGQQGPGGYGPSGPSGASAAAAVAGGQGPYGQGQQGPGGYGPSGPAGASAASAAAAAGGQGGQGPYGPGQQGPYGPGQQGPGGQGPGGYGPSGPGGASAAAAAAAAGGPGGQGQYGPGQQGPGGYGPSGPGGASAAAAASAAGGPGGQGPSGPGQQGPGGYGPSGPSGASAAAAVAVGQGPYGQGQQGPGGYGPSGPAGASAASAAAAAGGQGGQGPYGPGQQGPYGPGQQGPGQQGPGGYGPSGPGGSAAAAAAAAAGGPGGQGPSGPGQQGPGGYGPSGPSGASAAAAAAGGQGPYGQGQQGPGGYGPSGPAGASAASAAAAAGGQGGQGPYGPGQQGPYGPGQQGPGGQGPGGYGPSGPGGASAAAAAAAAGGSGGQGPYGPGQQGPYGPGQQGPGQQGPGGYGPSGPSGASAAAAAAGGQGPYGQGQQGPGGYGPSGPAGASAASAAAAAGGQGGQGPYGPGQQGPYGPGQQGPGGQGPGGYGPSGPGGASAAAAAAAAGGSGGQGPYGPGQQGPYGPGQQGPGQQGPGGYGPSGPSGASAAAAAAGGQGPYGQGQQGPGGYGPSGPAGASAASAAAAAGGQGGQGPYGPGQQGPYGPGQQGPGGQGPGGYGPSGPGGASAAAAAAAAGGSGGQGPYGPGQQGPYGPGQQGPGQQGPGGYGPSGPGGTAAAAAAAAAGGPAGQGPSGPGQQGPGGYGPSGPSGASAAAAAAGGQGPYGQGQQGPGGYGPSGPAGASAASAAAAAGGQGGQGPYGPGQQGPYGPGQQGPGGQGPGGYGPSGPGGASAAAAAAAAGGSGGQGPYGPGQQGPYGPGQQGPGQQGPGGYGPSGPSGASAAAAVAGGQGPYGQGQQGPGGYGPSGPAGASAASAAAAAGGQGGQGPYGPGQQGPYGPGQQGPGGQGPGGYGPSGPGGASAAAAAAAAGGSGGQGPYGPGQQGPGQQGPGGYGPSGPSGASAAAAAAGGQGPYGQGQQGPGGYGSSGSGGAAAAAATAGGPGGQGQYGPGQQGPGGYGPSGPGAASAAAAAAAAGGPGGQGLSGPGQQGPGGYGPSGPSGASAAAAVAGGQGPYGQGQQGPGGYGPSGPAGVSAASAAAAAGGQGGQGPYGPGQQGPYGPGQQGPGGQGPGGYGPSGPGGASAAAAAAAAGGSGGQGPYGPGQQGPYGPGQQGPGGQGPGGYGPSGPGGSAAAAAAAASAAAGGAGGQGPSGPGQQGPESYGPSGPSGASAATAAAGGQGPYGQGQQGPGGYGPSGPVSGVSVSVSSAASRLSSPAASSRVSSAVSTLASSGPSDAGVVSSALSNLVSQVSTNHPGLSECDVIVQALLELVSALVHILGSSSVGQVDYNGASYSAQNLGQAVAQALA

>Aaur_MaSp2.1b

MSCPRLVLAFLALLSTHALFAAAAGATPWDSPALADSFMKCFMDGIGTSGAFTSSQIDDMSTIGDTMIDSVNRLASSGRISKSKLQALNMAFASSMAEIAATEEGGLSIGAKTSAIASALRGAFLQTTGYSNEQFINEITSLVSMIAQANTNSVSASASASAGGGYGGSSYGPSSVSSVSASASSAGAGPAQQGPGSYGPSGPGGYGPSGSSAAAAASGGQGPGNYGSSGSGGAGPSGPGGYGPGSQGSSGPGNQGPGGVSAAAAAASGPGGYGPGSQGSSGPGGYGPLSQGQSGPGGAGGYGPGGASAAAAAVAASGPAGYGQGSQGPSGTGASGPGGAGGYGPGSQGPGGAAAAAAASGPGGYGPGSQGPSGPGGFGPGSQGQSGPGGYGPGNQGQSGPSGAGGYGPGGASAAAAAAAASGPGGYGPGSQGPYGPGSQGPSGPGGYGSGSSGPGGAGGYGPGSQGPGGQGTAAAAAAASGPGGYGSGNQGPSGTGASGPGGAGGYGPGSQGPGAAAAAAAASGPAGYGPGSQGQSGPGSQGPGGASAAAAAAASGPGGYGPGSQGPSGPSGYGPGASGPGGAAGYGPGSQGPGAASAAAAAAAASGSGGYGPGSQGPYGPGSQGPSGSGSQGPSGSGGYGPGASGPGGAGSYGPGSQGPGGASAAAAAAASAPGGYGPGSQGSSGPSGYGPGASGPGGAGGYGPGSQGPGGASAAAAAAAASGPGGYGPGSQGPYGPGNQGPSGPGSQGPSGSGGYGPGASGPGGTGSYGPGSQGPGGASAAAAAAASAPGGYGPGSQGLSGPSGYGPGASGPSGAGGYGPGSQGPGGASAAAAAAAASGPGGYGPGSQGPYGPGSQGPSGPGSQGPSGSGGYGPGASGPGGYGPGSQGPGGASAAAAAAASGPGGYGPGSQGQSGPGGYGSGSSGPGGAGGYGPGSQGPGGASAAAAAAASGPGGYGPGSQGPSGPGSQGPSGPGSQGPSGSYGYGPGASGPGGAGSYGPGSQGPGGASAAAAAAASGPGGYGPGSQGPSGPGSQGPSGSGGYGPGASGPGGYGPGSQGPGGASAAAAAAASGPGGYGPGSQGQSGPGGYGSGASGPGGAGGYGPGSQGPGGASAAAAAAASGPGGYGPGSQGPSGPGSQGPSGPGSQGPSGSGGYGPGASGPGGAGSYGPGSQGPGGASAAAAAAASGPEGYGPGSGGPSGPSGYGPGASGPGGTGGYGPGSQGPGGASAAAAAAASGPGGYGPGSQGPSGPGSQGPSGPGSQGPSGSGGYGPGASGPGGYGPGSQGPGGASAAAAAAASGPGGYGPGSQGQSGPGGYGSGSSGPGGAGGYGPGSQGPGGASAAAAAAASGPGGYGPGSQGPSGPGSQGPSGPGSQGPSGSYGYGPGASGPGGAGSYGPGSQGPGGASAAAAAAASGPGGYGPGSQGPSGPGSQGPSGSGGYGPGVYGPGSQGPGGASAAAAAAASGPGGYGPGSQGQSGPGGYGSGASGPGGAGGYGPGSQGPGGASAAAAAAASGPGGYGPGSQGPSGPGSQGPSGPGSQGPSGSGGYGPGASGPGGAGSYGPGSQGPGGASAAAAAAASGPGGYGPGSGGPSGPSGYGPGASGLGSAGGYGRGSQGPGGASAAAAAAASGPGGYGPGSQGPSGPGSQGPSGSGGYGPGASGPGGYGPGSQGPGGASAAAAAAASGPGGYGPGSQGQSGPGGYGSGSSGPGGAGGYGPGSQGPGGASAAAAAAASGPGGYGPGSQGPSGPGSQGPSGPGSQGPSGSYGYGPGASGPGGAGSYGPGSQGPGGASAAAAAAASGPGGYGPGSQGPSGPGSQGPSGSGGYGPGASGPGVYGPGSQGPGGASAAAAAAASGPGGYGPGSQGQSGPGGYGSGASGPGGAGGYGPGSQGPGGASAAAAAAASGPGGYGPGSQGSSGPGSQGPSGPGSQGPSGSGGYGPGASGPGGAGSYGPGSQGPGGASAAAAAAASGPGGYGPGSGGPSGPSGYGPGASGPGSAGGYGPGSQGPGGASAAAAAAASGPGGYGPGSQGPAGPGSQGPSGPGSQGPSGSGGYGPGASGPGGYGPGSQGPGGASAAAAAAASGPGGYGPGSQGQSGPGGYGSGSSGPGGAGGYGPGSQGPGGASAAAAAAASGPGGYGPGSQGPSGPGSQGPSGPGSQGPSGSGGYGPGASGPGGAGSYGPGSQGPGGASAAAAAAASGPGGYGPGSQGPSGPGSQGPSGSGGYGPGASGPGVYGPGSQGPGGASAAAAAAASGPGGYGPGSQGPSGPGSQGPSGPGSQGPSGSGGYGPGASGPGGAGSYGPGSQGPGGASAAAAAAASGPGGYGPGSGGPSGPSGYGPGASGPGSAGGYGPGSQGPGGASAAAAAAASGPGGYGPGSQGPSGPGSQGPSGPGSQGPSGSGGYGPGASGPGGYGPGSQGPGGASAAAAAAASGPGGYGPGSQGQSGPGGYGSGSSGPGGAGGYGPGSQGPGGASAAAAAAASGPGGYGPGSQGPSGPGSQGPSGPGGAGAAAAAAAASGPGGYGPGSQGSSGPGSQGPSGSGGYGPGASGSGGYGPGSQGPGGASAAAAAAASGPGGYGPGSQGPSGPGSQGPSGPSVYGPGASGPGGAGGYGPGSQGPGGASAAAAAASGPGGYGPGSQGPSGPGSQGPSGPGSQGPSGSGGYGPGASGPGGYGPGSQGPGGASAAAAAAASGPGGYGPGSQGPSGPGSQGPSGPSGYGPGASGPGGAGGYGPGSQGPGGASAAAAAASGPGGYGPGGQGPSGPGSQGPSGPGGSGAAAAAAASGPGGYGPGSQGLSGPGSQGSSGLGGYGPGGAGGYGPGSQGPGGASAAAAAAAASGPGGYGPGSQGPSGPGSQGPSGPGSQGPSGSGGYGPGASGSGGYGPGSQGPGGASAAAAAAASGPGGYGPGSQGPSGPGSQGPSGPSGYGPGASGPGGAGGYGPGSQGPGGASAAAAAASGPGGYGPGSQGTSGPGSQGPSGPGSQGPSGSGGYGPGASGPGGYGPGSQGSGGASAAAAAAASGPGGYGPGSQGSSGLGGYGPGGAGGYGPGSQGPGGASAAAAAAATSGPGGYGPGSQGPSGPGSQGPSGPGSFGPGGAGGYGPSASATVSVAASRLSSPAASSRVSSTVSSLVSSGPSNGAAVSGALNGLVSQISSSNPGLSGCDVLVQALLELVSALVAILGSANIGSVDYYSVGQTTQTISQYFS

>Atri_MaSp2.1a

MSCPRLVLAFLALLSTHALFASAGGQTPWDSPALADSFMKSFMDGIGASGAFSSSQIDDMSTIGDTMMDSVNRLASSGRISKSKLQALNMAFASSMAEIAATEEGGLSIGAKTSAIASALRGAFLQTTGYSNEQFINEITSLVSMIAQANVNTVSASASAAAGGGYGSPAYGPSSYGPSQQQSSASSVSVSASAAGPGPRGQAPSRPAQQGSAGYGPSGPGGAAAAAAAAGPGQQRPSGPSQQGPGSYGPSGPGGASAAAAAAAAGGPGGQGQYGPGQQGPGAYRQQGPGQQGPGGYGPSGPGGASAAAAAAAAGGPGGQGPYGPGQQGPGGYGPSGPGGASAAAAAAAAGGPGGQGPYGPGQQGPGAGQYGPGQQGPGGRGPGGYGPSGPGGASAAAAAAAAGGPGGQYGPGQQGPGSGGPYGQQGPGQQGPGGYGPSGPGGASAAAAAAAAGGPGGQGPSGPGQQGPGGYGPSGPGAAAAAAAAAGGPGSQGPGQQGPGGYGPSGPGGASAAAAAAAAGGPGGQGSYGPGQQGPGAGQYGPGQQGPGGRGPGGYGPSGPGGASAAAAAAAAGGPGGQYGPGQQGPGSGGPYGQQGPGQQGPGGYGPSGPGGASAAAAAAAAGGPGGQGPSGPGQQGPGGYGPSGPGAAAAAAAAAGGPGSQGPGQQGPGGYGPSGPGGASAAAAAAAAGGPGGQGSYGPGQQGPGAGQYGPGQQGPGGRGPGGYGPSGPGGASAAAAAAAAGGPGGQYGPGQQGPGSGGPYGQQGPGQQGPGGYGPSGPGGASAAAAAAAAGGPGGQGPSGPGQQGPGGYGPSGPGAAAAAAAAAGGPGSQGPGQQGPGGYGPSGPGGASAAAAAAAAGGPGGQGSYGPGQQGPGAGQYGPGQQGPGGRGPGGYGPSGPGGASAAAAAAAAGGPGGQYGPGQQGPGSGGPYGQQGPGQQGPGGYGPSGPGGASAAAAAAAAGGPGGQGPSGPGQQGPGGYGPSGPGAAAAAAAAAGGPGSQGPGQQGPGGYGPSGPGGASAAAAAAAAGGPGGQGSYGPGQQGPGAGQYGPGQQGPGGRGPGGYGPSGPGGASAAAAAAAAGGPGGQYGPGQQGPGSGGPYGQQGPGQQGPGGYGPSGPGGASAAAAAAAAGGPGGQGPSGPGQQGPGGYGPSGPGAAAAAAAAAGGPGSQGPGQQGPGGYGPSGPGGASAAAAAAAAGGPGGQGSYGPGQQGPGAGQYGPGQQGPGGRGPGGYGPSGPGGASAAAAAAAAGGPGGQYGPGQQGPGSGGPYGQQGPGQQGPGGYGPSGPGGASAAAAAAAAGGPGGQGPSGPGQQGPGGYGPSGPGAAAAAAAAAGGPGSQGPGQQGPGGYGPSGPGGASAAAAAAAAGGPGGQGSYGPGQQGPGAGQYGPGQQGPGGRGPGGYGPSGPGGASAAAAAAAAGGPGGQYGPGQQGPGSGGPYGQQGPGQQGPGGYGPSGPGGASAAAAAAAAGGPGGQGPSGPGQQGPGGYGPSGPGAAAAAAAAAGGPGSQGPGQQGPGGYGPSGPGGASAAAAAAAAGGPGGQGSYGPGQQGPGAGQYGPGQQGPGGRGPGGYGPSGPGGASAAAAAAAAGGPGGQYGPGQQGPGSGGPYGQQGPGQQGPGGYGPSGPGGASAAAAAAAAGGPGGQGPSGPGQQGPGGYGPSGPGAAAAAAAAAGGPGSQGPGQQGPGGYGPSGPGGASAAAAAAAAGGPGGQGSYGPGQQGPGAGQYGPGQQGPGGRGPGGYGPSGPGGASAAAAAAAAGGPGGQYGPGQQGPGSGGPYGQQGPGQQGPGGYGPSGPGGASAAAAAAAAGGPGGQGPSGPGQQGPGGYGPSGPGAAAAAAAAAGGPGSQGPGQQGPGGYGPSGPGGASAAAAAAAAGGPGGQGSYGPGQQGPGAGQYGPGQQGPGGRGPGGYGPSGPGGASAAAAAAAAGGPGGQYGPGQQGPGSGGPYGQQGPGQQGPGGYGPSGPGGASAAAAAAAAGGPGGQGPSGPGQQGPGGYGPSGPGAAAAAAAAAGGPGSQGPGQQGPGGYGPSGPGGASAAAAAAAAGGPGGQGSYGPGQQGPGAGQYGPGQQGPGGRGPGGYGPSGPGGASAAAAAAAAGGPGGQYGPGQQGPGSGGPYGQQGPGQQGPGGYGPSGPGGASAAAAAAAAGGPGGQGPSGPGQQGPGGYGPSGPGAAAAAAAAAGGPGSQGPGQQGPGGYGPSGPGGASAAAAAAAAGGPGGQGSYGPGQQGPGAGQYGPGQQGPGGRGPGGYGPSGPGGASAAAAAAAAGGPGGQYGPGQQGPGSGGPYGQQGPGQQGPGGYGPSGPGGASAAAAAAAAGGPGGQGPSGPGQQGPGGYGPSGPGAAAAAAAAAGGPGSQGPGQQGPGGYGPSGPGGASAAAAAAAAGGPGGQGSYGPGQQGPGAGQYGPGQQGPGGRGPGGYGPSGPGGASAAAAAAAAGGPGGQYGPGQQGPGSGGPYGQQGPGQQGPGGYGPSGPGGASAAAAAAAAGGPGGQGPSGPGQQGPGGYGPSGPGAAAAAAAAAGGPGSQGPGQQGPGGYGPSGPGGASAAAAAAAAGGPGGQGSYGPGQQGPGAGQYGPGQQGPGGRGPGGYGPSGPGGASAAAAAAAAGGPGGQYGPGQQGPGSGGPYGQQGPGQQGPGGYGPSGPGGASAAAAAAAAGGPGGQGPSGPGQQGPGGYGPSGPGAAAAAAAASGGPGSQGPGQQGPGAYGPSGPGGASAAAAAAAAGGPGGQGPYGPGQQGPGAGQYGPGQQGPGQQGPGGYGPSGPSGAAAAAAAAAAGGQGPYGPGQQGPGGYGPSGPAQQGPGSYGPSGPSGAAAAAAAAGGQGPYGQRQQGPGGYGPSGPVSGISASVSSAASRLSSPAASSRVSSAVSTLASSGPSDAGVVSSALSNLVSQVSSNHPGLSGCDVIVQALLELVSALVHILGSSSLGQVDYNGASYSAQTLGQAVAQALA

>Atri_MaSp2.1b

MSYPRLVLAFLALLSTHALFAAAGGQTPWDTPTLADNFMKCFMNEIGNSGAFTSNQVDDMSTIGDTMMDSVNRLASSGRISKSKLQALNMAFASSMAEIAATEEGGLSIGSKTNAIASALRGAFLQTTGYSNEQFINEITSLVSMIAEANVNTVSASASAYAGGGYGGSSYGSSSVNSASAAATGPAQQGPGSYGPSSVPGGYGPSGSSAAAAASGGQGLGNYGPSGSGGAGPSGTGGYGPGSQGPSRPSGPGAAAAAAAASGPGGYGPSAPSGPGSQGPSGPSGSGASAAAAAASGSGGYGSGSQGPSGPGSQGPSGPGTSAAAAAAANGPGGYGPGSQGPSGPGGYRPGSQGPSGPGSSGPGMSGGYGPGNQGPGGASVAAAAAGSGPGGYGPGSQGPSGPGSSGPGMSGGYGPGNQGPGGASAAAAAASGPGGYGPGSQGSSGPGAYGPGSQGSSGPGSSGPGMSGGYGPGNQGPGGASAAAAAAASGPGGYGPXSKGPGRHXGPGSSGPGMSGGYGPGNQGPGGASAAAAAAASGPGGYGPGSQGPSGPGAYGPGSQGSSGPGSSGPGMSGGYGPGNQGPGGASAAAAAAASGPGGYGPGSQGPSGPGAYGPGSQGSSGPLSSGPDMSGGYGPGNQGPGGASAAAAAAASGPGGYGPGSQGPSGPGAYGPGSQGSSGPGSSGPGMSGGYGPGNQGPGGASAAAAAVASGPGGYGPGSQGSSGTGAYGTGSQGSTGPGSSGPGMSGGYGPGNQGPGGASAAAAAAASGPGGYGPGSQGSSGPGAYGPGSQGSSRPLSSGPGMSGGYGPGNQGPGGASAAAAAAASGPGGYGPGSQGPSGPGAYGSGSQGSSGPGSSGPGMSGGYGPGNQGPGRASAAAAAAASGPGGYGPGSQGPSGPGAYGPGSQGSSGPLSYGTDMSGGYGPGNQGPGGASAAAAAAASGPGGYGPGSQGPSGPGAYGPGSQGSSGPGSSGPGMSGGYGPGNQGPGGASAAAAAEASGPGAYGPGSQGSSRLLSSGPGMSGGYGPGNQGPGGASAAAYAAASGLGGYGPGSQGPSGPGAYGSGSQGSSGPGSSGPGMSGGYGSGNQGPGRASAAAAAAAIGPGGYGPGSQGSSGTGAYGPGSQGSSGPLSSGPGMSGGYGPGNQGPGGASAAAAAAASGPGGYGPGSQGPSGPGAYGPGSQGSSGPLSYGTDMSGGYGPGNQGPGGASAAAAAAASGPGGYGPGSQGSSGPGAYGTGSQGSTGPGSSGPGMSGGYGPGNQGPGGANAAAAAAASGPGGYGPGSQGSSGPGAYGPGSQGSSRPLSSGPGMSGGYEPGNQGPGGASAAAAAAASGPGGYGPGSQGPSGPGAYGPGSQGSSGPRSSGPGMSGGYGPGNQGPGGASAAAAAAASGPGAYGPGSQGSSGPGAYGPGSQGSSGPVSSGPDMSGGYGPGNQGPGGASAAAAAAASGPGGYGPGSQGPSGPGAYGPGSQGSSGPGSSGPGMSGGYEPGNQGPGGASAAAAAAASGPGGYGPGSQGPSGPGAYGPGSQGSSGPGSSGPGMSGGYGPGNQSTGGASAAAAAAASXPGAYGPGSQGSSGPGAYGPGSQGSSGPGSYGPGMSGGYGPGNQGPGGASAAAAAAASGPAGYGLGSQGSSGPGSSGPGMSGGYGPGNQGLGRASAVAAAAASGPGGYGPGSQGPSRPVAYGPGSQGPSVPGAYGPGSQGSSGPGSSGPGVSGSYGPGNQGPGGASAAAAAAASGPGAYGPGSQGSSGPGSFGPGMSGGYGPGNQGPGGASAAADAAASGPGGYGPGSQGPSGLGAYGPGSQGSSGPGSSGPGMSGGYGPGNQGPGGASAAAAASGPGSQGPSVLGGYGPGSQGPSGWGSQGPSAPSGYGPSASVSASAAASRLSSPAASSRVSSAVSSLVSSGPTSGAAVSGALNGLVSQISSNNPGLSGCDVLVQALLELVSALVAILGSASIGAVDYNSVGQTTQTISQYFS

>Aarg_MaSp2.2a

MNLSIRLALLGFVVLSTQTIFAAGQAATPWQNSQLAEQFINSFLRFIGQSGAFSPDQLDDMSTIGETLKTAIEKMAQSRNSSRSKLQALNMAFASSMAEIAVAEQGGLSLEAKTNAIASALTSAFLETTGVVNQQFVSEIKGLIYMIAQASSNEISGSAAASGGGSGGGGGGYGQGSYASASAAAAYGSAPQGAGGPASQGPSQQGPVSQPSYGPSATVVVSAVGGYGPVAGQQGPSGASQQGPGGQGPSGPVAAAAAVTGGYGPGAGAGGPQRPIGAGPSVPSARGPGAAGAGPQAGPGGPGGAGPSAAAAAAAGAGGFGPGAGGQQGPGGAGAYGPSAGGQRGPGGQGSYGPGAAATAAAAAAGGFGPGGAGAGPQAGPGQQGPGGQGPYGPGAAAAAAAAGGSGPGVGGYQGPGGAGQQEPGGQGPYGPGAAAAAAAAGGSGPGAGGQRGPGQQGPGGQGPYGPGAAAAAAAAAGGYGPGGAGAGPQAGPGGPGGAGPSAAAAAAAGAGGFGPGAGGQQGPGGAGAYGPSAGGQRGPGGQGPYGPGAAAAAAAAAGGFGPGGAGAGPQAGPGQQGPGGQGPYGPGAAAAAAAAGGSGPGAGGYQGPGGAGQQGPGGQGPYGPGAAAAAAAAGGSGPGAGGQRGPGQQGPGGQGPYGPGAAAAAAAAAGGYGPGGAGAGPQAGPGGPGGAGPSAAAAAAAGAGGYGPGAGGQQGPGGAGAYGPSAGGQRGPGGQGPYGPGAAAAAAAGGFGPGGAGAGPQAGPGQQGPGGQGPYGPGAAAAAAAAGGSGPGAGGYQGPGGAGQQGPGGQGPYGPGAAAAAAAAGGSGPGAGGQRGPGQQGPGGQGPYGPGAAAAAAAAAGGYGPGGAGAGPQAGPGGPGGAGPSAAAAAAAGAGGYGPGAGGQQGPGGAGAYGPSAGGQRGPGGQGPYGPGAAAAAAAGGFGPGGAGAGPQAGPGQQGPGGQGPYGPGAAAAAAAAGGSGPGAGGYQGPGGAGQQGPGGQGPYGPGAAAAAAAAGGSGPGAGGQRGPGQQGPGGQGPYGPGAAAAAAAAAGGYGPGGAGAGPQAGPGGPGGAGPSAAAAAAAGAGGFGPGAGGQQGPGGAGAYGPSAGGQRGPGGQGPYGPGAAAAAAAGGFGPGGAGAGPQAGPGQQGPGGQGPYGPGAAAAAAAAGGSGPGAGGYQGPGGAGQQGPGGQGPYGPGAAAAAAAAGGSGPGAGGQRGPGQQGPGGQGPYGPGAAAAAAAAAGGYGPGGAGAGPQAGPGGPGGAGPSAAAAAAAGAGGFGPGAGGQQGPGGAGAYGPSAGGQRGPGGQGPYGPGAAAAAAAGGFGPGGAGAGPQAGPGQQGPGGQGPYGPGAAAAAAAAGGSGPGAGGYQGPGGAGQQGPGGQGPYGPGAAAAAAAAGGSGPGAGGQRGPGQQGPGGQGPYGPGAAAAAAAAAGGYGPGGAGAGPQAGPGGPGGAGPSAAAAAAAGAGGFGPGAGGQQGPGGAGAYGPSAGGQRGPGGQGPYGPGAAAAAAAGGFGPGGAGAGPQAGPGQQGPGGQGPYGPGAAAAAAAAGGSGPGAGGYQGPGGAGQQGPGGQGPYGPGAAAAAAAAGGSGPGAGGQRGPGQQGPGGQGPYGPGAAAAAAAAAGGYGPGGAGAGPQAGPGGPGGAGPSAAAAAAAGAGGFGPGAGGQQGPGGAGAYGPSAGGQRGPGGQGPYGPGAAAAAAAGGFGPGGAGAGPQAGPGQQGPGGQGPYGPGAAAAAAAAGGSGPGAGGYQGPGGAGQQGPGGQGPYGPGAAAAAAAAGGSGPGAGGQRGPGGQGPYGPGAAAAAAAAAGGYGPGGAGAGPQAGPGGPGGAGPSAAAAAAAGAGGFGPGAGGQQGPGGAGAYGPSAGGQRGPGGQGPYGPGAAAAAAAGGFGPGGAGAGPQAGPGQQGPGGQGPYGPGAAAAAAAAGGSGPGAGGYQGPGGAGQQGPGGQGPYGPGAAAAAAAAGGSGPGAGGQRGPGQQGPGGQGPYGPGAAAAAAAAAGGYGPGGAGAGPQAGPGGPGGAGPSAAAAAAAGAGGFGPGAGGQQGPGGAGAYGPSAGGQRGPGGQGPYGPGAAAAAAAGGFGPGGAGAGPQAGPGQQGPGGQGPYGPGAAAAAAAAGGSGPGAGGYQGPGGAGQQGPGGQGPYGPGAAAAAAAAGGSGPGAGGQRGPGQQGPGGQGPYGPGAAAAAAAAAGGYGPGGAGAGPQAGPGGPGGAGPSAAAAAAAGAGGFGPGAGGQQGPGGAGAYGPSAGGQRGPGGQGPYGPGAAAAAAAGGFGPGGAGAGPQAGPGQQGPGGQGPYGPGAAAAAAAAGGSGPGAGGYQGPGGAGQQGPGGQGPYGPGAAAAAAAAGGSGPGAGGQRGPGQQGPGGQGPYGPGAAAAAAAAAGGYGPGGAGAGPQAGPGGPGGAGPSAAAAAAAGAGGFGPGAGGQQGPGGAGAYGPSAGGQRGPGGQGPYGPGAAAAAAAGGFGPGGAGAGPQAGPGQQGPGGQGPYGPGAAAAAAAAGGSGPGAGGYQGPGGAGQQGPGGQGPYGPGAAAAAAAAGGSGPGAGGQRGPGQQGPGGQGPYGPGAAAAAAAVAGGYGPGGAAAGPQAGPGGPGGAGPSAAAATAAGAGGFGPGAGGQQGPGGAGAYGPSAGGQRGPGGQGPYGPGAAAAAAAAAGGFGPGGAGPGPQAGPRGAQPYGPSAAAAVGGYGPGAGQQGPGRQGPAGPGQQGSGGQGPYGPGASAAAAAAGGYGPGAGQQGPRSQAPVASAAASRLASPQASSRVSSAASTLVSSGPANPAALSNTISSVVSQISASNPGLSGCDVLVQALLEIVSALVYILGSSSIGQINYGAASQYTQLVGRSVAQALG

>Aarg_MaSp2.2b

MNWSIRLALFGFVVLSTQTVFAVGQAATPWENSQLAEDFINSFLRFIAQSGAFSPNQLDDMSSIGDTLKTAIEKMAQSRKSSKSKLQALNMAFASSMAEIAVAEQGGLSLEAKTNAIASALASAFLETTGVVNQQFVSEIKGLIYMIAQASSNEISGSASGSGGGSGGGGGGGGGYGPGSYASASVAAAYGSAPQGAGGPSPQGPSQQAPISQGPYGPGAAAAAAASGGYGPGAGQQGPSGGGQQGPGGAGQQGPGGQGPYVPSAAAAAAGGYGPGAGQQGPGGAGQQGPGPQGPGGAGQRGPYGPGAAAAAAAAGGYGPGAGQQGPGSGGQQGPSGQGPYGPGASAAAAAAGGYGPGARQQGPGGQGAGSGGQQGPGSQGPGGAVQQGPYGPGAAAAAAAARGYGPGAGQQGPGGAGQQGPGSQGPGGAGQRGPYGPGAAAAAAAAGGSGPGAGQQGPGSGGQQGPSGQGPYGPGASAAAAAAGGYGPGAGQQGPGGQGAGSGGQQGPGSQGPGGAGQQGPYGPGAAAAAAAARGYGPGAGQQGSGRAGQQGPGSQGPGGAGQRGPYGPGAAAAAAAAGGSGPGAGQQGPGSGGQQGPSGQGPYGPGASAAAAAAGGYGPGAGQQGPGGQGAGSGGQQGPGSQGPGGAGQQGPYGPGAAAAGGYGPGAGQQGPRSGGQQGPSGQGPYGPGASAAAAAAGGYGPGAGQQGPGGQGAGSGGQQGPGSQGPGGAGQQGPYGPGAAAAAAAAGGYGPGGGQQGPGGAGQQGPGSQGPGGAGQRGPYGPGAAAAAAAAGGYGPGAGQQGPGSGGQQGPSGQGPYGPGASAAAAAAGGYGPGAGKQGPGGQGAGSGGQQGPGSQGPGGAGQQGPYGPGAAAAAAAAGGYGPGAGQQGPGSGGQQGPSGQGPYGPGASAAAAAAGGYGPGAGQQGPGGQGAGSGGQQGPGSQGPGGAGQQGPYGPGAAAAAAAARGYGPGAGQQGPGGAGQQGPVSQGPGGAGQQGPYGPGAAAAAAAAGGYGPGAGQQGPGGAGQQGPGSQGPGGAGQRGPYGPGAAAAAAAAGGYGPGAGQQGPGSGGQQGPSGQGPYGPGASAAAAAAGGYGPGAGQQGPGGQGAGSGGQQGPGSQGPGGAGQQGPYGPGAAAAAAAAGGYGPGAGQQGPGGAGQQGPGSQGPGGAGQRGPYGPGAAAAAAAAGGYGPGAGQQGPGSGGQQGPSGQGPYGPGASAAAAAAGGYGPGAGQQGPGGQGAGSGGQQGPGSQGPGGAGQQGPYGPGAAAAAAAAGGYGPGAGQQGPGGAGQQGPGSQGPGGAGQRGPYGPGAAAAAAAAGGYGPGAGQQGPGSGGQQGPSGQGPYGPGASAAAAAAGGYGPGAGQQGPGGQGAGSGGQQGPGSQGPGGAGQQGPYGPGAAAAAAAAGGYGPGAGQQGPGGAGQQGPGSQGPGGAGQRGPYGPGAAAAAAAAGGYGPGAGQQGPGSGGQQGPSGQGPYGPGASAAAAAAGGYGPGAGQQGPGGQGAGSGGQQGPGSQGPGGAGQQGPYGPGAAAAAAAAGGYGPGAGQQGPGGAGQQGPGSQGPGGAGQRGPYGPGAAAAAAAAGGYGPGAGQQGPGSGGQQGPSGQGPYGPGASAAAAAAGGYGPGAGQQGPGGQGAGSGGQQGPGSQGPGGAGQQGPYGPGAAAAAAAAGGYGPGAGQQGPGGAGQQGPGSQGPGGAGQRGPYGPGAAAAAAAAGGYGPGAGQQGPGSGGQQGPSGQGPYGPGASAAAAAAGGYGPGAGQQGPGGQGAGSGGQQGPGSQGPGGAGQQGPYGPGAAAAAAAAGGYGPGAGQQGPGGAGQQGPGSQGPGGAGQRGPYGPGAAAAAAAAGGYGPGAGQQGPGSGGQQGPSGQGPYGPGASAAAAAAGGYGPGAGQQGPGGQGAGSGGQQGPGSQGPGGAGQQGPYGPGAAAAAAAAGGYGPGAGQQGPGGAGQQGPGSQGPGGAGQRGPYGPGAAAAAAAAGGYGPGAGQQGPGSGGQQGPSGQGPYGPGASAAAAAAGGYGPGAGQQGPGGQGAGSGGQQGPGSQGPGGAGQQGPYGPGAAAAAAAARGYGPGAGQQGPGGAGQQGPGSQGPGGAGQQGPYGPGAAAAAAAAGGYGPGAGQQGPGGAGQQGPGSQGPGGAGQQGPYGPGAAAAAAAAGGYGPGAGQQGPGGAGQQGPGSQGPGGAGQRGPYGPGAAAAAAAAGGYGPGAGQQGPGSGGQQGPSGQGPYGPGASAAAAAAGGYGPGAGQQGPGGQGAGSGGQQGPGSQGPGGAGQQGPYGPGAAAAAAAAGGYGPGAGQQGPGGAGQQGPGSQGPGGAGQRGPYGPGAAAAAAAAGGYGPGAGQQGPGSGGQQGPSGQGPYGPGASAAAAAAGGYGPGAGQQGPGGQGAGSGGQQGPGSQGPGGAGQQGPYGPGAAAAAAAARGYGPGAGQQGPGGAGQQGPGSQGPGGAGQQGPYGPGAAAAAAAAGGYGPGAGQQGPGGAGQQGPGSQGPGGAGQRGPYGPGAAAAAAAAGGYGPGAGQQGPGSGGQQGPSGQGPYGPGASAAAAAAGGYGPGAGQQGPGGQGAGSGGQQGPGSQGPGGAGQQGPYGPGAAAAAAAAGGYGPGAGQQGPGGAGQQGPGSQGPGGAGQRGPYGPGAAAAAAAAGGYGPGAGQQGPGSGGQQGPSGQGPYGPGASAAAAAAGGYGPGAGQQGPGGQGAGSGGQQGPGSQGPGGAGQQGPYGPGAAAAAAAAGGYGPGAGQQGPGGAGQQGPGSQGPGGAGQRGPYGPGAAAAAAAAGGYGPGAGQQGPGSGGQQGLKFITYYSKGPSGQGPYGPGASAAAAAAGGYGPGAGQQGPGGQGAGSGGQQGPGSQGPGGAGQQGPYGPGAAAAAAAARGYGPGAGQQGPGGAGQQGPGSQGPGGAGQQGPYGPGAAAXXAAAGGYGPGAGQQGPGGAGQQGPGSQGPGGAGQRGPYGPGAAAAAAAAGGYGPGAGQQGPGSGGQQGPSGQGPYGPGASAAAAAAGGYGPGAGQQGPGGQGAGSGGQQGPGSQGPGGAGQQGPYGPGAAAAAAAAGGYGPGAGQQGPGGAGQQGPGSQGPGGAGQRGPYGPGAAAAAAAAGGYGPGAGQQGPGSGGQQGPSGQGPYGPGASAAAAAAGGYGPGAGQQGPGGQGAGSGGQQGPGSQGPGGAGQQGPYGPGAAAAAAAARGYGPGAGQQGPGGAGQQGPGSQGPGGAGQQGPYGPGAAAAAAGGYGPGAGQQGPGGAGQQGPGSQGPGGAGQRGPYGPGAAAAAAAAGGYGPGAGQQGPGSGGQQGPSGQGPYGPGASAAAAAAGGYGPGAGQQGPGGQGAGSGGQQGPGSQGPGGAGQQGPYGPGAAAAAAAARGYGPGAGQQGPGGAGQQGPGSQGPGGAGQQGPYGPGAAAAAAAGGYGPGAGQQGPGGAGQQGPGSQGPGGAGQRGPYGPGAAAAAAAAGGYGPGAGQQGPGSGGQQGPSGQGPYGPGASAAAAAAGGYGPGAGQQGPGGQGAGSGGQQGPGSQGPGGAGQQGPYGPGAAAAAAAAGGYGPGAGQQGPGGAGQQGPGSQGPGGAGQRGPYGPGAAAAAAAAGGYGPGAGQQGPGSGGQQGPSGQGPYGPGASAAAAAAGGYGPGAGQQGPGGQGAGSGGQQGPGSQGPGGAGQQGPYGPGATAAAAAARGYGPGAGQQGPGGAGQQGPGSQGPGGAGQQGPYGPGAAAAAAGGYGPGAGQQGPGGAGQQGPGSQGPGGAGQRGPYGPGAAAAAAAAGGYGPGAGQQGPGSGGQQGPSGQGPYGPGASAASAAAGGYGPGAGQQGPGGQGAGSGGQQGPGSQGPGGAGQQGPYGPGAAAAAAVPGGYGPGAGQQGPGGAGQQGSGSQGPGGAGQQGPFGPGAAAAAAAAAGGYGPGAGQQGPGSGGQQGPSGQGPYGPAASTAAAAAGGFGPGAGQQGPGGQGAGSGGQQGPGSQGPGGAGQQGPYGPGAAAAAAAAGGYGPGAGQQGPGGAGQQGPGSQGPGGAGQRGPYGPGAAAAAAAAGGYGPGAGQQGPGGAGQQGPGSQGPGSAGQQGPYGPGAAAATAAVGGYGPGAGQQGPRSQAPVASAAASRLSSPQASSRVSSAVSSLVSNGPTNPAALSNTIGSVVSQISASNPGLSNCDVLVQALLEMVSALVHILGSSSIGQINYGASSQYARMVGQSVAQALG

>Aarg_MaSp2.2c

MNWSIRLALFGFVVLSTQTVFSAAQAATPWQNSQLAEQFINSFLRFIAQSGAFSPNQLDDMSSIGDTLKTAIEKMAQSRKSSKSKLQALNMAFASSMAEIAVAEQGGLSLEAKTNAIASALASAFLETTGVVNQQFVSEIKGLIYMIAQASSNEISGSAAASGGGSGGGGGGGYGQGSYASASAAAAYGSAPQGAGGPASQGPSQQGPVSQPSYGPSATVAVSAVGGRPQGQTGPSQQGPGQQGPGQQGPGQQGPYGPSAAAASAAVGGYGPGAGQQGPGAQGPGQQGPGGQGPYGPGASAAAAAASGYGPGAGQQGPGSQGPSGPGQQGPGGQGPYGQGASAAAAAAGGYGPGAGQQGPGGQGSGGQQGPGSQGPGGAGQQGPYGPGAAAAAAAAGGYGPGAGQQGPGGQGAGSGGQQGPGSQGPGGAGQRGPYGPGAAAAAAAAGGYGPGAGQQGPGSGGQQGPSGQGPYGPGASAAAAAASGYGPGAGQQGPGSQGPSGPGQQGPGSQGPYGPGASAAAAAAGGYGPGAGQQGPGGQGSGGQQGPGSQGPGGAGQQGPYGPGAAAAAAAAGGYGPGAGQQGPGGQGAGSGGQQGPGSQGPGGAGQRGPYGPGAAAAAAAAGGYGPGAGQQGPGSGGQQGPSGQGPYGPGASAAAAAASGYGPGAGQQGPGSQGPSGPGQQGPGSQGPYGPGASAAAAAAGGYGPGAGQQGPGGQGSGGQQGPGSQGPGGAGQQGPYGPGAAAAAAAAGGYGPGAGQQGPGGQGAGSGGQQGPGSQGPGGAGQRGPYGPGAAAAAAAAGGYGPGAGQQGPGSGGQQGPSGQGPYGPGASAAAAAASGYGPGAGQQGPGSQGPSGPGQQGPGSQGPYGPGASAAAAAAGGYGPGAGQQGPGGQGSGGQQGPGSQGPGGAGQQGPYGPGAAAAAAAAGGYGPGAGQQGPGGQGAGSGGQQGPGSQGPGGAGQRGPYGPGAAAAAAAAGGYGPGAGQQGPGSGGQQGPSGQGPYGPGASAAAAAASGYGPGAGQQGPGSQGPSGPGQQGPGSQGPYGPGASAAAAAAGGYGPGAGQQGPGGQGSGGQQGPGSQGPGGAGQQGPYGPGAAAAAAAAGGYGPGAGQQGPGGQGAGSGGQQGPGSQGPGGAGQRGPYGPGAAAAAAAAGGYGPGAGQQGPGSGGQQGPSGQGPYGPGASAAAAAASGYGPGAGQQGPGSQGPSGPGQQGPGSQGPYGPGASAAAAAAGGYGPGAGQQGPGGQGSGGQQGPGSQGPGGAGQQGPYGPGAAAAAAAAGGYGPGAGQQGPGGQGAGSGGQQGPGSQGPGGAGQRGPYGPGAAAAAAAAGGYGPGAGQQGPGSGGQQGPSGQGPYGPGASAAAAAASGYGPGAGQQGPGSQGPSGPGQQGPGSQGPYGPGASAAAAAAGGYGPGAGQQGPGGQGSGGQQGPGSQGPGGAGQQGPYGPGAAAAAAAAGGYGPGAGQQGPGGQGAGSGGQQGPGSQGPGGAGQRGPYGPGAAAAAAAAGGYGPGAGQQGPGSGGQQGPSGQGPYGPGASAAAAAASGYGPGAGQQGPGSQGPSGPGQQGPGSQGPYGPGASAAAAAAGGYGPGAGQQGPGGQGSGGQQGPGSQGPGGAGQQGPYGPGAAAAAAAAGGYGPGAGQQGPGGQGAGSGGQQGPGSQGPGGAGQRGPYGPGAAAAAAAAGGYGPGAGQQGPGSGGQQGPSGQGPYGPGASAAAAAASGYGPGAGQQGPGSQGPSGPGQQGPGSQGPYGPGASAAAAAAGGYGPGAGQQGPGGQGSGGQQGPGSQGPGGAGQQGPYGPGAAAAAAAAGGYGPGAGQQGPGGQGAGSGGQQGPGSQGPGGAGQRGPYGPGAAAAAAAAGGYGPGAGQQGPGSGGQQGPSGQGPYGPGASAAAAAASGYGPGAGQQGPGSQGPSGPGQQGPGSQGPYGPGASAAAAAAGGYGPGAGQQGPGGQGSGGQQGPGSQGPGGAGQQGPYGPGAAAAAAAAGGYGPGAGQQGPGGQGAGSGGQQGPGSQGPGGAGQRGPYGPGAAAAAAAAGGYGPGAGQQGPGSGGQQGPSGQGPYGPGASAAAAAASGYGPGAGQQGPGSQGPSGPGQQGPGSQGPYGPGASAAAAAAGGYGPGAGQQGPGGQGSGGQQGPGSQGPGGAGQQGPYGPGAAAAAAAAGGYGPGAGQQGPGGQGAGSGGQQGPGSQGPGGAGQRGPYGPGAAAAAAAAGGYGPGAGQQGPGSGGXQGPSGQGPYGPGASAAAAAASGYGPGAGQQGPGSQGPSGPGQQGPGSQGPYGPGASAAAAAAGGYGPGAGQQGPGGQGSGGQQGPGSQGPGGAGQQGPYGPGAAAAAAAAGGYGPGAGQQGPGGQGAGSGGQQGPGSQGPGGAGQRGPYGPGAAAAAAAAGGYGPGAGQQGPGSGGQQGPSGQGPYGPGASAAAAAASGYGPGAGQQGPGSQGPSGPGQQGPGSQGPYGPGASAAAAAAGGYGPGAGQQGPGGQGSGGQQGPGSQGPGGAGQQGPYGPGAAAAAAAAGGYGPGAGQQGPGGQGAGSGGQQGPGSQGPGGAGQRGPYGPGAAAAAAAAGGYGPGAGQQGPGSGGQQGPSGQGPYGPGASAAAAAASGYGPGAGQQGPGSQGPTGPGQQGPGSQGPYGPGASAAAAAAGGYGPGAGQQGPGGQGSGGQQGPGSQGPGGAGQQGPYGPGAAAAAAAAGGYGPGAGQQGPGGQGAGSGGQQGPGSQGPGGAGQRGPYGPGAAAAAAAAGGYGPGAGQQGPGSGGQQGPSGQGPYGPGASAAAAAASGYGPGAGQQGPGSQGPSGPGQQGPGSQGPYGPGASAAAAAAGGYGPGAGQQGPGGQGSGGQQGPGSQGPGGAGQQGPYGPGAAAAAAAAGGYGPGAGQQGPGGQGAGSGGQQGPGSQGPGGAGQRGPYGPGAAAAAAAAGGYGPGAGQQGPGSGGQQGPSGQGPYGPGASAAAAAASGYGPGAGQQGPGSQGPSGPGQQGPGSQGPYGPGASAAAAAAGGYGPGAGQQGPGGQGSGGQQGPGSQGPGGAGQQGPYGPGAAAAAAAAGGYGPGAGQQGPGGQGAGSGGQQGPGSQGPGGAGQRGPYGPGAAAAAAAAGGYGPGAGQQGPGSGGQQGPSGQGPYGPGASAAAAAASGYGPGAGQQGPGSQGPSGPGQQGPGSQGPYGPGASAAAAAAGGYGPGAGQQGPGGQGSGGQQGPGSQGPGGAGQQGPYGPGAAAAAAAAGGYGPGAGQQGPGGQGAGSGGQQGPGSQGPGGAGQQGPYGPGAAAAAAAAGGYGPGAGQQGPGSQGPSGPSQQGPGGQGPYGPGASAAAAAASGYGPGSGQQGPSGPGQQGPGSQGPYGPGPSAAAAAAGGYGPGAGQQGPRSQAPVASAAASRLSSPQASSRVSSAVSSLVSSGPTNPAALSNTIGSVVSQVRSSNPGLSNCDVLVQALLEMVSALVHILGSSSIGQINYGASSQYAQLVGQSITQALA

>Aarg_MaSp2.2d

MNWSIRLALFGFVVLSTQTVFSAAQAATPWQNSQLAEQFINSFLRFIAQSGAFSPNQLDDMSSIGDTLKTAIEKMAQSRKSSKSKLQALNMAFASSMAEIAVAEQGGLSLEAKTNAIASALASAFLETTGVVNQQFVSEIKGLIYMIAQASSNEISGSAAASGGGSGGGGGGGYGQGSYASASAAAAYGSAPQGAGGPASQGPSQQGPVSQPSYGPSATVAVSAVGGRPQGQTGPSQQGPGQQGPGQQGPYGPSAAAASAAVGGYGPGAGQQGQQGPGAQGPGQQGPGGQGPYGPGASAAAAAASGYGPGAGQQGPGSQGPSGPGQQGPGGQGPYGPGASAAAAAAGGYGPGAGQQGPGGQGSGGQQGPGSQGPGGAGQQGPYGQGAAAAAAAAGGYGPGAGQQGPGSGGQQGPSGQGPYGPGASAAAAAASGYGPGAGQQGPGSQGPSGPGQQGPGSQGPYGPGASAAAAAAGGYGPGAGQQGPGGQGSGGQQGPGSQGPGGAGQQGPYGPGAAAAAAAAGGYGPGAGQQGPGGQGAGSGGQQGPGSQGPGGAGQRGPYGPGAAAAAAAAGGYGPGAGQQGPGSGGQQGPSGQGPYGPGASAAAAAASGYGPGAGQQGPGSQGPSGPGQQGPGSQGPYGPGASAAAAAAGGYGPGAGQQGPGGQGSGGQQGPGSQGPGGAGQQGPYGPGAAAAAAAAGGYGPGXGQQGPGGQGAGSGGQQGPGSQGPGGAGQRGPYGPGAAAAAAAAGGYGPGAGQQGPGSGGQQGPSGQGPYGPGASAAAAAASGYGPGAGQQGPGSQGPSGPGQQGPGSQGPYGPGASAAAAAAGGYGPGAGQQGPGGQGSGGQQGPGSQGPGGAGQQGPYGPGAAAAAAAAGGYGPGAGQQGPGGQGAGSGGQQGPGSQGPGGAGQRGPYGPGAAAAAAAAGGYGPGAGQQGPGSGGQQGPSGQGPYGPGASAAAAAASGYGPGAGQQGPGSQGPSGPGQQGPGSQGPYGPGASAAAAAAGGYGPGAGQQGPGGQGSGGQQGPGSQGPGGAGQQGPYGPGAAAAAAAAGGYGPGAGQQGPGGQGAGSGGQQGPGSQGPGGAGQRGPYGPGAAAAAAAAGGYGPGAGQQGPGSGGQQGPSGQGPYGPGASAAAAAASGYGPGAGQQGPGSQGPSGPGQQGPGSQGPYGPGASAAAAAAGGYGPGAGQQGPGGQGSGGQQGPGSQGPGGAGQQGPYGPGAAAAAAAAGGYGPGAGQQGPGGQGAGSGGQQGPGSQGPGGAGQRGPYGPGAAAAAAAAGGYGPGAGQQGPGSGGQQGPSGQGPYGPGASAAAAAASGYGPGAGQQGPGSQGPSGPGQQGPGSQGPYGPGASAAAAAAGGYGPGAGQQGPGGQGSGGQQGPGSQGPGGAGQQGPYGPGAAAAAAAAGGYGPGAGQQGPGGQGAGSGGQQGPGSQGPGGAGQRGPYGPGAAAAAAAAGGYGPGAGQQGPGSGGQQGPSGQGPYGPGASAAAAAASGYGPGAGQQGPGSQGPSGPGQQGPGSQGPYGPGASAAAAAAGGYGPGAGQQGPGGQGSGGQQGPGSQGPGGAGQQGPYGPGAAAAAAAAGGYGPGAGQQGPGGQGAGSGGQQGPGSQGPGGAGQRGPYGPGAAAAAAAAGGYGPGAGQQGPGSGGQQGPSGQGPYGPGASAAAAAASGYGPGAGQQGPGSQGPSGPGQQGPGSQGPYGPGASAAAAAAGGYGPGAGQQGPGGQGSGGQQGPGSQGPGGAGQQGPYGPGAAAAAAAAGGYGPGAGQQGPGGQGAGSGGQQGPGSQGPGGAGQRGPYGPGAAAAAAAAGGYGPGAGQQGPGSGGQQGPSGQGPYGPGASAAAAAASGYGPGAGQQGPGSQGPSGPGQQGPGSQGPYGPGASAAAAAAGGYGPGAGQQGPGGQGSGGQQGPGSQGPGGAGQQGPYGPGAAAAAAAAGGYGPGAGQQGPGGQGAGSGGQQGPGSQGPGGAGQRGPYGPGAAAAAAAAGGYGPGAGQQGPGSGGQQGPSGQGPYGPGASAAAAAASGYGPGAGQQGPGSQGPSGPGQQGPGSQGPYGPGASAAAAAAGGYGPGAGQQGPGGQGSGGQQGPGSQGPGGAGQQGPYGPGAAAAAAAAGGYGPGAGQQGPGGQGAGSGGQQGPGSQGPGGAGQRGPYGPGAAAAAAAAGGYGPGAGQQGPGSGGQQGPSGQGPYGPGASAAAAAASGYGPGAGQQGPGSQGPSGPGQQGPGSQGPYGPGASAAAAAAGGYGPGAGQQGPGGQGSGGQQGPGSQGPGGAGQQGPYGPGAAAAAAAAGGYGPGAGQQGPGGQGAGSGGQQGPGSQGPGGAGQRGPYGPGAAAAAAAAGGYGPGAGQQGPGSGGQQGPSGQGPYGPGASAAAAAASGYGPGAGQQGPGSQGPSGPGQQGPGSQGPYGPGASAAAAAAGGYGPGAGQQGPGGQGSGGQQGPGSQGPGGAGQQGPYGPGAAAAAAAAGGYGPGAGQQGPGGQGAGSGGQQGPGSQGPGGAGQRGPYGPGAAAAAAAAGGYGPGAGQQGPGSGGQQGPSGQGPYGPGASAAAAAASGYGPGAGQQGPGSQGPSGPGQQGPGSQGPYGPGASAAAAAAGGYGPGAGQQGPGGQGSGGQQGPGSQGPGGAGQQGPYGPGAAAAAAAAGGYGPGAGQQGPGGQGAGSGGQQGPGSQGPGGAGQRGPYGPGAAAAAAAAGGYGPGAGQQGPGSGGQQGPSGQGPYGPGASAAAAAASGYGPGAGQQGPGSQGPSGPGQQGPGSQGPYGPGASAAAAAAGGYGPGAGQQGPGGQGSGGQQGPGSQGPGGAGQQGPYGPGAAAAAAAAGGYGPGAGQQGPGGQGAGSGGQQGPGSQGPGGAGQRGPYGPGAAAAAAAAGGYGPGAGQQGPGSGGQQGPSGQGPYGPGASAAAAAASGYGPGAGQQGPGSQGPSGPGQQGPGSQGPYGPGASAAAAAAGGYGPGAGQQGPGGQGSGGQQGPGSQGPGGAGQQGPYGPGAAAAAAAAGGYGPGAGQQGPGGQGAGSGGQQGPGSQGPGGAGQRGPYGPGAAAAAAAAGGYGPGAGQQGPGSGGQQGPSGQGPYGPGASAAAAAASGYGPGAGQQGPGSQGPSGPGQQGPGSQGPYGPGASAAAAAAGGYGPGAGQQGPGGQGSGGQQGPGSQGPGGAGQQGPYGPGAAAAAAAAGGYGPGAGQQGPGGQGAGSGGQQGPGSQGPGGAGQRGPYGPGAAAAAAAAGGYGPGAGQQGPGSGGQQGPSGQGPYGPGASAAAAAASGYGPGAGQQGPGSQGPSGPGQQGPGSQGPYGPGASAAAAAAGGYGPGAGQQGPGGQGSGGQQGPGSQGPGGAGQQGPYGPGAAAAAAAAGGYGPGAGQQGPGGQGAGSGGQQGPGSQGPGGAGQRGPYGPGAAAAAAAAGGYGPGAGQQGPGSGGQQGPSGQGPYGPGASAAAAAASGYGPGAGQQGPGSQGPSGPGQQGPGSQGPYGPGASAAAAAAGGYGPGAGQQGPGGQGSGGQQGPGSQGPGGAGQQGPYGPGAAAAAAAAGGYGPGAGQQGPGGQGAGSGGQQGPGSQGPGGAGQQGPYGPGAAAAAAAAGGYGPGAGQQGPGSQGPSGPSQQGPGGQGPYGPGASAAAAAASGYGPGSGQQGPSGPGQQGPGSQGPYGPGPSAAAAAAGGYGPGAGQQGPRSQAPVASAAASRLSSPQASSRVSSAVSSLVSSGPTNPAALSNTIGSVVSQVRSSNPGLSNCDVLVQALLEMVSALVHILGSSSIGQINYGASSQYAQLVGQSITQALA

>Aaur_MaSp2.2a

MNWSIRLALLGFVVLSTQTIFAAGQAATPWENTQLAEDFIISFLRFIGQSGAFSPDQLDDMSTIGETLKTAIEKMAQSRKSSKSKLQALNMAFASSMAEIAVAEKGGLSLEAKTNAIANALASAFLETTGFVNQQFVSEIKSLIYMIAQASANEISGSAAAAGGGSGGFGSGQGGYGQGAYASASAASAYGSAPQGAGGPAPQGLSQQGPVRQGPYGPSAAVAATAVGGRPQGRSASSQQGPSQQGPYGPGAAGAAAAAGGYGPGVGQQGPGDAGQQGPYGPGAAAVGGYGPGARAGGPQRPIGAGPSLPSARGPQGPGGSGPGSQGPFEPAAAAAAAAAARGFGPGASGQKGPGEAGQQGPGGAGQQGPGGQGLFGPGAAAAAAAAAGGFGPGAGGQRGPGQQGPGGQGPSGPGAAAAAAAAAGGFGPGGAGAGPQAGQRGPGGAGAGAAAAAAAGAGGFGPGAGGQQGPGGAGPYGPSAGGQRGPGGVGQQGPGGQGPFGPGAAAAAAAAAGGFGPGGAGVGPQAAPGQQGPGGAGPYGPGAAAAAAAAGGFGPGAGGQRGPGQQGLFGPGAAAAAAAAAGGFGPGAGGQKVPGGAGQQGPGGQGPYGPGAAAAAAAAGGFGPGAGGQRGPGQQGPGGQGPSGPGAAAAAAAAAAGGFGPGGAGAGPQAGQRGPGGAGAGAAAAAAAGAGGFGPGAGGQQGPGGAGPYGPSAGGQRGPGGVGQQGPGGQGPFGPGAAAAAAAAAGGFGPGGAGVGPQAAPGQQGPGGAGPYGPGAAAAAAAAAAGGFGPGAGGQRGPGQQGLFGPGAAAAAAAAAGGFGPGAGGQKVPGGAGQQGPGGQGPYGPGAAAAAAAAGGFGPGAGGQRGPGQQGPGGQGPSGPGAAAAAAAAAAGGFGPGGAGAGPQAGQRGPGGAGAGAAAAAAAGAGGFGPGAGGQQGPGGAGPYGPSAGGQRGPGGVGQQGPGGQGPFGPGAAAAAAAAAGGFGPGGAGVGPQAAPGQQGPGGAGPYGPGAAAAAAAAAAGGFGPGAGGQRGPGQQGLFGPGAAAAAAAAAGGFGPGAGGQKVPGGAGQQGPGGQGPYGPGAAAAAAAAGGFGPGAGGQRGPGQQGPGGQGPSGPGAAAAAAAAAAGGFGPGGAGAGPQAGQRGPGGAGAGAAAAAAAGAGGFGPGAGGQQGPGGAGPYGPSAGGQRGPGGVGQQGPGGQGPFGPGAAAAAAAAAGGFGPGGAGVGPQAAPGQQGPGGAGPYGPGAAAAAAAAAAGGFGPGAGGQRGPGQQGLFGPGAAAAAAAAAGGFGPGAGGXKVPGGAGQQGPGGQGPYGPGAAAAAAAAGGFGPGAGGQRGPGQQGPGGQGPSGPGAAAAAAAAAAGGFGPGGAGAGPQAGQRGPGGAGAGAAAAAAAGAGGFGPGAGGQQGPGGAGPYGPSAGGQRGPGGVGQQGPGGQGPFGPGAAAAAAAAAGGFGPGGAGVGPQAAPGQQGPGGAGPYGPGAAAAAAAAGGFGPGAGGQRGPGQQGLFGPGAAAAAAAAAGGFGPGAGGXKVPGGAGQQGPGGQGPYGPGAAAAAAAAGGFGPGAGGQRGPGQQGPGGQGPSGPGAAAAAAAAAAGGFGPGGAGAGPQAGQRGPGGAGAGAAAAAAAGAGGFGPGAGGQQGPGGAGPYGPSAGGQRGPGGVGQQGPGGQGPFGPGAAAAAAAAAGGFGPGGAGVGPQAAPGQQGPGGAGPYGPGAAAAAAAAAAGGFGPGAGGQRGPGQQGLFGPGAAAAAAAAAGGFGPGAGGXKVPGGAGQQGPGGQGPYGPGAAAAAAAAGGFGPGAGGQRGPGQQGPGGQGPSGPGAAAAAAAAAAGGFGPGGAGAGPQAGQRGPGGAGAGAAAAAAAGAGGFGPGAGGQQGPGGAGPYGPSAGGQRGPGGVGQQGPGGQGPFGPGAAAAAAAAAGGFGPGGAGVGPQAAPGQQGPGGAGPYGPGAAAAAAAAAAGGFGPGAGGQRGPGQQGLFGPGAAAAAAAAAGGFGPGAGGQKVPGGAGQQGPGGQGPYGPGAAAAAAAAGGFGPGAGGQRGPGQQGPGGQGPSGPGAAAAAAAAAAGGFGPGGAGAGPQAGQRGPGGAGAGAAAAAAAGAGGFGPGAGGQQGPGGAGPYGPSAGGQRGPGGVGQQGPGGQGPFGPGAAAAAAAAAGGFGPGGAGVGPQAAPGQQGPGGAGPYGPGAAAAAAAAAAGGFGPGAGGQRGPGQQGLFGPGAAAAAAAAAGGFGPGAGGQKVPGGAGQQGPGGQGPYGPGAAAAAAAAGGFGPGAGGQRGPGQQGPGGQGPSGPGAAAAAAAAAAGGFGPGGAGAGPQAGQRGPGGAGAGAAAAAAAGAGGFGPGAGGQQGPGGAGPYGPSAGGQRGPGGVGQQGPGGQGPFGPGAAAAAAAAAGGFGPGGAGVGPQAAPGQQGPGGAGPYGPGAAAAAAAAAAGGFGPGAGGQRGPGQQGLFGPGAAAAAAAAAGGFGPGAGGQKVPGGAGQQGPGGQGPYGPGAAAAAAAAGGFGPGAGGQRGPGQQGPGGQGPSGPGAAAAAAAAAAGGFGPGGAGAGPQAGQRGPGGAGAGAAAAAAAGAGGFGPGAGGQQGPGGAGPYGPSAGGQRGPGGVGQQGPGGQGPFGPGAAAAAAAAAGGFGPGGAGVGPQAAPGQQGPGGAGPYGPGAAAAAAAAAAGGFGPGAGGQRGPGQQGLFGPGAAAAAAAAAGGFGPGAGGQKVPGGAGQQGPGGQGPYGPGAAAAAAAAGGFGPGAGGQRGPGQQGPGGQGPSGPGAAAAAAAAAAGGFGPGGAGAGPQAGQRGPGGAGAGAAAAAAAGAGGFGPGAGGQQGPGGAGPYGPSAGGQRGPGGVGQQGPGGQGPFGPGAAAAAAAAAGGFGPGGAGVGPQAAPGQQGPGGAGPYGPGAAAAAAAAAAGGFGPGAGGQRGPGQQGLFGPGAAAAAAAAAGGFGPGAGGQKVPGGAGQQGPGGQGPYGPGAAAAAAAAGGFGPGAGGQRGPGQQGPGGQGPSGPGAAAAAAAAAAGGFGPGGAGAGPQAGQRGPGGAGAGAAAAAAAGAGGFGPGAGGQQGPGGAGPYGPSAGGQRGPGGVGQQGPGGQGPFGPGAAAAAAAAAGGFGPGGAGVGPQAAPGQQGPGGAGPYGPGAAAAAAAAAAGGFGPGAGGQRGPGQQGLFGPGAAAAAAAAAGGFGPGAGGQKVPGGAGQQGPGGQGPYGPGAAAAAAAAGGFGPGAGGQRGPGQQGPGGQGPSGPGAAAAAAAAAAGGFGPGGAGAGPQAGQRGPGGAGAGAAAAAAAGAGGFGPGAGGQQGPGGAGPYGPSAGGQRGPGGVGQQGPGGQGPFGPGAAAAAAAAAGGFGPGGAGVGPQAAPGQQGPGGAGPYGPGAAAAAAAAAAGGFGPGAGGQRGPGQQGLFGPGAAAAAAAAAGGFGPGAGGQKVPGGAGQQGPGGQGPYGPGAAAAAAAAGGFGPGAGGQRGPGQQGPGGQGPSGPGAAAAAAAAAAGGFGPGGAGAGPQAGQRGPGGAGAGAAAAAAAGAGGFGPGAGGQQGPGGAGPYGPSAGGQRGPGGVGQQGPGGQGPFGPGAAAAAAAAAGGFGPGGAGVGPQAAPGQQGPGGAGPYGPGAAAAAAAAGGFGPGAGGQRGPGQQGPGGQGLFGPGAAAAAAAAAGGFGPGAGGQKGPGGAGQQGPGGQGPYGPGAAAAAAAAGGFGPGTGGQRGPGQQVPGGQGPSGPGAAAAAAAAAGGFGPGGAGPGPKAGQGGARFYRPGAAVATAAVGGYGPGAGQQGPAAPSQQGPGRQIPYGPGAAAAVVGVYAPVPQRPTASAAASRLASPEASSRVSSAVSSLVSSGPTNPAALSNTISSVVSQISASNPGLSGCDVLVQALLEIVSALVHILGYSSIGQINYGAASQYARLVGQSVAQALG

>Aaur_MaSp2.2b

MNWSIRLALLGFVVLSTQTVFSAGQGATPWENSQLAEDFINSFLRFIAQSGAFSPNQLDDMSSIGDTLKTAIEKMAQSRKSSKSKLQALNMAFASSMAEIAVAEQGGLSLEAKTNAIANALTSAFLETTGVVNQQFVSEIKSLIYMIAQASSNEISGSAAAAGGGSGGGGGSGQGGYGQGAYASASAAAAYGSAPQGAGGPAPQGPSQQGPVSQGPYGPGAAAAAAAAGGYGPGAGQQRQQGPGRQGKAGAGQQGPGGQGPYGPSAAAAAAAAGGYGQGAGQQGPGGAGQQGPGSQRPGGAGQQGPGGQGPYGPAAAAAAAAVGGYGPGAGQQGPGSQGPGSGGQQGPGSGGQQGPGGQGPYGSGQQGPGGAGQQGPGGQGPYGPGAAAAAAAAGGYGPGAGQQGPGGAGQQGPGSQGPGGAGQRGPGGQGPYGPGAAAAAAAAGGYGPGAGQQGPGSQGPGSGGQQGPGGQGPYGPSAAAAAAAAGGYGPGAGQQGRGSGGQQGPGSGGQQGPGGQGPYGSGQQGPGGAGQQGPGGQGPYGPGAAAAAAAAGGYGPGAGQQGPGGAGQQGPGSQGPGGAGQRGPGGQGPYGPGAAAAAAAAAGYGPGAGQQGPGSQGPGSGGQQGPGGQGPYGPSAAAAAAAAGGYGPGAGQQGRGSGGQQGPGSGGQQGPGGQGPYGSGQQGPGGAGQQGPGGQGPYGPGAAAAAAAAGGYGPGAGQQGPGGAGQQGPGGAGQQGPGSQGPGGAGQRGPGGQGPYGPGAAAAAAAAAGYGPGAGQQGPGSQGPGSGGQQGPGGQGPYGPSAAAAAAAAGGYGPGAGQQGRGSGGQQGPGSGGQQGPGGQGPYGSGQQGPGGAGQQGPGGQGPYGPGAAAAAAAAGGYGPGAGQQGPGGAGQQGPGGAGQQGPGGAGQQGPGSQGPGGAGQRGPGGQGPYGPGAAAAAAAAGGYGPGAGQQGPGSQGPGSGGQQGPGGQGPYGPSAAAAAAAAGGYGPGAGQQGRGSGGQQGPGSGGQQGPGGQGPYGSGQQGPGGAGQQGPGGQGPYGPGAAAAAAAAGGYGPGAGQQGPGGAGQQGPGSQGPGGAGQRGPGGQGPYGPGAAAAAAAAGGYGPGAGQQGPGGAGQQGPGGAGQQGPGSQGPGGAGQRGPGGQGPYGPGAAAAAAAAGGYGPGAGQQGPGSQGPGSGGQQGPGGQGPYGPSAAAAAAAAGGYGPGAGQQGPGGAGQQGPGSQGPGGAGQRGPGGQGPYGPGAAAAAAAAGGYGPGAGQQGPGSQGPGSGGQQGPGGQGPYGPSAAAAAAAAGGYGPGAGQQGPGSQGPGSGGQQGPGGQGPYGPSAAAAAAAAGGYGPGAGQQGPGSGGQQGPGGQGPYGSGQQGPGGAGQQGPGGQGPYGPGAAAAAAAAGGYGPGAGQQGPGGAGQQGPGGAGQQGPGSQGPGGAGQRGPGGQGPYGPGAAAAAAAAGGYGPGAGQQGPGSQGPGSGGQQGPGGQGPYGPSAAAAAAAAGGYGPGAGQQGPGGAGQQGPGSQGPGGAGQRGPGGQGPYGPGAAAAAAAAGGYGPGAGQQGPGSQGPGSGGQQGPGGQGPYGPSAAAAAAAAGGYGPGAGQQGPGSQGPGSGGQQGPGGQGPYGPSAAAAAAAAGGYGPGAGQQGPGSGGQQGPGGQGPYGSGQQGPGGAGQQGPGGQGPYGPGAAAAAAAAGGYGPGAGQQGPGGAGQQGPGSQGPGGAGQRGPGGQGPYGPGAAAAAAAAGGYGPGAGQQGPGSQGPGSGGQQGPGGQGTYGPSAAAAAAAAGGYGPGAGQQGPGGAGQQGPGSQGPGGAGQRGPGGQGPYGPGAAAAAAAAGGYGPGAGQQGPGSQGPGSGGQQGPGGQGPYGPSAAAAAAAAGGYGPGAGQQGPGSGGQQGPGGQGPYGSGQQGPGGAGQQGPGGQGPYGPGAAAAAAAAGGYGPGAGQQGPGGAGQQGPGSQGPGGAGQRGPGGQGPYGPGAAAAAAAAGGYGPGAGQQGPGSQGPGSGGQQGPGGQGPYGPSAAAAAAAAGGYGPGAGQQGPGGAGQQGPGSQGPGGAGQRGPGGQGPYGPGAAAAAAAAGGYGPGAGQQGPGSQGPGSGGQQGPGGQGPYGPSAAAAAAAAGGYGPGAGQQGPGSQGPGSGGQQGPGGQGPYGPSAAAAAAAAGGYGPGAGQQGPGSGGQQGPGGQGPYGSGQQGPGGAGQQGPGGQGPYGPGAAAAAAAAGGYGPGAGQQGPGGAGQQGPGSQGPGGAGQRGPGGQGPYGPGAAAAAAAAGGYGPGAGQQGPGSQGPGSGGQQGPGGQGPYGPSAAAAAAAAGGYGPGAGQQGPGGAGQQGPGSQGPGGAGQRGPGGQGPYGPGAAAAAAAAGGYGPGAGQQGPGSQGPGSGGQQGPGGQGPYGPSAAAAAAAAGGYGPGAGQQGPGSQGPGSGGQQGPGGQGPYGPSAAAAAAAAGGYGPGAGQQGPGSGGQQGPGGQGPYGSGQQGPGGAGQQGPGGQGPYGPGAAAAAAAAGGYGPGAGQQGPGGAGQQGPGSQGPGGAGQRGPGGQGPYGPGAAAAAAAAGGYGPGAGQQGPGSQGPGSGGQQGPGGQGPYGPSAAAAAAAAGGYGPGAGQQGPGGAGQQGPGSQGPGGAGQRGPGGQGPYGPGAAAAAAAAGGYGPGAGQQGPGSQGPGSGGQQGPGGQGPYGPSAAAAAAAAGGYGPGAGQQGPGSQGPGSGGQQGPGGQGPYGPSAAAAAAAAGGYGPGAGQQGPGSGGQQGPGGQGPYGSGQQGPGGAGQQGPGGQGPYGPGAAAAAAAAGGYGPGAGQQGPGGAGQQGPGSQGPGGAGQRGPGGQGPYGPGAAAAAAAAGGYGPGAGQQGPGSQGPGSGGQQGPGGQGPYGPSAAAAAAAAGGYGPGAGQQGPGGAGQQGPGSQGPGGAGQRGPGGQGPYGPGAAAAAAAAGGYGPGAGQQGPGSQGPGSGGQQGPGGQGPYGPSAAAAAAAAGGYGPGAGQQGPGSQGPGSGGQQGPGGQGPYGPSAAAAAAAAGGYGPGAGQQGPGSGGQQGPGGQGPYGSGQQGPGGAGQQGPGGQGPYGPGAAAAAAAAGGYGPGAGQQGPGGAGQQGPGSQGPGGAGQRGPGGQGPYGPGAAAAAAAAGGYGPGAGQQGPGSQGPGSGGQQGPGGQGPYGPSAAAAAAAAGGYGPGAGQQGPGGAGQQGPGSQGPGGAGQRGPGGQGPYGPGAAAAAAAAGGYGPGAGQQGPGSQGPGSGGQQGPGGQGPYGPSAAAAAAAAGGYGPGAGQQGPGSQGPGSGGQQGPGGQGPYGPSAAAAAAAAGGYGPGAGQQGPGSGGQQGPGGQGPYGSGQQGPGGAGQQGPGGQGPYGPGAAAAAAAAGGYGPGAGQQGPGGAGQQGPGSQGPGGAGQRGPGGQGPYGPGAAAAAAAAGGYGPGAGQQGPGSQGPGSGGQQGPGGQGPYGPSAAAAAAAAGGYGPGAGQQGPGGAGQQGPGSQGPGGAGQRGPGGQGPYGPGAAAAAAAAGGYGPGAGQQGPGSQGPGSGGQQGPGGQGPYGPSAAAAAAAAGGYGPGAGQQGPGSQGPGSGGQQGPGGQGPYGPSAAAAAAAAGGYGPGAGQQGPGSGGQQGPGGQGPYGSGQQGPGGAGQQGPGGQGPYGPGAAAAAAAAGGYGPGAGQQGPGGAGQQGPGGAGQQGPGSQGPGGAGQQGPGGQGPYGPGAAAAAAAAGGYGPGAGQQGPGSQGPGSGGQQGPGGQGPYGPSAAAAAAAAGGYGPGAGQQGPGGAGQQGLGSQGPGGAGQRGPGGQGPYGPGAAAAAAAAGGYGPGAGQQGPGSQGPVASAAASRLSSPQASSRVSSAVSTLVSSGPTNPAALSNAISNVVSQVSASNPGLSGCDVLVQALLEIVSALVHILGSSSIGQINYAASSQYAQMVGNSVTQALG

>Aaur_MaSp2.2c

MNWSIRLALLGFVVLSTQTVFXAGQGATPWENSQLAEDFINSFLRFIAQSGAFSPNQLDDMSSIGDTLKTAIEKMAQSRKSSKSKLQALNMAFASSMAEIAVAEQGGLSLEAKTNAIANALTSAFLETTGVVNQQFVSEIKSLIYMIAQASSNEISGSAAAAGGGSGGGGGSGQGGYGQGAYASASAAAAYGSAPQGAGGPAPQGPSQQGPVSQGPYGPGAAAAAAAAGGYGPGAGQQRQQGPGRQGKAGAGQQGPGGQGPYGPSAAAAAAAAGGYGPGAGQQGPGSQGPGAGQQGPGSQGPGSGGQQGPGGQGPYGPSAAAAAAAAGGYGSGAGQQGPGSQGPGSGGQQGPGGQGPYGPSAAAAAAAAGGYGPGAGQQGPGSGGQQGPGGQGPYGSGQQGPGGAGQQGPGGQGPYGPGAAAAAAAAGGYGPGAGQQGPGGAGQQGPGSQGPGGAGQRGPGGQGPYGPGAAAAAAAAGGYGPGAGQQGPGSQGPGSGGQQGPGGQGPYGPSAAAAAAAAGGYGPGAGQQGPGSGGQQGPGGQGPYGSGQQGPGGAGQQGPGGQGPYGPGAAAAAAAAGGYGPGAGQQGPGGAGQQGPGSQGPGGAGQRGPGGQGPYGPGAAAAAAAAGGYGPGAGQQGPGSQGPGSGGQQGPGGQGPYGPSAAAAAAAAGGYGPGAGQQGPGSQGPGSGGQQGPGGQGPYGPSAAAAAAAAGGYGPGAGQQGPGSGGQQGPGGQGPYGSGQQGPGGAGQQGPGGQGPYGPGAAAAAAAAGGYGPGAGQQGPGGAGQQGPGSQGPGGAGQRGPGGQGPYGPGAAAAAAAAGGYGPGAGQQGPGSGGQQGSGGQGPYGPSAAAAAAAAGGYGPRAGQQGPGSQGPGGAGQQGPGGQGPYGPGAAAAAAAAGGYGPGAGQQGPGSQGPGSGGQQGPGGQGPYGPSAAAAAAAAGGYGPGAGQQGPGSQGPGSGGQQGPGGQGPYGPSAAAAAAAAGGYGPGAGQQGPGSGGQQGPGGQGPYGSGQQGPGGAGQQGPGGQGPYGPGAAAAAAAAGGYGPGAGQQGPGGAGQQGPGSQGPGGAGQQGPGGQGPYGPGAAAAAAAAGGYGPGAGQQGPGSQGPGSGGQQGPGGQGPYGPSAAAAAAAAGGYGPGAGQQGPGSQGPGSGGQQGPGGQGPYGPSAAAAAAAAGGYGPGAGQQGPGSGGQQGPGGQGPYGSGQQGPGGAGQQGPGGQGPYGPGAAAAAAAAGGYGPGAGQQGPGGAGQQGPGSQGPGGAGQRGPGGQGPYGPGAAAAAAAAGGYGPGAGQQGPGSGGQQGSGGQGPYGPSAAAAAAAAGGYGPRAGQQGPGSQGPGGAGQQGPGGQGPYGPGAAAAAAAAGGYGPGAGQQGPGSQGPGSGGQQGPGGQGPYGPSAAAAAAAAGGYGPGAGQQGPGSQGPGSGGQQGPGGQGPYGPSAAAAAAAAGGYGPGAGQQGPGSGGQQGPGGQGPYGSGQQGPGGAGQQGPGGQGPYGPGAAAAAAAAGGYGPGAGQQGPGGAGQQGPGSQGPGGAGQQGPGGQGPYGPGAAAAAAAAGGYGPGAGQQGPGSQGPGSGGQQGPGGQGPYGPSAAAAAAAAGGYGPGAGQQGPGSQGPGSGGQQGPGGQGPYGPSAAAAAAAAGGYGPGAGQQGPGSGGQQGPGGQGPYGSGQQGPGGAGQQGPGGQGPYGPGAAAAAAAAGGYGPGAGQQGPGGAGQQGPGSQGPGGAGQRGPGGQGPYGPGAAAAAAAAGGYGPGAGQQGPGSGGQQGPGGQGPYGPSAAAAAAAAGGYGPRAGQQGPGSQGPGGAGQQGPGGQGPYGPGAAAAAAAAGGYGPGAGQQGPGSQGPGSGGQQGPGGQGPYGPSAAAAAAAAGGYGPGAGQQGPGSQGPGSGGQQGPGGQGPYGPSAAAAAAAAGGYGPGAGQQGPGSGGQQGPGGQGPYGSGQQGPGGAGQQGPGGQGPYGPGAAAAAAAAGGYGPGAGQQGPGGAGQQGPGSQGPGGAGQRGPGGQGPYGPGAAAAAAAAGGYGPGAGQQGPGSGGQQGSGGQGPYGPSAAAAAAAAGGYGPRAGQQGPGSQGPGGAGQQGPGGQGPYGPGAAAAAAAAGGYGPGAGQQGPGSQGPGSGGQQGPGGQGPYGPSAAAAAAAAGGYGPGAGQQGPGSQGPGSGGQQGPGGQGPYGPSAAAAAAAAGGYGPGAGQQGPGSGGQQGPGGQGPYGSGQQGPGGAGQQGPGGQGPYGPGAAAAAAAAGGYGPGAGQQGPGGAGQQGPGSQGPGGAGQQGPGGQGPYGPGAAAAAAAAGGYGPGAGQQGPGSQGPGSGGQQGPGGQGPYGPSAAAAAAAAGGYGPGAGQQGPGSQGPGSGGQQGPGGQGPYGPSAAAAAAAAGGYGPGAGQQGPGSGGQQGPGGQGPYGSGQQGPGGAGQQGPGGQGPYGPGAAAAAAAAGGYGPGAGQQGPGGAGQQGPGSQGPGGAGQRGPGGQGPYGPGAAAAAAAAGGYGPGAGQQGPGSGGQQGSGGQGPYGPSAAAAAAAAGGYGPRAGQQGPGSQGPGGAGQQGPGGQGPYGPGAAAAAAAAGGYGPGAGQQGPGSQGPGSGGQQGPGGQGPYGPSAAAAAAAAGGYGPGAGQQGPGSQGPGSGGQQGPGGQGPYGPSAAAAAAAAGGYGPGAGQQGPGSGGQQGPGGQGPYGSGQQGPGGAGQQGPGGQGPYGPGAAAAAAAAGGYGPGAGQQGPGGAGQQGPGSQGPGGAGQQGPGGQGPYGPGAAAAAAAAGGYGPGAGQQGPGSQGPGSGGQQGPGGQGPYGPSAAAAAAAAGGYGPGAGQQGPGSQGPGSGGQQGPGGQGPYGPSAAAAAAAAGGYGPGAGQQGPGSGGQQGPGGQGPYGSGQQGPGGAGQQGPGGQGPYGPGAAAAAAAAGGYGPGAGQQGPGGAGQQGPGSQGPGGAGQQGPGGQGPYGPGAAAAAAAAGGYGPGAGQQGPGSQGPGSGGQQGPGGQGPYGPSAAAAAAAAGGYGPGAGQQGPGSQGPGSGGQQGPGGQGPYGPSAAAAAAAAGGYGPGAGQQGPGSGGQQGPGGQGPYGSGQQGPGGAGQQGPGGQGPYGPGAAAAAAAAGGYGPGAGQQGPGGAGQQGPGSQGPGGAGQQGPGGQGPYGPGAAAAAAAAGGYGPGAGQQGPGSQGPGSGGQQGPGGQGPYGPSAAAAAAAAGGYGPGAGQQGPGSQGPGSGGQQGPGGQGPYGPSAAAAAAAAGGYGPGAGQQGPGSGGQQGPGGQGPYGSGQQGPGGAGQQGPGGQGPYGPGAAAAAAAAGGYGPGAGQQGPGGAGQQGPGSQGPGGAGQQGPGGQGPYGPGAAAAAAAAGGYGPGAGQQGPGSQGPGSGGQQGPGGQGPYGPSAAAAAAAAGGYGPGVGQQGPGSQGPGSGGQQGPGGQGPYGPSAAAAAAAAGGYGPGAGQQGPGSGGQQGPGGQGPYGSGQQGPGGAGQQGPGGQGPYGPSAAAAAAAAGGYGPGAGQQVLGSQGPVASAAASRLSSPQASSRVSSAVSTLVSSGPTNPAALSNAISNVVSQVSASNPGLSGCDVLVQALLEIVSALVHILGSSSIGQINYAASSQYAQMVGNSVTQALG

>Aaur_MaSp2.2d

MNWSIRLALLGFVVLSTQTVFSAGQGATPWENSQLAEEFINSFLRFIAQSGAFSPNQLDDMSSIGDTLKTAIEKMAQSRKSSKSKLQALNMAFASSMAEIAVAEQGGLSLESKTNAIANALASAFLETTGFVNQQFVSEIKSLIYMIAQASSNEISGSAAAAGGGSGGGGGSGQGGYGQGAYASASAAAAYGSAPQGAGGPAPQGPSQQGPVSQGPYGPGAAVAAAAAGGYGPGAGQQGQQGPGRQGNAGPGQQGPGGQGPYGPSAAAAAAAAGGYGPGAGQQGPGGAGQQGPGSQGPGGAGQQGPGGQGPYGPGAAAAAAAVGGYGPGAGQQGPGSQGPGSGGQQGPGGQGPYGPSAAAAAAAAGGYGPGAGQQGPGSQGPGSGGQQGPGGQGPYGPSAAAAAAAAGGYGPGAGQQGPGSGGQQGPGGQGPYGSGQQGPGGAGQQGPGGQGPYGPGAAAAAAAAGGYGPGAGQQGPGGAGQQGPGSQGPGGAGQQGPGGQGPYGPGAAAAAAAVGGYGPGAGQQGPGSQGPGRGGQQGPGGQGPYGPSAAAAAAAAGGYGPGAGQQGPGSQGPGSGGQQGPGGQGPYGPSAAAAAAAAGGYGPGAGQQGPGSQGPGSGGQQGPGGQGPYGPSAAAAAAAAGGYGPGAGQQGPGSGGQQGPGGQGPYGSGQQGPGGAGQQGPGGQGPYGPGAAAAAAAAGGYGPGAGQQGPGGAGQQGPGSQGPGGAGQQGPGGQGPYGPGAAAAAAAVGGYGPGAGQQGPGSQGPGSGGQQGPGGQGPYGPSAAAAAAAAGGYGPGAGQQGPGSQGPGSGGQQGPGGQGPYGPSAAAAAAAAGGYGPGAGQQGPGSGGQQGPGGQGPYGSGQQGPGGAGQQGPGGQGPYGPGAAAAAAAAGGYGPGAGQQGPGGAGQQGPGSQGPGGAGQQGPGGQGPYGPGAAAAAAAVGGYGPGAGQQGPGSQGPGSGGQQGPGGQGPYGPSAAAAAAAAGGYGPGAGQQGPGSQGPGSGGQQGPGGQGPYGPSAAAAAAAAGGYGPGAGQQGPGSGGQQGPGGQGPYGSGQQGPGGAGQQGPGGQGPYGPGAAAAAAAAGGYGPGAGQQGPGGAGQQGPGSQGPGGAGQQGPGGQGPYGPGAAAAAAAVGGYGPGAGQQGPGSQGPGSGGQQGPGGQGPYGPSAAAAAAAAGGYGPGAGQQGPGSQGPGSGGQQGPGGQGPYGPSAAAAAAAAGGYGPGAGQQGPGSGGQQGPGGQGPYGSGQQGPGGAGQQGPGGQGPYGPGAAAAAAAAGGYGPGAGQQGPGGAGQQGPGSQGPGGAGQQGPGGQGPYGPGAAAAAAAVGGYGPGAGQQGPGSQGPGSGGQQGPGGQGPYGPSAAAAAAAAGGYGPGAGQQGPGSQGPGSGGQQGPGGQGPYGPSAAAAAAAAGGYGPGAGQQGPGSGGQQGPGGQGPYGSGQQGPGGAGQQGPGGQGPYGPGAAAAAAAAGGYGPGAGQQGPGGAGQQGPGSQGPGGAGQQGPGGQGPYGPGAAAAAAAVGGYGPGAGQQGPGSQGPGSGGQQGPGGQGPYGPSAAAAAAAAGGYGPGAGQQGPGSQGPGSGGQQGPGGQGPYGPSAAAAAAAAGGYGPGAGQQGPGSGGQQGPGGQGPYGSGQQGPGGAGQQGPGGQGPYGPGAAAAAAAAGGYGPGAGQQGPGGAGQQGPGSQGPGGAGQQGPGGQGPYGPGAAAAAAAVGGYGPGAGQQGPGSQGPGSGGQQGPGGQGPYGPSAAAAAAAAGGYGPGAGQQGPGSQGPGSGGQQGPGGQGPYGPSAAAAAAAAGGYGPGAGQQGPGSGGQQGPGGQGPYGSGQQGPGGAGQQGPGGQGPYGPGAAAAAAAAGGYGPGAGQQGPGGAGQQGPGSQGPGGAGQQGPGGQGPYGPGAAAAAAAVGGYGPGAGQQGPGSQGPGSGGQQGPGGQGPYGPSAAAAAAAAGGYGPGAGQQGPGSQGPGSGGQQGPGGQGPYGPSAAAAAAAAGGYGPGAGQQGPGSQGPGSGGQQGPGGQGPYGPSAAAAAAAAGGYGPGAGQQGPGSGGQQGPGGQGPYGSGQQGPGGAGQQGPGGQGPYGPGAAAAAAAAGGYGPGAGQQGPGGAGQQGPGSQGPGGAGQQGPGGQGPYGPGAAAAAAAVGGYGPGAGQQGPGSQGPGSGGQQGPGGQGPYGPSAAAAAAAAGGYGPGAGQQGPGSQGPGSGGQQGPGGQGPYGPSAAAAAAAAGGYGPGAGQQGPGSQGPGSGGQQGPGGQGPYGPSAAAAAAAAGGYGPGAGQQGPGSGGQQGPGGQGPYGSGQQGPGGAGQQGPGGQGPYGPGAAAAAAAAGGYGPGAGQQGPGGAGQQGPGSQGPGGAGQQGPGGQGPYGPGAAAAAAAVGGYGPGAGQQGPGSQGPGSGGQQGPGGQGPYGPSAAAAAAAAGGYGPGAGQQGPGSQGPGSGGQQGPGGQGPYGPSAAAAAAAAGGYGPGAGQQGPGSGGQQGPGGQGPYGSGQQGPGGAGQQGPGGQGPYGPGAAAAAAAAGGYGPGAGQQGPGGAGQQGPGSQGPGGAGQQGPGGQGPYGPGAAAAAAAVGGYGPGAGQQGPGSQGPGSGGQQGPGGQGPYGPSAAAAAAAAGGYGPGAGQQGPGSQGPGSGGQQGPGGQGPYGPSAAAAAAAAGGYGPGAGQQGPGSGGQQGPGGQGPYGSGQQGPGGAGQQGPGGQGPYGPGAAAAAAAAGGYGPGAGQQGPGGAGQQGPGSQGPGGAGQQGPGGQGPYGPGAAAAAAAVGGYGPGAGQQGPGSQGPGSGGQQGPGGQGPYGPSAAAAAAAAGGYGPGAGQQGPGSQGPGSGGQQGPGGQGPYGPSAAAAAAAAGGYGPGAGQQGPGSGGQQGPGGQGPYGSGQQGPGGAGQQGPGGQGPYGPGAAAAAAAAGGYGPGAGQQGPGGAGQQGPGSQGPGGAGQQGPGGQGPYGPGAAAAAAAVGGYGPGAGQQGPGSQGPGSGGQQGPGGQGPYGPSAAAAAAAAGGYGPGAGQQGPGSQGPGSGGQQGPGGQGPYGPSAAAAAAAAGGYGPGAGQQGPGSGGQQGPGGQGPYGSGQQGPGGAGQQGPGGQGPYGPGAAAAAAAAGGYGPGAGQQGPGGAGQQGPGSQGPGGAGQQGPGGQGPYGPGAAAAAAAVGGYGPGAGQQGPGSQGPGSGGQQGPGGQGPYGPSAAAAAAAAGGYGPGAGQQGPGSQGPGSGGQQGPGGQGPYGPSAAAAAAAAGGYGPGAGQQGPGSQGPGSGGQQGPGGQGPYGPSAAAAAAAAGGYGPGAGQQGPGSGGQQGPGGQGPYGSGQQGPGGAGQQGPGGQGPYGPGAAAAAAAAGGYGPGAGQQGPGGAGQQGPGSQGPGGAGQQGPGGQGPYGPGAAAAAAAVGGYGPGAGQQGPGSQGPGSGGQQGPGGQGPYGPSAAAAAAAAGGYGPGAGQQGPGSQGPGSGGQQGPGGQGPYGPSAAAAAAAAGGYGPGAGQQGPGSQAPVASAAASRLSSPQASSRVSSAVSTLVSSGPTNPAALSNAISSVVSQVSASNPGLSGCDVLVQALLELVSALVHILGSSSIGQINYAASSQYAQMVGNSVTQALG

>Aaur_MaSp2.2e

MNWSIRLALLGLVVLSTQTVFSAGQGATPWENSQLAEEFINSFLRFIAQSGAFSPNQLDDMSSIGDTLKTAIEKMAQSRKSSKSKLQALNMAFASSMAEIAVAEQGGLSLEAKTNAIANALTSAFLETTGVVNQQFVSEIKGLIYMIAQASSNEISGSAAAAGGGSGGGSSGQGGYGQGAYASVSTATTYGSAPQGAGGPAPQGPSQQGPISQPSYGASATVTVTTVGGRQQGPTGPSQQGPGQQGPYGPSAAAASAAVSGYGPGGGQQEQQGPGGQGPGSAGQQGPGQQGPYGSSAAATAAAAGGYGPGAGQQGPGRAGQQGPGSQGPGGAGQQGPGGQGPYGPGADAAAAAVGGYGPGAGQQGPGSQGPGSGGQQGPGGQGPYGPSAAAAAAATAGYGPGAGQQGPGSQGPGGAGQQGPGGQGPYGPGSAAAAAAAGGYGPGVGQQGPGSQGPGSSGQQGPGGQGPYGPSAAAAAAAAGGYGPGAGQQGPGGAGQQGPGSQGPGGAGQQGPGGQGPYGPGAAASAAAVGGYGPGAGQQGPGSQGPGSGGQQGPGGQGPYAPSAAAAAAATGGYGPGAGQQGPGSQGPGSGGQQGPGSQGPYGPSAATAAAAAGGYGPGAGQQGPGSQGPGSGGQQGPGSQGPYGPSAAAAAAAAGGYGPGAGQQGPGSGGQQGSGGQGPYGSGQQGPGGAGQQGPGGQGPYGPGSAAAAAAAGGYGPGVGQQGPGSQGPGSSGQQGPGGQGPYGPSAAAAAAAAGGYGPGAGQQGPGGAGQQGPGSQGPGGAGQQGPGGQGPYGPGAAAAAAAVGGYGPGAGQQGPGSQGPGSGGQQGPGGQGPYGPSAAAAAAATGGYGPGAGQQGPGSQGPGSGGQQGPGGQGAYGSSAAAAAAAAGGYGPGAGQQGPGSGGQQGSGGQGPYGSGQQGPGGAGQQGPGGQGPYGPGSAAAAAAAGGYGPGVGQQGPGSQGPGSSGQQGPGGQGPYGPSAAAAAAAAGGYGPGAGQQGPGGAGQQGPGSQGPGGAGQQGPGGQGPYGPGAAAAAAAVGGYGPGAGQQGPGSQGPGSGGQQGPGGQGPYGPSAAAAAAATGGYGPGAGQQGPGSQGPGSGGQQGPGGQGPYGSSAAAAAAAAGGYGPGAGQQGPGSGGQQGSGGQGPYGSGQQGPGGAGQQGPGGQGPYGPGSAAAAAAAGGYGPGVGQQGPGSQGPGSSGQQGPGGQGPYGPSAAAAAAAAGGYGPGAGQQGPGGAGQQGPRSQGPGGAGQQGPGGQGPYGPGAAAAAAAVGGYGPGAGQQGPGSQGPGSGGQQGPGGQGPYGPSAAAAAAATGGYGPGAGQQGPGSQGPGSGGQQGPGGQGAYGSSAAAAAAAAGGYGPGAGQQGPGSGGQQGSGGQGPYGSGQQGPGGAGQQGPGGQGPYGPGSAAAAAAAGGYGPGVGQQGPGSQGPGSSGQQGPGGQGPYGPSAAAAAAAAGGYGPGAGQQGPGGAGQQGPGSQGPGGAGQQGPGGQGPYGPGAAAAAAAVGGYGPGAGQQGPGSQGPGSGGQQGPGGQGPYGPSAAAAAAATGGYGPGAGQQGPGSQGPGSGGQQGPGGQGAYGSSAAAAAAAAGGYGPGAGQQGPGSGGQQGSGGQGPYGSGQQGPGGAGQQGPGGQGPYGPGSAAAAAAAGGYGPGVGQQGPGSQGPGSSGQQGPGGQGPYGPSAAAAAAAAGGYGPGAGQQGPGGAGQQGPGSQGPGGAGQQGPGGQGPYGPGAAAAAAAVGGYGPGAGQQGPGSQGPGSGGQQGPGGQGPYGPSAAAAAAATGGYGPGAGQQGPGSQGPGSGGQQGPGGQGAYGSSAAAAAAAAGGYGPGAGQQGPGSGGQQGSGGQGPYGSGQQGPGGAGQQGPGGQGPYGPGSAAAAAAAGGYGPGVGQQGPGSQGPGSSGQQGPGGQGPYGPSAAAAAAAAGGYGPGAGQQGPGGAGQQGPGSQGPGGAGQQGPGGQGPYGPGAAAAAAAVGGYGPGAGQQGPGSQGPGSGGQQGPGGQGPYGPSAAAAAAATGGYGPGAEQQGPGSQGPGSGGQQGPGGQGAYGSSAAAAAAAAGGYGPGAGQQGPGSGGQQGSGGQGPYGSGQQGPGGAGQQGPGGQGPYGPGSAAAAAAAGGYGPGVGQQGPGSQGPGSSGQQGPGGQGPYGPSAAAAAAAAGGYGPGAGQQGPGGAGQQGPGSQGPGGAGQQGPGGQGPYGPGAAAAAAAVGGYGPGAGQQGPGSQGPGSGGQQGPGGQGPYGPSAAAAAAATGGYGPGAEQQGPGSQGPGSGGQQGPGGQGAYGSSAAAAAAAAGGYGPGAGQQGPGGQGPYGSGQQGPGGAGQQGPGGQGPYGPGSAAAAAAAGGYGPGDGQQGPGGAGQQGPGSQGPGGAGQQRPGGQGPYGPGAAAAAAAVGGYGPGAGQQGPGSQGPGSGGQLGPGSQGLYGPSSAAAAAAVGGYGPGAGQQGPGSQGPGSGGQQGPYGPSSSTAAASAGGYGPGTVQQGPRSQAPVASAAASRLSSPQASSRVSSAVSTLVSSGPTNPAALSNAISSVVSQVSASNPGLSGCDVLVQALLEIVSALVHILGSSSIGQINYAASSQYTQMVGNSVAQALG

>Atri_MaSp2.2a

MNWSIRLALLGFVVLSTQTVFSAGQGATPWENSQLAEQFINSFLRFIGQSGAFSPNQLDDMSSIGDTLKTAIEKMAQSRKSSKSKLQALNMAFASSMAEIAVAEQGGLSLEAKTNAIENALISAFLETTGVVNQQFVSEIKSLIYMIAQASSNEISGSAAAAGGGSGGGGSGQGGYGQGSYASASAAAAYGSAPQGTGGPAPQGPSQQGPVSQPSYGPSAAVAVTAVGGRQQGPSAPSQQGPSQQGPGQQGSGGQGPYGPSAAAAAAAAGGYGPGAGQQGQQGGQGPSGSGQQGPGSAGQRGPGGQGPYGPGAAAAAAAAAGGYGPGAGQQGPGSQGPGSGGQQGPGSQGPYGPSAAAAAAAAGPGYGPGAGQQGPGSQGPGSGGQQGPGGQGPYGPGAAAAAAAAGGYGPGAGQQGPGSGGQQGGQGSGQQGPGGAGQGGPGGQGPYGPGAAAAAAAGGYGPGAGQQGPGSQGPGSGGQQGPGAQGPYGPGAAAAAAAAGPGYGPGAGQQGPGSQGPGSGGQQGPGGQGPYGPSAAAAAAAAGGYGPGAGRQGPGSGGQQGGQGSGQQGPGGAGQGGPGGQGPYGPGAAAAAAAAAGGYGPGAGQQGPGSQGPGSGGQQGPGAQGPYGPSAAAAAAAAGPGYGPGAGQQGPGSQGPGSGGQQGPGGQGPYGPSAAAAAAAAGGYGPGAGRQGPGSGGQQGGQGSGQQGPGGAGQGGPGGQGPYGPGAAAAAAAAAGGYGPGAGQQGPGSQGPGSGGQQGPGAQGPYGPSAAAAAAAAGPGYGPGAGQQGPGSQGPGSGGQQGPGGQGPYGPSAAAAAAAAGGYGPGAGRQGPGSGGQQGGQGSGQQGPGGAGQGGPGGQGPYGPGAAAAAAAAAGGYGPGAGQQGPGSQGPGSGGQQGPGAQGPYGPSAAAAAAAAGPGYGPGAGQQGPGSQGPGSGGQQGPGGQGPYGPSAAAAAAAAGGYGPGAGRQGPGSGGQQGGQGSGQQGPGGAGQGGPGGQGPYGPGAAAAAAAAAGGYGPGAGQQGPGSQGPGSGGQQGPGAQGPYGPSAAAAAAAAGPGYGPGAGQQGPGSQGPGSGGQQGPGGQGPYGPSAAAAAAAAGGYGPGAGRQGPGSGGQQGGQGSGQQGPGGAGQGGPGGQGPYGPGAAAAAAAAAGGYGPGAGQQGPGSQGPGSGGQQGPGAQGPYGPSAAAAAAAAGPGYGPGAGQQGPGSQGPGSGGQQGPGGQGPYGPSAAAAAAAAGGYGPGAGRQGPGSGGQQGGQGSGQQGPGGAGQGGPGGQGPYGPGAAAAAAAAAGGYGPGAGQQGPGSQGPGSGGQQGPGAQGPYGPSAAAAAAAAGPGYGPGAGQQGPGSQGPGSGGQQGPGGQGPYGPSAAAAAAAAGGYGPGAGRQGPGSGGQQGGQGSGQQGPGGAGQGGPGGQGPYGPGAAAAAAAAAGGYGPGAGQQGPGSQGPGSGGQQGPGAQGPYGPSAAAAAAAAGPGYGPGAGQQGPGSQGPGSGGQQGPGGQGPYGPSAAAAAAAAGGYGPGAGRQGPGSGGQQGGQGSGQQGPGGAGQGGPGGQGPYGPGAAAAAAAAAGGYGPGAGQQGPGSQGPGSGGQQGPGAQGPYGPSAAAAAAAAGPGYGPGAGQQGPGSQGPGSGGQQGPGGQGPYGPSAAAAAAAAGGYGPGAGRQGPGSGGQQGGQGSGQQGPGGAGQGGPGGQGPYGPGAAAAAAAAAGGYGPGAGQQGPGSQGPGSGGQQGPGAQGPYGPSAAAAAAAAGPGYGPGAGQQGPGSQGPGSGGQQGPGGQGPYGPSAAAAAAAAGGYGPGAGRQGPGSGGQQGGQGSGQQGPGGAGQGGPGGQGPYGPGAAAAAAAAAGGYGPGAGQQGPGSQGPGSGGQQGPGAQGPYGPSAAAAAAAAGPGYGPGAGQQGPGSQGPGSGGQQGPGGQGPYGPSAAAAAAAAGGYGPGAGRQGPGSGGQQGGQGSGQQGPGGAGQGGPGGQGPYGPGAAAAAAAAAGGYGPGAGQQGPGSQGPGSGGQQGPGAQGPYGPSAAAAAAAAGPGYGPGAGQQGPGSQGPGSGGQQGPGGQGPYGPSAAAAAAAAGGYGPGAGRQGPGSGGQQGGQGSGQQGPGGAGQGGPGGQGPYGPGAAAAAAAAAGGYGPGAGQQGPGSQGPGSGGQQGPGAQGPYGPSAAAAAAAAGPGYGPGAGQQGPGSQGPGSGGQQGPGGQGPYGPSAAAAAAAAGGYGPGAGRQGPGSGGQQGGQGSGQQGPGGAGQGGPGGQGPYGPGAAAAAAAAAGGYGPGAGQQGPGSQGPGSGGQQGPGAQGPYGPSAAAAAAAAGPGYGPGAGQQGPGSQGPGSGGQQGPGGQGPYGPSAAAAAAAAGGYGPGAGRQGPGSGGQQGGQGSGQQGPGGAGQGGPGGQGPYGPGAAAAAAAAAGGYGPGAGQQGPGSQGPGSGGQQGPGAQGPYGPSAAAAAAAAGPGYGPGAGQQGPGSQGPGSGGQQGPGGQGPYGPSAAAAAAAAGGYGPGAGRQGPGSGGQQGGQGSGQQGPGGAGQGGPGGQGPYGPGAAAAAAAGGYGPGAGQQGPGSQGPGSGGQQGPGGQGPYGPSAAAAAAAAGGYGPGAGRQGPGSGGQQGGQGSGQQGPGGAGQGGPGGQGPYGPGAAAAAAAAAGGYGPGAGQQGPGSQGPGSGGQQGPGAQGPYGPSAAAAAAAAGPGYGPGAGQQGPGSQGPGSGGQQGPGGQGPYGPSAAAAAAAAGGYGPGAGRQGPGSGGQQGGQGSGQQGPGGAGQGGPGGQGPYGPGAAAAAAAAAGGYGPGAGQQGPGSQGPGSGGQQGPGAQGPYGPSAAAAAAAAGPGYGPGAGQQGPGSQGPGSGGQQGPGGQGPYGPSAAAAAAAAGGYGPGAGRQGPGSGGQQGGQGSGQQGPGGAGQGGPGGQGPYGPGAAAAAAAAAGGYGPGAGQQGPGSQGPGSGGQQGPGAQGPYGPSAAAAAAAAGPGYGPGAGQQGPGSQGPGSGGQQGPGGQGPYGPSAAAAAAAAGGYGPGAGRQGPGSGGQQGGQGSGQQGPGGAGQGGPGGQGPYGPGAAAAAAAAGGYGPGAGQQGPGSQGPGSGGQQGPGAQGPYGPSAAAAAAAAGPGYGPGAGQQGPGSQGPGSGGQQGPGGQGPYGPSAAAAAAAAGGYGPGAGRQGPGSGGQQGGQGSGQQGPGGAGQGGPGGQGPYGPGAAAAAAAAAGGYGPGAGQQGPGSQGPGSGGQQGPGAQGPYGPSAAAAAAAAGPGYGPGAGQQGPGSQGPGSGGQQGPGGQGPYGPSAAAAAAAAGGYGPGAGRQGPGSGGQQGGQGSGQQGPGGAGQGGPGGQGPYGPGAAAAAAAAGGYGPGAGQQGPGSQGPGSGGQQGPGAQGPYGPSAAAAAAAAGPGYGPGAGQQGPGSQGPGSGGQQGPGGQGPYGPSAAAAAAAAGGYGPGAGRQGPGSGGQQGGQGSGQQGPGGAGQGGPGGQGPYGPGAAAAAAAAAGGYGPGAGQQGPGSQGPGSGGQQGPGSQGPYGPSAAAAAAAAGPGYGPGAGQQGPGSQGPGSGGQQGPGSQGPYGPSAAAAAAAAGPGYGPGAGRQGPGSQAPVASAAASRLSSPQASSRVSSAVSTLVSSGPTNPASLSNAISSVVSQVSASNPGLSGCDVLVQALLEIVSALVHILGSSSIGQINYAASSQYAQMVGQSLTQALG

>Atri_MaSp2.2b

MNWSIRLALLGFVVLSTQTVFSAGQGATPWENSQLAESFISSFLRFIGQSGAFSPNQLDDMSSIGDTLKTAIEKMAQSRKSSKSKLQALNMAFASSMAEIAVAEQGGLSLEAKTNAIASALSAAFLETTGYVNQQFVNEIKTLIFMIAQASSNEISGSAAAAGGSSGGGGGSGQGGYGQGAYASASAAAAYGSAPQGTGGPASQGPSQQGPVSQPSYGPSATVAVTAVGGRPQGPSAPRQQGPSQQGPGQQGPGGRGSYGPSAAAAAAAAGGYGPGAGQQGQQGQGSGQQGPGGAGQGGPRGQGPYGPGAATAAAAAAGPGYGPGAGQQGPGSQGPGSSGQQGPGSQGPYGLSAAAAAAAAGPGYGPGAGQQGPGSQGPGSGGQQGPGGRGPYGPSAAAAAAAAGPGYGPGAGQQGPGSGGQQGGQGSGQQGPGGAGQGGPRGQGPYGPGAAAAAAAAAGGYGPGAGQQGPGSQGPGSGGQQGPGSQGPYGPSAAAAAAAAGPGYGPGAGQQGPGSXGPGSGGQQGPGGQGPYGPSAAAAAAAAGPGYGPGAGQQGPGSGGQQGGQGSGQQGPGGAGQGGPRGQGPYGPGAAAAAAAAAGGYGPGAGQQGPGSQGPGSGGQQGPGSXGPYGPSAAAAAAAAGPGYGPGAGQQGPGSQGPGSGGQQGPGGQGPYGPSAAAAAAAAGPGYGPGAGQQGPGSGGQQGGQGSGQQGPGGAGQGGPRGQGPYGPGAAAAAAAAGGYGPGAGQQGPGSQGPGSGGQQGPGSQGPYGPSAAAAAAAAGPGYGPGAGQQGPGSQGPGSGGQQGPGGQGPYGPSAAAAAAAAGPGYGPGAGQQGPGSGGQQGGQGSGQQGPGGAGQGGPRGQGPYXPGAAAAAAAAAGGYGPXAGQQGPGSQGPGSGGQQGPGSQRPYGPSAAAAAAAAGPGYGPGAGQQGPGSQGPGSGGQQGPGGQGPYGPSAAAAAAAAGPGYGPGAGQQGPGSGGQQGGQGSGQQGPGGAGQGGPRGQGPYGPGAAAXAAAAAGGYGPGAGQQGPGSQGPGSGGQQGPGSQGPYGPSAAAAAAAAGPGYGPGAGQQGPGSXGPGSGGQQGPGGQGPYGPSAAAAAAAAGPGYGPGAGQQGPGSGGQQGGQGSGQQGPGGXGXGGPRGQGPYXPGAAAAAAAAGXYGPGAGQQGPGSQGPGSGGQQGPGSXGPYGPXAAAAAAAAXPGYGPGAGQXGPGSQGPGSGGQQGPGGQGPYGPSAAAAVAAAGPGYGPGAGQRPGSGGQQGGQGSGQQGRGGIGRGPRGQGPHGPGAAAAAAAAAGGYGPGAGQQGPGSQGPGSGGQQGPGSQGPXGPSAAAAAAAXPGYXPGAXQQGPGSQGPGSGGQQGPGGQGPYGPSAAAAAAAAGPGYGPGAXQQGPGSGGQQGGQGSGQQGPGGACQGGPRGQGPYGPGAAAAAAAAGGYGPGAGQQGPGSQGPGSGGQQGPGSQGPYGPSAAAAAAAAGPGYGPGAGQQGPGSQGPGSGGQQGPGGQGPYGPSAAAAAAAAGPGYGPGAGQQGPGSGGQQGGQGSGQQGPGGAGQGGPRGQGPYGPGAAAAAAAAAGGYGPGAGQQGPGSQGPGSGGQQGPGSQGPYGPSAAAAAAAAGPGYGPGAGQQGPGSQGPGSGGQQGPGGQGPYGPSAAAAAAAAGPGYGPGAGQQGPGSGGQQGGQGSGQQGPGGAGQGGPRGQGPYGPGAAPAAAAAAGGYGPGAGQQGPGSQGPGSGGQQGPGSQGPYGPSAAAAAAAAGPGYGPGAGQQGPGSQGPGSGGQQGPGGQGPYGPSAAAAAAAAGPGYGPGAGQQGPGSGGQQGGQGSGQQGPGGAGQGGPRGQGPYGPGAAAAAAAAGGYGPGAGQQGPGSQGPGSGGQQGPGSQGPYGPSAAAAAAAAGPGYGPGAGQQGPGSQGPGSGGQQGPGGQGPYGPSAAAAAAAAGPGYGPGAGQQGPGSGGQQGGQGSGQQGPGGAGQGGPRGQGPYGPGAAAAAAAAGGYGPGAGQQGPGSQGPGSGGQQGPGSQGPYGPSAAAAAAAAGPGYGPGAGQQGPGSQGPGSGGQQGPGGQGPYGPSAAAAAAAAGPGYGPGAGQQGPGSGGQQGGQGSGQQGPGGAGQGGPRGQGPYGPGAAAAAAAAGGYGPGAGQQGPGSQGPGSGGQQGPGSQGPYGPSAAAAAAAAGPGYGPGAGQQGPGSQGPGSGGQQGPGGQGPYGPSAAAAAAAAGPGYGPGAGQQGPGSGGQQGGQGSGQQGPGGAGQGGPRGQGPYGPGAAAAAAAAGGYGPGAGQQGPGSQGPGSGGQQGPGSQGPYGPSAAAAAAAAGPGYGPGAGQQGPGSQGPGSGGQQGPGGQGPYGPSAAAAAAAAGPGYGPGAGQQGPGSGGQQGGQGSGQQGPGGAGQGGPRGQGPYGPGAAAAAAAAGGYGPGAGQQGPGSQGPGSGGQQGPGSQGPYGPSAAAAAAAAGPGYGPGAGQQGPGSQGPGSGGQQGPGGQGPYGPSAAAAAAAAGPGYGPGAGQQGPGSGGQQGGQGSGQQGPGGAGQGGPRGQGPYGPGAAAAAAAAGGYGPGAGQQGPGSQGPGSGGQQGPGSQGPYGPSAAAAAAAAGPGYGPGAGQQGPGSQGPGSGGQQGPGGQGPYGPSAAAAAAAAGPGYGPGAGQQGPGSGGQQGGQGSGQQGPGGAGQGGPRGQGPYGPGAAAAAAAAGGYGPGAGQQGPGSQGPGSGGQQGPGSQGPYGPSAAAAAAAAGPGYGPGAGQQGPGSQGPGSGGQQGPGGQGPYGPSAAAAAAAAGPGYGPGAGQQGPGSQAPVASAAASRLSSPQASSRVSSAVSTLVSSGPTNPASLSNAISSVVSQVSSSNPGLSGCDVLVQALLEIVSALVHILGSSSIGQINYAASSQYAQLVGQSLTQALG

>Atri_MaSp2.2c

MNWSIRLALLGFVVFSTQTVFSAGQSATPWENSQLAEQFINSFLRFIGQSGAFSPNQLDDMSSIGDTLKTAIEKMAQSRKSSKSKLQALNMAFASSMAEIAVAEQGGLSLEAKTNAIENALISAFLETTGVVNQQFVSEIKSLIYMIAQASSNEISGSTAAAGGGSGGGGSGGRGGYGQGSYASASAAAAYGSAPQGTGGPAPQGPSQQGPVSQPSYGPSASVTVAVVGGRQQGPAGPSQQGPGQQGPGQQAPGGQGPYGPSAAAAAAASGGYGPGAGQQGGQGPGSQGPGSGGQQGPGIQGPYGPSAAAAAAAGPGYGPGAGQQGPGSQRPGAGQQGPGSQGQGGAGQQGPGGQGPYGPGAAAAAAAVGGYGPGAGQQGPGSGGQQGPGSQGPYGPSAAAAAAAGPGYGPGAGQQGPGSQGPGAGQQGPGSQGPGGAGQQGPGGQGPYGPGAAAAAAAVRGYGPGAGQQGPGSQGPGGGGQQGPGSQGPYGPSAAAAAAAGPGYGPGAGQQGPGSQGPGSGGQQGPGSLGPYGPSAAAAAAAGPGAGQQGPGSQGQGAGQQGPGSQGPGGAGQQGPGRQGPYGPGAAAAAAAVGGYGPGAGQQGPGSQGPGSGGPQGSGSQGPYGPSAAAAAGPGYGPGAGQQGPGSQGPGSGGQQGPGSQGPYGPSAAAAAAAGPGYGPGAGQQGPGSQGPGAGQQGPGSQGPGGAGQQGPGRQGPYGPGAAAAAAAVGGYGPGAGQQGPGSQGRGSGGPQGPGSQGPYGPSAAAAAAAGPGYGPGAGQQGPGSQGPGSGGQQGPGSQGPYGPSAAAAAAAGPGYGPGAGQQGPGSQGPGAGQQGPGSQGPGGASQQGPGGQGPYGPGAAAAAAAVGGYGPGAGQQGPGSQGPGSGGQQGPGSQGPYGPSAAAAAAAGPGYGPGAGQQGPGSQGQGAGQQGPGSQGPGGAGQQGPGRQGPYGPGAAAAAAAVGGYGPGAGQQGPGSQGQGSGGPQGPGSQGPYGPSAAAAAAAGPGYGPGAGQQGPGSQGPGSGGQQGPGSQGPYGPSAAAAAAAGPGYGPGAGQQGPGSQGPGAGQQGPGSQGPGGAGQQGPGRQGPYGPGAASAAAAVGGYGPGSGQQGPGSQGPGSGGPQGPGSQGPYGPSAAAAAAAGPGYGPGAGQQGPGSQGPGSGGQGPGSQGPYGPSAAAAAAAGPGYGPGAGQQGPGSQGPGAGQQGPGSQGPGGAGQQGPGGQGPYGPGAAAAAAAVGGYGPGAGSQGPGSGGPQGPGSQGPYGPSAAAAAAAGPGYGPGAGQQGPGSQGPGSGGQQGPGSQGPYGPSAAAAAAAGPGYGPGAGQQGPGSQGPGAGQQGPGSQGPGGAGQQGPGRQGPYGPGAAAAAAAVGGYGPGAGQQGPGSQGQGSGGPQGPGSQGPYGPSAAAAAAAGPGYGPGAGQQGPGSQGPGSGGQQGPGSQGPYGPSAAAAAAAGPGYGPGAGQQGPGSQGPGAGQQGPGSQGPGGAGQQGPGRQGPYGPGAAAAAAAVGGYGPGSGQQGPGSQGPGSGGPQGPGSQGPYGPSAAAAAAAGPGYGPGAGQQGPGSQGPGSGGQQGPGSQGPYGPSAAAAAAAGPGYGPGAGQQGPGAGQQGPGSQGPGGAGQQGPGRQGPYGPGAAAAAAAVGGYGPGAGQQGPGSQGPGSGGPQGPGSQGPYGPSAAAAAAVGPGYGPGAGQQGPGSQGPGSGGQQGPGSQGPYGPSAAAAAAAGPGYGPGAGQQGPGSQGQGAGQQGPGSQGPGGAGQQGPGRQGPYGPGAAAAAAAVGGYGPGSGQQGPGSQGPGSGGPQGPGSQGPYGPSAAAAAAAGPGYGPGAGQQGPGSQGPGSGGQQGPGSQGPYGPSAAAAAAAGPGYGPGAGQQGPGSQGPGAGQQGPGSQGPGGAGQQGPGRQGPYGPGAAAAAAAVGGYGPGAGQQGPGSQGPGSGGPQSPGSQGPYGPSAAAAAAAGPGYGPGAGQQGPGSQGPGSGGQQGPGSQGPYGPSAAAAAAAGPGYGPGAGQQGPGSQGPGAGQQGLGSQGPGGAGQQGPGRQGPYGPGAAAAAAAVGGYGPGAGQQGPGSQGPGSGGPQGPGSQGPYGPSAAAAAAVGPGYGPGAGQQGPGSQGPGSGGQQGPGSQGPYGPSAAAAAAAGPGYGPGAGQQGPGSQGQGAGQQGPGSQGPGGAGQQGPGRQGPYGPGAAAVAAAVGGYGPGSGQQGPGSQGPGSGGPQGPGSQGPYGPSAAAAAAAGPGYGPGAGQQGPGSQGPGSGGQQGPGSQGPYGPSAAAAAAAGPGYGPGAGQQGPGSQGPGSGQQGPGSQGPGGAGQQGPGRQGPYGPGAAAAAAAVGGYGPGAGQQGPGSQGPGSGGPQGPGSQGPYGPSAAAAAAAGPGYVPGAGQQGPGSQGPGSGGQQGPGSQGPYGPSAAAAAAAGPGYGPGAGQQGPGSQGPGAGQQGPGSQGPGGASQQGPGGQGPYGPGAAAAAAAVGGYGPGAGQQGPGSQGPGSGGQQGPGSRGPYGPSASAAAAAGPGYGPGAGQQGPGSQGPGSGGQQGPGSQGPYGPSAAAAAAAGPGYGPGAGQQGPGSQGPGAGQQGPGSQGPGGAGQQGPGRQGPSGPGAAAAAAAVGGYGPGAGQQGPGSQGPGSGGQQGPGSQGPYGPSAAAAAAAGPGYGPGAGQQGPGSQGPGSGGQQGPGSQGPYGPSAAAAAAAGPGYGPGSGQQGPGSQGPGAGQQGPGSQGPGGAGQQGPGRQGPYGPGAAAAAAAVGGYGPGAGQQGPGSQGPGSGGQQGPGSQGPYGPSAAAAAAAGPGYGPGAGQQGPGSQGPGSGGQQGPGSQGPYGPSAAAAAAAGPGYGPGAGQQGPGSQGPGAGQQGPGSQGPGGSGQQGPGRQGPYGPGAAAAAAAVGGYGPGAGQQGPGSQGPGSGGQQGPGSQGPYGPSAAAAAAAGPGYGPGAGQQGPGSQGPGAGQQGPGSQGPGGAGQQGPGRQGPYGPGAAAAAAAVGGYGPGAGQQGPGSQGPGSGGQQGPGSQGPYGPSAAAAAAAGPGYGPGAGQQGPGSQGPGSGGQQGPGSQGAYGPSAAAAAAAGPGYGPGAGQQGPGSQGPGSGQQGPGSQGPGGAGQQGPGRQGPYGPGGAAAAAAVAGYGPGAGQQGPGSQGPGSGGQQGPGSQGPYGPSAAAAAAAGPGYGPGAGQQGPGSQGPGSGGQQGPGSQGPYGPSAAAAAAAGPGYGPGAGQQGPGSQGPGAGQQGPGSQGPGGAGQQGPGRQGPYGPGGAAAAAAVGGYGPGAGQQGPGSQGPGSGGQQGPGSQGPYGPRAAAAAAAGPGYGPGAGQQGPGSQGPGSGGQQGPGSQGPYGPSAAAAAAAGPGYGPGAGQQGPGSQGLGAGQQGPGSQGPGGAGQQGPGRQGPYGPGAAAAAAAVGGYGPGAGQQGPGSQGPGSGGPQGPGSQGPYGPSAAAAAAAGPGYGPGAGQQGPGSQGPGSGGQQGPGSQGPYGPSAAAAAAAGPGYGPGAGQQGPGSQGPGAGQQGPGSQGPGGAGQQGPGRQGPYGPGGAAAAAVGGYGPGAGQQGPGSQGPGSGGQQGPGSQGPYGPSAAAAAAAGPGYGPGAGQQGPGSQGPGSGGQQGPGSQGPYGPSAAAAAAAGPGYGPGTGQQGPGSQGPGAGQQGPGSQGPGGAGQQGPGRQGPYGPGGAAAAAAVGGYGPGAGQQGPGSQGPGSGGQQGPGSQGPYGPSAAAAAAAGPGYGPGAGQQGPGSQGPGSGGQQGPGSQGPYGPSAAAAAAIGPGYGPGAAQQGPGSQAPVASAAASRLSSPQAGSRVSSAVSSLVSNGPTNPASLANAISSVVSQVSASNPGLSGCDVLVQALLEIVSALVHILGSSSIGQINYAASSQYAQMVGNSVAQALG

>Aur_MaSp3a

MAWIARLPLLVLVALCTQTMIVHGQDSHPWKDTRTTESFMENFVEYFRQSGYFNSDDIESIKDLADTLIQSLNEMQAKGKNSHQVLQALNMGFAAGVAELVNSDGINLKEKQNAIREAMKKSQLQTTGVINESFMNEMDKLMQMFSQINALNDDSVGYGAGAVSYASSASASNAQGIGQNFGYQGQGSSSSSVSSISVGGLPQGPVGSDSYEYSLSVNSLSGLPNAYGGQYDSGVGMEQGSLGTGSSGGAAVATASGGASGNGYGPGYGGIGGFGLGGTSAAVAVGRAGESGYGRGGNRLGGVRAAASSGVGPGGPGYGGDGYSGTGRSGPGVAAAAAASGGRGGGDRYGPLGAGGYGQGSGSGTGGAAAAAASSGEGPGGAGYSGDGYGGPGGSGPGSAAAAAASGGRGGDGRYGQQGAGGYGQGGNGLGGAGAAATSGEGPGGAGYGGPGGSGPGSAAAAAASGGRGGGGRYGQQDADGYGQGGSGLGGVGAAANAASSDEGPGGAGYGGEGGSGSGSAAAAAASGGQGGGGRYGPQGVGGYGQGGSGLGGAGAAASSGEGPGGAGYGGDGYGGPGGSGPGSAAAAAVSGGRGGSGRYGQQGAGGYGQGNGSEAAGAAAAAASSGGGPGGAGYGGQGGTGPGSAASAATSGGRGGGGRYGPQGAGGYGQGGNGLGGAGAAASSGEGPGGAGYGGPGGSGPGSAAAAAVSGGRGGAGRYGQQGADGYGQGGSGLGGAGAAAAAASSGEGPGDAGYGDDGYGGPGGSGPGSAAAAAASGGRGGDGRYGQQGAGGYGQGGNGLGGAGAAASSGEGPGGAGYGGPGGSGPGSAAAAAASGGRGGGGRYGLQGAGGYGQGGSEIGGVGAAASAASSGEGPGGAGYGGDGYGGPGGSGPGSASAAAASGGRGGFGGYGPQGARGYGQGGSGLGGVGAAASAASSVEGPAGAGYVGDGYGGPGWSGPGTAAAAAASGGRGGGRRYGQQGAGGYGQGNGSEGAGAAAAAASSSEGPGGAGYGGDGYVGQGGSGPGGAASAASALGGQGVGGGYGQQGAGGYGEGGSGSGGSGAAAAAASSGEGQGGARYSGDGYAVPGGSGQDGTASAAASASGIRGPGGLRGSkeiikkiivhrrvgsasdaeasvieenGYGGEGGYGAGYDGQGGSAPGGAAAAAASGGQGGGSGYGPLGAGGYGQGGSGLGGAGAAAAAALSGEGPGGAGYGGPGLSGPGSAAAAAASGGRGGGGRYGQQGAGGYGQRGSGLGGEGSAAAAASSGEGSGGAGYGGDGYGGPGGSGLGAAAAAAASGGRGGGGRYGQQGAGGYGQGNGSEGAGASAAAASSGEGPGGAGYGGQGGSGPGSAASAAALGGRGGGGRYGPQGAGGYGQGGSGLGGAGEGAAAAAASSGEGPGGIDNGGDGYGGPGGSGPGSAAAAAASGGQVGGSRYGPQGAGGYGQRSGSVRARAAAAAASSFEISGGAGYDGQVVSGPGSAAAAAASGGRGSGGRYGPQGADGYGQGSGSEGAGAAAAAASSSEGPGGAGYRGQGRSGSGSAAAAAASGGQGGGGRYGPQGAGGYGQRGSGLGGAGAAAAAVSSGQGPGGADYGRDAYGGPEGSGPGSAAAAAASGGRGGGGRYGPQGAGGYGPGGAGVAAAAASSGEGPGGAGYGGDGYGGPGGNGPDNAAAAAASGGRGGGGRYGPQGAGGYGQGGSGLGEGAGEGGAGAAASSGEGPGGAGYDDDGYGGPGGSGPGSAAAAAASGGRGGGGRYGQQGAGGYGQGGSGLGGVGAATSAASSGEGPGGGGYGGEGGSVPGSAAAAAASGGQGGGGRYGPQGAGGYGEGGSGLGGAGAAAAAASSVEGPGGAGYDDDGYGAPGGSGPGSAAAAAASGGRGGGGRYGQQGAGGYGQGGSGLGGVGAATSAASSGEGPGGGGYGGEGGSGPGSAAAAAASGGREGGGRYGPQGAGGYGQGGSGLGGAGAAASSVEFPGGAGYGDDGYGGPGGSEGNGVASAGTSSNGGPVELGSGRRGGSGLGGALSSAASTGGFSGPGGLRGPkeiikkiivhrrlgsasdasasvieenLYGPEAIGYYGQGGRGAGGAGAASAAVSSSEGPGGVGYGGQLGSGSGSAAAAAPLGGRGGGGRYGAEGAGGFGKGGGGFGGAAAAASGLGPGENGFDAAGYGGDGEAGPEGAAAAAAASGGGGSYGPQGAGGYGEGGSGSIGAGAAASAASSSGPGGASYGGQGESGPGGAAGAAAAAAGGRGGRGRYGAYGAGGYGEGGSGSGGTGGFGSGSDVYGGQGGSGEGAAAAAAVESSGQGGRGRLGSNGALGARAAGAAASSGVGSGGAGYGGDGYDGQGGSEGNGAAAAAASSDEGPGGFRPAVRGGSGQGGAVSSAASAGGRAGRDRLGSQGAGGAGGFDYGRDGYGGQGGSLEGGAAAAAAAGSAGYGPQGAGDYGQGGSGPGGNGAAAASASSTAASVASRLSSPATLSRVSSAVSLFLDDDLDYPVAFSNAFDNVVSGITLSYSNISGCELLVQSLMEVLSAVLGTAYGLNANSSVDIVRSVVNRFDY

>Aur_MaSp3b

MAWIARLPLLVLVALCTQTMIVHGQDSHPWKDTRTTELFFENFVECIRQSSYFNSEDIESIKVLAETLIQSLNGMQAKGKTSHQMLQALNMGYAAGVAELVNSDGSNLQEKRNAIREAMKKSLLQATGVVNESFMNEMDKLMQMFSQINGLNDDSGGYGAGAVSYASSASASNAQGIGQNFGYQGQGSSSSSVSSISVGGLPQGPVGSGTYEYSLSVNSLSGSPSGYGGQYVRGVGVGQGGFGASGAGGAAAATTSGGASGNGYGFGYGGIGGTGLGGASAAVAVGIAGQGGYGQEGKGLERTGAAASSSVGPEGAIYGGEGYDGQGGSGPSGTAAAATASDGQGGGGRYGPQGAGGYGEGGSGSGGAAAAAASSGVGPGGAGYGGDGYGGQGGSGPGGAAAAAGRGGDGRYGQQGFGGFGQRGSETGGAGAAAAAASSGEGPGGTGYGGIEGSGPGGAASAAAASGGRGAGGRYGPEGSGGYGQGGRGSGGAGAAASSGVGPGGAGYGGDGYGGQGGSGPGSAASAAAASDGEGGRGRYGQQGSGGYGQRGSGSGEASASAAAASSGGGPGGAGYGGDGYGGQGGSGPGGAAASAASGGRGGGRYGQQGFGGFGQGGSETLGAGAAAAAASSGEGSGGAGYGGIGRSGPGGAASAAAGGRGAGGRYGPEGSGGYGQGGRGSGLAGAAASSGVGQGGAGYGGDDYGGQGGSGPVSAASAAAASDEQGGDGRYGQQGSGGYGQGGSGSGGASASAAAASSGEGPGGAGYGGDGYGGQGGSGPGGAAASAASGGRGGGRYGQQGFGGFGQGGSETGGAGAAAAAASSGEGPGGAGYGGIGGSGPGGAASAAAASGGRGAGGRYGPEGSGGYGQGGRGSGGASAAASSGVGPGGAGYGGDGYGGQGGSGPGSAASAAAASDGQGGRGRYGQQGSGGYGQGGSGSGGASASAAAASSGEGPGGAGYGGDGYGGQGGSGPVGAAASAASGGRGGGRYGQQGFGGFGQGGSETGGAGAAAAAASSGEGPGGAGYGGIGGSGPGGAASAAAASGGRGAGGRYGPEGSGGYGQGGRGSGGAGAAASSGVGPGGAGYGGDGYGGPGGSGPGSAASAAAASDGQGGRGRYGQQGSGGYGQGGSGSGGASASAAAASSGEGPGGAGYGGDGYGGQGGSGPGGAAASAASGGRGGGRYGQQGFGGFGQGGSETGGAGAAAAAASSGEGPGGAGYGGIEGSGPGGAASAAAASGGRGAGGRYGPEGSGGYGQGGRGSGGAGAAASSGVGPGGAGYGGDGYGGQGGSGPGSAASAAAASDGQGGRGRYGQQGSGGYGQGGSGSGGASASAAAASSGEGPGGAGYGGDGYGGQGGSGPGGAAASAASGGRGGGRYGQQGFGGFGQGGSETGGAGAAAAAASSGEGPGGAGYGGIEGSGPGGAASAAAASSGRGARGRYGPEGSGGYGQGGRGSGGAGAAASSGVGPGGAGYGGDGYGGPGGSGPGSAASAAAASDGQGGRGRYGQQGLGGYGQGGSGSGGASASAAASLSGEGPGGTGYGGDGYDGQVGSGPAGTAASAASGGRGGGRYGQQGFGGFGQGGSETGGAGAAAAAASSGEGPVGAGYGGIEGSGPGGAASAAAASGGRGARGRYGPEGSGGYGQGGRGSGGASAAASSGVGPGGAGYGGDGYGGQGGSGPGSAASAAAASDGQGGRGRYGQQGSGGYGQGGSGSGGASASAAAASSGEGPGGTGYGGDGYGGQGGSGPGGAAASGGRGGGRYGLQGFGGFGQGGSETGGAGAAAAAASSGEGPGGAGYGGIEGSGPGGAASAAAASSRRGARGRYGPEGSGGYGQGGRGSGGASAAASSGVGPGGAGYGGDGYGGQGGSGPGSAASAAAASDGQGGRGRYGQQGSGGYGQGGSGSGGASASAAAASSGEGPGGAGYGGDGYVGQGGSGPGGAAASAASGGRGGGRYGQHGFGGFGQGGSETGGAGAAAAAASSGEGPGGAGYGGIGGSGPGGAASAAAASGGRGAGGRYGPEGSGGYGQGGRGSGGASAAASSGVGPGGAGYGGDGYGGQGGSGPGSAASAAAASDGQGGRGRYGQQGSGGYGQLGSGSGGASASAAAATSGEGPGGTGYGGDGYGGQGGSGPGGEASAASASGGQGGGGGYGQQGAGDYDQGGSGSGGSGAAAAAASSGEGPGGARYGGDGYAVQGGSGQDGIASAAASASGIGGPGGLRGSkeiikkiivhrrvgsasdaeasvieenGYGGQGGYGAGYDGQGGSAPGGEAAAASGGRGGGGRYSSQGAGRYGEGGSGSRGAGAAASAASSSGTGGTGYSGQGVSGPGGAASAAAAASGGRGGRGRYNAEGAGGYGEGRNESGGTGGFGSGSDGYGEQGESGGSGAAAAAGSAGHGPQGAGDYGQGGSGSGGNEAAAASSTAASVASRLSSPAALSRVSSAVSVFLDDDLDYPVAFTNAFDNVVSGITLSNSDISGCELLVQSLMEVLSAVLGTAYGLNANSSVDIVRSVVNRFDY

>Aarg_MaSp3a

MAWITRLPLLVLVALCTQSIIVHGQDSHPWSDVRTTESFMKNFVECIRQSSYFNTDDIESIRDLSDTMIQSLNGMTAIGKTSHQMLQALNMGYAAGVAELVNSDGFYVQEKRNAIREAMRNSLLQTTGVVNESFINEMDSLMQMFSQINVLNEDSGGNGASAASSASASNVPGIGQSLGPQGQGSSSVSVSSTSVGGLQQRPVGSGSYEYPLSVNSIGGSGYGPGGSGTGGSGAAAAAASSGGGTGSPGYGGQGGYGPGGQAAAAAASDGQGGTGGGRYGPQGAGGYGQGGYGPGGSGAAAAAASSGGGSGSPGYGPGGTAAAAAASNGQGGTGGGRYGPQGTGGYGQGGYGPGGSGAAAAAASSDGGTGGPGYGGQGGYGPGGTATAAAASDGQGGTGGGRYGPQGAGGYGQGGYGPGGSGAAAAAASSGGGTGSPGYGGQGGYGPGGTAAAAAASDGQGGTGGGRYGPQGAGGYGQGGSGSGGSGAAAAAASSSGGTGSPGYGGQGGYGPGGVRYGPQGAGGYDQGGIGTGSGLGAASAAASSGAGQGTIGYGGEGIGGYGPGGGDAAAAASSGQGGGRGDRYGPQGYGGYGQGGRGPGESGAAASAASAGEGTGSPGYGGQGGYGAGGAAAAAAASGGQGGGGRYGPQGAGGYGQGGIGTGSGPGAAAAAASSGAGTGSPGYGGQGGYGPGGTAAAASASDGQGGTGGGRYGPQGAGGYGQGRSGSGGSGAAAAAASSGAGTGSPGYEGQGGYGPGGTAAAAAASDGQGGTGGGRYGPQGAGGYGQGGYGPGESEAAAAAASSGAGPGYGGRGRkeiikkiivrrkggsqydteafeiegnnYGAQGGSGSGPGSSAAAASSSTGPGSAGYGGQGGSGPGGEAAAAASGGQGGRGRYGSQGAGGYGQGGPGYGSGAAAAGASSGTGDDGYGGQGGSGPGGAAAAAASSGGQGGRGRYGSQGAGGYGQGGPGSGSGAAAAAASSGTGDDGYGGQGGSGPGGASAAAASSGGQGGRGRYGPQGAGGYGQGGPGSGSGAAAATASSGTGDDGYGGQGGSGPGGAAAAAASSGGQGGRGRYGPQGAGGYGQGGPGSGSGAAAAAASSGTGEGDDGYGGQGGSGPGGAAAAASGGQGGRGRYGPQGAGGYGQGGPGSGSGAAAAAASSGTGDDGYGGQGGSGPGGAAAAAASSGGQGGRGRYGSQGAGGYGQGGPGSGSGAAAAAASSGTGDDGYGGQGGSGPGGAAAAAASSGGQGGRGRYGSQGAGGYGQGGPGSGSGAAAAAASSGTGDDGYGGQGGSGSGGAAAAAASSGGQGGRGRYGSQGAGGYGQGGPGSGSGAAAAAASSGTGDDGYGGQGGSGPGGAAAAAASSGGQGGRGRYGSQGAGGYGQGGPGSGSGAAAAAASSGTGDDGYGGQGGSGSGGAAAAAASSGGQGGRGGYGSQGAGGYGQGGPGSGSGAAAAAASSGTGDDGYGGQGGSGSGGAAAAAASSGGQGGRGRYGPQGAGGYGQGGPGSGSGAAAAAASSGTGDDGYGGQGGSGPGGAAAAAASSGGQGGRGRYGPQGAGGYGQGGPGSGSGAAAAAASSGTGDDGYGGQGGSGPGGAAAAAASSGGQGGRGRYGPQGAGGYGQGGPGSGSGAAAAAASSGTGDDGYGGQGGSGPGGASAAAASSGGQGGRGRYGPQGAGGYGQGGPGSGSGAAAAAASSGTGDDGYGGQGGSGPGGASAAAASSGGQGGRGRYGPQGAGGYGQGGPGSGSGAAAAAASSGTGDDGYGGQGGSGPGGAAAAASSGGQGGRGRYGPQGAGGYGQGGPGSGSGAAAAAASSGTGDDGYGGQGGSGPGGAAAAAASSGGQGGRGRYGPQGAGGYGQGGPGSGSGAAAAAASSGTGDDGYGGQGGSGPGGAAAAAASSGGQGGRGRYGPQGAGGYGQGGPGSGSGAAAAAASSGTGDDGYGGQGGSGPGGAAAAAASSGGQGGRGRYGPQGAGGYGQGGPGSGSGAAAAAASSGTGDDGYGGQGGSGPGGAAAAAASSGGQGGRGRYGPQGAGGYGQGGPGSGSGAAAAAASSGTGDDGYGGQGGSGPGGAAAAAASSGGQGGRGRYGPQGAGGYGQGGPGSGSGAAAAAASSGTGDDGYGGQGGSGSGGAAAAAASSGGQGGRGRYGSQGAGGYGQGGPGSGSGAAAAAASSGTGDDGYGGQGGSGSGGSAAAAASSGGQGGRGRYGSQGAGGYGQGGPGSGSGAAAAAASSGTGDDGYGGQGGSGPGGAAAAAASSGGQGGRGRYGPQGAGGYGQGGPGSGSGAAAAAASSGTGDDGYGGQGGSGPGGAAAAAASSGGQGGRGRYG?QGAGGYGQGGPGSGSGAAAAAASSGTGDDGYGGQGGSGPGGAAAAAASSGGQGGRGRYGPQGAGGYGQGGPGSGSGAAAAAASSGTGDDGYGGQGGSGPGGAAAAAASSGGQGGRGRYGPQGAGAYGQGGPGSGAGAAAAAASSGTGDDGYGGQGGSGPGGASAAAASSGGQGGRGRYGPQGAGGYGQGGPGSGSGAAGAAASSGTGDNGYGGQGGSGPGGAAAAAASSGGQGGRGRYGPQGAGGYGQGGPGSGSGAAAAAASSGTGDDGYGGQGGSGPGEAAAAAASSGRQGGRGRYGPQGAGGYGQGGPGSGSGAAASSGTGDDGYGGQGGSVAGGAAAAAASSAGQGGRGRYGSQGAGGYGQGGNGRGGNEAAAATASSTAALVANRLSSPSSLSRVSSAVSVFLDDDLEYPVAFSNAFDNVVSGITLSNSDISGCELLVQSLMEVLSAVLGTAYGLNANSSVDIVRSVVNRFD

>Aarg_MaSp3b

MAWITRLPLLVLVALCTQSIIVHGQDSHPWSDVRTTESFMKNFVECIRQSSYFNTDDIESIRDLSDTMIQSLNGMTAIGKTSHQMLQALNMGYAAGVAELVNSDGFYVQEKRNAIREAMRNSLIQTTGVVNESFMNEMDKLMQMFSQINVLNEDSGGNGVSAASSASASNVPGIGQSLGPQGQGSSSVSVSSTSVGGLQQRPVGSGSYEYSLSVNSIGGSGYGQGGYGPGGSGAAAVAASSGGGSGSPGYGGQVGYGPGGTAAAAAASDGQGGTGGGRYGPQGAGGYGQGGYGPGGSGAAAASSGGGTGSPGYGGQGGYGPGGTAAAAAASDGQGGTGGGKYGPQGAGGYGQGGYGPSGSGAAAAAASSGGGTGSPGYGPGGSAAAAAASDGQGGTGGGRYGPQGAGGYGQGGSGQDGTAAAAASSSGGTGSPGYGGQGGYGPGGTGGVRYGPQGAGGYGQGGIGTGSGLGAAAAAASSGAGPGTIGYGGEGIGGYGQGGADAAAAALGGQGGGRGGRYGPQGYGGYGQGGYGPGGSGAAAAAASSGEGTGRSGYGEQGGSGTDSAAAAAAAAGGGGGRYGPQGAGGYGQGGRGPGGSGAAAAAASAGEVTGSNGYGGQGGYGPGGAAAAAAASGGQGGGGRYGSQGAGGYGQGGIGTGSGPGAAAAAASSGAGKGSLGYGGQGGYGSDGTAAAAAASDGQGGTGGGRYGSQGAGGYGQRGSGPGGSGAAAAADSSGAGTGSPGYGGQGGYGPGGTAAAAAASDGLVGTGGGRYGPQGAGGYVQGGYGPGGSGAAAAAASSGAGPGYGERGRkeiikkiivrrkggsqydteafeiegnnYGPQGGSGSGSGSSAAAASSSTGSGSAGYGEQGESGPGGAAAAAASSGGQGGRGRYGPQGAGGYGQGGPGYGSGAAAAAASSGTGDDGYGGQGGSGPGGAAAAAASSGGQGGRGRYGPQGAGGYGQGGPGSGSGAAAAAASSGTGDDGYGGQGGSGPGGAAAAAASSGGQGGRGRYGPQGAGGYGQGGPGSGSGAAAAAASSGTGDDGYGGQGGPGGAAAAAASSGGQGGRGRYGPQGAGGYGQGGPGSGSGAAASSGTGDDGYGGQGGSGPGGAAAAAASSGGQGGRGRYGPQGAGGYGQGGPGSGSGAAAAAASSGTGDDGYGGQGGPGG-AAAAAAASGGQGGRGRYGPQGAGGYGQGGPGSGSGAAAAAASSGTGDDGYGGQGGPGGAAAAAASSGGQGGRGRYGPQGAGGYGQGGPGSGSGAAAAAASSGTGDDGYGGQGGPGGAAAAAAASGGQGGRGRYGPQGAGGYGQGGPGSGSGAAAAAASSGTGDDGYGGQGGPGGAAAAAAASGGQGGRGRYGPQGAGGYGQGGPGSGSGAAAAAASSGTGDDGYGGQGGPGGAAAAAAASGGQGGRGRYGPQGAGGYGQGGPGSGSGAAAAAASSGTGDDGYGGQGGPGGAAAAAAASGGQGGRGRYGPQGAGGYGQGGPGSGSGAAAAAASSGTGDDGYGGQGGPGGAAAAAAASGGQGGRGRYGPQGAGGYGQGGPGSGSGAAAAAASSGTGDDGYGGQGGPGGAAAAAAASGGQGGRGRYGPQGAGGYGQGGPGSGSGAAAAAASSGTGDDGYGGQGGSGPGGAAAAAASSGGQGGRGRYGPQGAGGYGQGGPGSGSGAAAAAASSGTGDDGYGGQGGPGGAAAAAAASGGQGGRGRYGPQGAGGYGQGGPGSGSGAAAAAASSGTGDDGYGGQGGPGGAAAAAAASGGQGGRGRYGPQGAGGYGQGGPGSGSGAAAAAASSGTGDDGYGGQGGPGGAAAAAAASGGQGGRGRYGPQGAGGYGQGGPGSGSGAAAAAASSGTGDDGYGGQGGSG?GGAAAAAASSGGQGGRGRYGPQGAGGYGQGGPGSGSGAAAAAASSGTGDDGYGGQGGPGGAAAAAAASGGQGGRGRYGPQGAGGYGQGGPGSGSGAAAAAASSGTGDDGYGGQGGPGGAAAAAAASGGQGGRGRYGTQGAGGYGQGGPGSGSGAAAAAASSGTGDDGYGGQGGPGGAAAAAAASGGQGGRGRYGPEGAGRYGQGGRGSGSGAAASSGTGDDGYGGQGGPGGAAAAAAASGGQGGRGRYGPQGAGGYGQGGPGSGSGAAAAAASSGTGDDGYGGQGGSGPGGAAAAAASSGGQGGRGRYGPQGAGGYGQGAPGSGSGAAAAAASSGTGDDGYGGQGGPGGAAAAAAASGGQGRRGRYGPQGAGGYGQGGPGSGSGAAAAAASSGTGDDGYGGQGGPGGAAAAAAASGGQGGRGRYGPQGAGGYGQGGPGSGSGAAAAAASSGTGDDGYGGQGGPGGAAAAAAASGGQGGRGRYGPQGAGGYGQGGPGSGSGAAAAAASSGTGDDGYGGQGGSGSGGAAAAAASSGGQGGRGRYGSQGAGGYGQGGSGRGGNGAAAATASSTAALVANRLSSPSSLSRVSSAVSVFLDDDLEYPVAFSNAFDNVVSGITLSNSDISGCELLVQSLMEVLSAVLGTAYGLNANSSVDIVRSVVNRYDY

>Atri_MaSp3a

MAWIARLPLLVLVALCTQSIVVNGLDRHPWQDTGTTELFMENFVECIRQSSYFNNEDIESIRDLAETLIQSLNGMQAKGKTSHQVLQALNMGYAAGVAELVNSDGTNLQEKRNAVREAMKNSLLQATGEVNESFMNEMDKLMQMFSQINALKGDSGGYGAGAESYASSASASNIQGIGQNLGSQGQGLSSVSVSSASVGGLPQGPVGSGSYGYSLSVNSLGGSPSGYGGQYASGTGVGQGRIGTGAAGGAAAATASSGASLNGNGLGYGGIGGYGFDGASAAVAVGSGGQGGYGPGNLYGQGGSGAGASASSGEGTGGAGYGLDGNGGYGGSGSAAATAASASGGQGGDGRYGPQGTSGYGLGGIGSGGAGAAAATASGEGPGGNGYGGQGGPAASAAAAAASGERGGNGRYGPQGTSGYGLGGIGSGGAGAAAAAASGEGPGGNGYGGQGGSAASAAAAAASGERGGNGRYGPQGTSGYGLRGIGSGGAGAAAAAGEVPGGAGYGGQGGEAEAAAAASTSESGENGALPRSRefikkiivhrrlgsssnaeasvieeneyGPQAAGRYGIGGSGSGTAASAASSGNGLLGANNGGYGYGGQGGSGAAAASSGRGGDGRYGPRGVGAYGQGGSGLGAAAAAASSGEGPGGAGYGGQGGSGAAAAAASSGEGPGGAGYGRDGYGRDGYGGQGGSGAAAAAASSGEGPVGAGYGRDGYGRGGYGRDGYGGQGGSGAAAAAASSGEGPGGAGYGRDGYGGQGGSGDAAAAAASGGEGRDGRYGRRGSGSDGAGAAAAAASSGEGPGGAGYGGQGGSGAAAAAASSGEGPGGAGYGRDGYGRGGYGRDGLGGQGGSGAAAAAASSGEGTGGAGYGRDGYGRGGYGRGGYGRDGYGGQDGTGAAAAAASSGEGPGGAGYGRDGYGRGGYGRDGLGGQGGSGAAAAAASSGEGPGGAGYGGQGGSGAAAAAASSGEGPGGAGYGRDGYGRDGLGGQGGSGAAAAAASSGEGPGGAGYGGQGGSGAAAAAASSGEGPGGAGYGRDGYGGQGGSGDAAAAAASGGRGRDGRYGRRGSGSDGAGAAAAAASSGEGPGGAGYGGQGGSGAAAAAASSGEGPGGAGYGRDGYGRGGYGRDGYGRGGYGRDGLGGQGGSGAAAAAASSGEGPGGAGYGRDGYGGQGGSGAAAAAASSGEGPGGTGYGRDGYGRDGLGGQGGSGAAAAAASSGEGPGGAGYGGQGGSGAAAAASSSGEGPGGAGYGRDGYGRGGYGRDGLGGQGGSGAAAAAASSGEGPGGAGYGGQGGSGAAAAAASSGEGPGGAGYGRDGYGRDGLGGQGGSGAAAAAASSGEGPGGAGYGGQGGSGAAAAASSGEGPGGAGYGRDGYGGQGGSGDAAAAAASGGRGRDGRYGRRGSGSDGAGAAAAAASSGGAGYGGQGGSGAAAAAASSGEGPGGAGYGRDGYGRGGYGRGGYGRDGLGGQGGSGAAAAAASSGEGPGGAGYGGQGGSGAAAAAASSGEGPGGAGYGRDGYGRGGYGRDGLGGQGGSGAAAAAASSGEGPGGAGYGGQGGSGAAAAASSSGEGPGGAGYGRDGYGRGGYGRDGLGGQGGSGAAAAAASSGEGPGGAGYGGQGGSGAAAAAASSGEGPGGAGYGRGGYGGQGGSGDAAAAAASGGRGRDGRYGRRGSGSDGAGAAAAAASSGGAGYGGQGLSGAAAAAASSGEGPGGAGYGRDGYGRGGYGRGGYGRDGLGGQGGSGAAAAAASSGEGPGGAGYGGQGVSGAAAAAASSGEGPGGAGYGRDGYGRGGYGRDGLGGQGGSGAAAAAASSGEGPGGAGYGGQGGSGAAAAAASSGEGPGGAGYGRDGYGRGGYGRDGLGGQGGSGAAAAAASSGEGPGGAGYGGQGGSGAAAAAASSGEGPGGAGYGRDGYGRGGYGRDGLGGQGGSGAAAAAASSGEGPGGAGYGRGGYGRDGYGGQGGSGAAAAAASSGEGPGGAGYGRDGYGGQGGSGDAAAAASSGGGGGDGSYGRRGSGSDGAGAAAAAASSGGAGYGGQGGSGAAAAAASSGEGPGGAGYGRDGLGGQGGSGAAAAAASSGEGPGGAGYGGQGGSGAAAAAASSGEGPRGAGYGRDGYGRGGYGRDGYGEQGGSGAAAAAASSGEGPGGAGYGGQGGSGAAAAAASSGEGPGGAGYGRDGYGRGGYGRDGLGEQGGSGAAAAAASSGEGPGGAGYGGQGGSGAAAAAASSGEGPGGAGYGRDGYGRGGYGRDGLGGQGGSGAAAAAASSGEGPGGAGYGGQGGSGAAAAAASSGEGPGGAGYGRGGYGRDGYGGQGGSGAAAAAASSGEGPGGAGYGRDGYGGQGESGDAAAAASSGERGGDGSYGRRGSGSDGAGAVAAAASSGEGPGGAGYGGQGGSGAAAAAASSGEGPGGAGYGRDGYGRGGYGRGGYGRGGYGRDGLGGQGGSGAAAAAASSGEGPGGAGYGGQGGSGAAAAAASSGEGPGGAGYGRDGYGRGGYGRDGYGGQGGSGAAAAAASSGEGPGGAGYGGQGGSGAAAAAASSGEGPRGAGYGRDGYGRGGYGRDGYGGQGGSGAAAAAASSGEGPGGAGYGGQGGSGAAAAAASSGEGPRGAGYGRDGYGRGGCGRDGYGRDGYGGQGGSGAAAAAASSGEGPGGAGYGRDGYGRDGYGGQGGSGDAAAAASSGGQRQLWSEQGSGSDGAGAAAAAASSGEGPGGAGYGGQGGSGAAAAAASSGEGPGGAGYGRDGYGRGGYGRDGLGGQGGSGAAAAAASSGEGPGGAGYGGQGGSGAAAAAASSGEGPGGAGYGGQGGSGAAVAAASSGEGPGGAGYGRDGYGGQGGSGAAAAAAASSGRGGDGRYASGGSGSDGAAAASASSTAATVASRLSSPTSLSRVSSAVSIFLDDDLDYPEAFSNAFDNVVSGITLANSDISGCELLVQSLMEVLCAVMGTAYGLNANSSVDIVRNVVNRYDY

>Atri_MaSp3b

MTQIIFIRKHRSDKMRRRNIEPPASYHEIYDIKGWKIDRKINQSRRTKTTMTWIARLPLLVLVTLCIQSIIVHGLDSHPWQDASTTGLFMENFVQYIRQSSYFNSDDIDSIKVLADTLIQSLNGMQAKGKTSHQMLQALNLGYAAGVAELVNSDGTNLQEKRNAIREAMKNSLLQATGEVNESFMNEMDKLMQMFSQVNALNEDSGGYGAGAESYASSASASNIQGIGQNLGSQGQGSSSVSVSSASVGGLPQGPVGSGSYGFSLSVNSLGGSPNGYGGQYASGTGVELGRLGTGAAGGAAAATASGGASLNGNGFGYGGIGGYGLDGASAAVAVGSGGQRGYGQGIGFSQRGSGPGAAAAAASSGEGPGGTGYGGNGYGGQGGSGAAAAAAASGGRGGDGRYGPQGAGSYGPGGSGSGAAAAAASSGEGPSGAVYGGQGGSGAAAAVAASSGRGGDGRYGSLGAGSYGPGGSGSGSAAAAASSGEGQSGTGYGRNGYGGQDGSGAAAAAAASGGRGGEGRYGPQGAGSYGPGGNGSGAAAAAASSGEGPSGAGYGGNGYGGQGGSGAATAAAASGGRGGDGRYGPQGAGSYGPGGSGSGAAAAAVSSGEGESGAGYGENGYGLEDGSASATASGFGGSGGLRRSkevikkiivhrrlganGVDYDGQGGSRSDAAAATSGGRGGDGRYGLRGVGGYGPEGSGSGAAAAAASSGEGPGGAGYGRDGYGGQGGSGAAAAVASSGEGPGGAGYGGDRYGGQGGSGAAAAAAASGGLEGDGRYGLRGVVGYGPGGSGSGAAAAAASSGEGPGGAGYGRDRYGGEGGSEAAASSGEGPGGAGYGGQGGSGASAAAASSGEGGRGGYGRYGPRGIGSNGAGAAAAAGEGPGGAGYGGQGGSGAAAASASSGKGGRGGYGRYGPSGSGSNGAGAAAAAASSGEGPGGAGYGRDGYGGQGGSEAAAAAASSGEGPGGAGYGGQGGSGASAAAASSGEGGRGGYGRYGPRGIGSNGAGAAAAASEGPGGAGYGGQGGSGASAAAASSGEGGRGGYGRYGPSGSGSNGAGAAAAAASSGEGPGGAGYGGDGYGGQGGSGAAAAAAASGGREGDGRYGLRGVVGYGPGGSGAGAAAAAASSGEGPGGAGYGRDGYGGQGGSEAAAAAASSGEGPGGAGYGGQGGSGASAAAASSGEGGRGGYGRYGPRGIGSNGAGAAAAAGEGPGGAGYGGQGGSGASAAAASSGEGGRGGYGRYGPRGSGSNGAGAAAAAASSGEGPGGAGYGGTKWGTGGSGASAAAASSGEGGRGGYGRYGAGAAAAAASSGEGPGGAGYGRDGYGGQGGSEAAAAAASSGEGPGGAGYGGQGGSGASAAAASSGEGGRGGYGRYGPRGSGSNGAGAAAAAGEGPGGAGYGGQGGSGASAAAASSGEGGRGGYGRYGPSGSGSNGAGAAAAAASSGEGPGGAGYGGDGYGGQGGSGAAAAAAASGGREGDGRYGLRGVVGYGPGGSGAGAAAAAASSGEGPGGAGYGRDGYGGQGGSEAAAAAASSGEGPGGAGYGGQGGSGASAAAASSGEGGRGGYGRYGPRGIGSNGAGAAAAAGEGPGGAGYGGQGGSGASAAAASSGEGGRGGYGRYGPSGSGSNGAGAAAAAASSGEGPGGAGYGGDGYGGQGGSGAAAAAASSGGREGDGRYGLRGVVGYGPGGSGAGAAAAAASSGEGPGGAGYGRDGYGGQGGSEAAAAAASSGEGPGGAGYGGQGGSGASAAAASSGEGGRGGYGRYGPRGIGSNGAGAAAAAGEGPGGAGYGGQGGSGASAAAASSGEGGRGGYGRYGPSGSGSNGAGAAAAAASSGEGPGGAGYGGDGYGGQGGSGAAAAAAASGGREGDGRYGLRGVVGYGPGGSGAGAAAAAASSGEGPGGAGYGRDGYGGQGGSEAAAAAASSGEGPGGAGYGGQGGSGASAAAASSGEGGRGGYGRYGPRGIGSNGAGAAAAAGEGPGGAGYGGQGGSGASAAAASSGEGGRGGYGRYGPSGSGSNGAGAAAAAASSGEGPGGAGYGGDGYGGQGGSGDAAAAAASGGREGDGRYGLRGVVGYGPGGSGAGAAAAAASSGEGPGGAGYGRDGYGGQGGSEAAAAAASSGEGPGGAGYGGQGGSGASAAAASSGEGGRGGYGRYGPRGIGSNGAGAAAAAGEGPGGAGYGGQGGSGASAAAASSGEGGRGGYGRYGPSGSGSNGAGAAAAAASSGEGPGGAGYGGDGYGGQGGSGAAAAAASSGGREGDGRYGIRGVVGYGPGGSGAGAAAAAASSGEGPGGAGYGRDGYGGQGGSEAAAAAASSGEGPGGAGYGGQGGSGASAAAASSGEGGRGGYGRYGPRGSGSNGAGAAAAAGEGPGGAGYGGQGGSGASAAAASSGEGGRGGYGRYGPRGIGSNGAGAAAAAASSGEGPGGAGYGGDGYGGQGGSGAAAAAASSGEGPGGAGYGGQGGSGASAAAASSGEGGRGGYGRYGPRGSGSNGAGAAAAAGEGPGGAGYGGQGGSGASAAAASSGEGGRGGYGRYGPSGSGSNGAGAAAAAASSGEGPGGAGYGGDGYGGQGGSGAAAAAASSGGREGDGRYGIRGVVGYGPGGSGAGAAAAAASSGEGPGGAGYGRDGYGGQGGSEAAAAAASSGEGPGGAGYGGQGGSGASAAAASSGEGGRGGYGRYGPRGSGSNGAGAAAAAGEGPGGAGYGGQGGSGASAAAASSGEGGRGGYGRYGPRGIGSNGAGAAAAAASSGEGPGGAGYGGDGYGGQGGSGAAAAAASSGGREGDGRYGLRGVVGYGPGGSGAGAAAAAASSGEGPGGAGYGRDGYGGQGGSEAAAAAASSGEGPGGAGYGGQGGSGASAAAASSGEGGRGGYGRYGPRGSGSNGAGAAAAAGEGPGGAGYGGQGGSGASAAAASSGEGGRGGYGRYGPRGIGSNGAGAAAAAASSGEGPGGAGYGGDGYGGQGGSGAAAAASSSGGREGDGRYGLRGVVGYGPGGSGAGAAAAAGSSGEGPGGAGYGRDGYGGQGGSEAAAAAASSGEGPGGAGYGGQGGSGASAAAASSGEGGRGGYGRYGPRGSGSNGAGAAAAAGEGPGGAGYGGQGGSGASAAEASSGEGGRGGYGRYGPRGIGSNGAGAAAAAASSGEGPGGAGYGRDGYGGQGGSGAAAAAAASSGRGGDGRYGPGGSGSDGAAAASASSTAATVASRLSSPTSLSRISSAVSIFLDDDLDYPEAFSNAFDNVVSGITLANSDISGCELLVQSLMEVLCAVMGTAYGLNANSSVDMIRSVVNRYDY

>Aaur_Masp2.3

MNWSKTFSLLCLLVLSTQALLLVEAARSPWESTQLAESFLKSFLRAIARSGAFSSNQLDDMSTIGETLTTSIEKLASSSKTSKAKLQALDMAFASSMAEIAVAEEGGLSINEKTEAIGNALKTAFLETTGRINAQFVSEIKSLIFLIAQATSNEITSASPTGATGYGTPGQGGTYASVSIAGSYSQTPQRPQTTGQEPSSQGPLSPEPQGTAFSSVSSYGPGPQGPSGPGPQTPLPQGPSGPGLQISGPSVGILSSGPGPQGSSGPIPQESFPEGSSSPATQGPSVSSISFFEPGPKGPGGPGPQAPLPQGPSGPGPQRPTFSSVSAYGPGSLGPSGPGPQAPSPQGPSGPGPQGPTVSSISFFGPGPQAPSLQGPSGLGTQGPEQSASVLTSYGPGPQGSLKVPSAQGPYIPKSQGPAVSSVSFFGPGRQGSSRPSPQGPIPQGPSGPGPQGPVARSVSFFGPGTQGPTGPSPEGPITQGPSVPGPQGSAVSSVSFFGPGSQGPSGPSTQGPIPQGPSGPGPQGPVARSVSFFGPGTQGPTGPSPEGPITQGPSVPGPQGSVVSSVSFFGPGSQGPSGPSTQGPIPQGTSGTGPQGPAVSSVSFFGPGTRGPSGPSPQGTIPQGPSGPEPQGPAASSVSFFRPGTQGPSGPSPQGPIPQGPSVPGPQGSAVSSVSFFIPGSQGPSGPSTQGPIPQGPSSPGTQGPAVRSVSFESGSQGPYGPSPQGPIPQRPSGPGSQVPTESSVSFEPGSQGPYESSPQGPIPQHPSGPGPQGPPASSVSFFGPGTQGPSGPSPQGPIPQGTSGTGPQGPAVSSVSFFGPGPQGPSGAIPQGPSGPGTQVPTESSVSFEPGSQGPYESSPQGPIPQRPSGPGPQGPPASSISFFGPGTQGPSGPSPQGPIPQGPSGTGPQGPAVSTVSFFGPGPQEPSGSIPLGPSGPGPQGPPASSVSFFGPGTQGPSGPSPQGPIPQGSSVPGSQGSAVSSVSFFGPGSEGPSGPSTQGPIPQGTSLTGPQGPAVSSVSFFGPGPLGPSGPITQRPTSQGPSGPVPQGPAVSFVSFFAPGPQGPSGPSLQGPIPQGTSGPGPQGSSLSSVSFFGPGSQGPSGPSSHGPIPQGPFGPGSQGPTVSTVSFFGLGPQGPFGPSPQGSIPQGPSIPGPQVPAVSSVSFFEPGPQGLSGPSPQGPIPQGPSIPGPQVPAVSSVSFFGPGSQGPSGPSTQGSIPQGTSGTGPQGPAVTSVSFEPGSQRPYGPSPQGPIPQGPSGPGPHGPAASSVSFFGPGTQGPSGPSPQGPIPQGPFGPGSQGPTVSSVSFFGPGSQGPSGPSTQGPIPQGTSGTGPQGPAVSSVSFFGPGTRGPSGPSPQDPIPQGPSIAGPQVPAVSSVSFFGPGSQGPSEPSPQGPISRGPSGPGPQGPAVSSVSFFGPRPRGPSGPSAQGPIPQGPSFPGPQRPAVSSVSFEPGSQGPYGPSPPGSILQGPSGAGPQGPAASSVSFFGPGTQGPSGPSPQGPIPQGPFGPGSQGPTVSSVSFFGSRPQGPSGPSPVGPIPQGLSGPLPQAPAVSSLSMYDAVDQGLSRFASQVPLPLGQSARTPQRSSASFGLGLSGVSVLHQSPVVSSAASRLSSPAAISRISSAMSSLAASGPRNPIGLSKVLGSISSQIKASNPGLSECETFAQTLLEIVSALIQILNSSNIGQVNLRPTGQSNAVVNQAVLQTLG

>Atri_Masp2.3

MCFVVLCTQALLVVEAARSPWESTQLAESFLSSFLRAIARSGAFSSNQLDDMSTIGETLTISIEKLASSSKTSKAKLQALDMAFASSMAEIAVAEEGGLSINEKTDAIGNALETAFLETTGRVNAQFVSEIKSLIFLIAQASSNEISSALTSGASGYGTPGQGGTYASVSLAGSYSQTPQRPQTTGQGPSGPASQGPVFSSVSTYGPGPIGPSPHGPTPQVPSGPAPQGPVVSSVSFFEPGPQGPVGPSLQAPLPQGPSGPEPQGSAVSSVSFFGSGPQGPSGPSPQGPTPQGPSGPGPQGPAVSSVSLFGQGPVVPGPQAPLPEGPSGPVPQGSAVSSVSFFGPGPLGPSGPSPQGPTPQGPSGPGPQGPAVSAVLVFGPRPQRPAGPGPQAPSSQGSSELGPQAPGPTANVLTSYGPGLQRPLKVPTPQSPSGPGPQGPSVSSMSVFAPVPQRQSDAGLQEETPQGPSASGSPGPSVSSVSFFRPGPQGPSGPNQQGPTPQGPSGPGPQGPSVSSVSFFGPGPQGPSGPNQQGPPPQGPSGTGPPGPSVSSASLFGQQGPAAPGPQGPTPQGRSGPGPQGPSVSSVSFFGPGPQGPSGSNQQEPIPQGPSGSGPQGPFSSVSFFGPGPQGPSGPNQQGPSAPGPSDAGPQGPTVSSSSFFGPQGPSGPGPQGPTPQGPSTPGPQGPSVTFTSFFSPQGPSGPGPQGPIPQEPSGPGPQGPSVSSVSVFAPVPQRPSAPGLQGPNPQGPSASGSQGPSVSSVSFFGPGPQGPSGPSPKGPTPQGPSIPGPQGPSVSFTSFFGPQGPSGPGPQGPTPQGPSGPGPQGPSVSSVSFFGPGPQGPSGPNQQGPHPQGPSAPGPQGPSVSSVSFFGPGPQGPSGSNQQGPILQGPSGPGPQGPSVSSVSFFGPGPQGPSGPNQQGPSAPGPSDAGPQGPTVSSSSLFGPQGPSGPGPQGPTPQGPSGPGPQGPSVSSVSVFAPVPQGPSGPNQQGPTPQGPSGPGPQRPSVSSVSFFGPGPQGPSGPNQQGPTPQGPSGPGPQGPSVSSVSFFAPVPQRPSAPGLQGPTPQGPSAPGPQGPSVSSVSFFGPQGPSGPGPQGPTPQGPSGPGPQGPSVSSVSFFGPGPQGPSGPNQQGPTPQGPSGPGPQGPSVSSVSFFGPGPQGPSEPNQLGPSAQGPSDAGPQGPTVSSSSFFGPQGPSGPGPQGPTPQGPSGPGPQGPSVSSVSVFAPVPQRPSDAGLQGATPQGPSASGSQGPSVSSVSFFRPGPQGPSGPNQQGPTPQGPSGPGPQGPSVSSVSFFGPGPQGPSVPNQQGPSAPGPSGPGPQGPSVGSVSVFAPVPQRPSALCLQGPTPQGPSGPGPQGPSVSSVSFFRPGPQGPSGPNQQGPSAPGPSGAGPLGSSVSSASLFGPQGLSGPGPQAPTPQGPSGPGPQGPSVSSTFFFGPVPRRPSESSPQGPFSEGQSGTILQVPTLSSVSMNGPIQQGPSGFAPQAPLFLGQSPRTLQSPSKSYGFGFSGATLLRQSPVVSSAVSQLSSPAATSRISSAMSALAASGPRNPSSLSKVLRIILSQIKSSNPGLSECEVLGQGLLEIVSALIQILNSSNIGQVNLSATSQSAKIVSQAVFQILA

>Aaur_Masp2.4

MNLSIRLSLFSFMIFIMQIKFGVAQVATPWGSTQLAEGFIINFLRIIARSGAFSPNQLYEMSSIPKTLTTAMEKFNSSNTSLKIKFQALNMVFASAMAEIIAEGGSTNIELKIGAIESALNSAYKETMGVVNAQFVNDIKSLIYTLAHTAANGVSTASFAKVEDYRQDSLKQQETSISDKIIRINDSILQRETRPELELSNLESSLAPNVPGGYVSKLPFMASLQRPSLQNPYEQQIKGPPVTLNTKRNLQGTPGFEPQESSPPGITSVQDQKGLGGYGERLTTSTATDRNLISAVPLSSRFEVNGLVQQGPGDYKLIKEDQSNQTKSRTIDEESGNDGLIASRNYGVGKFGVGLQEISELEGKKSEIQQHSRAGGQEFDGKKTAALFTIDKEEPTRDSKGLIEQGSLGSREYQQNDLQSFVAVNQRSEGYGPNYELDAATLVSDRVRSFKSVPNTGKSQESQGPTRKDTEGSNGYGPRVIATVVTDNYESGTGSRRSRPEEPDVQGQFRKENQFEREVQGQGPSRQKFNIREPYETEYQETNRLEGQRSDANLPVTAGKYESSGGTSLFEQGLNIRKTSKVGNLTANSGLNDGEHKMAATVSTAVAYESTNNTKGLHGLVSNGSTSELRKSYASEFAAAVSGFDTERPIRASRISNSDDNSEISSIASSLVPGIAIKSPAVANSSAPQVSIANQSLSNCNVLIQTLLDIISALVHLLGSCNIRQFNDVASTLR

>Aarg_Masp2.4

MRMIRCFGSEPNKMNLSVRISFFFFMVIITQIKFGAGQVTTPWGSTQLAEDFITNFLRIIARGSAFSSKHLDEMSSIRKTLATAMGKFSSGNTSSKIKFQALNMVFASTMAEITAEGGSANIELKIGEIENALNLAYIETMGEVNEQFVDDIKNLIYTLAHTTAIRVSTSAFAKIGDYRQDSPEQQETSTSDKINRINDSILQREPEPGQELPNLESSLAPNASTGDVSTLPFTTRIQRPSLQAPYEQQTKESPINLNTKRNLKDLPGSELQGSSPRVITSVLDQNGSGGYGARIDMSTDTEGSLSSAGPLSSRFEVNGLVKQGPGEYEHFNEEQSNQKIGKIAVGLQGKSQVEGKGSEIQQPSRAVDQEAGGKKAAASFTIDKVEPNRDAKGLIERGSLGYREYQPNDLQSFVAVNQRSEGYGPNYELDAATSVSDRVRSFKSVPNTQEIQGPTNRNTEGSDGYGSKEIGTIVTDSYDSGSESRRSAPEEQKIQEPFRNGQYEREKQGQVPSGQRFNNRESYETEYQETNRLEDEKSDFNLPVTDGKYESGGVASSFEQGLGIRKTSKVGNLTSNSGLNDGEHRTAATVARAMIGYESTNDTKELHGFINNGPKSDGRDSYASGFAAAVSGFNADRPIRSSKISNPDDNSEISPIAPSLVPGIPIKSPAVTNSSAPQVSIANTTLSDCNVLIQTLLDIVSTLVHLLGLCKIGQMDRSDDVASTRYASMTGQSAFKPIS

>Atri_Masp2.4

MTRCFGSELNKMNLLVRLSILSFMVFFMQIKFGVGQIATPWGSTQLAEDFIANFLRIIASSTAFSSNQLDEMSSIRKTLTTAMENFSSSNISSKIKFQALNMVFASAMAEITAEGGIANIQLKIDAIESALHSAYVDTMGEVNVQFVSDIKSLIYTLAHTAANGVSTESFSKVGEYRQGSLEQQESPTSDKIVRKNNSTPQRELRPEQELSNLESSLSLNVPDDYVSILPFMTRLKRPSLQTPYEQQAKESSVTLNTKKNLQGPPKFKPQESSQKGITSVQDQKGIGDYGASVFMPPEGSLMSEHIKEEPSNQERSRIIDKEFDGDIVIASRNDGVDKIAVGLQGTSVLEGKGLEIQQTSKTGAQEYGGKKTAASFTIDKDESSRDAQGLIERGFLGSRDYQQKYLQSSVTINPRSEGVEPNYEQDAATPTSDRMLNFKSVPSTGEPQESQRLIGKDTVGSNGYGSRVVATVVTDNYESGTGSKRSRPEEQDVHGPFRKENKYEREMLKKVPSRQRFDIREPYEAEYQEANHLEGQRSDVNLPVTAGKYESGGGVSPFGLGSSIQKASEVENLTENSGLNVGEHRTGAIGATTAVRYESTNDTKGLHGLGSNGPKSEERNSYASGFEAAVNGFDMDRPIRVSRISNPDGNSEISSIASSLASGILFKSPAIANSSAPQVNIANPSLSNCNVLIQQLLDIISAFMHLLGSLNIGQFHDVSSTLYASVTGQSETKPIS

>Aaur_Masp6

MNWKNSWLPLLLLATLCINISMAQDVVESPWSTTEKADLFIKYFIETISRSPAFTREQIDDMSSIGDTLIASLDNMSKSGKSSAKILQALNLAFASSMAEIAVVEQGGQSIDIKTDAIIDALNEAFIKTGRRVNNQFLSEIRQLIIMFSRNSMNDITSGNTITSSGFAAGDYVSSFPTTAVVTSDGYTAAQSPYQISYTLSSTGDNRQQNNGIGQGVSSATTSSTQQYGLSQTSNGISSFATGDSQGYGQELSGYPDQQGYSSSSSAIAISLGYSQNDNGPTTGATGIGSGAGQGQGGYGGQLGGPGGSSSASASTAVQSYRPGLGQQAGESGSSSSATSSVDSQVFSAGQSGYSGQTDYSSSSIAISLGYGSGSDATGTGLGAGQGKGGYDGQLGGPDGSTLASASTAGQSYGPGLGQQAGGIGSSSSDTSSVDSQGYGHSRYSGQQGYSSSSIAISLGYGQNGYGPGSGATGTGSGIDQGQGRYDGQLGGPVGSSSASASSAGQSYGPGLGQQAGAGESSSSATSSADSQDYGPGQSGYSGQQGYSSSSIVISLGYGQNGYGSGSGATGTGSGIGQGQGGYDGQLGGPVVSSSASASSAGQSYGPGLGQQASGIGSSSSATSSVDSQVYGAGQSGYSGQPGYSSSSIAISLGYGQNGYGPGSGVTGTGSGRDQGQGGYDGQLGGPDGSSSASASSAGQSYGPGLGQQAGGIGSSSSATSSVDSQVYGAGQSGYSGQPGYSSSSIAISLGYGQNGYGPGSGATGTGSGRDQGQGGYDGQLGGPYGSTPASASTAGQSYGPGLGQQAGGVGSSSSDTSSVDSQGYGDSGYSGQQGYSSSSIAISLGYGQNGYGPSSGATGTGSRIGQGQGGYDGQLGRPGGSSSASASSAGQSYWPGLGQQAGGIGSSSSATSSVDSQVYGAGQSGYSGQPGYSSSSIAISLGYGQNGYGPGSGATGTGSGIGQGQGGYDGQLGGPDGSSSASASSAGQSYWPGLGQQAGGIGSSSSATSSVDSQVYGAGQSGYSGQPGYSSSSIAISLGYGQNGYGPGSGATGTGSGIGQGQGGYDGQLGGPDGSSSASASSAGQSYGPGLGQQAGGIGSSSSATSSVDSQVYGAGQSGYSGQPGYSSSSIAISLGYGQNGYGPGSGATGTGSGIGQGQGGYDGQLGGPGGSSSASASSAGQSYGPGLGQQAGAGGSSSSDTSSVDTQDYGAGQSGYSGQPGYPSSSIAISLGYGQNEYGPGSGATGTGSGRDQGQGRYDGQLGGPDGSSSASASSAGQSYGPGLGQQAGGIGSSSSATSSVDSQGYGAGQYEYSGEQGYSSSSIAISLGYGQNGYGYSTGSDFNQGQYDSSLNGQSASSAAILDSSPGQGQLKAIGSSPSASAATSSSQLYGPGQSSYFDQQRSARASSATASAAASRLSSSESSSRVSSAVSSLVSNGPTNPDALTNAISSVISQVNADNSGLSECDILVQSLLEILSALVHILGSANLGEVNYDGTSQTAQMVSQSIAQIFS

>Aarg_Masp6

MIWKNFCLPLLLVASVCVNISMAQDVVESPWTTAENADLFIKYFIEAISRSPAFTREQIDDMSSIGDTLIASLDKMAKSGKSSAKILQALNMAFASSMAEIAIVEQGGQSIDIKTDAIIDALNEAFIKTSGIVNNEFLSEIRQLIIMFSRNSMNDITSGNTITSSGFAAGAYVSSYPTTSVVTSDGYTADQSSYQTSYTPSSTGANGPQNNRIGQGLSSATTSSNQGYGLSETNYGKSSFATDDSQGYGPGLSGYLDQQGYSSSSIAISLGYDRNGYGPGSGVIGTGSGTGQGQGVYSGQLGGPDGSSSVSEGQSYGPVLGQQVRSSGSSSTATSSVDTQGYDASQSGYSEQGYSSSSFAISLSYGQNGYDSGSAATGTDSGAIQGQGGYGGQLGGSDDSSSVSAGQSYGPGQIGPIGSSSSATSSADSQRYSAEQSGYSGQQGYSSSSIAISLGYDQNGYGPSSGAIGTGSGAGQGQEGYSGQLGKPNFSSSISSSSAGQSYGPELRQQVGSSGSSSSATSSVDTQGYEASRSGYSELQGYSSSSIAISLGYGQNGYGPGSGATGTGRGAGQGQVVYGGQLGGPGDASSVSAAQSYGPRLEQQIGSSGASSSETSSVDTQGYGAGYSGQQGYSSSSIAISLGYSPGSGAGQGVYGGQLGEPDGSSSSSAGQSYRPGQVGPSSSSSSATSSVDSQSYSAEQSGYSGQQGYSSSSIPISLGYGQNGYGPGSGGTGTGAGQGQGVYAEQLGGSDDSSSVSAGQSYGPGLGQQIGSSGASSSETPSIDTQDYGTGYSGQQGYSSSSIAISLGYSPGSGGGQGVYGGQLGGPGGSSSSSAGQSYGPGQVGPSSSSSSATSSVDSQGYSAEQSGYSGQQGYSSSSIAISLGYGQNGYGSGLGGIGTGAGEGQGVYAGQLGGSDDSSSVSAGQSYGPVLGQQIGSSGASSSETPSVDTQDYGTGYSGQQGYSSSSIAISLGYSPGSGGGQGVYGGQLGGPDGSSSSSAGQSYGPGQVGPSSSSSSATSSVYSQGYSAEQSGYSGQQGYSSSSIAISLGYGENGYGSGSGAIGSGSGAGQGQEGYSGQLGKPNFSSLISSSSAGRSYGPELGQQVGSSVSSSSAKSSVDSVGYGSGQSEYSGQQGYSSSSISISLGPSQNGYGYNTGSEFSQGQYYSQDTSSLAIPDSSSGQQQLNGIGSSLSTSATTSSSQLYGPGQSSYFDQQRSAGASAGTASVAASRLSSSESSSRVSSAVSSLVLNGPTNPDALTNAISSVISQVNTSNSDLSECDVLVQSLLEILSALVHILGFATIGEVNYDGTSQTAQMVGQSLAQIFS

>Atri_Masp6

MNWRNSCIPLLLLATLCINVSMAQDVAESPWSTSEKADLFIKYFIGDISRSPAFTREQIDDMSSIGETLIASLDKMATSGKSSANILQALNMAFASSMAEIAIVEQGGQSIDIKTDAIIDALNEAFIKTSGLVNNEFLREIRQLIIMFSRNSTNEITSGNSITSSGFGTADYGSSFPTIAVVTSDGYAAGQSLDQTSYTLSATGTNRLQNNRIGQGVSSNTASITQEYGQTGNSISSFATGDVQGYGKVVSQYPDQQGYSSSSSAIAISLGYSQNGYGSTTGATGSLSGAGQGLGGYGGQLGGPDDSSSTSASSVGQSYGPGTGQQAGASGSSSSAISPVDSQSYGAGQSGFSGQQGYSSSSIAISLGYGQNGYGPDSSSRAGQGQGGYGGQSGGPDDSSTSASSVGQSYGPGTGQQDGTSGSSSSVISSIDSQSYGAGQSGFSGQQGYSSSSIAISLGYGQNGYGPDSSSGAGQGQGGYGGQLGGPDDSSSTSASSVGQSYGPGTGQQAGASGSSSSAISPVDSQSYGAGQSGFSGQQGYSSSSIAISLGYGQNGYGPDSGSGAGQGQGGYGGQLGGPDDSSSTTASSVGQSYGPGTGQQAGASGSSYSESSPVNSESYGAGQSEFSGQQGYSSSSIAISLGYGQNRYGPDSSSGAGQGQGGYGGQLGGPDDSSSTSASSVGQSYGPGTEQQAGASGSSSSAISPVDSQSYGAGQSGFSGQQRYLSSSIAISLGYGQNGYGPDSSSRAGQGQGGYGGQLGGPDDSSTSASSVGQSYGPGTGQQDGTSGSSSSVISPVDSQSYGAGQSGFSGQQGYSSSSIAISLGYGQNGYGPDSGSGAGQGQGGYGGQLGGPDDSSSTTASSVGQSYGPGTGQEAGASGSSYSESSPVNSESYGAGQSEFSGQQGYSSSSIAISLGYGQNRYGPDSSSGAGQGQGGYGEQLGGPDDSSTSASSVGQSYGPGTGQQAGASGSSSSAISPVDSQSYGVEQSGFSGQQGYSSSSIAISLGYGQNGYGPYSSSGASQGQGGYDGQLGGPNDSSSTSASSAGQSYGPGTRQEVGRSGSSSSSTSTVDYQGYSTGQSGYFGEQGYSSSLIAVSLGYGQNGYGYSTGPEFNQGQYDSSLNGQGVSSVAIFDYSPGQGPFKGVESSSFGSAETYSSQLYGPGQSNYFDQQRNGGASSTTASAAASRLSSPESSSRVSSAVSSLLSKGSTNPEALASAISSVISQVNISNSDFSECDVIVQSLLEILSALVHILGSATIEEVNYDGTSQTAQMVSQSIAQIFS
