# Supplementary material for: Rapid molecular diversification and homogenization of clustered major ampullate silk genes in Argiope garden spiders
Source: PLoS Genet. 2022 Dec 12;18(12):e1010537. doi: 10.1371/journal.pgen.1010537 (PMC9779670; doi:10.1371/journal.pgen.1010537)
Supplement: S2 File — Poly-A containing Argiope MaSp proteins broken down by poly-A units. In most genes, ensemble repeats are evident as a repeating set of poly-A units of stereotypical size. Linker regions in MaSp3 genes presented as lowercase residues. (DOCX) [file pgen.1010537.s025.docx]

>Aarg_Masp1

MIWTTRLALSILLVICSQSIFALGQSPWQSASMAESFMTSFSNALGQSQAFTDEQMDDIDTIAASIKMGVDKMERSGKTSQNKLQAMNMAFASAVAEIAISEGGGQSAQVKTNAIADALASAFLQTTGVVNGQFINEIRGLISMFAQANSISSSSASASASAGGAGGYGSQASGAAAATNGGYGQGSSQGSQAYGAGAPGAGPKIQSTQGQGQNSYQYSISVRSQSGSQGTIGGQQGGQGGYSSQGAGGADQGGYGGQGRAGAAAAAAAA

GGAGQGYGSGLGSQGGTGQGGANAAAAAAA

GGQGGQGGYGGLGSQGAGGAGQGGYGAGLGGSGAGAAAAAAAA

GGAGGAGRGYGSGLGGQGGAGQGGAAAAAAAA

GGQGGQGGYGGLGSQGAGGAGQGAGAAAAAA

GGAGGAGRGYGSGLGGQGGAGQGGAAAAAAAA

GGQGGQGGYGGLGSQGAGGAGQGGYGAGLGGSGAGAAAAAAAA

GGAGGAGRGYGSGLGGQGGAGQGGAAAAAAAA

GGQGGQGGYGGLGSQGAGGAGQGAGAAAAAA

GGAGGAGRGYGSGLGGQGGAGQGAASAAAA

GGQGGQGGYGGLGSQGAGGAGQGGYGAGLGGSGSGAAAAAAAA

GGAGGAGRGYGSGLGGQGGAGQGGAAAAAAAA

GGQGGQGGYGGLGSQGAGGAGQGAGAAAAAA

GGAGGAGRGYGSGLGGQGGAGQGAASAAAA

GGQGGQGGYGGLGSQGAGGAGQGGYGAGLGGSGSGAAAAAAAA

GGAGGAGRGYGSGLGGQGGAGQGGAASAAAAA

GGQGGQGGYGGLGSQGAGGAGQGAGAAAAAAAA

GGAGGAGRGYGSGLGSQGGAGQGAASAAAA

GGQGGQGGYGGLGSQGAGGAGQGGYGAGLGGSGSGSGAAAAAAAA

GGAGGAGRGYGSGLGSQGGAGQGGAAAAAAAAA

GGQGGQGGYGGLGSQGAGGAGQGGYGAGLGGSGAGAAAAAAAA

GGAGGAGRGYGSGLGGQGGAGQGGAAGAAAA

GGQGGQGGYGGLGSQGAGGAGQGAGAAAAAA

GGAGGAGRGYGSGLGGQGGAGQGAASAAAA

GGQGGQGGYGGLGSQGAGGAGQGGYGAGLGGSGSGSGAAAAAAAA

GGAGGAGRGYGSGLGGQGGAGQGGAAAAAAAA

GGQGGQGGYGGLGSQGAGGAGQGAGAAAATA

GGAGGAGRGYGSGLGGQGGAGQGGAAAAAAAA

GGQGGQGGYGGLGSQGAGGAGQGAGAAAAAA

GGAGGAGRGYGSGLGGQGGAGQGAASAAAA

GGQGGQGGYGGLGSQGAGGAGQGGYGAGLGGSGSGAAAAAAAA

GGAGGAGRGYGSGLGGQGGAGQGGAAAAAAAA

GGQGGQGGYGGLGSQGAGGAGQGAGAAAAAA

GGAGGAGRGYGSGLGGQGGAGQGAASAAAA

GGQGGQGGYGGLGSQGAGGAGQGGYGAGLGGSGSGAAAAAAAA

GGAGGAGRGYGSGLGGQGGAGQGGAAAAAAAA

GGQGGQGGYGGLGSQGAGGAGQGAGAAAAAAAA

GGAGGAGRGYGSGLGGQGGAGQGAASAAAA

GGQGGQGGYGGLGSQGAGGAGQGGYGAGLGGSGSGAAAAAAAA

GGAGGAGRGYGSGLGGQGGAGQGGAAAAAAAA

GGQGGQGGYGGLGSQGAGGAGQGAGAAAAAAA

GGAGGAGRGYGSGLGGQGGAGQGAASAAAA

GGQGGQGGYGGLGSQGAGGAGQGGYGAGLGGSGAGAAAAAAAA

GGAGGAGRGYGSGLGGQGGAGQGGAAAAAAAA

GGQGGQGGYGGLGSQGAGGAGQGAGAAAAAA

GGAGGAGRGYGSGLGGQGGAGQGAASAAAA

GGQGGQGGYGGLGSQGAGGAGQGGYGAGLGGSGAGAAAAAAAA

GGAGGAGRGYGSGLGGQGGAGQGGAAAAAAAA

GGQGGQGGYGGLGSQGAGGAGQGAGAAAAAA

GGAGGAGRGYGAGLGGSGAGAAAAAAAA

GGAGGAGRGYGSGLGGQGGAGQGAASAAAA

GGQGGQGGYGGLGSQGAGGAGQGGYGAGLGGSGAGAAAAAAAA

GGAGGAGRGYGSGLGGQGGAGQGGAAAAAAAA

GGQGGQGGYGGLGSQGAGGAGQGAGAAAAAA

GGAGGAGRGYGSGLGGQGGAGQGAASAAAA

GGQGGQGGYGGLGSQGAGGAGQGGYGAGLGGSGAGAAAAAAAA

GGAGGAGRGYGSGLGGQGGAGQGGAAAAAAAA

GGQGGQGGYGGLGSQGAGGAGQGAGAAAAAAA

GGAGGAGRGYGSGLGGQGGAGQGAASAAAA

GGQGGQGGYGGLGSQGAGGAGQGGYGAGLGGSGAGAAAAAAAA

GGAGGAGRGYGSGLGGQGGAGQGGAAAAAAAA

GGQGGQGGYGGLGSQGAGGAGQGAGAAAAAA

GGAGGAGRGYGSGLGGQGGAGQGAASAAAA

GGQGGQGGYGGLGSQGAGGAGQGGYGAGLGGSGAGAAAAAAAA

GGAGGAGRGYGSGLGGQGGAGQGGAAAAAAAA

GGQGGQGGYGGLGSQGAGGAGQGAGAAAAAA

GGAGGAGRGYGSGLGGQGGAGQGAASAAAA

GGQGGQGGYGGLGSQGAGGAGQGGYGAGLGGSGAGAAAAAAAA

GGAGGAGRGYGSGLGGQGGAGQGGAAAAAAAA

GGQGGQGGYGGLGSQGAGGAGQGAGAAAAAA

GGAGGAGRGYGSGLGGQGGAGQGAASAAAA

GGQGGQGGYGGLGSQGAGGAGQGGYGAGLGGSGAGAAAAAAAA

GGAGGAGRGYGSGLGGQGGAGQGGAAAAAAAA

GGQGGQGGYGGLGSQGAGGAGQGAGAAAAAA

GGAGGAGRGYGSGLGGQGGAGQGAASAAAA

GGQGGQGGYGGLGSQGAGGAGQGGYGAGLGGSGAGAAAAAAAA

GGAGGAGRGYGSGLGGQGGAGQGGAAAAAAAA

GGQGGQGGYGGLGSQGAGGAGQGAGAAAAAA

GGAGGAGRGYGSGLGGQGGAGQGAASAAAA

GGQGGQGGYGGLGSQGAGGAGQGGYGAGLGGSGAGAAAAAAAA

GGAGGAGRGYGSGLGGQGGAGQGGAAAAAAAA

GGQGGQGGYGGLGSQGAGGAGQGAGAAAAAA

GGAGGAGRGYGSGLGGQGGAGQGAASAAAA

GGQGGQGGYGGLGSQGAGGAGQGGYGAGLGGSGAGAAAAAAAA

GGAGGAGRGYGSGLGGQGGAGQGGAAAAAAAA

GGQGGQGGYGGLGSQGAGGAGQGAGAAAAAA

GGAGGAGRGYGSGLGGQGGAGQGAASAAAA

GGQGGQGGYGGLGSQGAGGAGQGGYGAGLXGSGAGAAAAAAAA

GGAGGAGRGYGSGLGGQGGAGQGGAAAAAAAA

GGQGGQGGYGGLGSQGAGGAGQGAGAAAAAA

GGAGGAGRGYGSGLGGQGGAGQGAASAAAA

GGQGGQGGYGGLGSQGAGGAGQGGYGAGLXGSGAGAAAAAAAA

GGAGGAGRGYGSGLGGQGGAGQGGAAAAAAAA

GGQGGQGGYGGLGSQGAGGAGQGAGAAAAAA

GGAGGAGRGYGSGLGGQGGAGQGAAAAAAA

GGQGGQGGYGGLGSQGAGGAGQGGYGAGLGGSGAGAAAAAAAA

GGAGGAGRGYGSGLGGQGGAGQGGAAAAAAAA

GGQGGQGGYGGLGSQGAGGAGQGAGAAAAAAAA

GGAGGAGRGYGSGLGDXGGAGQGGGAAAAA

GGQGGQGGYGGLGSQGAGQGGYGSGSYSGQQSGAASVAAASAAASRLSSPGAASRVSSAVTSLVSSGGPTNGAALSNTISNVVSQISASNPGLSGCDVLVQALLEIVSALVHILGSANIGQVNSNSAGRSASLVGQSVYQALS

>Aaur_Masp1a

MNWTTRLALSVLVVICSQSIFALGQSPWQSASMAESFMTYFSAALGQSGAFTNEQMDDIDTIATSIKMGVDKMERSGKTSQNKLQAMNMAFASAVAEIAIAEGGGQSAQVKTNAIADALASAFLQTTGVVNRQFINEIRGLISMFAQANSISSSSGYASASAEAAAGSAGGAGQGYGAGLGGQGGAGQGGAAAAAAAA

GGQGGQGGYGGLGSQGAGQGGYGAGQGGAGAAAAAAAA

GGAGGAGRGGLGAGGGGQGYGSGLGGQGGAGQGGAAAAAAAA

GGQGGQGGYGGLGSQGAGQGGYGAGQGGAGAAAAAAAA

GGAGGAGRGGLGAGGAGQGYGSGLGGQGGAGQGGAAAAAAAA

GGQGGQGGYGGLGSQGAGQGGAGAAAAAAAA

GGAGGAGRGGLGAGGAGQGYGSGLGGQGGAGGGAAAAAAAA

GGQGGQGGYGGLGSQGASQGGAGRGAAAAAAAA

GGQGGQGGYGGLGSQGAGQGGYGAGQGGAGAAAAAAAA

GGTGGAGRGGLGAGGAGQGYGSGLGGQGGAGQGGAAAAAAAA

GGQGGLGGYGGLGSQGAGQGGYGAGQGGAGAAAAAAAA

GGAGGAGRGGLGAGGAGQGYGSGLGGQGGAGQGGAAAAAAAA

GSQGGQGGYGGLGSQGAGQGGYGAGQGGAGAAAAAAAA

GGAGRGGLGAGGAGQGYGSGLGGQGGAGGGAAAAAAAA

GGQGGQGGYGGLGSQSAGQVGAGRGAAAAAAAA

GGQGGQGGYGGLGSQGAGQGGYGAGQGGAGAAAAAAAA

GGAGGAGRGGLGAGGAGQGYGSGLGGQGGAGQGGAAAAAAAA

GGQGGQGGYGGLGSQGAGQGGYGAGQRGAGAAAAAAAA

GGAGGAGRGGLGAGGAGQGYGSGLGGQGGAGQGGAAAAAAAA

GDQGGQGGYGGLGSQGAGPGGYGAGQGGAGAAAAAAAA

GGAGRGGLGAGGAGQGYGSGLGGQGGAGQGGAAAAAAAS

GGQGGQGGYGGLGSQGAGQGGYGAGAAAAAAAA

GGAGRGGLGAGGAGQGYGSGLGGQGGARGGAAAAAAAAS

GQGGQGGYGGLGSQGAGQGGAGRGASAAAAAA

GGQGGQGGYGGLGSQGAGQGGYGAGQGGAGAAAAAAAD

GGSGGAGRGGLGAGGAGRYGSGLGGQGGDGQGGAAAAAAAAA

GGQSGQGGYGGLGSQGAGQGGYGAGQGGAGAAAAAAAA

GGAGGAGRGGLGAGGAGQGYGSRLGGQGRAGQGGAAAAAAAA

GGQGGQGGYGGLGSQGAGQGGYGAGQGGAGAAAAAAAA

GAAGGAGRGGLGAGGAGQGYGSGLGGQGGAGQGGAAAAAAAA

GGQGGQGGYGGLGSQGAGQGGYGAGQGGAGAAAAAAAA

GGAGGAGRGGLGAGGAGRYGSGLGGQGGDGQGGAAAAAAAAA

GGQGGYGGLGSQGAGQGGAGRGAAAAAAAA

GGQGGQGGYGGLGSQGAGQGGYGAGQGGAGAAAAAAAA

GGAGGAGRGGLGAGGAGQGYGSGLGGQGGAGGGAAAAAAAA

GGQGGQGGYGGLGSQGAGQGGAGRGASAAAAAA

GGQGGQGGYGRLGSQGAGQGGYGAGQGGAGAAAAAA

GGAGGAGRGGLGAGGAGRYGSGLGGQGGDGQGGAAAAAAAAA

GGQGGYGGLGSQGVGQGGAGRGAAAAAAAA

GGQGGQGGYGGLGSQGAGQGGYGAGQGGAGAAAAAAAA

GGAGGAGRGGLGAGGAGRYGSGLGGQGGAGQGGAAAAAAAA

GGQGGQGGYGGLGSQGAGQGGYGAGAAAAAAAA

GGAGGAGRGGLGAGGAGQGYGSGLGGQGGAGQGGAAAAAAAA

GGQGGQGGYGGLGSQGAGQGGYGAGQGGAGAAAAAAAA

GGAGGAGRGGLGAGGAGRYGSGLGGQGGDGQGGAAAAAAAAA

GGQGGQGGYGGLGSQGAGQGGAGRGAAAAAAAA

GGQGGQGGYGGLGSQGAGQGGYGAGQGGAGAAAAAAAA

GGAGGAGRGGIGAGGAGRYGSGLGGQGGAGQGGAAAAAAAA

GGQGGQGGYGGLGSQGAGQGGYGAGAAAAAAAA

GGAGGAGRGGLGAGGAGQGYGSGLGGQGGAGQGGAAAAAAAA

GGQGGQGGYGGLGSQGAGQGGYGAGQGGAGAAAAAAAA

GGAGGAGRGGLGAGGAGQGYGSGLGGQGGAGQGGAAAAAAAAA

GGQGGQGGYGGLGSQGAGQGGYGAGQGGAGAAAAA

GGAGGAGRGGLGAGGAGQGYGSGLGGQGGAGQGGAAAAAAAA

GGQGGQGGYGGLGSEGAGQGGYGAGQGGAGAAAAAAAA

GGAGGAGRGGLGAGGAGQGYGSGLGGQGGAGQGGAAAAAAAA

GGQGGQGGYGGLGSQGAGQGGAGRGAAAAAAAA

GGQGGQGGYGGLGSQGAGQGGYGAGQGGAGAAAAAAAA

GGAGGAGRGGLGAGGAGQGYGSGLGGQGGAGQGGAAAAAAAA

GGQGGQGGYGGLGSQGAGQGGYGAGQGGAGAAAAAATA

GGAGGAGRGGLGAGGAGQGYGSGLGGQGGAGQGGAAAAAAAA

GGQGGQGGYGGLGSQGAGQGGYGAGAAAAAAAA

GGAGGAGRGGLGAGGAGQGYGSGLGGQGGAGQGGAAAAAAAA

GGQGGQGGYGGLGSQGAGQGGYGAGQGGAGAAAAAAAA

GGAGGAGRGGLGAGGAGRYGSGLGGQGGDGQGGAAAAAAAAA

GGQGGQGGYGGLGSQGVGRDAAAAAAAA

GGQGGQGGYGGLGSQGAGQGGYGAGQGGAGAAAAAAAA

GGAGGAGRGGLGAGGAGQGYGSGLGGQGGAGQGGAAAAAAAAA

GGQGGQGGYGGLGSQGAGQGGYGVGQRGAGAAAAAAAA

GGAGGAGRGGLGAGGAGQVYGSGLGGQGGAGQGGAATAAAAA

GGQGGQGGYGGLGSEGAGQGGYGAGQGGAGAAAAAAAA

GGAGGAGRGGLGAGGAGQGYGSGLGGQGGAGQGGAAAAAAAA

GGQGGQGGYGGLGSQGAGQGGAGRGAAAAAAAA

GGQGGQGGYGGLGSQGAVQGGAGRGAAAAAAAA

GGQGGQGGYGGLGSQGAGQGGAGRGAAAAAAAA

GGQGGQGGYGGLGSEGAGQGGAGRGAAAAAAAA

GGQGGQGGYGGLGSQGAGQGGYGAGQGGARAAAAAAAA

GGAGGAGRGGLGAGGAGQGYGSGLGGQGGAGQGGAAAAAAAAAA

GGQGGQGGYGGLGSQGAGQGGYGAGQGGAGAAAAAATA

GGAGGAGRGGLGAGGAGQGYGSGLGGQGGAGQGGAAAAAAAA

GGQGGQGGFGRFSSQEAGQGAYGGGAYSGQQGAAASVSAASAAASRLSSPGAASRVSSAVTSLVSSGGPTNPAALSNTISNVVSQISESNPGLSGCDVLVQALLELVSALVHILGSANIGQVNSSAAGQSASLVRQSVYQALS

>Aaur_Masp1b

MIWTVRFSLSLLIVICSQSIFALGQSPWQSASMAESFMTYFSAALGQSGAFTNEQMDDIDTIASSIKMGVDKMERSGKTSQNKLQAMNMAFASAVADIAIAEGGGQSAQIKTNAIADALASAFLQTTGTVNNQFINEIRGLISMFAQANSISSSSASASESTAAAAGGPGGAGQGYGSGLGGAGGVGAASAAAAA

GGLGGRGGFGGLGSQGVGGAGQGAGAAAAAAAA

GGDGGAGLRGLGAGQGYGSGLGGAGGAGAASAAAAA

GGLGGRGGFGGLGSQGASGAGQGGAGAAAAAAAA

GGDGGAGLRGLGAGQGYGSGLGGAGGAGAASAAAAA

GGLGGRGGFGGLGSQGVGGAGQGAGAAAAAAAA

GGDGGAGLRGLGAGQGYGSGLXGAGAGAASAAAAA

GGLGGRGGFGGLGSQGVGGAGQGGAGAAAAAAAA

GGDGGAGLRGLGAGQGYGSGLGGAGGAGAASAAAAA

GGVGGRGGFGGLGSQGASGAGQGGAGAASAAAAA

GGLGGRGGFGGLGSQGASGAGQGGAGAAAAAAAA

GGDGGAGLRGLGAGQGYGSGLGGAGGAGAASAAAAA

GGLGGRGGFGGLGSQGASGAGQGAGAAAAAAAA

GGDGGAGLRGLGAGQGYGSGLGGAGGAGAASAAAAA

GGLGGRGGFGGLGSQGVGGAGQGGAGAAAAAAAA

GGDGGAGLRGLGAGQGYGSGLGGAGGAGAASAAAAA

GGLGGRGGFGGLGSQGASGAGQGGAGAAAAAAAA

GGDGGAGLRGLGAGQGYGSGLGGAGGAGAASAAAAAA

GGDGGAGLRGLGAGQGYGSGLGGAGGAGAASAAAAA

GGLGGRGGFGGLGSQGVGGAGQGAGAAAAAAAA

GGDGGAGLRGLGAGQGYGSGLGGAGGAGAASAAAAA

GGLGGRGGFGGLGSQGASGAGQGGAGAAAAAAAA

GGDGGAGLRGLGAGQGYGSGLGGAGGAGAASAAAAA

GGLGGRGGFGGLGSQGASGAGQGGAGAAAAAAAA

GGDGGAGLRGLGAGQGYGSGLGGAGGAGAASAAAAA

GGLGGRGGFGGLGSQGASGAGQGGAGAAAAAAAA

GGDGGAGLRGLGAGQGYGSGLGGAGGAGAASAAAAA

GGLGGRGGFGGLGSQGASGAGQGGAGAAAAAAAA

GGDGGAGLRGLGAGQGYGSGLGGAGGAGAASAAAAA

GGLGGRGGFGGLGSQGVGGAGQGAGAAAAAAAA

GGDGGAGLRGLGAGQGYGSGLGGAGGAGAASAAAAA

GGLGGRGGFGGLGSQGVGGAGQGAGAAAAAAAA

GGDGGAGLRGLGAGQGYGSGLGGAGGAGAASAAAAA

GGLGGRGGFGGLGSQGASGAGQGGAGAAAAAAAA

GGDGGAGLRGLGAGQGYGSGLGGAGGAGAASAAAAA

GGLGGRGGFGGLGSQGASGAGQGGAGAAAAAAAA

GGDGGAGLRGLGAGQGYGSGLGGAGGAGAASAAAAA

GGLGGRGGFGGLGSQGASGAGQGAGAAAAAAAA

GGDGGAGLRGLGAGQGYGSGLGGAGGAGAASAAAAA

GGLGGRGGFGGLGSQGASGAGQGGAGAAAAAAAA

GGDGGAGLRGLGAGQGYGSGLGGAGAASAAAAA

GGLGGRGGFGGLGSQGASGAGQGGAGAAAAAAAA

GGDGGAGLRGLGAGQGYGSGLGGAGGAGAASAAAAA

GGLGGRGGFGGLGSQGASGAGQGGAGAAAAAAAA

GGDGGAGLRGLGAGQGYGSGLGGAGGAGAASAAAAA

GGLGGRGGFGGLGSQGVGGAGQGAGAAAAAAAA

GGDGGAGLRGLGAGQGYGSGLGGAGGAGAASAAAAA

GGLGGRGGFGGLGSQGASGAGQGGAGAASAAAAA

GGVGGRGGFGGLGSEGASGAGQGGAGAAAAAAAA

GGDGGAGLRGLGAGQGYGSGLGGAGGAGAASAAAAA

GGLGGRGGFGGLGSQGVGGAGQGGAGAAAAAAAA

GGDGGAGLRGLGAGQGYGSGLGGAGGAGAASAAAAAA

GGDGGAGLRGLGAGQGYGSGLGGAGGAGAASAAAAA

GGLGGRGGFGGLGSQGVGGAGQGAGAAAAAAAA

GGDGGAGLRGLGAGQGYGSGLGGAGAASAAAAA

GGLGGRGGFGGLGSQGASGAGQGGAGAAAAAAAA

GGDGGAGLRGLGAGQGYGSGLGGAGGAGAAAAAAAA

GGDGGAGLRGLGAGQGYGSGLGGAGGAGAASAAAAA

GGLGGRGGFGGLGSQGASGAGQGAGAAAAAAAA

GGDGGAGLRGLGAGQGYGSGLGGAGGAGAASAAAAA

GGLGGRGGFGGLGSQGASGAGQGGAGAAAAAAAA

GGDGGAGLRGLGAGQGYGSGLGGAGGAGAASAAAAA

GGLGGRGGFGGLGSQGASGAGQGGAGAAAAAAAA

GGDGGAGLRGLGAGQGYGSGLGGAGGAGAASAAAAA

GGLGGRGGFGGLGSQGASGAGQGGAGAAAAAAAA

GGDGGAGLRGLGAGQGYGSGLGGAGGAGAASAAAAA

GGLGGRGGFGGLGSQGVGGAGQGAGAAAAAAAA

GGDGGAGLRGLGAGQGYGSGLGGAGAASAAAAA

GGLGGRGGFGGLGSQGVGGAGQGGAGAAAAAAAA

GGDGGAGLRGLGAGQGYGSGLGGAGGAGAAGAAAAA

GGLGGRGGFGGLGSQGVGSAGQGAGAAAAAAAA

GGDGGTGLRGLGAGKGYGAGLGGAGGAGAASAAAAAA

GGDGGAGLRGLGAGQGYGSGLGGAGGAGAASAAAAA

GGLGGRGGFGGLGSQGVGGAGQGAGAAAAAAAA

GGDGSAGLRGLDAIQGYGSGLGGAGAASAAAAA

GGLGGRGGFGGLGSQGASGAGQGGAGAAAAAAAA

GGDGGAGLRGLGAGQGYGSGLGGAGGAGAASAAAAAA

GGDGGTGLRGLGAGQGYGSGLGGAGGAGAASVAAAA

GGVGGRGGFGGLGSQGASGAGQGGAGAAAAAAAA

GGDGGAGLRGLGAGKGYGSGLGGAGGAGAASAAAAAA

GGDGSAGLRGLDAIQGYGSGLGGAGAASAAAAA

GGLGGRGGFGGLGSQGASGAGQGGAAAAAAA

GGDSGAGLRGLGAGQGYGSGLGGAGGAGAASAAAAA

GGLGGRGGFGGLGSQGVGGAGKGAGAAAAAAAA

GGDGGAGLRGLGAGQGYGSGLGXAGGAGAASAAAAA

GGLGGRGGFGGLGSQGVGGAGQGAGAAAAAAAA

GGDGGAGLRGLGAGQGYGSGLGGAGGAGAASAAAAA

GGLGGRGGFGGLGSQGVGGAGQGAGAAAAAAAA

GGDGGAGLRGLGAGQGYGSGLGGAGGAGAASAAAAAA

GGDGSAGLRGLGAGQGYGSGLGGAGGAGAASAAAAA

GGLGGRGGFGGLGSQGVGSAGQGAGAAAAAAAA

GGDGGAGLRGLGAGQGYGSGLLGAGAASAAAAA

GGLGGRGGFGGLGSQGASGAGQGGAGAAAAAAAA

GGDGGAGLRGLGAGQGYGSGLGGAGGAGAASAAAAA

GGLEGRGGFGGLGSQGVGGAVQGGAGASASAAASGGYGGLGSELEVQGAYRSGAYSGQQSAAVSVAAVSAAASRLSSPNAASRISSAVTSLISGGGPTNLAALSNTFSNVVYQISVSNPGLSGCDVLVQALLELVSALVHILGSAIIGHVNSSAAGETAALVGQSVYQAFS

>Atri_Masp1

MIWTTRLALSILVVICSQSIFAQGQSPWQSASMAESFMTYFSAALGQSGAFTNEQMDDIDTIATSIKMGVDKMERSGKTSLNKLQAMNMAFASAVAEIAISEGGGQSAQVKTNAIADALASAFLQTTGVVNRQFINEIRGLISMFAQANSISSSSASASASAAGAGGYGSQGSGAAVSAGGGYGQGTSQGSQAYGAGASGAGPQTQSTQGQGQSSYQYSISVSSQGGYGGLGSQGAGQGGYGGGQGGAAAAAVAASGASGAGQGGLGAGGAGQGYGAGLGGQGGAGRGGAAAAAAAAA

GGQGGQGGYGGLGSQGAGQGGYGQGGAAAAAAAAS

GAGGAGRGGLGAGGAGQGYGAGLGGQGGAGQGGAAAAAAAAA

GGQGGQGGYGGLGSQGAGQGGYGQGGAAAAAAAAS

GAGGAGRGGLGAGGAGQGYGAGSGGQGGAGQGGAAAAAAAAA

GGXGGLGGYGGLGSQGAGQGGYGQGGAAAAAAAAS

GAGGAGRGGLGAGGAGQGYGAGSGGQGGAGQGGAAAAAAAAA

GGQGGQGGYGGLGSQGAGQGGYGQGGAAAAAAAAS

GSGGAGRGGLGAGGAGQGYGAGSGGQGGAGQGGAAAAAAA

GGQGGQGGYGGLGSQGAGQGGYGQGGAAAAAAAAS

GSGGAGRGGLGAGGAGQGYGAGLGGQGGAGQGGAAAAAAAAA

GGQGGQGGYGGLGSQGAGQGGYGQGGAAAASAAAS

GAGGAGRGGLGAGGAGQGYGAGSGGQGGAGQGGAAAAAAA

GGQGGQGGYGGLGSQGAGQGGYGQGGAAAAAAAAS

GAGGAGRGGLGAGGAGQGYGAGLGGQGGAGQGGAAAAAAAAA

GGQGGQGGYGGLGSQGAGQGGYGQGGAAAAAAAAS

GAGGAGRGGLGAGGAGQGYGAGSGGQGGAGQGGAAAAAAA

GGQGGQGGYGGLGSQGAGQGGYGQGGAAAAAAAAS

GAGGAGRGGLGAGGAGQGYGAGSGGQGGAGQGGAAAAAAAAA

GGQGGQGGYGGLGSQGAGQGGYGQGGAAAAAAAAS

GAGGAGRGGLGAGGAGQGYGAGSGGQGGAGQGGAAAAAAA

GGQGGQGGYGGLGSQGAGQGGYGQGGAAAAAAAAS

GAGGAGRGGLGAGGAGQGYGAGSGGQGGAGQGGAAAAAAAAA

GGQGGQGGYGGLGSQGAGQGGYGQGGAAAAAAAAS

GAGGAGRGGLGAGGAGQGYGAGLGGQGGAGQGGAAAAAAAAA

GGQGGQGGYGGLGSQGAGQGGYGQGGAAAAAAAAS

GAGGAGRGGLGAGGAGQGYGAGSGGQGGAGQGGAAAAAAAA

GGQGGQGGYGGLGSQGAGQGGYGQGGAAAAAAAAS

GAGGAGRGGLGAGGAGQGYGAGLGGQGGAGQGGAAAAAAAAA

GGQGGQGGYGGLGSQGAGQGGYGQGGAAAAAAAAS

GAGGAGRGGLGAGGAGQGYGAGLGGQGGAGQGGAAAAAAAAA

GGQGGQGGYGGLGSQGAGQGGYGQGGAAAAAAAAS

GAGGAGRGGLGAGGAGQGYGAGSGGQGGAGQGGAAAAAAAAA

GGQGGQGGYGGLGSQGAGQGGYGQGGAAAAAAAAS

GAGGAGRGGLGAGGAGQGYGAGSGGQGGAGQGGAAAAAAAAA

GGQGGQGGYGGLGSQGAGQGGYGQGGAAAAAAAAS

GAGGAGRGGLGAGGAGQGYGAGSGGQGGAGQGGAAAAAAA

GGQGGQGGYGGLGSQGAGQGGYGQGGAAAAAAAAS

GAGGAGRGGLGAGGAGQGYGAGLGGQGGAGQGGAAAAAAA

GGQGGQGGYGGLGSQGAGQGGYGQGGAAAAAAAAS

GAGGAGRGGLGAGGAGQGYGAGSGGQGGAGQGGAAAAAAAA

GGQGGQGGYGGLGSQGAGQGGYGQGGAAAAAAAAS

GAGGAGRGGLGAGGAGQGYGAGSGGQGGAGQGGAAAAAAAAA

GGQGGQGGYGGLGSQGAGQGGYGQGGAAAAAAAAS

GAGGAGRGGLGAGGAGQGYGAGSGGQGGAGQGGAAAAAAAAA

GGQGGQGGYGGLGSQGAGQGGYGQGGAAAAAAAAS

GAGGAGRGGLGAGGAGQGYGAGSGGQGGAGQGGAAAAAAAAA

GGQGGQGGYGGLGSQGAGQGGYGQGGAAAAAAAAS

GAGGAGRGGLGAGGAGQGYGAGSGGQGGAGQGGAAAAAAAAA

GGQGGQGGYGGLGSQGAGQGGYGQGGAAAAAAAAS

GAGGAGRGGLGAGGAGQGYGAGSGGQGGAGQGGAAAAAAAA

GGQGGQGGYGGLGSQGAGQGGYGQGGAAAAAAAAS

GAGGAGRGGLGAGGAGQGYGAGSGGQGGAGQGGAAAAAAAAA

GGQGGQGGYGGLGSQGAGQGGYGQGGAAAAAAAAS

GAGGAGRGGLGAGGAGQGYGAGSGGQGGAGQGGAAAAAAAAA

GGQGGQGGYGGLGSQGAGQGGYGQGGAAAAAAAAS

GAGGAGRGGLGAGGAGQGYGAGSGGQGGAGQGGAAAAAAAAA

GGQGGQGGYGGLGSQGAGQGGYGQGGAAAAAAAAS

GAGGAGRGGLGAGGAGQGYGAGSGGQGGAGQGGAAAAAAAAA

GGQGGQGGYGGLGSQGAGQGGYGQGGAAAAAAAAS

GAGGAGRGGLGAGGAGQGYGAGSGGQGGAGQGGAAAAAAAAA

GGQGGQGGYGGLGSQGAGQGGYGQGGAAAAAAAAS

GAGGAGRGGLGAGGAGQGYGAGSGGQGGAGQGGAAAAAAAAA

GGQGGQGGYGGLGSQGAGQGGYGQGGAAAAAAAAS

GAGGAGRGGLGAGGAGQGYGAGSGGQGGAGQGGAAAAAAA

GGQGGQGGYGGLGSQGAGQGGYGQGGAAAAAAAAS

GAGGAGRGGLGAGGAGQGYGAGLGGQGGAGQGGAAAAAAAAA

GGQGGQGGYGGLGSQGAGQGGYGQGGAAAAAAAAS

GAGGAGRGGLGAGGAGRGYGAGSGGQGGAGQGGAAAAAAAAA

GGQGGQGGYGGLGSQGAGQGGYGQGGAAAAAAAAS

GAGGAGRGGLGAGGAGQGYGAGSGGQGGAGQGGAAAAAAAAA

GGQGGQGGYGGLGSQGAGQGGYGQGGAAAAAAAAS

GAGGAGRGGLGAGGAGQGYGAGSGGQGGAGQGGAAAAAAAAA

GGQGGQGGYGGLGSQGGYGQGGAAAAAAAAS

GAGGAGRGGLGAGGAGQGYGAGSGGQGGAGQGGAAAAAAAAA

GGQGGQGGYGGLGSQGAGQGGYGQGGAAAAAAAAS

GAGGAGRGGLGAGGAGQGYGAGLGGQGGAGQGSAAAAAAAAA

GGQGGQGGYGGLGSQGAGQGGYGQGGAAAAAAAAS

GAGGAGRGGLGAGGAGQGYGAGLGGQGGAGQGGAAAAAAAAA

GGQGGQGGYGGLGSQGAGQGGYGQGGAAAAAAAAS

GAGGAGRGGLGAGGAGQGYGAGSGGQGGAGQGGAAAAAAAAA

GGKGGQGGYGGLGSQGAGQGGYGQGGAAAAAAAAS

GAGGAGRGGLGAGGAGQGYGAGSGGQGGAGQGGAAAAAAAAA

GGQGGQGGYGGLGSQGAGQGGYGQGGAAAAAAAAS

GAGGAGRGGLGAGGAGQGYGAGSGGQGGAGQGGAAAAAAAAA

GGQGGQGGYGGLGSQGAGQGGYGQGGAAAAAAAAS

GAGGAGRGGLGAGGAGQGYGAGLGGQGGAGQGGAAAAAAAAA

GGQGGQGGYGGLGSQGAGQGGYGQGGAAAAAAAAS

GAGGARRGGLGAGGAGQGYGAGLGGQGGAGQGGAAAAAAAAA

GGQAGQGGYGGLGSQGAGQGGYGQGGAAAAAAAAS

GAGGARRGGLGAGGAGQGYGAGSGGQGGAGQGGAAAASAAAA

GSQGGPGRYGGLGSQGSGQGGYGQGGAAAAAAAAS

GAGGAGRGGLGAGGAGQGYGAGSGGQGGAGQGGAAAAAAAAA

GGQGGQGGYGGLGSQGAGQGGYGQGGAAAAAAAAS

GAGGAGRGGLGAGGAGQGYGAGLGGQGGAGQGGAAAAAAA

GGKGGQGGYGGLGSQGAGQGGYGQGGAAAAAAAAS

GAGGAGQGGLGAGGAGQGYGAGSGGQGGAGQGGAAAAAAAAA

GGQGGQGGYGGLGSQGAGQGGYGQGAAAAAAAAS

GAGGAGRGGLGAGGAGQGYGARLGSQGGAGQGGAAAAAAA

GGQGGLGGYGGLGSQGSGQGGYGQGGAAATAAAAS

GAGGAGQGGLGAAGAGQGYGAGSGGQGGAGQGGAAAAAAAAA

GGQGGQGGYGGLGSQGAGQGGYGQGGVAAAAAAAS

GAGGAGRGGLGAGGAGQVYGAVSGGQGGAGQGGEAAAAAAAA

GGQGGQGGYGGLGSQGAGQGGYGQGGAAAAIAAAS

GAGGAGQGGLGAGGAGQGYGAGSGGQGGAGQGGAAAAAAAAS

GQGGQGGYGGLGSQGAGQGGYGQGGAAAAAASA

GGQGGQGGYGGLGSQGAGQGGYGGGAFSGQQGGAASVATASAAASRLSSPGAASRVSSAVTSLVSSGGPTNSAALSNTISNVVSQISSSNPGLSGCDVLVQALLEIVSALVHILGSANIGQVNSSGVGRSASIVGQSINQAFS

>Aarg_MaSp2.1a

MSCPRLVLAFLALLSTHALFASAAGATPWSSPAMADSFMTNFINGIANSRAFTGGQIDDMTTIGDTMMDSVNRLASSGKISKSKLQALNMAFASSMAEIAATEEGGMSIGAKTSAIANALRGAFLQTTGYANEQFINEITSLVSMIAQANANSVSASASASAGGGGGGGYGGSSYGPSGYGPSQQQSSASSVSVSASAAGAGPRGQAPSGPAQQGPRGYGPSGPGGASAAAAAAAS

GPRGQGPYGPAQQGPGARGPSGPSQQGPGPQGPGGYGPSGPGSASAAAAAAST

GGQGPSGQGQQGPGGYGQSGPGQQGPGGYGPSGPGSASAAAAAASA

GGQGPSGQGQQGPGGYGQSGPGQQGPGGYGPSGPGSASAAAAAASA

GGQGPSGQGQQGPGGYGQSGPGQQGPGGYGPSGPGSASAAAAAASA

GGQGPSGQGQQGPGGYGQSGQGQRGPGGYGPSGPSGAAAAAASA

GGQGPYGQGQQGPGQQGPGAGGYGPGGASAAAAAAAA

GGPGGQGPYGPGQGAGGPYGPGAQGPGSQGPGGYGPSGPGSASAAAAAAAA

GGQGPSGQGQQGPGGYGPSGPSGAAAAAAAA

GGQGPYGQGQQGPGQQGPGAGGYGPGGASAAAAAAAA

GGPGGQGPYGPGQGAGGPYGPGAQGPGSQGPGGYGPSGPGSASAAAAAAAA

GGQGPSGQGQQGPGGYGPSGPSGAAAAAAAA

GGQGPYGQGQQGPGQQGPGAGGYGPGGASAAAAAAAA

GGPGGQGPYGPGQGAGGPYGPGAQGPGSQGPGGYGPSGPGSASAAAAAAAA

GGQGPSGQGQQGPGGYGPSGPSGAAAAAAAA

GGQGPYGQGQQGPGQQGPGAGGYGPGGASAAAAAAAA

GGPGGQGPYGPGQGAGGPYGPGAQGPGSQGPGGYGPSGPGSASAAAAAAAA

GGQGPSGQGQQGPGGYGPSGPSGAAAAAAAA

GGQGPYGQGQQGPGQQGPGAGGYGPGGASAAAAAAAA

GGPGGQGPYGPGQGAGGPYGPGAQGPGSQGPGGYGPSGPGSASAAAAAAAA

GGQGPSGQGQQGPGGYGPSGPSGAAAAAAAA

GGQGPYGQGQQGPGQQGPGAGGYGPGGASAAAAAAAA

GGPGGQGPYGPGQGAGGPYGPGAQGPGSQGPGGYGPSGPGSASAAAAAAAA

GGQGPSGQGQQGPGGYGPSGPSGAAAAAAAA

GGQGPYGQGQQGPGQQGPGAGGYGPGGASAAAAAAAA

GGPGGQGPYGPGQGAGGPYGPGAQGPGSQGPGGYGPSGPGSASAAAAAAAA

GGQGPSGQGQQGPGGYGPSGPSGAAAAAAAA

GGQGPYGQGQQGPGQQGPGAGGYGPGGASAAAAAAAA

GGPGGQGPYGPGQGAGGPYGPGAQGPGSQGPGGYGPSGPGSASAAAAAAAA

GGQGPSGQGQQGPGGYGPSGPSGAAAAAAAA

GGQGPYGQGQQGPGQQGPGAGGYGPGGASAAAAAAAA

GGPGGQGPYGPGQGAGGPYGPGAQGPGSQGPGGYGPSGPGSASAAAAAAAA

GGQGPSGQGQQGPGGYGPSGPSGAAAAAAAA

GGQGPYGQGQQGPGQQGPGAGGYGPGGASAAAAAAAA

GGPGGQGPYGPGQGAGGPYGPGAQGPGSQGPGGYGPSGPGSASAAAAAAAA

GGQGPSGQGQQGPGGYGPSGPSGAAAAAAAA

GGQGPYGQGQQGPGQQGPGAGGYGPGGASAAAAAAAA

GGPGGQGPYGPGQGAGGPYGPGAQGPGSQGPGGYGPSGPGSASAAAAAAAA

GGQGPSGQGQQGPGGYGPSGPSGAAAAAAAA

GGQGPYGQGQQGPGQQGPGAGGYGPGGASAAAAAAAA

GGPGGQGPYGPGQGAGGPYGPGAQGPGSQGPGGYGPSGPGSASAAAAAAAA

GGQGPSGQGQQGPGGYGPSGPSGAAAAAAAA

GGQGPYGQGQQGPGQQGPGAGGYGPGGASAAAAAAAA

GGPGGQGPYGPGQGAGGPYGPGAQGPGSQGPGGYGPSGPGSASAAAAAAAA

GGQGPSGQGQQGPGGYGPSGPSGAAAAAAAA

GGQGPYGQGQQGPGQQGPGAGGYGPGGASAAAAAAAA

GGPGGQGPYGPGQGAGGPYGPGAQGPGSQGPGGYGPSGPGSASAAAAAAAA

GGQGPSGQGQQGPGGYGPSGPSGAAAAAAAA

GGQGPYGQGQQGPGQQGPGAGGYGPGGASAAAAAAAA

GGPGGQGPYGPGQGAGGPYGPGAQGPGSQGPGGYGPSGPGSASAAAAAAAA

GGQGPSGQGQQGPGGYGPSGPSGAAAAAAAA

GGQGPYGQGQQGPGQQGPGAGGYGPGGASAAAAAAAA

GGPGGQGPYGPGQGAGGPYGPGAQGPGSQGPGGYGPSGPGSASAAAAAAAA

GGQGPSGQGQQGPGGYGPSGPSGAAAAAAAA

GGQGPYGQGQQGPGQQGPGAGGYGPGGASAAAAAAAA

GGPGGQGPYGPGQGAGGPYGPGAQGPGSQGPGGYGPSGPGSASAAAAAAAA

GGQGPSGQGQQGPGGYGPSGPSGAAAAAAAA

GGQGPYGQGQQGPGGYGPSGPSGAAASAAAA

GGQGPYGQGQQGPGGYGPSGPSGAAAAAAAA

GGQGPYGQGQQGPGGYGPSGPASGVSASVSSAASRLSSPAASSRVSSAVSTLASSGPSNAGVVSSALSNLVSQVSANHPGLSGCDVIVQALLELVSALVHILGSSSVGQVDYNGASYSAQSLGQAVAQALG

>Aarg_MaSp2.1b

MSCPRLVLAFLALLSTHALFASAAGATPWSTPALADSFMRSFIGGISSSGAFTGGQIDDMSTISDTMTDSVNRLASSGKISKSKLQALNMAFASSMAEIAATEEGGLSIGAKTSAIADALRGAFLQTTGYSNEQFINEITSLVSMIAQANANSVSASASASSGGGGGGGYGGSSYGPSSVSSASASASAAGAGAGPAQQGSGSYGPSGPGGYGPSGSSAAAAAS

GGQGPGNYGPSGSGGAGPSGPGGYGPGSQGSSGSGNQGPGAAASAAAAAS

GPGGYGPGSQGPSGPGGYGPGSQGPGGAGGYGPGSQGPGGSGAAAAAAAASS

GPGGYGPGSQGPSGPGSQGSSGPGGASAAAAAASS

GPGGYGPGSQGPSGPGGYGPGSQGPSGPGGYGPGSSGPGGYGPGSQGPGAASAAAAAS

GPGGYGPGSQGPSGAGSQGPSGPGSQGPSGPGGASAAAAAASS

GPGGYGPGSQGPSGPGGYRPGSQGPSGPGGFGPGSSGPGGYGPGSQGPGAASAAAAAS

GPGGYGPGSQGPSGAGSQGPSGPGSQGPSGPGGASAAAAAASS

GPGGYGPGSQGPSGLGGYGPGSQGPSGPGGYGPGSSGPGGAGGYGPGSQGPGGSGAAAAAAAASS

GPGGYGPRSQGPSGPGSQGPSGPGGYGPGSSGPGGYGPGSQGPGAAPAAAAAS

GPGGYGPGSQGPGAASAAAAAS

GPGGYGPGSQGPSGPGSQGPSGPGGASAAAAAASS

GPGGYGPGSQGPSGLGGYGPGSQGPSGPGGYGPGSSGPGGAGGYGPGSQGQGGSGAAAAAAAASS

GPGGYGPRSQGPSGPGSQGPSGPGGYGPGSSGPGGYGPGSQGPGAASAAAAAS

GPGGYGPGSQGPGAASAAAAAS

GPGGYGPGSQGPSGPGSQGPSGPGGASAAAAAASS

GPGGYGPGSQGPSAPGGYGPGSQGPSGPGGYGPGSSGPGGYGPGSQGPGAASAAAAAS

GPGGYGPGSQGPSGAGSQGPSGPGSQGPSGPGGASAAAAAASS

GPGGYGPGSQGPSGLGGYGPGSQGPSGPGGYGPGSSGPGGAGGYGPGSQGPGGSGAAAAAAAASS

GPGGYGPRSQGPSGPGSQGPSGPGGASAAAAAASS

GPGGYGPGSQGSSGPGGYGPGSSGPGGYGPGSQGPGAAAAAAASS

GPGGYGPGSQGPSGPGSQGPAGPGGYGPGSSGPGGAGGYGPGSQGPGGSGAAAAAAAASS

GPGGYGPGSQGPSGPGSQGPSGPLSQGPSGPGSQGPSGPGGASAAAAAASS

GPGGYGPGSQGPSGPGGYGPGSQGPSGPGGYGPGSQGPSGPGGYGPGPSGPGAAGGYGPGSQGPGGSGAAAAAAAASS

GPGGYGPGSQGPSGPXSQGPSGPGGASAAAAAASS

GPGGYGPGSQGPSGPGGYGPGNQGPSGPGGYGPGSSGPGGYGPGSQGPGAASAAAAAS

GPGGYGPGSQGPSGPGSQGPSGPGGASAAAAAASS

GPGGYGPGSQGPSGPGGYGPGSQGPSGPGGYGPGSSGPGGYGPGSQGPGAASAAAAAS

GPGGYGPGSQGPSGAGIQGPSGPGIQGPSGPGGASAAAAAASS

GPGGYGPGSQGPSGLGGYGPGSQGPSGPGGYGPGSSGPGGAGGYGPGSQGPGGSGAAAAAAAASS

GPGGYGPRSQGPSGPGSQGPSGPGGYGPGSSGPGGYGPGSQGPGAASAAAAAS

GPGGYGPGSQGPSGPGGASAAAAAASS

GPGGYGPGSQGSSGPGGYGPGSQGPSGPGGYGPGSSGPGGYGPGSQGPGAAAAAAASS

GPGGYGAGSQGPSGPGSQGPSGPLSQGPSGPGSQGPSGPGGASAAAAAASS

GPGGYGPGSQGPSGPGGYGPGNQGPSGPGGYGPGSSGPGGYGPGSQGPGAASAAAAAS

GPGGYGPGSQGPSGPGSQGPSGPGSQGPSGPGSQGPSGPGGASAAAAAASS

GPGGYGPGSQGPSGPGGYGPGSQGPSGPGGYGPGSQGPSGPGGYGPGSSGPGGAGGYGPGSQGPGGSGAAAAAAAASS

GPGGYGPGSQGPSGPGGYGPGSQGPSGPGGAGGYGPGSQGLGGSGAAAAAAAASS

GPGGYGPGSQGPSGPGGYGPGSQGPSGPGGYGPGSSGPGGYGPGSQGPGAASAAAAAS

GPGGYGPGSQGPSGPGSQGPSGPGGASAAAAAASS

GPGGYGPGSQGPSGPGSQGPSGPGGYGPGSSGPGGYGPGSQGPGAASAAAAAS

GPGGYGPGSQGPSGAGSQGPSGPGSQGPSGPGGYGPGSSGPGGAGGYGPGSQGPGGSGAAAAAAAASS

GPGGYGPGSQGPSGPGGYGPGSSGPGGAGGYGPGSQGPGRSGAAAAAAAASS

GPGGYGPGSQGPSGPGGYGPGSSGPGGYGPGSQGPGAASAAAAAS

GPGGYGPGSQGPSGPGSQGPSGPGGASAAAAAASS

GPGGYGPGSQGPSGPGGYGPGSQGPSGPGGYGPGSSGPGGAGGYGPGSQGPGGSGAAAAAAAASS

GPGGYGPGSQGPSGPGSQGPSGPGGASAAAAAASS

GPGGYGPGSQGPSGPRGYGPGSQGPSGPGGNGPGSSGPGGYGPGSQGPGAASAAAAAS

GPGGYGPGSQGPSGPGSQGPSGPGGASAAAAAASS

GPGGYGPGSQGPSGPGGYGPGSQGLSGPGGYGPGSSGPGGAGGYGPGSQGPGGSGAAAAAAAASS

GPGGYGPGSQGPSGPGSQGPSGPGGASAAAAAASS

GPGGYGPGSQGPSGPGGYGPGSQGPSGPGGYGPGSSGPGGYGPGSQGPGAASAAAAAS

GPVGYGPGSQGPSGPGSQGPSGPGGASAAAAAASS

GPGGFGPGSQGPSGPGGYGPGSQGPSGPGGYGPGSSGPGGAGGYGPGSQGPGGSGAAAAAAAASS

GPGGYGPGSQGPSGPGSQGPSGPGGYGPGSSGPVGYGPGSQGPGAASAAAAAS

GPGGYGPGSQGPSGPGSQGPSGPGGANAAAAAASS

GPGGYGPGSQGPSGPGGYGPGSQGPSGPGGYGPGSSGPGGYGPGSQGPGAASAAAAAS

GPGGYGPGSQGPSGPGSQGPSGPGGASAAAAAASS

GPGGYGPGSQGPSGPGGYGPGSQGPSGPGGYGPGSQGPSGPGGYGPGSSGPGGYGPGSQGPGAAAAAAAAS

GPGGYGPGSQGPSGPGGAGGNGPGSQGPGGSGGYGPGNQGPGGAGAAASAAASS

GPSGPGGYRPGVQGPSNAGGYGSSAPASVSVAASRLSSPAASSRVSSAVTSLVSSGPTNGASVSGALNGLVSQISSSNPGLSGCDVLVQALLELVSALVAILGSASIGAVDYNSVGQTTQTISQYFS

>Aaur_MaSp2.1a

MSCPRLVLAFLALLSTNALFAAAAAATPWDSPALADSFMKSFMDGIGTSGAFTSSQIDDMSTIGDTMMDSVNRLASSGRISKSKLQALNMAFASSMAEIAATEEGGLSIGAKTSAIASALRGAFLQTTGYANEQFINEITSLINMIAQANVNAVSASASASAGGGYGAPAYGPSSYGPSQQQSSASSVSVSASAAGAGPRSQAPSRPAQQGPRGYGPSGPGGTAAASASAGGPGSQGPYGPGQQGPGPRGPSRPSQQGPGGYGPSGPGGASAAAAAAAA

GGPGGQGPYGPGQQGPGAGPYGPGQQGPGQQGPGGYGPSGPGGAAAAAAAAAA

GGPGGQGPSGPGQQGPGGYGPSGPSGASAAAAAA

GGQGPYGQGQQGPRGYGPSGPGGTAAAAAAA

GGPGGQGQYGPGQQGPGGYGSSGTGGASAAAAAAAA

GGPGGQGPYGPGQQGPYGPGQQGPGGQGRGGYGPSGPGGASAAAAAAAA

GGPGGQGQYGPGQQGPGGYGPSGPGGASAAAAASAA

GGPGGQGPSGPGGQGPSGPGQQGPGGYGPSGPSGASAAAAVA

GGQGPYGQGQQGPGGYGPSGPAGASAASAAAAA

GGQGGQGPYGPGQQGPYGPGQQGPGGQGRGGYGPSGPGGASAAAAAAAA

GGPGGRGEYGPGQQGPGGYGPSGPGGASAAAAASAA

GGPGGQGPSGPGQQGPGGYGPSGPSGASAAAAVA

GGQGPYGQGQQGPGGYGPSGPAGASAASAAAAA

GGQGGQGPYGPGQQRPYGPGQQGPGGQGRGGYGPSGPGGASAAAAAAAA

GGPGGQGQYGPGQQGPGGYGPSGPGGASAAAAASAA

GGPGGQGPSGPGQQGPGGYGPSGPSGASAAAAVA

GGQGPYGQGQQGPGGYGPSGPAGASAASAAAAA

GGQGGQGPYGTGQQGPYGPGQQGPGGQGRGGYGPSGPGDASAAAAAAAA

GGPGGQGQYGPGQQGPGGYGPSGPGGASAAAAASAA

GGPGGQGPSGPGQQGPGGYGPSGPSGASAAAAVA

GGQGPYGQGQQGPGGYGPSGPAGASAASAAAAA

GGQGGQGPYGPGQQGPYGPGQQGPGGQGPGGYGPSGPGGASAAAAAAAA

GGPGGQGQYGPGQQGPGGYGPSGPGGASAAAAASAA

GGPGGQGPSGPGQQGPGGYGPSGPSGASAAAAVAV

GQGPYGQGQQGPGGYGPSGPAGASAASAAAAA

GGQGGQGPYGPGQQGPYGPGQQGPGQQGPGGYGPSGPGGSAAAAAAAAA

GGPGGQGPSGPGQQGPGGYGPSGPSGASAAAAAA

GGQGPYGQGQQGPGGYGPSGPAGASAASAAAAA

GGQGGQGPYGPGQQGPYGPGQQGPGGQGPGGYGPSGPGGASAAAAAAAA

GGSGGQGPYGPGQQGPYGPGQQGPGQQGPGGYGPSGPSGASAAAAAA

GGQGPYGQGQQGPGGYGPSGPAGASAASAAAAA

GGQGGQGPYGPGQQGPYGPGQQGPGGQGPGGYGPSGPGGASAAAAAAAA

GGSGGQGPYGPGQQGPYGPGQQGPGQQGPGGYGPSGPSGASAAAAAA

GGQGPYGQGQQGPGGYGPSGPAGASAASAAAAA

GGQGGQGPYGPGQQGPYGPGQQGPGGQGPGGYGPSGPGGASAAAAAAAA

GGSGGQGPYGPGQQGPYGPGQQGPGQQGPGGYGPSGPGGTAAAAAAAAA

GGPAGQGPSGPGQQGPGGYGPSGPSGASAAAAAA

GGQGPYGQGQQGPGGYGPSGPAGASAASAAAAA

GGQGGQGPYGPGQQGPYGPGQQGPGGQGPGGYGPSGPGGASAAAAAAAA

GGSGGQGPYGPGQQGPYGPGQQGPGQQGPGGYGPSGPSGASAAAAVA

GGQGPYGQGQQGPGGYGPSGPAGASAASAAAAA

GGQGGQGPYGPGQQGPYGPGQQGPGGQGPGGYGPSGPGGASAAAAAAAA

GGSGGQGPYGPGQQGPGQQGPGGYGPSGPSGASAAAAAA

GGQGPYGQGQQGPGGYGSSGSGGAAAAAATA

GGPGGQGQYGPGQQGPGGYGPSGPGAASAAAAAAAA

GGPGGQGLSGPGQQGPGGYGPSGPSGASAAAAVA

GGQGPYGQGQQGPGGYGPSGPAGVSAASAAAAA

GGQGGQGPYGPGQQGPYGPGQQGPGGQGPGGYGPSGPGGASAAAAAAAA

GGSGGQGPYGPGQQGPYGPGQQGPGGQGPGGYGPSGPGGSAAAAAAAASAAA

GGAGGQGPSGPGQQGPESYGPSGPSGASAATAAAGGQGPYGQGQQGPGGYGPSGPVSGVSVSVSSAASRLSSPAASSRVSSAVSTLASSGPSDAGVVSSALSNLVSQVSTNHPGLSECDVIVQALLELVSALVHILGSSSVGQVDYNGASYSAQNLGQAVAQALA

>Aaur_MaSp2.1b

MSCPRLVLAFLALLSTHALFAAAAGATPWDSPALADSFMKCFMDGIGTSGAFTSSQIDDMSTIGDTMIDSVNRLASSGRISKSKLQALNMAFASSMAEIAATEEGGLSIGAKTSAIASALRGAFLQTTGYSNEQFINEITSLVSMIAQANTNSVSASASASAGGGYGGSSYGPSSVSSVSASASSAGAGPAQQGPGSYGPSGPGGYGPSGSSAAAAAS

GGQGPGNYGSSGSGGAGPSGPGGYGPGSQGSSGPGNQGPGGVSAAAAAAS

GPGGYGPGSQGSSGPGGYGPLSQGQSGPGGAGGYGPGGASAAAAAVAAS

GPAGYGQGSQGPSGTGASGPGGAGGYGPGSQGPGGAAAAAAAS

GPGGYGPGSQGPSGPGGFGPGSQGQSGPGGYGPGNQGQSGPSGAGGYGPGGASAAAAAAAAS

GPGGYGPGSQGPYGPGSQGPSGPGGYGSGSSGPGGAGGYGPGSQGPGGQGTAAAAAAAS

GPGGYGSGNQGPSGTGASGPGGAGGYGPGSQGPGAAAAAAAAS

GPAGYGPGSQGQSGPGSQGPGGASAAAAAAAS

GPGGYGPGSQGPSGPSGYGPGASGPGGAAGYGPGSQGPGAASAAAAAAAAS

GSGGYGPGSQGPYGPGSQGPSGSGSQGPSGSGGYGPGASGPGGAGSYGPGSQGPGGASAAAAAAASA

PGGYGPGSQGSSGPSGYGPGASGPGGAGGYGPGSQGPGGASAAAAAAAAS

GPGGYGPGSQGPYGPGNQGPSGPGSQGPSGSGGYGPGASGPGGTGSYGPGSQGPGGASAAAAAAASA

PGGYGPGSQGLSGPSGYGPGASGPSGAGGYGPGSQGPGGASAAAAAAAAS

GPGGYGPGSQGPYGPGSQGPSGPGSQGPSGSGGYGPGASGPGGYGPGSQGPGGASAAAAAAAS

GPGGYGPGSQGQSGPGGYGSGSSGPGGAGGYGPGSQGPGGASAAAAAAAS

GPGGYGPGSQGPSGPGSQGPSGPGSQGPSGSYGYGPGASGPGGAGSYGPGSQGPGGASAAAAAAAS

GPGGYGPGSQGPSGPGSQGPSGSGGYGPGASGPGGYGPGSQGPGGASAAAAAAAS

GPGGYGPGSQGQSGPGGYGSGASGPGGAGGYGPGSQGPGGASAAAAAAAS

GPGGYGPGSQGPSGPGSQGPSGPGSQGPSGSGGYGPGASGPGGAGSYGPGSQGPGGASAAAAAAAS

GPEGYGPGSGGPSGPSGYGPGASGPGGTGGYGPGSQGPGGASAAAAAAAS

GPGGYGPGSQGPSGPGSQGPSGPGSQGPSGSGGYGPGASGPGGYGPGSQGPGGASAAAAAAAS

GPGGYGPGSQGQSGPGGYGSGSSGPGGAGGYGPGSQGPGGASAAAAAAAS

GPGGYGPGSQGPSGPGSQGPSGPGSQGPSGSYGYGPGASGPGGAGSYGPGSQGPGGASAAAAAAAS

GPGGYGPGSQGPSGPGSQGPSGSGGYGPGVYGPGSQGPGGASAAAAAAAS

GPGGYGPGSQGQSGPGGYGSGASGPGGAGGYGPGSQGPGGASAAAAAAAS

GPGGYGPGSQGPSGPGSQGPSGPGSQGPSGSGGYGPGASGPGGAGSYGPGSQGPGGASAAAAAAAS

GPGGYGPGSGGPSGPSGYGPGASGLGSAGGYGRGSQGPGGASAAAAAAAS

GPGGYGPGSQGPSGPGSQGPSGSGGYGPGASGPGGYGPGSQGPGGASAAAAAAAS

GPGGYGPGSQGQSGPGGYGSGSSGPGGAGGYGPGSQGPGGASAAAAAAAS

GPGGYGPGSQGPSGPGSQGPSGPGSQGPSGSYGYGPGASGPGGAGSYGPGSQGPGGASAAAAAAAS

GPGGYGPGSQGPSGPGSQGPSGSGGYGPGASGPGVYGPGSQGPGGASAAAAAAAS

GPGGYGPGSQGQSGPGGYGSGASGPGGAGGYGPGSQGPGGASAAAAAAAS

GPGGYGPGSQGSSGPGSQGPSGPGSQGPSGSGGYGPGASGPGGAGSYGPGSQGPGGASAAAAAAAS

GPGGYGPGSGGPSGPSGYGPGASGPGSAGGYGPGSQGPGGASAAAAAAAS

GPGGYGPGSQGPAGPGSQGPSGPGSQGPSGSGGYGPGASGPGGYGPGSQGPGGASAAAAAAAS

GPGGYGPGSQGQSGPGGYGSGSSGPGGAGGYGPGSQGPGGASAAAAAAAS

GPGGYGPGSQGPSGPGSQGPSGPGSQGPSGSGGYGPGASGPGGAGSYGPGSQGPGGASAAAAAAAS

GPGGYGPGSQGPSGPGSQGPSGSGGYGPGASGPGVYGPGSQGPGGASAAAAAAAS

GPGGYGPGSQGPSGPGSQGPSGPGSQGPSGSGGYGPGASGPGGAGSYGPGSQGPGGASAAAAAAAS

GPGGYGPGSGGPSGPSGYGPGASGPGSAGGYGPGSQGPGGASAAAAAAAS

GPGGYGPGSQGPSGPGSQGPSGPGSQGPSGSGGYGPGASGPGGYGPGSQGPGGASAAAAAAAS

GPGGYGPGSQGQSGPGGYGSGSSGPGGAGGYGPGSQGPGGASAAAAAAAS

GPGGYGPGSQGPSGPGSQGPSGPGGAGAAAAAAAAS

GPGGYGPGSQGSSGPGSQGPSGSGGYGPGASGSGGYGPGSQGPGGASAAAAAAAS

GPGGYGPGSQGPSGPGSQGPSGPSVYGPGASGPGGAGGYGPGSQGPGGASAAAAAAS

GPGGYGPGSQGPSGPGSQGPSGPGSQGPSGSGGYGPGASGPGGYGPGSQGPGGASAAAAAAAS

GPGGYGPGSQGPSGPGSQGPSGPSGYGPGASGPGGAGGYGPGSQGPGGASAAAAAAS

GPGGYGPGGQGPSGPGSQGPSGPGGSGAAAAAAAS

GPGGYGPGSQGLSGPGSQGSSGLGGYGPGGAGGYGPGSQGPGGASAAAAAAAAS

GPGGYGPGSQGPSGPGSQGPSGPGSQGPSGSGGYGPGASGSGGYGPGSQGPGGASAAAAAAAS

GPGGYGPGSQGPSGPGSQGPSGPSGYGPGASGPGGAGGYGPGSQGPGGASAAAAAAS

GPGGYGPGSQGTSGPGSQGPSGPGSQGPSGSGGYGPGASGPGGYGPGSQGSGGASAAAAAAAS

GPGGYGPGSQGSSGLGGYGPGGAGGYGPGSQGPGGASAAAAAAATS

GPGGYGPGSQGPSGPGSQGPSGPGSFGPGGAGGYGPSASATVSVAASRLSSPAASSRVSSTVSSLVSSGPSNGAAVSGALNGLVSQISSSNPGLSGCDVLVQALLELVSALVAILGSANIGSVDYYSVGQTTQTISQYFS

>Atri_MaSp2.1a

MSCPRLVLAFLALLSTHALFASAGGQTPWDSPALADSFMKSFMDGIGASGAFSSSQIDDMSTIGDTMMDSVNRLASSGRISKSKLQALNMAFASSMAEIAATEEGGLSIGAKTSAIASALRGAFLQTTGYSNEQFINEITSLVSMIAQANVNTVSASASAAAGGGYGSPAYGPSSYGPSQQQSSASSVSVSASAAGPGPRGQAPSRPAQQGSAGYGPSGPGGAAAAAAAA

GPGQQRPSGPSQQGPGSYGPSGPGGASAAAAAAAA

GGPGGQGQYGPGQQGPGAYRQQGPGQQGPGGYGPSGPGGASAAAAAAAA

GGPGGQGPYGPGQQGPGGYGPSGPGGASAAAAAAAA

GGPGGQGPYGPGQQGPGAGQYGPGQQGPGGRGPGGYGPSGPGGASAAAAAAAA

GGPGGQYGPGQQGPGSGGPYGQQGPGQQGPGGYGPSGPGGASAAAAAAAA

GGPGGQGPSGPGQQGPGGYGPSGPGAAAAAAAAA

GGPGSQGPGQQGPGGYGPSGPGGASAAAAAAAA

GGPGGQGSYGPGQQGPGAGQYGPGQQGPGGRGPGGYGPSGPGGASAAAAAAAA

GGPGGQYGPGQQGPGSGGPYGQQGPGQQGPGGYGPSGPGGASAAAAAAAA

GGPGGQGPSGPGQQGPGGYGPSGPGAAAAAAAAA

GGPGSQGPGQQGPGGYGPSGPGGASAAAAAAAA

GGPGGQGSYGPGQQGPGAGQYGPGQQGPGGRGPGGYGPSGPGGASAAAAAAAA

GGPGGQYGPGQQGPGSGGPYGQQGPGQQGPGGYGPSGPGGASAAAAAAAA

GGPGGQGPSGPGQQGPGGYGPSGPGAAAAAAAAA

GGPGSQGPGQQGPGGYGPSGPGGASAAAAAAAA

GGPGGQGSYGPGQQGPGAGQYGPGQQGPGGRGPGGYGPSGPGGASAAAAAAAA

GGPGGQYGPGQQGPGSGGPYGQQGPGQQGPGGYGPSGPGGASAAAAAAAA

GGPGGQGPSGPGQQGPGGYGPSGPGAAAAAAAAA

GGPGSQGPGQQGPGGYGPSGPGGASAAAAAAAA

GGPGGQGSYGPGQQGPGAGQYGPGQQGPGGRGPGGYGPSGPGGASAAAAAAAA

GGPGGQYGPGQQGPGSGGPYGQQGPGQQGPGGYGPSGPGGASAAAAAAAA

GGPGGQGPSGPGQQGPGGYGPSGPGAAAAAAAAA

GGPGSQGPGQQGPGGYGPSGPGGASAAAAAAAA

GGPGGQGSYGPGQQGPGAGQYGPGQQGPGGRGPGGYGPSGPGGASAAAAAAAA

GGPGGQYGPGQQGPGSGGPYGQQGPGQQGPGGYGPSGPGGASAAAAAAAA

GGPGGQGPSGPGQQGPGGYGPSGPGAAAAAAAAA

GGPGSQGPGQQGPGGYGPSGPGGASAAAAAAAA

GGPGGQGSYGPGQQGPGAGQYGPGQQGPGGRGPGGYGPSGPGGASAAAAAAAA

GGPGGQYGPGQQGPGSGGPYGQQGPGQQGPGGYGPSGPGGASAAAAAAAA

GGPGGQGPSGPGQQGPGGYGPSGPGAAAAAAAAA

GGPGSQGPGQQGPGGYGPSGPGGASAAAAAAAA

GGPGGQGSYGPGQQGPGAGQYGPGQQGPGGRGPGGYGPSGPGGASAAAAAAAA

GGPGGQYGPGQQGPGSGGPYGQQGPGQQGPGGYGPSGPGGASAAAAAAAA

GGPGGQGPSGPGQQGPGGYGPSGPGAAAAAAAAA

GGPGSQGPGQQGPGGYGPSGPGGASAAAAAAAA

GGPGGQGSYGPGQQGPGAGQYGPGQQGPGGRGPGGYGPSGPGGASAAAAAAAA

GGPGGQYGPGQQGPGSGGPYGQQGPGQQGPGGYGPSGPGGASAAAAAAAA

GGPGGQGPSGPGQQGPGGYGPSGPGAAAAAAAAA

GGPGSQGPGQQGPGGYGPSGPGGASAAAAAAAA

GGPGGQGSYGPGQQGPGAGQYGPGQQGPGGRGPGGYGPSGPGGASAAAAAAAA

GGPGGQYGPGQQGPGSGGPYGQQGPGQQGPGGYGPSGPGGASAAAAAAAA

GGPGGQGPSGPGQQGPGGYGPSGPGAAAAAAAAA

GGPGSQGPGQQGPGGYGPSGPGGASAAAAAAAA

GGPGGQGSYGPGQQGPGAGQYGPGQQGPGGRGPGGYGPSGPGGASAAAAAAAA

GGPGGQYGPGQQGPGSGGPYGQQGPGQQGPGGYGPSGPGGASAAAAAAAA

GGPGGQGPSGPGQQGPGGYGPSGPGAAAAAAAAA

GGPGSQGPGQQGPGGYGPSGPGGASAAAAAAAA

GGPGGQGSYGPGQQGPGAGQYGPGQQGPGGRGPGGYGPSGPGGASAAAAAAAA

GGPGGQYGPGQQGPGSGGPYGQQGPGQQGPGGYGPSGPGGASAAAAAAAA

GGPGGQGPSGPGQQGPGGYGPSGPGAAAAAAAAA

GGPGSQGPGQQGPGGYGPSGPGGASAAAAAAAA

GGPGGQGSYGPGQQGPGAGQYGPGQQGPGGRGPGGYGPSGPGGASAAAAAAAA

GGPGGQYGPGQQGPGSGGPYGQQGPGQQGPGGYGPSGPGGASAAAAAAAA

GGPGGQGPSGPGQQGPGGYGPSGPGAAAAAAAAA

GGPGSQGPGQQGPGGYGPSGPGGASAAAAAAAA

GGPGGQGSYGPGQQGPGAGQYGPGQQGPGGRGPGGYGPSGPGGASAAAAAAAA

GGPGGQYGPGQQGPGSGGPYGQQGPGQQGPGGYGPSGPGGASAAAAAAAA

GGPGGQGPSGPGQQGPGGYGPSGPGAAAAAAAAS

GGPGSQGPGQQGPGAYGPSGPGGASAAAAAAAA

GGPGGQGPYGPGQQGPGAGQYGPGQQGPGQQGPGGYGPSGPSGAAAAAAAAAA

GGQGPYGPGQQGPGGYGPSGPAQQGPGSYGPSGPSGAAAAAAAA

GGQGPYGQRQQGPGGYGPSGPVSGISASVSSAASRLSSPAASSRVSSAVSTLASSGPSDAGVVSSALSNLVSQVSSNHPGLSGCDVIVQALLELVSALVHILGSSSLGQVDYNGASYSAQTLGQAVAQALA

>Atri_MaSp2.1b

MSYPRLVLAFLALLSTHALFAAAGGQTPWDTPTLADNFMKCFMNEIGNSGAFTSNQVDDMSTIGDTMMDSVNRLASSGRISKSKLQALNMAFASSMAEIAATEEGGLSIGSKTNAIASALRGAFLQTTGYSNEQFINEITSLVSMIAEANVNTVSASASAYAGGGYGGSSYGSSSVNSASAAATGPAQQGPGSYGPSSVPGGYGPSGSSAAAAAS

GGQGLGNYGPSGSGGAGPSGTGGYGPGSQGPSRPSGPGAAAAAAAAS

GPGGYGPSAPSGPGSQGPSGPSGSGASAAAAAAS

GSGGYGSGSQGPSGPGSQGPSGPGTSAAAAAAANGPGGYGPGSQGPSGPGGYRPGSQGPSGPGSSGPGMSGGYGPGNQGPGGASVAAAAA

GSGPGGYGPGSQGPSGPGSSGPGMSGGYGPGNQGPGGASAAAAAAS

GPGGYGPGSQGSSGPGAYGPGSQGSSGPGSSGPGMSGGYGPGNQGPGGASAAAAAAAS

GPGGYGPXSKGPGRHXGPGSSGPGMSGGYGPGNQGPGGASAAAAAAAS

GPGGYGPGSQGPSGPGAYGPGSQGSSGPGSSGPGMSGGYGPGNQGPGGASAAAAAAAS

GPGGYGPGSQGPSGPGAYGPGSQGSSGPLSSGPDMSGGYGPGNQGPGGASAAAAAAAS

GPGGYGPGSQGPSGPGAYGPGSQGSSGPGSSGPGMSGGYGPGNQGPGGASAAAAAVAS

GPGGYGPGSQGSSGTGAYGTGSQGSTGPGSSGPGMSGGYGPGNQGPGGASAAAAAAAS

GPGGYGPGSQGSSGPGAYGPGSQGSSRPLSSGPGMSGGYGPGNQGPGGASAAAAAAAS

GPGGYGPGSQGPSGPGAYGSGSQGSSGPGSSGPGMSGGYGPGNQGPGRASAAAAAAAS

GPGGYGPGSQGPSGPGAYGPGSQGSSGPLSYGTDMSGGYGPGNQGPGGASAAAAAAAS

GPGGYGPGSQGPSGPGAYGPGSQGSSGPGSSGPGMSGGYGPGNQGPGGASAAAAAEAS

GPGAYGPGSQGSSRLLSSGPGMSGGYGPGNQGPGGASAAAYAAAS

GLGGYGPGSQGPSGPGAYGSGSQGSSGPGSSGPGMSGGYGSGNQGPGRASAAAAAAAI

GPGGYGPGSQGSSGTGAYGPGSQGSSGPLSSGPGMSGGYGPGNQGPGGASAAAAAAAS

GPGGYGPGSQGPSGPGAYGPGSQGSSGPLSYGTDMSGGYGPGNQGPGGASAAAAAAAS

GPGGYGPGSQGSSGPGAYGTGSQGSTGPGSSGPGMSGGYGPGNQGPGGANAAAAAAAS

GPGGYGPGSQGSSGPGAYGPGSQGSSRPLSSGPGMSGGYEPGNQGPGGASAAAAAAAS

GPGGYGPGSQGPSGPGAYGPGSQGSSGPRSSGPGMSGGYGPGNQGPGGASAAAAAAAS

GPGAYGPGSQGSSGPGAYGPGSQGSSGPVSSGPDMSGGYGPGNQGPGGASAAAAAAAS

GPGGYGPGSQGPSGPGAYGPGSQGSSGPGSSGPGMSGGYEPGNQGPGGASAAAAAAAS

GPGGYGPGSQGPSGPGAYGPGSQGSSGPGSSGPGMSGGYGPGNQSTGGASAAAAAAAS

XPGAYGPGSQGSSGPGAYGPGSQGSSGPGSYGPGMSGGYGPGNQGPGGASAAAAAAAS

GPAGYGLGSQGSSGPGSSGPGMSGGYGPGNQGLGRASAVAAAAAS

GPGGYGPGSQGPSRPVAYGPGSQGPSVPGAYGPGSQGSSGPGSSGPGVSGSYGPGNQGPGGASAAAAAAAS

GPGAYGPGSQGSSGPGSFGPGMSGGYGPGNQGPGGASAAADAAA

SGPGGYGPGSQGPSGLGAYGPGSQGSSGPGSSGPGMSGGYGPGNQGPGGASAAAAAS

GPGSQGPSVLGGYGPGSQGPSGWGSQGPSAPSGYGPSASVSASAAASRLSSPAASSRVSSAVSSLVSSGPTSGAAVSGALNGLVSQISSNNPGLSGCDVLVQALLELVSALVAILGSASIGAVDYNSVGQTTQTISQYFS

>Aarg_MaSp2.2a

MNLSIRLALLGFVVLSTQTIFAAGQAATPWQNSQLAEQFINSFLRFIGQSGAFSPDQLDDMSTIGETLKTAIEKMAQSRNSSRSKLQALNMAFASSMAEIAVAEQGGLSLEAKTNAIASALTSAFLETTGVVNQQFVSEIKGLIYMIAQASSNEISGSAAASGGGSGGGGGGYGQGSYASASAAAAYGSAPQGAGGPASQGPSQQGPVSQPSYGPSATVVVSAVGGYGPVAGQQGPSGASQQGPGGQGPSGPVAAAAAVT

GGYGPGAGAGGPQRPIGAGPSVPSARGPGAAGAGPQAGPGGPGGAGPSAAAAAAA

GAGGFGPGAGGQQGPGGAGAYGPSAGGQRGPGGQGSYGPGAAATAAAAAA

GGFGPGGAGAGPQAGPGQQGPGGQGPYGPGAAAAAAAA

GGSGPGVGGYQGPGGAGQQEPGGQGPYGPGAAAAAAAA

GGSGPGAGGQRGPGQQGPGGQGPYGPGAAAAAAAAA

GGYGPGGAGAGPQAGPGGPGGAGPSAAAAAAA

GAGGFGPGAGGQQGPGGAGAYGPSAGGQRGPGGQGPYGPGAAAAAAAAA

GGFGPGGAGAGPQAGPGQQGPGGQGPYGPGAAAAAAAA

GGSGPGAGGYQGPGGAGQQGPGGQGPYGPGAAAAAAAA

GGSGPGAGGQRGPGQQGPGGQGPYGPGAAAAAAAAA

GGYGPGGAGAGPQAGPGGPGGAGPSAAAAAAA

GAGGYGPGAGGQQGPGGAGAYGPSAGGQRGPGGQGPYGPGAAAAAAA

GGFGPGGAGAGPQAGPGQQGPGGQGPYGPGAAAAAAAA

GGSGPGAGGYQGPGGAGQQGPGGQGPYGPGAAAAAAAA

GGSGPGAGGQRGPGQQGPGGQGPYGPGAAAAAAAAA

GGYGPGGAGAGPQAGPGGPGGAGPSAAAAAAA

GAGGYGPGAGGQQGPGGAGAYGPSAGGQRGPGGQGPYGPGAAAAAAA

GGFGPGGAGAGPQAGPGQQGPGGQGPYGPGAAAAAAAA

GGSGPGAGGYQGPGGAGQQGPGGQGPYGPGAAAAAAAA

GGSGPGAGGQRGPGQQGPGGQGPYGPGAAAAAAAAA

GGYGPGGAGAGPQAGPGGPGGAGPSAAAAAAA

GAGGFGPGAGGQQGPGGAGAYGPSAGGQRGPGGQGPYGPGAAAAAAA

GGFGPGGAGAGPQAGPGQQGPGGQGPYGPGAAAAAAAA

GGSGPGAGGYQGPGGAGQQGPGGQGPYGPGAAAAAAAA

GGSGPGAGGQRGPGQQGPGGQGPYGPGAAAAAAAAA

GGYGPGGAGAGPQAGPGGPGGAGPSAAAAAAA

GAGGFGPGAGGQQGPGGAGAYGPSAGGQRGPGGQGPYGPGAAAAAAA

GGFGPGGAGAGPQAGPGQQGPGGQGPYGPGAAAAAAAA

GGSGPGAGGYQGPGGAGQQGPGGQGPYGPGAAAAAAAA

GGSGPGAGGQRGPGQQGPGGQGPYGPGAAAAAAAAA

GGYGPGGAGAGPQAGPGGPGGAGPSAAAAAAA

GAGGFGPGAGGQQGPGGAGAYGPSAGGQRGPGGQGPYGPGAAAAAAA

GGFGPGGAGAGPQAGPGQQGPGGQGPYGPGAAAAAAAA

GGSGPGAGGYQGPGGAGQQGPGGQGPYGPGAAAAAAAA

GGSGPGAGGQRGPGQQGPGGQGPYGPGAAAAAAAAA

GGYGPGGAGAGPQAGPGGPGGAGPSAAAAAAA

GAGGFGPGAGGQQGPGGAGAYGPSAGGQRGPGGQGPYGPGAAAAAAA

GGFGPGGAGAGPQAGPGQQGPGGQGPYGPGAAAAAAAA

GGSGPGAGGYQGPGGAGQQGPGGQGPYGPGAAAAAAAA

GGSGPGAGGQRGPGGQGPYGPGAAAAAAAAA

GGYGPGGAGAGPQAGPGGPGGAGPSAAAAAAA

GAGGFGPGAGGQQGPGGAGAYGPSAGGQRGPGGQGPYGPGAAAAAAA

GGFGPGGAGAGPQAGPGQQGPGGQGPYGPGAAAAAAAA

GGSGPGAGGYQGPGGAGQQGPGGQGPYGPGAAAAAAAA

GGSGPGAGGQRGPGQQGPGGQGPYGPGAAAAAAAAA

GGYGPGGAGAGPQAGPGGPGGAGPSAAAAAAA

GAGGFGPGAGGQQGPGGAGAYGPSAGGQRGPGGQGPYGPGAAAAAAA

GGFGPGGAGAGPQAGPGQQGPGGQGPYGPGAAAAAAAA

GGSGPGAGGYQGPGGAGQQGPGGQGPYGPGAAAAAAAA

GGSGPGAGGQRGPGQQGPGGQGPYGPGAAAAAAAAA

GGYGPGGAGAGPQAGPGGPGGAGPSAAAAAAA

GAGGFGPGAGGQQGPGGAGAYGPSAGGQRGPGGQGPYGPGAAAAAAA

GGFGPGGAGAGPQAGPGQQGPGGQGPYGPGAAAAAAAA

GGSGPGAGGYQGPGGAGQQGPGGQGPYGPGAAAAAAAA

GGSGPGAGGQRGPGQQGPGGQGPYGPGAAAAAAAAA

GGYGPGGAGAGPQAGPGGPGGAGPSAAAAAAA

GAGGFGPGAGGQQGPGGAGAYGPSAGGQRGPGGQGPYGPGAAAAAAA

GGFGPGGAGAGPQAGPGQQGPGGQGPYGPGAAAAAAAA

GGSGPGAGGYQGPGGAGQQGPGGQGPYGPGAAAAAAAA

GGSGPGAGGQRGPGQQGPGGQGPYGPGAAAAAAAVA

GGYGPGGAAAGPQAGPGGPGGAGPSAAAATAA

GAGGFGPGAGGQQGPGGAGAYGPSAGGQRGPGGQGPYGPGAAAAAAAAA

GGFGPGGAGPGPQAGPRGAQPYGPSAAAAV

GGYGPGAGQQGPGRQGPAGPGQQGSGGQGPYGPGASAAAAAA

GGYGPGAGQQGPRSQAPVASAAASRLASPQASSRVSSAASTLVSSGPANPAALSNTISSVVSQISASNPGLSGCDVLVQALLEIVSALVYILGSSSIGQINYGAASQYTQLVGRSVAQALG

>Aarg_MaSp2.2b

MNWSIRLALFGFVVLSTQTVFAVGQAATPWENSQLAEDFINSFLRFIAQSGAFSPNQLDDMSSIGDTLKTAIEKMAQSRKSSKSKLQALNMAFASSMAEIAVAEQGGLSLEAKTNAIASALASAFLETTGVVNQQFVSEIKGLIYMIAQASSNEISGSASGSGGGSGGGGGGGGGYGPGSYASASVAAAYGSAPQGAGGPSPQGPSQQAPISQGPYGPGAAAAAAAS

GGYGPGAGQQGPSGGGQQGPGGAGQQGPGGQGPYVPSAAAAAA

GGYGPGAGQQGPGGAGQQGPGPQGPGGAGQRGPYGPGAAAAAAAA

GGYGPGAGQQGPGSGGQQGPSGQGPYGPGASAAAAAA

GGYGPGARQQGPGGQGAGSGGQQGPGSQGPGGAVQQGPYGPGAAAAAAAA

RGYGPGAGQQGPGGAGQQGPGSQGPGGAGQRGPYGPGAAAAAAAA

GGSGPGAGQQGPGSGGQQGPSGQGPYGPGASAAAAAA

GGYGPGAGQQGPGGQGAGSGGQQGPGSQGPGGAGQQGPYGPGAAAAAAAA

RGYGPGAGQQGSGRAGQQGPGSQGPGGAGQRGPYGPGAAAAAAAA

GGSGPGAGQQGPGSGGQQGPSGQGPYGPGASAAAAAA

GGYGPGAGQQGPGGQGAGSGGQQGPGSQGPGGAGQQGPYGPGAAAA

GGYGPGAGQQGPRSGGQQGPSGQGPYGPGASAAAAAA

GGYGPGAGQQGPGGQGAGSGGQQGPGSQGPGGAGQQGPYGPGAAAAAAAA

GGYGPGGGQQGPGGAGQQGPGSQGPGGAGQRGPYGPGAAAAAAAA

GGYGPGAGQQGPGSGGQQGPSGQGPYGPGASAAAAAA

GGYGPGAGKQGPGGQGAGSGGQQGPGSQGPGGAGQQGPYGPGAAAAAAAA

GGYGPGAGQQGPGSGGQQGPSGQGPYGPGASAAAAAA

GGYGPGAGQQGPGGQGAGSGGQQGPGSQGPGGAGQQGPYGPGAAAAAAAA

RGYGPGAGQQGPGGAGQQGPVSQGPGGAGQQGPYGPGAAAAAAAA

GGYGPGAGQQGPGGAGQQGPGSQGPGGAGQRGPYGPGAAAAAAAA

GGYGPGAGQQGPGSGGQQGPSGQGPYGPGASAAAAAA

GGYGPGAGQQGPGGQGAGSGGQQGPGSQGPGGAGQQGPYGPGAAAAAAAA

GGYGPGAGQQGPGGAGQQGPGSQGPGGAGQRGPYGPGAAAAAAAA

GGYGPGAGQQGPGSGGQQGPSGQGPYGPGASAAAAAA

GGYGPGAGQQGPGGQGAGSGGQQGPGSQGPGGAGQQGPYGPGAAAAAAAA

GGYGPGAGQQGPGGAGQQGPGSQGPGGAGQRGPYGPGAAAAAAAA

GGYGPGAGQQGPGSGGQQGPSGQGPYGPGASAAAAAA

GGYGPGAGQQGPGGQGAGSGGQQGPGSQGPGGAGQQGPYGPGAAAAAAAA

GGYGPGAGQQGPGGAGQQGPGSQGPGGAGQRGPYGPGAAAAAAAA

GGYGPGAGQQGPGSGGQQGPSGQGPYGPGASAAAAAA

GGYGPGAGQQGPGGQGAGSGGQQGPGSQGPGGAGQQGPYGPGAAAAAAAA

GGYGPGAGQQGPGGAGQQGPGSQGPGGAGQRGPYGPGAAAAAAAA

GGYGPGAGQQGPGSGGQQGPSGQGPYGPGASAAAAAA

GGYGPGAGQQGPGGQGAGSGGQQGPGSQGPGGAGQQGPYGPGAAAAAAAA

GGYGPGAGQQGPGGAGQQGPGSQGPGGAGQRGPYGPGAAAAAAAA

GGYGPGAGQQGPGSGGQQGPSGQGPYGPGASAAAAAA

GGYGPGAGQQGPGGQGAGSGGQQGPGSQGPGGAGQQGPYGPGAAAAAAAA

GGYGPGAGQQGPGGAGQQGPGSQGPGGAGQRGPYGPGAAAAAAAA

GGYGPGAGQQGPGSGGQQGPSGQGPYGPGASAAAAAA

GGYGPGAGQQGPGGQGAGSGGQQGPGSQGPGGAGQQGPYGPGAAAAAAAA

GGYGPGAGQQGPGGAGQQGPGSQGPGGAGQRGPYGPGAAAAAAAA

GGYGPGAGQQGPGSGGQQGPSGQGPYGPGASAAAAAA

GGYGPGAGQQGPGGQGAGSGGQQGPGSQGPGGAGQQGPYGPGAAAAAAAA

RGYGPGAGQQGPGGAGQQGPGSQGPGGAGQQGPYGPGAAAAAAAA

GGYGPGAGQQGPGGAGQQGPGSQGPGGAGQQGPYGPGAAAAAAAA

GGYGPGAGQQGPGGAGQQGPGSQGPGGAGQRGPYGPGAAAAAAAA

GGYGPGAGQQGPGSGGQQGPSGQGPYGPGASAAAAAA

GGYGPGAGQQGPGGQGAGSGGQQGPGSQGPGGAGQQGPYGPGAAAAAAAA

GGYGPGAGQQGPGGAGQQGPGSQGPGGAGQRGPYGPGAAAAAAAA

GGYGPGAGQQGPGSGGQQGPSGQGPYGPGASAAAAAA

GGYGPGAGQQGPGGQGAGSGGQQGPGSQGPGGAGQQGPYGPGAAAAAAAA

RGYGPGAGQQGPGGAGQQGPGSQGPGGAGQQGPYGPGAAAAAAAA

GGYGPGAGQQGPGGAGQQGPGSQGPGGAGQRGPYGPGAAAAAAAA

GGYGPGAGQQGPGSGGQQGPSGQGPYGPGASAAAAAA

GGYGPGAGQQGPGGQGAGSGGQQGPGSQGPGGAGQQGPYGPGAAAAAAAA

GGYGPGAGQQGPGGAGQQGPGSQGPGGAGQRGPYGPGAAAAAAAA

GGYGPGAGQQGPGSGGQQGPSGQGPYGPGASAAAAAA

GGYGPGAGQQGPGGQGAGSGGQQGPGSQGPGGAGQQGPYGPGAAAAAAAA

GGYGPGAGQQGPGGAGQQGPGSQGPGGAGQRGPYGPGAAAAAAAA

GGYGPGAGQQGPGSGGQQGLKFITYYSKGPSGQGPYGPGASAAAAAA

GGYGPGAGQQGPGGQGAGSGGQQGPGSQGPGGAGQQGPYGPGAAAAAAAA

RGYGPGAGQQGPGGAGQQGPGSQGPGGAGQQGPYGPGAAAXXAAA

GGYGPGAGQQGPGGAGQQGPGSQGPGGAGQRGPYGPGAAAAAAAA

GGYGPGAGQQGPGSGGQQGPSGQGPYGPGASAAAAAA

GGYGPGAGQQGPGGQGAGSGGQQGPGSQGPGGAGQQGPYGPGAAAAAAAA

GGYGPGAGQQGPGGAGQQGPGSQGPGGAGQRGPYGPGAAAAAAAA

GGYGPGAGQQGPGSGGQQGPSGQGPYGPGASAAAAAA

GGYGPGAGQQGPGGQGAGSGGQQGPGSQGPGGAGQQGPYGPGAAAAAAAA

RGYGPGAGQQGPGGAGQQGPGSQGPGGAGQQGPYGPGAAAAAA

GGYGPGAGQQGPGGAGQQGPGSQGPGGAGQRGPYGPGAAAAAAAA

GGYGPGAGQQGPGSGGQQGPSGQGPYGPGASAAAAAA

GGYGPGAGQQGPGGQGAGSGGQQGPGSQGPGGAGQQGPYGPGAAAAAAAA

RGYGPGAGQQGPGGAGQQGPGSQGPGGAGQQGPYGPGAAAAAAA

GGYGPGAGQQGPGGAGQQGPGSQGPGGAGQRGPYGPGAAAAAAAA

GGYGPGAGQQGPGSGGQQGPSGQGPYGPGASAAAAAA

GGYGPGAGQQGPGGQGAGSGGQQGPGSQGPGGAGQQGPYGPGAAAAAAAA

GGYGPGAGQQGPGGAGQQGPGSQGPGGAGQRGPYGPGAAAAAAAA

GGYGPGAGQQGPGSGGQQGPSGQGPYGPGASAAAAAA

GGYGPGAGQQGPGGQGAGSGGQQGPGSQGPGGAGQQGPYGPGATAAAAAA

RGYGPGAGQQGPGGAGQQGPGSQGPGGAGQQGPYGPGAAAAAA

GGYGPGAGQQGPGGAGQQGPGSQGPGGAGQRGPYGPGAAAAAAAA

GGYGPGAGQQGPGSGGQQGPSGQGPYGPGASAASAAAGGYGPGAGQQGPGGQGAGSGGQQGPGSQGPGGAGQQGPYGPGAAAAAAVPGGYGPGAGQQGPGGAGQQGSGSQGPGGAGQQGPFGPGAAAAAAAAA

GGYGPGAGQQGPGSGGQQGPSGQGPYGPAASTAAAAA

GGFGPGAGQQGPGGQGAGSGGQQGPGSQGPGGAGQQGPYGPGAAAAAAAA

GGYGPGAGQQGPGGAGQQGPGSQGPGGAGQRGPYGPGAAAAAAAA

GGYGPGAGQQGPGGAGQQGPGSQGPGSAGQQGPYGPGAAAATAAVGGYGPGAGQQGPRSQAPVASAAASRLSSPQASSRVSSAVSSLVSNGPTNPAALSNTIGSVVSQISASNPGLSNCDVLVQALLEMVSALVHILGSSSIGQINYGASSQYARMVGQSVAQALG

>Aarg_MaSp2.2c

MNWSIRLALFGFVVLSTQTVFSAAQAATPWQNSQLAEQFINSFLRFIAQSGAFSPNQLDDMSSIGDTLKTAIEKMAQSRKSSKSKLQALNMAFASSMAEIAVAEQGGLSLEAKTNAIASALASAFLETTGVVNQQFVSEIKGLIYMIAQASSNEISGSAAASGGGSGGGGGGGYGQGSYASASAAAAYGSAPQGAGGPASQGPSQQGPVSQPSYGPSATVAVSAVGGRPQGQTGPSQQGPGQQGPGQQGPGQQGPYGPSAAAASAAV

GGYGPGAGQQGPGAQGPGQQGPGGQGPYGPGASAAAAAAS

GYGPGAGQQGPGSQGPSGPGQQGPGGQGPYGQGASAAAAAA

GGYGPGAGQQGPGGQGSGGQQGPGSQGPGGAGQQGPYGPGAAAAAAAA

GGYGPGAGQQGPGGQGAGSGGQQGPGSQGPGGAGQRGPYGPGAAAAAAAA

GGYGPGAGQQGPGSGGQQGPSGQGPYGPGASAAAAAAS

GYGPGAGQQGPGSQGPSGPGQQGPGSQGPYGPGASAAAAAA

GGYGPGAGQQGPGGQGSGGQQGPGSQGPGGAGQQGPYGPGAAAAAAAA

GGYGPGAGQQGPGGQGAGSGGQQGPGSQGPGGAGQRGPYGPGAAAAAAAA

GGYGPGAGQQGPGSGGQQGPSGQGPYGPGASAAAAAAS

GYGPGAGQQGPGSQGPSGPGQQGPGSQGPYGPGASAAAAAA

GGYGPGAGQQGPGGQGSGGQQGPGSQGPGGAGQQGPYGPGAAAAAAAA

GGYGPGAGQQGPGGQGAGSGGQQGPGSQGPGGAGQRGPYGPGAAAAAAAA

GGYGPGAGQQGPGSGGQQGPSGQGPYGPGASAAAAAAS

GYGPGAGQQGPGSQGPSGPGQQGPGSQGPYGPGASAAAAAA

GGYGPGAGQQGPGGQGSGGQQGPGSQGPGGAGQQGPYGPGAAAAAAAA

GGYGPGAGQQGPGGQGAGSGGQQGPGSQGPGGAGQRGPYGPGAAAAAAAA

GGYGPGAGQQGPGSGGQQGPSGQGPYGPGASAAAAAAS

GYGPGAGQQGPGSQGPSGPGQQGPGSQGPYGPGASAAAAAA

GGYGPGAGQQGPGGQGSGGQQGPGSQGPGGAGQQGPYGPGAAAAAAAA

GGYGPGAGQQGPGGQGAGSGGQQGPGSQGPGGAGQRGPYGPGAAAAAAAA

GGYGPGAGQQGPGSGGQQGPSGQGPYGPGASAAAAAAS

GYGPGAGQQGPGSQGPSGPGQQGPGSQGPYGPGASAAAAAA

GGYGPGAGQQGPGGQGSGGQQGPGSQGPGGAGQQGPYGPGAAAAAAAA

GGYGPGAGQQGPGGQGAGSGGQQGPGSQGPGGAGQRGPYGPGAAAAAAAA

GGYGPGAGQQGPGSGGQQGPSGQGPYGPGASAAAAAAS

GYGPGAGQQGPGSQGPSGPGQQGPGSQGPYGPGASAAAAAA

GGYGPGAGQQGPGGQGSGGQQGPGSQGPGGAGQQGPYGPGAAAAAAAA

GGYGPGAGQQGPGGQGAGSGGQQGPGSQGPGGAGQRGPYGPGAAAAAAAA

GGYGPGAGQQGPGSGGQQGPSGQGPYGPGASAAAAAAS

GYGPGAGQQGPGSQGPSGPGQQGPGSQGPYGPGASAAAAAA

GGYGPGAGQQGPGGQGSGGQQGPGSQGPGGAGQQGPYGPGAAAAAAAA

GGYGPGAGQQGPGGQGAGSGGQQGPGSQGPGGAGQRGPYGPGAAAAAAAA

GGYGPGAGQQGPGSGGQQGPSGQGPYGPGASAAAAAAS

GYGPGAGQQGPGSQGPSGPGQQGPGSQGPYGPGASAAAAAA

GGYGPGAGQQGPGGQGSGGQQGPGSQGPGGAGQQGPYGPGAAAAAAAA

GGYGPGAGQQGPGGQGAGSGGQQGPGSQGPGGAGQRGPYGPGAAAAAAAA

GGYGPGAGQQGPGSGGQQGPSGQGPYGPGASAAAAAAS

GYGPGAGQQGPGSQGPSGPGQQGPGSQGPYGPGASAAAAAA

GGYGPGAGQQGPGGQGSGGQQGPGSQGPGGAGQQGPYGPGAAAAAAAA

GGYGPGAGQQGPGGQGAGSGGQQGPGSQGPGGAGQRGPYGPGAAAAAAAA

GGYGPGAGQQGPGSGGQQGPSGQGPYGPGASAAAAAAS

GYGPGAGQQGPGSQGPSGPGQQGPGSQGPYGPGASAAAAAA

GGYGPGAGQQGPGGQGSGGQQGPGSQGPGGAGQQGPYGPGAAAAAAAA

GGYGPGAGQQGPGGQGAGSGGQQGPGSQGPGGAGQRGPYGPGAAAAAAAA

GGYGPGAGQQGPGSGGXQGPSGQGPYGPGASAAAAAAS

GYGPGAGQQGPGSQGPSGPGQQGPGSQGPYGPGASAAAAAA

GGYGPGAGQQGPGGQGSGGQQGPGSQGPGGAGQQGPYGPGAAAAAAAA

GGYGPGAGQQGPGGQGAGSGGQQGPGSQGPGGAGQRGPYGPGAAAAAAAA

GGYGPGAGQQGPGSGGQQGPSGQGPYGPGASAAAAAAS

GYGPGAGQQGPGSQGPSGPGQQGPGSQGPYGPGASAAAAAA

GGYGPGAGQQGPGGQGSGGQQGPGSQGPGGAGQQGPYGPGAAAAAAAA

GGYGPGAGQQGPGGQGAGSGGQQGPGSQGPGGAGQRGPYGPGAAAAAAAA

GGYGPGAGQQGPGSGGQQGPSGQGPYGPGASAAAAAAS

GYGPGAGQQGPGSQGPTGPGQQGPGSQGPYGPGASAAAAAA

GGYGPGAGQQGPGGQGSGGQQGPGSQGPGGAGQQGPYGPGAAAAAAAA

GGYGPGAGQQGPGGQGAGSGGQQGPGSQGPGGAGQRGPYGPGAAAAAAAA

GGYGPGAGQQGPGSGGQQGPSGQGPYGPGASAAAAAAS

GYGPGAGQQGPGSQGPSGPGQQGPGSQGPYGPGASAAAAAA

GGYGPGAGQQGPGGQGSGGQQGPGSQGPGGAGQQGPYGPGAAAAAAAA

GGYGPGAGQQGPGGQGAGSGGQQGPGSQGPGGAGQRGPYGPGAAAAAAAA

GGYGPGAGQQGPGSGGQQGPSGQGPYGPGASAAAAAAS

GYGPGAGQQGPGSQGPSGPGQQGPGSQGPYGPGASAAAAAA

GGYGPGAGQQGPGGQGSGGQQGPGSQGPGGAGQQGPYGPGAAAAAAAA

GGYGPGAGQQGPGGQGAGSGGQQGPGSQGPGGAGQRGPYGPGAAAAAAAA

GGYGPGAGQQGPGSGGQQGPSGQGPYGPGASAAAAAAS

GYGPGAGQQGPGSQGPSGPGQQGPGSQGPYGPGASAAAAAA

GGYGPGAGQQGPGGQGSGGQQGPGSQGPGGAGQQGPYGPGAAAAAAAA

GGYGPGAGQQGPGGQGAGSGGQQGPGSQGPGGAGQQGPYGPGAAAAAAAA

GGYGPGAGQQGPGSQGPSGPSQQGPGGQGPYGPGASAAAAAAS

GYGPGSGQQGPSGPGQQGPGSQGPYGPGPSAAAAAA

GGYGPGAGQQGPRSQAPVASAAASRLSSPQASSRVSSAVSSLVSSGPTNPAALSNTIGSVVSQVRSSNPGLSNCDVLVQALLEMVSALVHILGSSSIGQINYGASSQYAQLVGQSITQALA

>Aarg_MaSp2.2d

MNWSIRLALFGFVVLSTQTVFSAAQAATPWQNSQLAEQFINSFLRFIAQSGAFSPNQLDDMSSIGDTLKTAIEKMAQSRKSSKSKLQALNMAFASSMAEIAVAEQGGLSLEAKTNAIASALASAFLETTGVVNQQFVSEIKGLIYMIAQASSNEISGSAAASGGGSGGGGGGGYGQGSYASASAAAAYGSAPQGAGGPASQGPSQQGPVSQPSYGPSATVAVSAVGGRPQGQTGPSQQGPGQQGPGQQGPYGPSAAAASAAV

GGYGPGAGQQGQQGPGAQGPGQQGPGGQGPYGPGASAAAAAAS

GYGPGAGQQGPGSQGPSGPGQQGPGGQGPYGPGASAAAAAA

GGYGPGAGQQGPGGQGSGGQQGPGSQGPGGAGQQGPYGQGAAAAAAAA

GGYGPGAGQQGPGSGGQQGPSGQGPYGPGASAAAAAAS

GYGPGAGQQGPGSQGPSGPGQQGPGSQGPYGPGASAAAAAA

GGYGPGAGQQGPGGQGSGGQQGPGSQGPGGAGQQGPYGPGAAAAAAAA

GGYGPGAGQQGPGGQGAGSGGQQGPGSQGPGGAGQRGPYGPGAAAAAAAA

GGYGPGAGQQGPGSGGQQGPSGQGPYGPGASAAAAAAS

GYGPGAGQQGPGSQGPSGPGQQGPGSQGPYGPGASAAAAAA

GGYGPGAGQQGPGGQGSGGQQGPGSQGPGGAGQQGPYGPGAAAAAAAA

GGYGPGXGQQGPGGQGAGSGGQQGPGSQGPGGAGQRGPYGPGAAAAAAAA

GGYGPGAGQQGPGSGGQQGPSGQGPYGPGASAAAAAAS

GYGPGAGQQGPGSQGPSGPGQQGPGSQGPYGPGASAAAAAA

GGYGPGAGQQGPGGQGSGGQQGPGSQGPGGAGQQGPYGPGAAAAAAAA

GGYGPGAGQQGPGGQGAGSGGQQGPGSQGPGGAGQRGPYGPGAAAAAAAA

GGYGPGAGQQGPGSGGQQGPSGQGPYGPGASAAAAAAS

GYGPGAGQQGPGSQGPSGPGQQGPGSQGPYGPGASAAAAAA

GGYGPGAGQQGPGGQGSGGQQGPGSQGPGGAGQQGPYGPGAAAAAAAA

GGYGPGAGQQGPGGQGAGSGGQQGPGSQGPGGAGQRGPYGPGAAAAAAAA

GGYGPGAGQQGPGSGGQQGPSGQGPYGPGASAAAAAAS

GYGPGAGQQGPGSQGPSGPGQQGPGSQGPYGPGASAAAAAA

GGYGPGAGQQGPGGQGSGGQQGPGSQGPGGAGQQGPYGPGAAAAAAAA

GGYGPGAGQQGPGGQGAGSGGQQGPGSQGPGGAGQRGPYGPGAAAAAAAA

GGYGPGAGQQGPGSGGQQGPSGQGPYGPGASAAAAAAS

GYGPGAGQQGPGSQGPSGPGQQGPGSQGPYGPGASAAAAAA

GGYGPGAGQQGPGGQGSGGQQGPGSQGPGGAGQQGPYGPGAAAAAAAA

GGYGPGAGQQGPGGQGAGSGGQQGPGSQGPGGAGQRGPYGPGAAAAAAAA

GGYGPGAGQQGPGSGGQQGPSGQGPYGPGASAAAAAAS

GYGPGAGQQGPGSQGPSGPGQQGPGSQGPYGPGASAAAAAA

GGYGPGAGQQGPGGQGSGGQQGPGSQGPGGAGQQGPYGPGAAAAAAAA

GGYGPGAGQQGPGGQGAGSGGQQGPGSQGPGGAGQRGPYGPGAAAAAAAA

GGYGPGAGQQGPGSGGQQGPSGQGPYGPGASAAAAAAS

GYGPGAGQQGPGSQGPSGPGQQGPGSQGPYGPGASAAAAAA

GGYGPGAGQQGPGGQGSGGQQGPGSQGPGGAGQQGPYGPGAAAAAAAA

GGYGPGAGQQGPGGQGAGSGGQQGPGSQGPGGAGQRGPYGPGAAAAAAAA

GGYGPGAGQQGPGSGGQQGPSGQGPYGPGASAAAAAAS

GYGPGAGQQGPGSQGPSGPGQQGPGSQGPYGPGASAAAAAA

GGYGPGAGQQGPGGQGSGGQQGPGSQGPGGAGQQGPYGPGAAAAAAAA

GGYGPGAGQQGPGGQGAGSGGQQGPGSQGPGGAGQRGPYGPGAAAAAAAA

GGYGPGAGQQGPGSGGQQGPSGQGPYGPGASAAAAAAS

GYGPGAGQQGPGSQGPSGPGQQGPGSQGPYGPGASAAAAAA

GGYGPGAGQQGPGGQGSGGQQGPGSQGPGGAGQQGPYGPGAAAAAAAA

GGYGPGAGQQGPGGQGAGSGGQQGPGSQGPGGAGQRGPYGPGAAAAAAAA

GGYGPGAGQQGPGSGGQQGPSGQGPYGPGASAAAAAAS

GYGPGAGQQGPGSQGPSGPGQQGPGSQGPYGPGASAAAAAA

GGYGPGAGQQGPGGQGSGGQQGPGSQGPGGAGQQGPYGPGAAAAAAAA

GGYGPGAGQQGPGGQGAGSGGQQGPGSQGPGGAGQRGPYGPGAAAAAAAA

GGYGPGAGQQGPGSGGQQGPSGQGPYGPGASAAAAAAS

GYGPGAGQQGPGSQGPSGPGQQGPGSQGPYGPGASAAAAAA

GGYGPGAGQQGPGGQGSGGQQGPGSQGPGGAGQQGPYGPGAAAAAAAA

GGYGPGAGQQGPGGQGAGSGGQQGPGSQGPGGAGQRGPYGPGAAAAAAAA

GGYGPGAGQQGPGSGGQQGPSGQGPYGPGASAAAAAAS

GYGPGAGQQGPGSQGPSGPGQQGPGSQGPYGPGASAAAAAA

GGYGPGAGQQGPGGQGSGGQQGPGSQGPGGAGQQGPYGPGAAAAAAAA

GGYGPGAGQQGPGGQGAGSGGQQGPGSQGPGGAGQRGPYGPGAAAAAAAA

GGYGPGAGQQGPGSGGQQGPSGQGPYGPGASAAAAAAS

GYGPGAGQQGPGSQGPSGPGQQGPGSQGPYGPGASAAAAAA

GGYGPGAGQQGPGGQGSGGQQGPGSQGPGGAGQQGPYGPGAAAAAAAA

GGYGPGAGQQGPGGQGAGSGGQQGPGSQGPGGAGQRGPYGPGAAAAAAAA

GGYGPGAGQQGPGSGGQQGPSGQGPYGPGASAAAAAAS

GYGPGAGQQGPGSQGPSGPGQQGPGSQGPYGPGASAAAAAA

GGYGPGAGQQGPGGQGSGGQQGPGSQGPGGAGQQGPYGPGAAAAAAAA

GGYGPGAGQQGPGGQGAGSGGQQGPGSQGPGGAGQRGPYGPGAAAAAAAA

GGYGPGAGQQGPGSGGQQGPSGQGPYGPGASAAAAAAS

GYGPGAGQQGPGSQGPSGPGQQGPGSQGPYGPGASAAAAAA

GGYGPGAGQQGPGGQGSGGQQGPGSQGPGGAGQQGPYGPGAAAAAAAA

GGYGPGAGQQGPGGQGAGSGGQQGPGSQGPGGAGQRGPYGPGAAAAAAAA

GGYGPGAGQQGPGSGGQQGPSGQGPYGPGASAAAAAAS

GYGPGAGQQGPGSQGPSGPGQQGPGSQGPYGPGASAAAAAA

GGYGPGAGQQGPGGQGSGGQQGPGSQGPGGAGQQGPYGPGAAAAAAAA

GGYGPGAGQQGPGGQGAGSGGQQGPGSQGPGGAGQRGPYGPGAAAAAAAA

GGYGPGAGQQGPGSGGQQGPSGQGPYGPGASAAAAAAS

GYGPGAGQQGPGSQGPSGPGQQGPGSQGPYGPGASAAAAAA

GGYGPGAGQQGPGGQGSGGQQGPGSQGPGGAGQQGPYGPGAAAAAAAA

GGYGPGAGQQGPGGQGAGSGGQQGPGSQGPGGAGQQGPYGPGAAAAAAAA

GGYGPGAGQQGPGSQGPSGPSQQGPGGQGPYGPGASAAAAAAS

GYGPGSGQQGPSGPGQQGPGSQGPYGPGPSAAAAAA

GGYGPGAGQQGPRSQAPVASAAASRLSSPQASSRVSSAVSSLVSSGPTNPAALSNTIGSVVSQVRSSNPGLSNCDVLVQALLEMVSALVHILGSSSIGQINYGASSQYAQLVGQSITQALA

>Aaur_MaSp2.2a

MNWSIRLALLGFVVLSTQTIFAAGQAATPWENTQLAEDFIISFLRFIGQSGAFSPDQLDDMSTIGETLKTAIEKMAQSRKSSKSKLQALNMAFASSMAEIAVAEKGGLSLEAKTNAIANALASAFLETTGFVNQQFVSEIKSLIYMIAQASANEISGSAAAAGGGSGGFGSGQGGYGQGAYASASAASAYGSAPQGAGGPAPQGLSQQGPVRQGPYGPSAAVAATAVGGRPQGRSASSQQGPSQQGPYGPGAAGAAAAA

GGYGPGVGQQGPGDAGQQGPYGPGAAAV

GGYGPGARAGGPQRPIGAGPSLPSARGPQGPGGSGPGSQGPFEPAAAAAAAAAA

RGFGPGASGQKGPGEAGQQGPGGAGQQGPGGQGLFGPGAAAAAAAAA

GGFGPGAGGQRGPGQQGPGGQGPSGPGAAAAAAAAA

GGFGPGGAGAGPQAGQRGPGGAGAGAAAAAAA

GAGGFGPGAGGQQGPGGAGPYGPSAGGQRGPGGVGQQGPGGQGPFGPGAAAAAAAAA

GGFGPGGAGVGPQAAPGQQGPGGAGPYGPGAAAAAAAA

GGFGPGAGGQRGPGQQGLFGPGAAAAAAAAA

GGFGPGAGGQKVPGGAGQQGPGGQGPYGPGAAAAAAAA

GGFGPGAGGQRGPGQQGPGGQGPSGPGAAAAAAAAAA

GGFGPGGAGAGPQAGQRGPGGAGAGAAAAAAA

GAGGFGPGAGGQQGPGGAGPYGPSAGGQRGPGGVGQQGPGGQGPFGPGAAAAAAAAA

GGFGPGGAGVGPQAAPGQQGPGGAGPYGPGAAAAAAAAAA

GGFGPGAGGQRGPGQQGLFGPGAAAAAAAAA

GGFGPGAGGQKVPGGAGQQGPGGQGPYGPGAAAAAAAA

GGFGPGAGGQRGPGQQGPGGQGPSGPGAAAAAAAAAA

GGFGPGGAGAGPQAGQRGPGGAGAGAAAAAAA

GAGGFGPGAGGQQGPGGAGPYGPSAGGQRGPGGVGQQGPGGQGPFGPGAAAAAAAAA

GGFGPGGAGVGPQAAPGQQGPGGAGPYGPGAAAAAAAAAA

GGFGPGAGGQRGPGQQGLFGPGAAAAAAAAA

GGFGPGAGGQKVPGGAGQQGPGGQGPYGPGAAAAAAAA

GGFGPGAGGQRGPGQQGPGGQGPSGPGAAAAAAAAAA

GGFGPGGAGAGPQAGQRGPGGAGAGAAAAAAA

GAGGFGPGAGGQQGPGGAGPYGPSAGGQRGPGGVGQQGPGGQGPFGPGAAAAAAAAA

GGFGPGGAGVGPQAAPGQQGPGGAGPYGPGAAAAAAAAAA

GGFGPGAGGQRGPGQQGLFGPGAAAAAAAAA

GGFGPGAGGXKVPGGAGQQGPGGQGPYGPGAAAAAAAA

GGFGPGAGGQRGPGQQGPGGQGPSGPGAAAAAAAAAA

GGFGPGGAGAGPQAGQRGPGGAGAGAAAAAAA

GAGGFGPGAGGQQGPGGAGPYGPSAGGQRGPGGVGQQGPGGQGPFGPGAAAAAAAAA

GGFGPGGAGVGPQAAPGQQGPGGAGPYGPGAAAAAAAA

GGFGPGAGGQRGPGQQGLFGPGAAAAAAAAA

GGFGPGAGGXKVPGGAGQQGPGGQGPYGPGAAAAAAAA

GGFGPGAGGQRGPGQQGPGGQGPSGPGAAAAAAAAAA

GGFGPGGAGAGPQAGQRGPGGAGAGAAAAAAA

GAGGFGPGAGGQQGPGGAGPYGPSAGGQRGPGGVGQQGPGGQGPFGPGAAAAAAAAA

GGFGPGGAGVGPQAAPGQQGPGGAGPYGPGAAAAAAAAAA

GGFGPGAGGQRGPGQQGLFGPGAAAAAAAAA

GGFGPGAGGXKVPGGAGQQGPGGQGPYGPGAAAAAAAA

GGFGPGAGGQRGPGQQGPGGQGPSGPGAAAAAAAAAA

GGFGPGGAGAGPQAGQRGPGGAGAGAAAAAAA

GAGGFGPGAGGQQGPGGAGPYGPSAGGQRGPGGVGQQGPGGQGPFGPGAAAAAAAAA

GGFGPGGAGVGPQAAPGQQGPGGAGPYGPGAAAAAAAAAA

GGFGPGAGGQRGPGQQGLFGPGAAAAAAAAA

GGFGPGAGGQKVPGGAGQQGPGGQGPYGPGAAAAAAAA

GGFGPGAGGQRGPGQQGPGGQGPSGPGAAAAAAAAAA

GGFGPGGAGAGPQAGQRGPGGAGAGAAAAAAA

GAGGFGPGAGGQQGPGGAGPYGPSAGGQRGPGGVGQQGPGGQGPFGPGAAAAAAAAA

GGFGPGGAGVGPQAAPGQQGPGGAGPYGPGAAAAAAAAAA

GGFGPGAGGQRGPGQQGLFGPGAAAAAAAAA

GGFGPGAGGQKVPGGAGQQGPGGQGPYGPGAAAAAAAA

GGFGPGAGGQRGPGQQGPGGQGPSGPGAAAAAAAAAA

GGFGPGGAGAGPQAGQRGPGGAGAGAAAAAAA

GAGGFGPGAGGQQGPGGAGPYGPSAGGQRGPGGVGQQGPGGQGPFGPGAAAAAAAAA

GGFGPGGAGVGPQAAPGQQGPGGAGPYGPGAAAAAAAAAA

GGFGPGAGGQRGPGQQGLFGPGAAAAAAAAA

GGFGPGAGGQKVPGGAGQQGPGGQGPYGPGAAAAAAAA

GGFGPGAGGQRGPGQQGPGGQGPSGPGAAAAAAAAAA

GGFGPGGAGAGPQAGQRGPGGAGAGAAAAAAA

GAGGFGPGAGGQQGPGGAGPYGPSAGGQRGPGGVGQQGPGGQGPFGPGAAAAAAAAA

GGFGPGGAGVGPQAAPGQQGPGGAGPYGPGAAAAAAAAAA

GGFGPGAGGQRGPGQQGLFGPGAAAAAAAAA

GGFGPGAGGQKVPGGAGQQGPGGQGPYGPGAAAAAAAA

GGFGPGAGGQRGPGQQGPGGQGPSGPGAAAAAAAAAA

GGFGPGGAGAGPQAGQRGPGGAGAGAAAAAAA

GAGGFGPGAGGQQGPGGAGPYGPSAGGQRGPGGVGQQGPGGQGPFGPGAAAAAAAAA

GGFGPGGAGVGPQAAPGQQGPGGAGPYGPGAAAAAAAAAA

GGFGPGAGGQRGPGQQGLFGPGAAAAAAAAA

GGFGPGAGGQKVPGGAGQQGPGGQGPYGPGAAAAAAAA

GGFGPGAGGQRGPGQQGPGGQGPSGPGAAAAAAAAAA

GGFGPGGAGAGPQAGQRGPGGAGAGAAAAAAA

GAGGFGPGAGGQQGPGGAGPYGPSAGGQRGPGGVGQQGPGGQGPFGPGAAAAAAAAA

GGFGPGGAGVGPQAAPGQQGPGGAGPYGPGAAAAAAAAAA

GGFGPGAGGQRGPGQQGLFGPGAAAAAAAAA

GGFGPGAGGQKVPGGAGQQGPGGQGPYGPGAAAAAAAA

GGFGPGAGGQRGPGQQGPGGQGPSGPGAAAAAAAAAA

GGFGPGGAGAGPQAGQRGPGGAGAGAAAAAAA

GAGGFGPGAGGQQGPGGAGPYGPSAGGQRGPGGVGQQGPGGQGPFGPGAAAAAAAAA

GGFGPGGAGVGPQAAPGQQGPGGAGPYGPGAAAAAAAAAA

GGFGPGAGGQRGPGQQGLFGPGAAAAAAAAA

GGFGPGAGGQKVPGGAGQQGPGGQGPYGPGAAAAAAAA

GGFGPGAGGQRGPGQQGPGGQGPSGPGAAAAAAAAAA

GGFGPGGAGAGPQAGQRGPGGAGAGAAAAAAA

GAGGFGPGAGGQQGPGGAGPYGPSAGGQRGPGGVGQQGPGGQGPFGPGAAAAAAAAA

GGFGPGGAGVGPQAAPGQQGPGGAGPYGPGAAAAAAAA

GGFGPGAGGQRGPGQQGPGGQGLFGPGAAAAAAAAA

GGFGPGAGGQKGPGGAGQQGPGGQGPYGPGAAAAAAAA

GGFGPGTGGQRGPGQQVPGGQGPSGPGAAAAAAAAA

GGFGPGGAGPGPKAGQGGARFYRPGAAVATAAVGGYGPGAGQQGPAAPSQQGPGRQIPYGPGAAAAVVGVYAPVPQRPTASAAASRLASPEASSRVSSAVSSLVSSGPTNPAALSNTISSVVSQISASNPGLSGCDVLVQALLEIVSALVHILGYSSIGQINYGAASQYARLVGQSVAQALG

>Aaur_MaSp2.2b

MNWSIRLALLGFVVLSTQTVFSAGQGATPWENSQLAEDFINSFLRFIAQSGAFSPNQLDDMSSIGDTLKTAIEKMAQSRKSSKSKLQALNMAFASSMAEIAVAEQGGLSLEAKTNAIANALTSAFLETTGVVNQQFVSEIKSLIYMIAQASSNEISGSAAAAGGGSGGGGGSGQGGYGQGAYASASAAAAY

GSAPQGAGGPAPQGPSQQGPVSQGPYGPGAAAAAAAA

GGYGPGAGQQRQQGPGRQGKAGAGQQGPGGQGPYGPSAAAAAAAA

GGYGQGAGQQGPGGAGQQGPGSQRPGGAGQQGPGGQGPYGPAAAAAAAAV

GGYGPGAGQQGPGSQGPGSGGQQGPGSGGQQGPGGQGPYGSGQQGPGGAGQQGPGGQGPYGPGAAAAAAAA

GGYGPGAGQQGPGGAGQQGPGSQGPGGAGQRGPGGQGPYGPGAAAAAAAA

GGYGPGAGQQGPGSQGPGSGGQQGPGGQGPYGPSAAAAAAAA

GGYGPGAGQQGRGSGGQQGPGSGGQQGPGGQGPYGSGQQGPGGAGQQGPGGQGPYGPGAAAAAAAA

GGYGPGAGQQGPGGAGQQGPGSQGPGGAGQRGPGGQGPYGPGAAAAAAAAA

GYGPGAGQQGPGSQGPGSGGQQGPGGQGPYGPSAAAAAAAA

GGYGPGAGQQGRGSGGQQGPGSGGQQGPGGQGPYGSGQQGPGGAGQQGPGGQGPYGPGAAAAAAAA

GGYGPGAGQQGPGGAGQQGPGGAGQQGPGSQGPGGAGQRGPGGQGPYGPGAAAAAAAAA

GYGPGAGQQGPGSQGPGSGGQQGPGGQGPYGPSAAAAAAAA

GGYGPGAGQQGRGSGGQQGPGSGGQQGPGGQGPYGSGQQGPGGAGQQGPGGQGPYGPGAAAAAAAA

GGYGPGAGQQGPGGAGQQGPGGAGQQGPGGAGQQGPGSQGPGGAGQRGPGGQGPYGPGAAAAAAAA

GGYGPGAGQQGPGSQGPGSGGQQGPGGQGPYGPSAAAAAAAA

GGYGPGAGQQGRGSGGQQGPGSGGQQGPGGQGPYGSGQQGPGGAGQQGPGGQGPYGPGAAAAAAAA

GGYGPGAGQQGPGGAGQQGPGSQGPGGAGQRGPGGQGPYGPGAAAAAAAA

GGYGPGAGQQGPGGAGQQGPGGAGQQGPGSQGPGGAGQRGPGGQGPYGPGAAAAAAAA

GGYGPGAGQQGPGSQGPGSGGQQGPGGQGPYGPSAAAAAAAA

GGYGPGAGQQGPGGAGQQGPGSQGPGGAGQRGPGGQGPYGPGAAAAAAAA

GGYGPGAGQQGPGSQGPGSGGQQGPGGQGPYGPSAAAAAAAA

GGYGPGAGQQGPGSQGPGSGGQQGPGGQGPYGPSAAAAAAAA

GGYGPGAGQQGPGSGGQQGPGGQGPYGSGQQGPGGAGQQGPGGQGPYGPGAAAAAAAA

GGYGPGAGQQGPGGAGQQGPGGAGQQGPGSQGPGGAGQRGPGGQGPYGPGAAAAAAAA

GGYGPGAGQQGPGSQGPGSGGQQGPGGQGPYGPSAAAAAAAA

GGYGPGAGQQGPGGAGQQGPGSQGPGGAGQRGPGGQGPYGPGAAAAAAAA

GGYGPGAGQQGPGSQGPGSGGQQGPGGQGPYGPSAAAAAAAA

GGYGPGAGQQGPGSQGPGSGGQQGPGGQGPYGPSAAAAAAAA

GGYGPGAGQQGPGSGGQQGPGGQGPYGSGQQGPGGAGQQGPGGQGPYGPGAAAAAAAA

GGYGPGAGQQGPGGAGQQGPGSQGPGGAGQRGPGGQGPYGPGAAAAAAAA

GGYGPGAGQQGPGSQGPGSGGQQGPGGQGTYGPSAAAAAAAA

GGYGPGAGQQGPGGAGQQGPGSQGPGGAGQRGPGGQGPYGPGAAAAAAAA

GGYGPGAGQQGPGSQGPGSGGQQGPGGQGPYGPSAAAAAAAA

GGYGPGAGQQGPGSGGQQGPGGQGPYGSGQQGPGGAGQQGPGGQGPYGPGAAAAAAAA

GGYGPGAGQQGPGGAGQQGPGSQGPGGAGQRGPGGQGPYGPGAAAAAAAA

GGYGPGAGQQGPGSQGPGSGGQQGPGGQGPYGPSAAAAAAAA

GGYGPGAGQQGPGGAGQQGPGSQGPGGAGQRGPGGQGPYGPGAAAAAAAA

GGYGPGAGQQGPGSQGPGSGGQQGPGGQGPYGPSAAAAAAAA

GGYGPGAGQQGPGSQGPGSGGQQGPGGQGPYGPSAAAAAAAA

GGYGPGAGQQGPGSGGQQGPGGQGPYGSGQQGPGGAGQQGPGGQGPYGPGAAAAAAAA

GGYGPGAGQQGPGGAGQQGPGSQGPGGAGQRGPGGQGPYGPGAAAAAAAA

GGYGPGAGQQGPGSQGPGSGGQQGPGGQGPYGPSAAAAAAAA

GGYGPGAGQQGPGGAGQQGPGSQGPGGAGQRGPGGQGPYGPGAAAAAAAA

GGYGPGAGQQGPGSQGPGSGGQQGPGGQGPYGPSAAAAAAAA

GGYGPGAGQQGPGSQGPGSGGQQGPGGQGPYGPSAAAAAAAA

GGYGPGAGQQGPGSGGQQGPGGQGPYGSGQQGPGGAGQQGPGGQGPYGPGAAAAAAAA

GGYGPGAGQQGPGGAGQQGPGSQGPGGAGQRGPGGQGPYGPGAAAAAAAA

GGYGPGAGQQGPGSQGPGSGGQQGPGGQGPYGPSAAAAAAAA

GGYGPGAGQQGPGGAGQQGPGSQGPGGAGQRGPGGQGPYGPGAAAAAAAA

GGYGPGAGQQGPGSQGPGSGGQQGPGGQGPYGPSAAAAAAAA

GGYGPGAGQQGPGSQGPGSGGQQGPGGQGPYGPSAAAAAAAA

GGYGPGAGQQGPGSGGQQGPGGQGPYGSGQQGPGGAGQQGPGGQGPYGPGAAAAAAAA

GGYGPGAGQQGPGGAGQQGPGSQGPGGAGQRGPGGQGPYGPGAAAAAAAA

GGYGPGAGQQGPGSQGPGSGGQQGPGGQGPYGPSAAAAAAAA

GGYGPGAGQQGPGGAGQQGPGSQGPGGAGQRGPGGQGPYGPGAAAAAAAA

GGYGPGAGQQGPGSQGPGSGGQQGPGGQGPYGPSAAAAAAAA

GGYGPGAGQQGPGSQGPGSGGQQGPGGQGPYGPSAAAAAAAA

GGYGPGAGQQGPGSGGQQGPGGQGPYGSGQQGPGGAGQQGPGGQGPYGPGAAAAAAAA

GGYGPGAGQQGPGGAGQQGPGSQGPGGAGQRGPGGQGPYGPGAAAAAAAA

GGYGPGAGQQGPGSQGPGSGGQQGPGGQGPYGPSAAAAAAAA

GGYGPGAGQQGPGGAGQQGPGSQGPGGAGQRGPGGQGPYGPGAAAAAAAA

GGYGPGAGQQGPGSQGPGSGGQQGPGGQGPYGPSAAAAAAAA

GGYGPGAGQQGPGSQGPGSGGQQGPGGQGPYGPSAAAAAAAA

GGYGPGAGQQGPGSGGQQGPGGQGPYGSGQQGPGGAGQQGPGGQGPYGPGAAAAAAAA

GGYGPGAGQQGPGGAGQQGPGSQGPGGAGQRGPGGQGPYGPGAAAAAAAA

GGYGPGAGQQGPGSQGPGSGGQQGPGGQGPYGPSAAAAAAAA

GGYGPGAGQQGPGGAGQQGPGSQGPGGAGQRGPGGQGPYGPGAAAAAAAA

GGYGPGAGQQGPGSQGPGSGGQQGPGGQGPYGPSAAAAAAAA

GGYGPGAGQQGPGSQGPGSGGQQGPGGQGPYGPSAAAAAAAA

GGYGPGAGQQGPGSGGQQGPGGQGPYGSGQQGPGGAGQQGPGGQGPYGPGAAAAAAAA

GGYGPGAGQQGPGGAGQQGPGGAGQQGPGSQGPGGAGQQGPGGQGPYGPGAAAAAAAA

GGYGPGAGQQGPGSQGPGSGGQQGPGGQGPYGPSAAAAAAAA

GGYGPGAGQQGPGGAGQQGLGSQGPGGAGQRGPGGQGPYGPGAAAAAAAA

GGYGPGAGQQGPGSQGPVASAAASRLSSPQASSRVSSAVSTLVSSGPTNPAALSNAISNVVSQVSASNPGLSGCDVLVQALLEIVSALVHILGSSSIGQINYAASSQYAQMVGNSVTQALG

>Aaur_MaSp2.2c

MNWSIRLALLGFVVLSTQTVFXAGQGATPWENSQLAEDFINSFLRFIAQSGAFSPNQLDDMSSIGDTLKTAIEKMAQSRKSSKSKLQALNMAFASSMAEIAVAEQGGLSLEAKTNAIANALTSAFLETTGVVNQQFVSEIKSLIYMIAQASSNEISGSAAAAGGGSGGGGGSGQGGYGQGAYASASAAAAYGSAPQGAGGPAPQGPSQQGPVSQGPYGPGAAAAAAAA

GGYGPGAGQQRQQGPGRQGKAGAGQQGPGGQGPYGPSAAAAAAAA

GGYGPGAGQQGPGSQGPGAGQQGPGSQGPGSGGQQGPGGQGPYGPSAAAAAAAA

GGYGSGAGQQGPGSQGPGSGGQQGPGGQGPYGPSAAAAAAAA

GGYGPGAGQQGPGSGGQQGPGGQGPYGSGQQGPGGAGQQGPGGQGPYGPGAAAAAAAA

GGYGPGAGQQGPGGAGQQGPGSQGPGGAGQRGPGGQGPYGPGAAAAAAAA

GGYGPGAGQQGPGSQGPGSGGQQGPGGQGPYGPSAAAAAAAA

GGYGPGAGQQGPGSGGQQGPGGQGPYGSGQQGPGGAGQQGPGGQGPYGPGAAAAAAAA

GGYGPGAGQQGPGGAGQQGPGSQGPGGAGQRGPGGQGPYGPGAAAAAAAA

GGYGPGAGQQGPGSQGPGSGGQQGPGGQGPYGPSAAAAAAAA

GGYGPGAGQQGPGSQGPGSGGQQGPGGQGPYGPSAAAAAAAA

GGYGPGAGQQGPGSGGQQGPGGQGPYGSGQQGPGGAGQQGPGGQGPYGPGAAAAAAAA

GGYGPGAGQQGPGGAGQQGPGSQGPGGAGQRGPGGQGPYGPGAAAAAAAA

GGYGPGAGQQGPGSGGQQGSGGQGPYGPSAAAAAAAA

GGYGPRAGQQGPGSQGPGGAGQQGPGGQGPYGPGAAAAAAAA

GGYGPGAGQQGPGSQGPGSGGQQGPGGQGPYGPSAAAAAAAA

GGYGPGAGQQGPGSQGPGSGGQQGPGGQGPYGPSAAAAAAAA

GGYGPGAGQQGPGSGGQQGPGGQGPYGSGQQGPGGAGQQGPGGQGPYGPGAAAAAAAA

GGYGPGAGQQGPGGAGQQGPGSQGPGGAGQQGPGGQGPYGPGAAAAAAAA

GGYGPGAGQQGPGSQGPGSGGQQGPGGQGPYGPSAAAAAAAA

GGYGPGAGQQGPGSQGPGSGGQQGPGGQGPYGPSAAAAAAAA

GGYGPGAGQQGPGSGGQQGPGGQGPYGSGQQGPGGAGQQGPGGQGPYGPGAAAAAAAA

GGYGPGAGQQGPGGAGQQGPGSQGPGGAGQRGPGGQGPYGPGAAAAAAAA

GGYGPGAGQQGPGSGGQQGSGGQGPYGPSAAAAAAAA

GGYGPRAGQQGPGSQGPGGAGQQGPGGQGPYGPGAAAAAAAA

GGYGPGAGQQGPGSQGPGSGGQQGPGGQGPYGPSAAAAAAAA

GGYGPGAGQQGPGSQGPGSGGQQGPGGQGPYGPSAAAAAAAA

GGYGPGAGQQGPGSGGQQGPGGQGPYGSGQQGPGGAGQQGPGGQGPYGPGAAAAAAAA

GGYGPGAGQQGPGGAGQQGPGSQGPGGAGQQGPGGQGPYGPGAAAAAAAA

GGYGPGAGQQGPGSQGPGSGGQQGPGGQGPYGPSAAAAAAAA

GGYGPGAGQQGPGSQGPGSGGQQGPGGQGPYGPSAAAAAAAA

GGYGPGAGQQGPGSGGQQGPGGQGPYGSGQQGPGGAGQQGPGGQGPYGPGAAAAAAAA

GGYGPGAGQQGPGGAGQQGPGSQGPGGAGQRGPGGQGPYGPGAAAAAAAA

GGYGPGAGQQGPGSGGQQGPGGQGPYGPSAAAAAAAA

GGYGPRAGQQGPGSQGPGGAGQQGPGGQGPYGPGAAAAAAAA

GGYGPGAGQQGPGSQGPGSGGQQGPGGQGPYGPSAAAAAAAA

GGYGPGAGQQGPGSQGPGSGGQQGPGGQGPYGPSAAAAAAAA

GGYGPGAGQQGPGSGGQQGPGGQGPYGSGQQGPGGAGQQGPGGQGPYGPGAAAAAAAA

GGYGPGAGQQGPGGAGQQGPGSQGPGGAGQRGPGGQGPYGPGAAAAAAAA

GGYGPGAGQQGPGSGGQQGSGGQGPYGPSAAAAAAAA

GGYGPRAGQQGPGSQGPGGAGQQGPGGQGPYGPGAAAAAAAA

GGYGPGAGQQGPGSQGPGSGGQQGPGGQGPYGPSAAAAAAAA

GGYGPGAGQQGPGSQGPGSGGQQGPGGQGPYGPSAAAAAAAA

GGYGPGAGQQGPGSGGQQGPGGQGPYGSGQQGPGGAGQQGPGGQGPYGPGAAAAAAAA

GGYGPGAGQQGPGGAGQQGPGSQGPGGAGQQGPGGQGPYGPGAAAAAAAA

GGYGPGAGQQGPGSQGPGSGGQQGPGGQGPYGPSAAAAAAAA

GGYGPGAGQQGPGSQGPGSGGQQGPGGQGPYGPSAAAAAAAA

GGYGPGAGQQGPGSGGQQGPGGQGPYGSGQQGPGGAGQQGPGGQGPYGPGAAAAAAAA

GGYGPGAGQQGPGGAGQQGPGSQGPGGAGQRGPGGQGPYGPGAAAAAAAA

GGYGPGAGQQGPGSGGQQGSGGQGPYGPSAAAAAAAA

GGYGPRAGQQGPGSQGPGGAGQQGPGGQGPYGPGAAAAAAAA

GGYGPGAGQQGPGSQGPGSGGQQGPGGQGPYGPSAAAAAAAA

GGYGPGAGQQGPGSQGPGSGGQQGPGGQGPYGPSAAAAAAAA

GGYGPGAGQQGPGSGGQQGPGGQGPYGSGQQGPGGAGQQGPGGQGPYGPGAAAAAAAA

GGYGPGAGQQGPGGAGQQGPGSQGPGGAGQQGPGGQGPYGPGAAAAAAAA

GGYGPGAGQQGPGSQGPGSGGQQGPGGQGPYGPSAAAAAAAA

GGYGPGAGQQGPGSQGPGSGGQQGPGGQGPYGPSAAAAAAAA

GGYGPGAGQQGPGSGGQQGPGGQGPYGSGQQGPGGAGQQGPGGQGPYGPGAAAAAAAA

GGYGPGAGQQGPGGAGQQGPGSQGPGGAGQQGPGGQGPYGPGAAAAAAAA

GGYGPGAGQQGPGSQGPGSGGQQGPGGQGPYGPSAAAAAAAA

GGYGPGAGQQGPGSQGPGSGGQQGPGGQGPYGPSAAAAAAAA

GGYGPGAGQQGPGSGGQQGPGGQGPYGSGQQGPGGAGQQGPGGQGPYGPGAAAAAAAA

GGYGPGAGQQGPGGAGQQGPGSQGPGGAGQQGPGGQGPYGPGAAAAAAAA

GGYGPGAGQQGPGSQGPGSGGQQGPGGQGPYGPSAAAAAAAA

GGYGPGAGQQGPGSQGPGSGGQQGPGGQGPYGPSAAAAAAAA

GGYGPGAGQQGPGSGGQQGPGGQGPYGSGQQGPGGAGQQGPGGQGPYGPGAAAAAAAA

GGYGPGAGQQGPGGAGQQGPGSQGPGGAGQQGPGGQGPYGPGAAAAAAAA

GGYGPGAGQQGPGSQGPGSGGQQGPGGQGPYGPSAAAAAAAA

GGYGPGVGQQGPGSQGPGSGGQQGPGGQGPYGPSAAAAAAAA

GGYGPGAGQQGPGSGGQQGPGGQGPYGSGQQGPGGAGQQGPGGQGPYGPSAAAAAAAA

GGYGPGAGQQVLGSQGPVASAAASRLSSPQASSRVSSAVSTLVSSGPTNPAALSNAISNVVSQVSASNPGLSGCDVLVQALLEIVSALVHILGSSSIGQINYAASSQYAQMVGNSVTQALG

>Aaur_MaSp2.2d

MNWSIRLALLGFVVLSTQTVFSAGQGATPWENSQLAEEFINSFLRFIAQSGAFSPNQLDDMSSIGDTLKTAIEKMAQSRKSSKSKLQALNMAFASSMAEIAVAEQGGLSLESKTNAIANALASAFLETTGFVNQQFVSEIKSLIYMIAQASSNEISGSAAAAGGGSGGGGGSGQGGYGQGAYASASAAAAYGSAPQGAGGPAPQGPSQQGPVSQGPYGPGAAVAAAAA

GGYGPGAGQQGQQGPGRQGNAGPGQQGPGGQGPYGPSAAAAAAAA

GGYGPGAGQQGPGGAGQQGPGSQGPGGAGQQGPGGQGPYGPGAAAAAAAV

GGYGPGAGQQGPGSQGPGSGGQQGPGGQGPYGPSAAAAAAAA

GGYGPGAGQQGPGSQGPGSGGQQGPGGQGPYGPSAAAAAAAA

GGYGPGAGQQGPGSGGQQGPGGQGPYGSGQQGPGGAGQQGPGGQGPYGPGAAAAAAAA

GGYGPGAGQQGPGGAGQQGPGSQGPGGAGQQGPGGQGPYGPGAAAAAAAV

GGYGPGAGQQGPGSQGPGRGGQQGPGGQGPYGPSAAAAAAAA

GGYGPGAGQQGPGSQGPGSGGQQGPGGQGPYGPSAAAAAAAA

GGYGPGAGQQGPGSQGPGSGGQQGPGGQGPYGPSAAAAAAAA

GGYGPGAGQQGPGSGGQQGPGGQGPYGSGQQGPGGAGQQGPGGQGPYGPGAAAAAAAA

GGYGPGAGQQGPGGAGQQGPGSQGPGGAGQQGPGGQGPYGPGAAAAAAAV

GGYGPGAGQQGPGSQGPGSGGQQGPGGQGPYGPSAAAAAAAA

GGYGPGAGQQGPGSQGPGSGGQQGPGGQGPYGPSAAAAAAAA

GGYGPGAGQQGPGSGGQQGPGGQGPYGSGQQGPGGAGQQGPGGQGPYGPGAAAAAAAA

GGYGPGAGQQGPGGAGQQGPGSQGPGGAGQQGPGGQGPYGPGAAAAAAAV

GGYGPGAGQQGPGSQGPGSGGQQGPGGQGPYGPSAAAAAAAA

GGYGPGAGQQGPGSQGPGSGGQQGPGGQGPYGPSAAAAAAAA

GGYGPGAGQQGPGSGGQQGPGGQGPYGSGQQGPGGAGQQGPGGQGPYGPGAAAAAAAA

GGYGPGAGQQGPGGAGQQGPGSQGPGGAGQQGPGGQGPYGPGAAAAAAAV

GGYGPGAGQQGPGSQGPGSGGQQGPGGQGPYGPSAAAAAAAA

GGYGPGAGQQGPGSQGPGSGGQQGPGGQGPYGPSAAAAAAAA

GGYGPGAGQQGPGSGGQQGPGGQGPYGSGQQGPGGAGQQGPGGQGPYGPGAAAAAAAA

GGYGPGAGQQGPGGAGQQGPGSQGPGGAGQQGPGGQGPYGPGAAAAAAAV

GGYGPGAGQQGPGSQGPGSGGQQGPGGQGPYGPSAAAAAAAA

GGYGPGAGQQGPGSQGPGSGGQQGPGGQGPYGPSAAAAAAAA

GGYGPGAGQQGPGSGGQQGPGGQGPYGSGQQGPGGAGQQGPGGQGPYGPGAAAAAAAA

GGYGPGAGQQGPGGAGQQGPGSQGPGGAGQQGPGGQGPYGPGAAAAAAAV

GGYGPGAGQQGPGSQGPGSGGQQGPGGQGPYGPSAAAAAAAA

GGYGPGAGQQGPGSQGPGSGGQQGPGGQGPYGPSAAAAAAAA

GGYGPGAGQQGPGSGGQQGPGGQGPYGSGQQGPGGAGQQGPGGQGPYGPGAAAAAAAA

GGYGPGAGQQGPGGAGQQGPGSQGPGGAGQQGPGGQGPYGPGAAAAAAAV

GGYGPGAGQQGPGSQGPGSGGQQGPGGQGPYGPSAAAAAAAA

GGYGPGAGQQGPGSQGPGSGGQQGPGGQGPYGPSAAAAAAAA

GGYGPGAGQQGPGSGGQQGPGGQGPYGSGQQGPGGAGQQGPGGQGPYGPGAAAAAAAA

GGYGPGAGQQGPGGAGQQGPGSQGPGGAGQQGPGGQGPYGPGAAAAAAAV

GGYGPGAGQQGPGSQGPGSGGQQGPGGQGPYGPSAAAAAAAA

GGYGPGAGQQGPGSQGPGSGGQQGPGGQGPYGPSAAAAAAAA

GGYGPGAGQQGPGSQGPGSGGQQGPGGQGPYGPSAAAAAAAA

GGYGPGAGQQGPGSGGQQGPGGQGPYGSGQQGPGGAGQQGPGGQGPYGPGAAAAAAAA

GGYGPGAGQQGPGGAGQQGPGSQGPGGAGQQGPGGQGPYGPGAAAAAAAV

GGYGPGAGQQGPGSQGPGSGGQQGPGGQGPYGPSAAAAAAAA

GGYGPGAGQQGPGSQGPGSGGQQGPGGQGPYGPSAAAAAAAA

GGYGPGAGQQGPGSQGPGSGGQQGPGGQGPYGPSAAAAAAAA

GGYGPGAGQQGPGSGGQQGPGGQGPYGSGQQGPGGAGQQGPGGQGPYGPGAAAAAAAA

GGYGPGAGQQGPGGAGQQGPGSQGPGGAGQQGPGGQGPYGPGAAAAAAAV

GGYGPGAGQQGPGSQGPGSGGQQGPGGQGPYGPSAAAAAAAA

GGYGPGAGQQGPGSQGPGSGGQQGPGGQGPYGPSAAAAAAAA

GGYGPGAGQQGPGSGGQQGPGGQGPYGSGQQGPGGAGQQGPGGQGPYGPGAAAAAAAA

GGYGPGAGQQGPGGAGQQGPGSQGPGGAGQQGPGGQGPYGPGAAAAAAAV

GGYGPGAGQQGPGSQGPGSGGQQGPGGQGPYGPSAAAAAAAA

GGYGPGAGQQGPGSQGPGSGGQQGPGGQGPYGPSAAAAAAAA

GGYGPGAGQQGPGSGGQQGPGGQGPYGSGQQGPGGAGQQGPGGQGPYGPGAAAAAAAA

GGYGPGAGQQGPGGAGQQGPGSQGPGGAGQQGPGGQGPYGPGAAAAAAAV

GGYGPGAGQQGPGSQGPGSGGQQGPGGQGPYGPSAAAAAAAA

GGYGPGAGQQGPGSQGPGSGGQQGPGGQGPYGPSAAAAAAAA

GGYGPGAGQQGPGSGGQQGPGGQGPYGSGQQGPGGAGQQGPGGQGPYGPGAAAAAAAA

GGYGPGAGQQGPGGAGQQGPGSQGPGGAGQQGPGGQGPYGPGAAAAAAAV

GGYGPGAGQQGPGSQGPGSGGQQGPGGQGPYGPSAAAAAAAA

GGYGPGAGQQGPGSQGPGSGGQQGPGGQGPYGPSAAAAAAAA

GGYGPGAGQQGPGSGGQQGPGGQGPYGSGQQGPGGAGQQGPGGQGPYGPGAAAAAAAA

GGYGPGAGQQGPGGAGQQGPGSQGPGGAGQQGPGGQGPYGPGAAAAAAAV

GGYGPGAGQQGPGSQGPGSGGQQGPGGQGPYGPSAAAAAAAA

GGYGPGAGQQGPGSQGPGSGGQQGPGGQGPYGPSAAAAAAAA

GGYGPGAGQQGPGSQGPGSGGQQGPGGQGPYGPSAAAAAAAA

GGYGPGAGQQGPGSGGQQGPGGQGPYGSGQQGPGGAGQQGPGGQGPYGPGAAAAAAAA

GGYGPGAGQQGPGGAGQQGPGSQGPGGAGQQGPGGQGPYGPGAAAAAAAV

GGYGPGAGQQGPGSQGPGSGGQQGPGGQGPYGPSAAAAAAAA

GGYGPGAGQQGPGSQGPGSGGQQGPGGQGPYGPSAAAAAAAA

GGYGPGAGQQGPGSQAPVASAAASRLSSPQASSRVSSAVSTLVSSGPTNPAALSNAISSVVSQVSASNPGLSGCDVLVQALLELVSALVHILGSSSIGQINYAASSQYAQMVGNSVTQALG

>Aaur_MaSp2.2e

MNWSIRLALLGLVVLSTQTVFSAGQGATPWENSQLAEEFINSFLRFIAQSGAFSPNQLDDMSSIGDTLKTAIEKMAQSRKSSKSKLQALNMAFASSMAEIAVAEQGGLSLEAKTNAIANALTSAFLETTGVVNQQFVSEIKGLIYMIAQASSNEISGSAAAAGGGSGGGSSGQGGYGQGAYASVSTATTYGSAPQGAGGPAPQGPSQQGPISQPSYGASATVTVTTVGGRQQGPTGPSQQGPGQQGPYGPSAAAASAAVS

GYGPGGGQQEQQGPGGQGPGSAGQQGPGQQGPYGSSAAATAAAA

GGYGPGAGQQGPGRAGQQGPGSQGPGGAGQQGPGGQGPYGPGADAAAAAV

GGYGPGAGQQGPGSQGPGSGGQQGPGGQGPYGPSAAAAAAATA

GYGPGAGQQGPGSQGPGGAGQQGPGGQGPYGPGSAAAAAAA

GGYGPGVGQQGPGSQGPGSSGQQGPGGQGPYGPSAAAAAAAA

GGYGPGAGQQGPGGAGQQGPGSQGPGGAGQQGPGGQGPYGPGAAASAAAV

GGYGPGAGQQGPGSQGPGSGGQQGPGGQGPYAPSAAAAAAAT

GGYGPGAGQQGPGSQGPGSGGQQGPGSQGPYGPSAATAAAAA

GGYGPGAGQQGPGSQGPGSGGQQGPGSQGPYGPSAAAAAAAA

GGYGPGAGQQGPGSGGQQGSGGQGPYGSGQQGPGGAGQQGPGGQGPYGPGSAAAAAAA

GGYGPGVGQQGPGSQGPGSSGQQGPGGQGPYGPSAAAAAAAA

GGYGPGAGQQGPGGAGQQGPGSQGPGGAGQQGPGGQGPYGPGAAAAAAAV

GGYGPGAGQQGPGSQGPGSGGQQGPGGQGPYGPSAAAAAAAT

GGYGPGAGQQGPGSQGPGSGGQQGPGGQGAYGSSAAAAAAAA

GGYGPGAGQQGPGSGGQQGSGGQGPYGSGQQGPGGAGQQGPGGQGPYGPGSAAAAAAA

GGYGPGVGQQGPGSQGPGSSGQQGPGGQGPYGPSAAAAAAAA

GGYGPGAGQQGPGGAGQQGPGSQGPGGAGQQGPGGQGPYGPGAAAAAAAV

GGYGPGAGQQGPGSQGPGSGGQQGPGGQGPYGPSAAAAAAAT

GGYGPGAGQQGPGSQGPGSGGQQGPGGQGPYGSSAAAAAAAA

GGYGPGAGQQGPGSGGQQGSGGQGPYGSGQQGPGGAGQQGPGGQGPYGPGSAAAAAAA

GGYGPGVGQQGPGSQGPGSSGQQGPGGQGPYGPSAAAAAAAA

GGYGPGAGQQGPGGAGQQGPRSQGPGGAGQQGPGGQGPYGPGAAAAAAAV

GGYGPGAGQQGPGSQGPGSGGQQGPGGQGPYGPSAAAAAAAT

GGYGPGAGQQGPGSQGPGSGGQQGPGGQGAYGSSAAAAAAAA

GGYGPGAGQQGPGSGGQQGSGGQGPYGSGQQGPGGAGQQGPGGQGPYGPGSAAAAAAA

GGYGPGVGQQGPGSQGPGSSGQQGPGGQGPYGPSAAAAAAAA

GGYGPGAGQQGPGGAGQQGPGSQGPGGAGQQGPGGQGPYGPGAAAAAAAV

GGYGPGAGQQGPGSQGPGSGGQQGPGGQGPYGPSAAAAAAAT

GGYGPGAGQQGPGSQGPGSGGQQGPGGQGAYGSSAAAAAAAA

GGYGPGAGQQGPGSGGQQGSGGQGPYGSGQQGPGGAGQQGPGGQGPYGPGSAAAAAAA

GGYGPGVGQQGPGSQGPGSSGQQGPGGQGPYGPSAAAAAAAA

GGYGPGAGQQGPGGAGQQGPGSQGPGGAGQQGPGGQGPYGPGAAAAAAAV

GGYGPGAGQQGPGSQGPGSGGQQGPGGQGPYGPSAAAAAAAT

GGYGPGAGQQGPGSQGPGSGGQQGPGGQGAYGSSAAAAAAAA

GGYGPGAGQQGPGSGGQQGSGGQGPYGSGQQGPGGAGQQGPGGQGPYGPGSAAAAAAA

GGYGPGVGQQGPGSQGPGSSGQQGPGGQGPYGPSAAAAAAAA

GGYGPGAGQQGPGGAGQQGPGSQGPGGAGQQGPGGQGPYGPGAAAAAAAV

GGYGPGAGQQGPGSQGPGSGGQQGPGGQGPYGPSAAAAAAAT

GGYGPGAEQQGPGSQGPGSGGQQGPGGQGAYGSSAAAAAAAA

GGYGPGAGQQGPGSGGQQGSGGQGPYGSGQQGPGGAGQQGPGGQGPYGPGSAAAAAAA

GGYGPGVGQQGPGSQGPGSSGQQGPGGQGPYGPSAAAAAAAA

GGYGPGAGQQGPGGAGQQGPGSQGPGGAGQQGPGGQGPYGPGAAAAAAAV

GGYGPGAGQQGPGSQGPGSGGQQGPGGQGPYGPSAAAAAAAT

GGYGPGAEQQGPGSQGPGSGGQQGPGGQGAYGSSAAAAAAAA

GGYGPGAGQQGPGGQGPYGSGQQGPGGAGQQGPGGQGPYGPGSAAAAAAA

GGYGPGDGQQGPGGAGQQGPGSQGPGGAGQQRPGGQGPYGPGAAAAAAAV

GGYGPGAGQQGPGSQGPGSGGQLGPGSQGLYGPSSAAAAAAV

GGYGPGAGQQGPGSQGPGSGGQQGPYGPSSSTAAASAGGYGPGTVQQGPRSQAPVASAAASRLSSPQASSRVSSAVSTLVSSGPTNPAALSNAISSVVSQVSASNPGLSGCDVLVQALLEIVSALVHILGSSSIGQINYAASSQYTQMVGNSVAQALG

>Atri_MaSp2.2a

MNWSIRLALLGFVVLSTQTVFSAGQGATPWENSQLAEQFINSFLRFIGQSGAFSPNQLDDMSSIGDTLKTAIEKMAQSRKSSKSKLQALNMAFASSMAEIAVAEQGGLSLEAKTNAIENALISAFLETTGVVNQQFVSEIKSLIYMIAQASSNEISGSAAAAGGGSGGGGSGQGGYGQGSYASASAAAAYGSAPQGTGGPAPQGPSQQGPVSQPSYGPSAAVAVTAVGGRQQGPSAPSQQGPSQQGPGQQGSGGQGPYGPSAAAAAAAA

GGYGPGAGQQGQQGGQGPSGSGQQGPGSAGQRGPGGQGPYGPGAAAAAAAAA

GGYGPGAGQQGPGSQGPGSGGQQGPGSQGPYGPSAAAAAAAA

GPGYGPGAGQQGPGSQGPGSGGQQGPGGQGPYGPGAAAAAAAA

GGYGPGAGQQGPGSGGQQGGQGSGQQGPGGAGQGGPGGQGPYGPGAAAAAAA

GGYGPGAGQQGPGSQGPGSGGQQGPGAQGPYGPGAAAAAAAA

GPGYGPGAGQQGPGSQGPGSGGQQGPGGQGPYGPSAAAAAAAA

GGYGPGAGRQGPGSGGQQGGQGSGQQGPGGAGQGGPGGQGPYGPGAAAAAAAAA

GGYGPGAGQQGPGSQGPGSGGQQGPGAQGPYGPSAAAAAAAA

GPGYGPGAGQQGPGSQGPGSGGQQGPGGQGPYGPSAAAAAAAA

GGYGPGAGRQGPGSGGQQGGQGSGQQGPGGAGQGGPGGQGPYGPGAAAAAAAAA

GGYGPGAGQQGPGSQGPGSGGQQGPGAQGPYGPSAAAAAAAA

GPGYGPGAGQQGPGSQGPGSGGQQGPGGQGPYGPSAAAAAAAA

GGYGPGAGRQGPGSGGQQGGQGSGQQGPGGAGQGGPGGQGPYGPGAAAAAAAAA

GGYGPGAGQQGPGSQGPGSGGQQGPGAQGPYGPSAAAAAAAA

GPGYGPGAGQQGPGSQGPGSGGQQGPGGQGPYGPSAAAAAAAA

GGYGPGAGRQGPGSGGQQGGQGSGQQGPGGAGQGGPGGQGPYGPGAAAAAAAAA

GGYGPGAGQQGPGSQGPGSGGQQGPGAQGPYGPSAAAAAAAA

GPGYGPGAGQQGPGSQGPGSGGQQGPGGQGPYGPSAAAAAAAA

GGYGPGAGRQGPGSGGQQGGQGSGQQGPGGAGQGGPGGQGPYGPGAAAAAAAAA

GGYGPGAGQQGPGSQGPGSGGQQGPGAQGPYGPSAAAAAAAA

GPGYGPGAGQQGPGSQGPGSGGQQGPGGQGPYGPSAAAAAAAA

GGYGPGAGRQGPGSGGQQGGQGSGQQGPGGAGQGGPGGQGPYGPGAAAAAAAAA

GGYGPGAGQQGPGSQGPGSGGQQGPGAQGPYGPSAAAAAAAA

GPGYGPGAGQQGPGSQGPGSGGQQGPGGQGPYGPSAAAAAAAA

GGYGPGAGRQGPGSGGQQGGQGSGQQGPGGAGQGGPGGQGPYGPGAAAAAAAAA

GGYGPGAGQQGPGSQGPGSGGQQGPGAQGPYGPSAAAAAAAA

GPGYGPGAGQQGPGSQGPGSGGQQGPGGQGPYGPSAAAAAAAA

GGYGPGAGRQGPGSGGQQGGQGSGQQGPGGAGQGGPGGQGPYGPGAAAAAAAAA

GGYGPGAGQQGPGSQGPGSGGQQGPGAQGPYGPSAAAAAAAA

GPGYGPGAGQQGPGSQGPGSGGQQGPGGQGPYGPSAAAAAAAA

GGYGPGAGRQGPGSGGQQGGQGSGQQGPGGAGQGGPGGQGPYGPGAAAAAAAAA

GGYGPGAGQQGPGSQGPGSGGQQGPGAQGPYGPSAAAAAAAA

GPGYGPGAGQQGPGSQGPGSGGQQGPGGQGPYGPSAAAAAAAA

GGYGPGAGRQGPGSGGQQGGQGSGQQGPGGAGQGGPGGQGPYGPGAAAAAAAAA

GGYGPGAGQQGPGSQGPGSGGQQGPGAQGPYGPSAAAAAAAA

GPGYGPGAGQQGPGSQGPGSGGQQGPGGQGPYGPSAAAAAAAA

GGYGPGAGRQGPGSGGQQGGQGSGQQGPGGAGQGGPGGQGPYGPGAAAAAAAAA

GGYGPGAGQQGPGSQGPGSGGQQGPGAQGPYGPSAAAAAAAA

GPGYGPGAGQQGPGSQGPGSGGQQGPGGQGPYGPSAAAAAAAA

GGYGPGAGRQGPGSGGQQGGQGSGQQGPGGAGQGGPGGQGPYGPGAAAAAAAAA

GGYGPGAGQQGPGSQGPGSGGQQGPGAQGPYGPSAAAAAAAA

GPGYGPGAGQQGPGSQGPGSGGQQGPGGQGPYGPSAAAAAAAA

GGYGPGAGRQGPGSGGQQGGQGSGQQGPGGAGQGGPGGQGPYGPGAAAAAAAAA

GGYGPGAGQQGPGSQGPGSGGQQGPGAQGPYGPSAAAAAAAA

GPGYGPGAGQQGPGSQGPGSGGQQGPGGQGPYGPSAAAAAAAA

GGYGPGAGRQGPGSGGQQGGQGSGQQGPGGAGQGGPGGQGPYGPGAAAAAAAAA

GGYGPGAGQQGPGSQGPGSGGQQGPGAQGPYGPSAAAAAAAA

GPGYGPGAGQQGPGSQGPGSGGQQGPGGQGPYGPSAAAAAAAA

GGYGPGAGRQGPGSGGQQGGQGSGQQGPGGAGQGGPGGQGPYGPGAAAAAAA

GGYGPGAGQQGPGSQGPGSGGQQGPGGQGPYGPSAAAAAAAA

GGYGPGAGRQGPGSGGQQGGQGSGQQGPGGAGQGGPGGQGPYGPGAAAAAAAAA

GGYGPGAGQQGPGSQGPGSGGQQGPGAQGPYGPSAAAAAAAA

GPGYGPGAGQQGPGSQGPGSGGQQGPGGQGPYGPSAAAAAAAA

GGYGPGAGRQGPGSGGQQGGQGSGQQGPGGAGQGGPGGQGPYGPGAAAAAAAAA

GGYGPGAGQQGPGSQGPGSGGQQGPGAQGPYGPSAAAAAAAA

GPGYGPGAGQQGPGSQGPGSGGQQGPGGQGPYGPSAAAAAAAA

GGYGPGAGRQGPGSGGQQGGQGSGQQGPGGAGQGGPGGQGPYGPGAAAAAAAAA

GGYGPGAGQQGPGSQGPGSGGQQGPGAQGPYGPSAAAAAAAA

GPGYGPGAGQQGPGSQGPGSGGQQGPGGQGPYGPSAAAAAAAA

GGYGPGAGRQGPGSGGQQGGQGSGQQGPGGAGQGGPGGQGPYGPGAAAAAAAA

GGYGPGAGQQGPGSQGPGSGGQQGPGAQGPYGPSAAAAAAAA

GPGYGPGAGQQGPGSQGPGSGGQQGPGGQGPYGPSAAAAAAAA

GGYGPGAGRQGPGSGGQQGGQGSGQQGPGGAGQGGPGGQGPYGPGAAAAAAAAA

GGYGPGAGQQGPGSQGPGSGGQQGPGAQGPYGPSAAAAAAAA

GPGYGPGAGQQGPGSQGPGSGGQQGPGGQGPYGPSAAAAAAAA

GGYGPGAGRQGPGSGGQQGGQGSGQQGPGGAGQGGPGGQGPYGPGAAAAAAAA

GGYGPGAGQQGPGSQGPGSGGQQGPGAQGPYGPSAAAAAAAA

GPGYGPGAGQQGPGSQGPGSGGQQGPGGQGPYGPSAAAAAAAA

GGYGPGAGRQGPGSGGQQGGQGSGQQGPGGAGQGGPGGQGPYGPGAAAAAAAAA

GGYGPGAGQQGPGSQGPGSGGQQGPGSQGPYGPSAAAAAAAA

GPGYGPGAGQQGPGSQGPGSGGQQGPGSQGPYGPSAAAAAAAA

GPGYGPGAGRQGPGSQAPVASAAASRLSSPQASSRVSSAVSTLVSSGPTNPASLSNAISSVVSQVSASNPGLSGCDVLVQALLEIVSALVHILGSSSIGQINYAASSQYAQMVGQSLTQALG

>Atri_MaSp2.2b

MNWSIRLALLGFVVLSTQTVFSAGQGATPWENSQLAESFISSFLRFIGQSGAFSPNQLDDMSSIGDTLKTAIEKMAQSRKSSKSKLQALNMAFASSMAEIAVAEQGGLSLEAKTNAIASALSAAFLETTGYVNQQFVNEIKTLIFMIAQASSNEISGSAAAAGGSSGGGGGSGQGGYGQGAYASASAAAAYGSAPQGTGGPASQGPSQQGPVSQPSYGPSATVAVTAVGGRPQGPSAPRQQGPSQQGPGQQGPGGRGSYGPSAAAAAAAA

GGYGPGAGQQGQQGQGSGQQGPGGAGQGGPRGQGPYGPGAATAAAAAA

GPGYGPGAGQQGPGSQGPGSSGQQGPGSQGPYGLSAAAAAAAA

GPGYGPGAGQQGPGSQGPGSGGQQGPGGRGPYGPSAAAAAAAA

GPGYGPGAGQQGPGSGGQQGGQGSGQQGPGGAGQGGPRGQGPYGPGAAAAAAAAA

GGYGPGAGQQGPGSQGPGSGGQQGPGSQGPYGPSAAAAAAAA

GPGYGPGAGQQGPGSXGPGSGGQQGPGGQGPYGPSAAAAAAAA

GPGYGPGAGQQGPGSGGQQGGQGSGQQGPGGAGQGGPRGQGPYGPGAAAAAAAAA

GGYGPGAGQQGPGSQGPGSGGQQGPGSXGPYGPSAAAAAAAA

GPGYGPGAGQQGPGSQGPGSGGQQGPGGQGPYGPSAAAAAAAA

GPGYGPGAGQQGPGSGGQQGGQGSGQQGPGGAGQGGPRGQGPYGPGAAAAAAAA

GGYGPGAGQQGPGSQGPGSGGQQGPGSQGPYGPSAAAAAAAA

GPGYGPGAGQQGPGSQGPGSGGQQGPGGQGPYGPSAAAAAAAA

GPGYGPGAGQQGPGSGGQQGGQGSGQQGPGGAGQGGPRGQGPYXPGAAAAAAAAA

GGYGPXAGQQGPGSQGPGSGGQQGPGSQRPYGPSAAAAAAAA

GPGYGPGAGQQGPGSQGPGSGGQQGPGGQGPYGPSAAAAAAAA

GPGYGPGAGQQGPGSGGQQGGQGSGQQGPGGAGQGGPRGQGPYGPGAAAXAAAAA

GGYGPGAGQQGPGSQGPGSGGQQGPGSQGPYGPSAAAAAAAA

GPGYGPGAGQQGPGSXGPGSGGQQGPGGQGPYGPSAAAAAAAA

GPGYGPGAGQQGPGSGGQQGGQGSGQQGPGGXGXGGPRGQGPYXPGAAAAAAAA

GXYGPGAGQQGPGSQGPGSGGQQGPGSXGPYGPXAAAAAAAA

XPGYGPGAGQXGPGSQGPGSGGQQGPGGQGPYGPSAAAAVAAA

GPGYGPGAGQRPGSGGQQGGQGSGQQGRGGIGRGPRGQGPHGPGAAAAAAAAA

GGYGPGAGQQGPGSQGPGSGGQQGPGSQGPXGPSAAAAAAA

XPGYXPGAXQQGPGSQGPGSGGQQGPGGQGPYGPSAAAAAAAA

GPGYGPGAXQQGPGSGGQQGGQGSGQQGPGGACQGGPRGQGPYGPGAAAAAAAA

GGYGPGAGQQGPGSQGPGSGGQQGPGSQGPYGPSAAAAAAAA

GPGYGPGAGQQGPGSQGPGSGGQQGPGGQGPYGPSAAAAAAAA

GPGYGPGAGQQGPGSGGQQGGQGSGQQGPGGAGQGGPRGQGPYGPGAAAAAAAAA

GGYGPGAGQQGPGSQGPGSGGQQGPGSQGPYGPSAAAAAAAA

GPGYGPGAGQQGPGSQGPGSGGQQGPGGQGPYGPSAAAAAAAA

GPGYGPGAGQQGPGSGGQQGGQGSGQQGPGGAGQGGPRGQGPYGPGAAPAAAAAA

GGYGPGAGQQGPGSQGPGSGGQQGPGSQGPYGPSAAAAAAAA

GPGYGPGAGQQGPGSQGPGSGGQQGPGGQGPYGPSAAAAAAAA

GPGYGPGAGQQGPGSGGQQGGQGSGQQGPGGAGQGGPRGQGPYGPGAAAAAAAA

GGYGPGAGQQGPGSQGPGSGGQQGPGSQGPYGPSAAAAAAAA

GPGYGPGAGQQGPGSQGPGSGGQQGPGGQGPYGPSAAAAAAAA

GPGYGPGAGQQGPGSGGQQGGQGSGQQGPGGAGQGGPRGQGPYGPGAAAAAAAA

GGYGPGAGQQGPGSQGPGSGGQQGPGSQGPYGPSAAAAAAAA

GPGYGPGAGQQGPGSQGPGSGGQQGPGGQGPYGPSAAAAAAAA

GPGYGPGAGQQGPGSGGQQGGQGSGQQGPGGAGQGGPRGQGPYGPGAAAAAAAA

GGYGPGAGQQGPGSQGPGSGGQQGPGSQGPYGPSAAAAAAAA

GPGYGPGAGQQGPGSQGPGSGGQQGPGGQGPYGPSAAAAAAAA

GPGYGPGAGQQGPGSGGQQGGQGSGQQGPGGAGQGGPRGQGPYGPGAAAAAAAA

GGYGPGAGQQGPGSQGPGSGGQQGPGSQGPYGPSAAAAAAAA

GPGYGPGAGQQGPGSQGPGSGGQQGPGGQGPYGPSAAAAAAAA

GPGYGPGAGQQGPGSGGQQGGQGSGQQGPGGAGQGGPRGQGPYGPGAAAAAAAA

GGYGPGAGQQGPGSQGPGSGGQQGPGSQGPYGPSAAAAAAAA

GPGYGPGAGQQGPGSQGPGSGGQQGPGGQGPYGPSAAAAAAAA

GPGYGPGAGQQGPGSGGQQGGQGSGQQGPGGAGQGGPRGQGPYGPGAAAAAAAA

GGYGPGAGQQGPGSQGPGSGGQQGPGSQGPYGPSAAAAAAAA

GPGYGPGAGQQGPGSQGPGSGGQQGPGGQGPYGPSAAAAAAAA

GPGYGPGAGQQGPGSGGQQGGQGSGQQGPGGAGQGGPRGQGPYGPGAAAAAAAA

GGYGPGAGQQGPGSQGPGSGGQQGPGSQGPYGPSAAAAAAAA

GPGYGPGAGQQGPGSQGPGSGGQQGPGGQGPYGPSAAAAAAAA

GPGYGPGAGQQGPGSQAPVASAAASRLSSPQASSRVSSAVSTLVSSGPTNPASLSNAISSVVSQVSSSNPGLSGCDVLVQALLEIVSALVHILGSSSIGQINYAASSQYAQLVGQSLTQALG

>Atri_MaSp2.2c

MNWSIRLALLGFVVFSTQTVFSAGQSATPWENSQLAEQFINSFLRFIGQSGAFSPNQLDDMSSIGDTLKTAIEKMAQSRKSSKSKLQALNMAFASSMAEIAVAEQGGLSLEAKTNAIENALISAFLETTGVVNQQFVSEIKSLIYMIAQASSNEISGSTAAAGGGSGGGGSGGRGGYGQGSYASASAAAAYGSAPQGTGGPAPQGPSQQGPVSQPSYGPSASVTVAVVGGRQQGPAGPSQQGPGQQGPGQQAPGGQGPYGPSAAAAAAAS

GGYGPGAGQQGGQGPGSQGPGSGGQQGPGIQGPYGPSAAAAAAA

GPGYGPGAGQQGPGSQRPGAGQQGPGSQGQGGAGQQGPGGQGPYGPGAAAAAAAV

GGYGPGAGQQGPGSGGQQGPGSQGPYGPSAAAAAAA

GPGYGPGAGQQGPGSQGPGAGQQGPGSQGPGGAGQQGPGGQGPYGPGAAAAAAAV

RGYGPGAGQQGPGSQGPGGGGQQGPGSQGPYGPSAAAAAAA

GPGYGPGAGQQGPGSQGPGSGGQQGPGSLGPYGPSAAAAAAA

GPGAGQQGPGSQGQGAGQQGPGSQGPGGAGQQGPGRQGPYGPGAAAAAAAV

GGYGPGAGQQGPGSQGPGSGGPQGSGSQGPYGPSAAAAA

GPGYGPGAGQQGPGSQGPGSGGQQGPGSQGPYGPSAAAAAAA

GPGYGPGAGQQGPGSQGPGAGQQGPGSQGPGGAGQQGPGRQGPYGPGAAAAAAAV

GGYGPGAGQQGPGSQGRGSGGPQGPGSQGPYGPSAAAAAAA

GPGYGPGAGQQGPGSQGPGSGGQQGPGSQGPYGPSAAAAAAA

GPGYGPGAGQQGPGSQGPGAGQQGPGSQGPGGASQQGPGGQGPYGPGAAAAAAAV

GGYGPGAGQQGPGSQGPGSGGQQGPGSQGPYGPSAAAAAAA

GPGYGPGAGQQGPGSQGQGAGQQGPGSQGPGGAGQQGPGRQGPYGPGAAAAAAAV

GGYGPGAGQQGPGSQGQGSGGPQGPGSQGPYGPSAAAAAAA

GPGYGPGAGQQGPGSQGPGSGGQQGPGSQGPYGPSAAAAAAA

GPGYGPGAGQQGPGSQGPGAGQQGPGSQGPGGAGQQGPGRQGPYGPGAASAAAAV

GGYGPGSGQQGPGSQGPGSGGPQGPGSQGPYGPSAAAAAAA

GPGYGPGAGQQGPGSQGPGSGGQGPGSQGPYGPSAAAAAAA

GPGYGPGAGQQGPGSQGPGAGQQGPGSQGPGGAGQQGPGGQGPYGPGAAAAAAAV

GGYGPGAGSQGPGSGGPQGPGSQGPYGPSAAAAAAA

GPGYGPGAGQQGPGSQGPGSGGQQGPGSQGPYGPSAAAAAAA

GPGYGPGAGQQGPGSQGPGAGQQGPGSQGPGGAGQQGPGRQGPYGPGAAAAAAAV

GGYGPGAGQQGPGSQGQGSGGPQGPGSQGPYGPSAAAAAAA

GPGYGPGAGQQGPGSQGPGSGGQQGPGSQGPYGPSAAAAAAA

GPGYGPGAGQQGPGSQGPGAGQQGPGSQGPGGAGQQGPGRQGPYGPGAAAAAAAV

GGYGPGSGQQGPGSQGPGSGGPQGPGSQGPYGPSAAAAAAA

GPGYGPGAGQQGPGSQGPGSGGQQGPGSQGPYGPSAAAAAAA

GPGYGPGAGQQGPGAGQQGPGSQGPGGAGQQGPGRQGPYGPGAAAAAAAV

GGYGPGAGQQGPGSQGPGSGGPQGPGSQGPYGPSAAAAAAV

GPGYGPGAGQQGPGSQGPGSGGQQGPGSQGPYGPSAAAAAAA

GPGYGPGAGQQGPGSQGQGAGQQGPGSQGPGGAGQQGPGRQGPYGPGAAAAAAAV

GGYGPGSGQQGPGSQGPGSGGPQGPGSQGPYGPSAAAAAAA

GPGYGPGAGQQGPGSQGPGSGGQQGPGSQGPYGPSAAAAAAA

GPGYGPGAGQQGPGSQGPGAGQQGPGSQGPGGAGQQGPGRQGPYGPGAAAAAAAV

GGYGPGAGQQGPGSQGPGSGGPQSPGSQGPYGPSAAAAAAA

GPGYGPGAGQQGPGSQGPGSGGQQGPGSQGPYGPSAAAAAAA

GPGYGPGAGQQGPGSQGPGAGQQGLGSQGPGGAGQQGPGRQGPYGPGAAAAAAAV

GGYGPGAGQQGPGSQGPGSGGPQGPGSQGPYGPSAAAAAAV

GPGYGPGAGQQGPGSQGPGSGGQQGPGSQGPYGPSAAAAAAA

GPGYGPGAGQQGPGSQGQGAGQQGPGSQGPGGAGQQGPGRQGPYGPGAAAVAAAV

GGYGPGSGQQGPGSQGPGSGGPQGPGSQGPYGPSAAAAAAA

GPGYGPGAGQQGPGSQGPGSGGQQGPGSQGPYGPSAAAAAAA

GPGYGPGAGQQGPGSQGPGSGQQGPGSQGPGGAGQQGPGRQGPYGPGAAAAAAAV

GGYGPGAGQQGPGSQGPGSGGPQGPGSQGPYGPSAAAAAAA

GPGYVPGAGQQGPGSQGPGSGGQQGPGSQGPYGPSAAAAAAA

GPGYGPGAGQQGPGSQGPGAGQQGPGSQGPGGASQQGPGGQGPYGPGAAAAAAAV

GGYGPGAGQQGPGSQGPGSGGQQGPGSRGPYGPSASAAAAA

GPGYGPGAGQQGPGSQGPGSGGQQGPGSQGPYGPSAAAAAAA

GPGYGPGAGQQGPGSQGPGAGQQGPGSQGPGGAGQQGPGRQGPSGPGAAAAAAAV

GGYGPGAGQQGPGSQGPGSGGQQGPGSQGPYGPSAAAAAAA

GPGYGPGAGQQGPGSQGPGSGGQQGPGSQGPYGPSAAAAAAA

GPGYGPGSGQQGPGSQGPGAGQQGPGSQGPGGAGQQGPGRQGPYGPGAAAAAAAV

GGYGPGAGQQGPGSQGPGSGGQQGPGSQGPYGPSAAAAAAA

GPGYGPGAGQQGPGSQGPGSGGQQGPGSQGPYGPSAAAAAAA

GPGYGPGAGQQGPGSQGPGAGQQGPGSQGPGGSGQQGPGRQGPYGPGAAAAAAAV

GGYGPGAGQQGPGSQGPGSGGQQGPGSQGPYGPSAAAAAAA

GPGYGPGAGQQGPGSQGPGAGQQGPGSQGPGGAGQQGPGRQGPYGPGAAAAAAAV

GGYGPGAGQQGPGSQGPGSGGQQGPGSQGPYGPSAAAAAAA

GPGYGPGAGQQGPGSQGPGSGGQQGPGSQGAYGPSAAAAAAA

GPGYGPGAGQQGPGSQGPGSGQQGPGSQGPGGAGQQGPGRQGPYGPGGAAAAAAVA

GYGPGAGQQGPGSQGPGSGGQQGPGSQGPYGPSAAAAAAA

GPGYGPGAGQQGPGSQGPGSGGQQGPGSQGPYGPSAAAAAAA

GPGYGPGAGQQGPGSQGPGAGQQGPGSQGPGGAGQQGPGRQGPYGPGGAAAAAAV

GGYGPGAGQQGPGSQGPGSGGQQGPGSQGPYGPRAAAAAAA

GPGYGPGAGQQGPGSQGPGSGGQQGPGSQGPYGPSAAAAAAA

GPGYGPGAGQQGPGSQGLGAGQQGPGSQGPGGAGQQGPGRQGPYGPGAAAAAAAV

GGYGPGAGQQGPGSQGPGSGGPQGPGSQGPYGPSAAAAAAA

GPGYGPGAGQQGPGSQGPGSGGQQGPGSQGPYGPSAAAAAAA

GPGYGPGAGQQGPGSQGPGAGQQGPGSQGPGGAGQQGPGRQGPYGPGGAAAAAV

GGYGPGAGQQGPGSQGPGSGGQQGPGSQGPYGPSAAAAAAA

GPGYGPGAGQQGPGSQGPGSGGQQGPGSQGPYGPSAAAAAAA

GPGYGPGTGQQGPGSQGPGAGQQGPGSQGPGGAGQQGPGRQGPYGPGGAAAAAAV

GGYGPGAGQQGPGSQGPGSGGQQGPGSQGPYGPSAAAAAAA

GPGYGPGAGQQGPGSQGPGSGGQQGPGSQGPYGPSAAAAAAI

GPGYGPGAAQQGPGSQAPVASAAASRLSSPQAGSRVSSAVSSLVSNGPTNPASLANAISSVVSQVSASNPGLSGCDVLVQALLEIVSALVHILGSSSIGQINYAASSQYAQMVGNSVAQALG

>Aur_MaSp3a

MAWIARLPLLVLVALCTQTMIVHGQDSHPWKDTRTTESFMENFVEYFRQSGYFNSDDIESIKDLADTLIQSLNEMQAKGKNSHQVLQALNMGFAAGVAELVNSDGINLKEKQNAIREAMKKSQLQTTGVINESFMNEMDKLMQMFSQINALNDDSVGYGAGAVSYASSASASNAQGIGQNFGYQGQGSSSSSVSSISVGGLPQGPVGSDSYEYSLSVNSLSGLPNAYGGQYDSGVGMEQGSLGTGSSGGAAVATASGGASGNGYGPGYGGIGGFGLGGTSAAVAVGRAGESGYGRGGNRLGGVRAAASS

GVGPGGPGYGGDGYSGTGRSGPGVAAAAAAS

GGRGGGDRYGPLGAGGYGQGSGSGTGGAAAAAASS

GEGPGGAGYSGDGYGGPGGSGPGSAAAAAAS

GGRGGDGRYGQQGAGGYGQGGNGLGGAGAAATS

GEGPGGAGYGGPGGSGPGSAAAAAAS

GGRGGGGRYGQQDADGYGQGGSGLGGVGAAANAASS

DEGPGGAGYGGEGGSGSGSAAAAAAS

GGQGGGGRYGPQGVGGYGQGGSGLGGAGAAASS

GEGPGGAGYGGDGYGGPGGSGPGSAAAAAVS

GGRGGSGRYGQQGAGGYGQGNGSEAAGAAAAAASS

GGGPGGAGYGGQGGTGPGSAASAATS

GGRGGGGRYGPQGAGGYGQGGNGLGGAGAAASS

GEGPGGAGYGGPGGSGPGSAAAAAVS

GGRGGAGRYGQQGADGYGQGGSGLGGAGAAAAAASS

GEGPGDAGYGDDGYGGPGGSGPGSAAAAAAS

GGRGGDGRYGQQGAGGYGQGGNGLGGAGAAASS

GEGPGGAGYGGPGGSGPGSAAAAAAS

GGRGGGGRYGLQGAGGYGQGGSEIGGVGAAASAASS

GEGPGGAGYGGDGYGGPGGSGPGSASAAAAS

GGRGGFGGYGPQGARGYGQGGSGLGGVGAAASAASS

VEGPAGAGYVGDGYGGPGWSGPGTAAAAAAS

GGRGGGRRYGQQGAGGYGQGNGSEGAGAAAAAASSS

EGPGGAGYGGDGYVGQGGSGPGGAASAASAL

GGQGVGGGYGQQGAGGYGEGGSGSGGSGAAAAAASS

GEGQGGARYSGDGYAVPGGSGQDGTASAAASAS

GIRGPGGLRGSkeiikkiivhrrvgsasdaeasvieenGYGGEGGYGAGYDGQGGSAPGGAAAAAAS

GGQGGGSGYGPLGAGGYGQGGSGLGGAGAAAAAALS

GEGPGGAGYGGPGLSGPGSAAAAAAS

GGRGGGGRYGQQGAGGYGQRGSGLGGEGSAAAAASS

GEGSGGAGYGGDGYGGPGGSGLGAAAAAAAS

GGRGGGGRYGQQGAGGYGQGNGSEGAGASAAAASS

GEGPGGAGYGGQGGSGPGSAASAAAL

GGRGGGGRYGPQGAGGYGQGGSGLGGAGEGAAAAAASS

GEGPGGIDNGGDGYGGPGGSGPGSAAAAAAS

GGQVGGSRYGPQGAGGYGQRSGSVRARAAAAAASS

FEISGGAGYDGQVVSGPGSAAAAAAS

GGRGSGGRYGPQGADGYGQGSGSEGAGAAAAAASSS

EGPGGAGYRGQGRSGSGSAAAAAAS

GGQGGGGRYGPQGAGGYGQRGSGLGGAGAAAAAVSS

GQGPGGADYGRDAYGGPEGSGPGSAAAAAAS

GGRGGGGRYGPQGAGGYGPGGAGVAAAAASS

GEGPGGAGYGGDGYGGPGGNGPDNAAAAAAS

GGRGGGGRYGPQGAGGYGQGGSGLGEGAGEGGAGAAASS

GEGPGGAGYDDDGYGGPGGSGPGSAAAAAAS

GGRGGGGRYGQQGAGGYGQGGSGLGGVGAATSAASS

GEGPGGGGYGGEGGSVPGSAAAAAAS

GGQGGGGRYGPQGAGGYGEGGSGLGGAGAAAAAASS

VEGPGGAGYDDDGYGAPGGSGPGSAAAAAAS

GGRGGGGRYGQQGAGGYGQGGSGLGGVGAATSAASS

GEGPGGGGYGGEGGSGPGSAAAAAAS

GGREGGGRYGPQGAGGYGQGGSGLGGAGAAASS

VEFPGGAGYGDDGYGGPGGSEGNGVASAGTSSNGGPVELGSGRRGGSGLGGALSSAASTGGFSGPGGLRGPkeiikkiivhrrlgsasdasasvieenLYGPEAIGYYGQGGRGAGGAGAASAAVSSSE

GPGGVGYGGQLGSGSGSAAAAAPL

GGRGGGGRYGAEGAGGFGKGGGGFGGAAAAAS

GLGPGENGFDAAGYGGDGEAGPEGAAAAAAAS

GGGGSYGPQGAGGYGEGGSGSIGAGAAASAASSS

GPGGASYGGQGESGPGGAAGAAAAAA

GGRGGRGRYGAYGAGGYGEGGSGSGGTGGFGSGSDVYGGQGGSGEGAAAAAAVESS

GQGGRGRLGSNGALGARAAGAAASS

GVGSGGAGYGGDGYDGQGGSEGNGAAAAAASS

DEGPGGFRPAVRGGSGQGGAVSSAASA

GGRAGRDRLGSQGAGGAGGFDYGRDGYGGQGGSLEGGAAAAAAA

GSAGYGPQGAGDYGQGGSGPGGNGAAAASASSTAASVASRLSSPATLSRVSSAVSLFLDDDLDYPVAFSNAFDNVVSGITLSYSNISGCELLVQSLMEVLSAVLGTAYGLNANSSVDIVRSVVNRFDY

>Aur_MaSp3b

MAWIARLPLLVLVALCTQTMIVHGQDSHPWKDTRTTELFFENFVECIRQSSYFNSEDIESIKVLAETLIQSLNGMQAKGKTSHQMLQALNMGYAAGVAELVNSDGSNLQEKRNAIREAMKKSLLQATGVVNESFMNEMDKLMQMFSQINGLNDDSGGYGAGAVSYASSASASNAQGIGQNFGYQGQGSSSSSVSSISVGGLPQGPVGSGTYEYSLSVNSLSGSPSGYGGQYVRGVGVGQGGFGASGAGGAAAATTSGGASGNGYGFGYGGIGGTGLGGASAAVAVGIAGQGGYGQEGKGLERTGAAASSSVGPEGAIYGGEGYDGQGGSGPSGTAAAATASDGQGGGGRYGPQGAGGYGEGGSGSGGAAAAAASS

GVGPGGAGYGGDGYGGQGGSGPGGAAAAA

GRGGDGRYGQQGFGGFGQRGSETGGAGAAAAAASS

GEGPGGTGYGGIEGSGPGGAASAAAAS

GGRGAGGRYGPEGSGGYGQGGRGSGGAGAAASS

GVGPGGAGYGGDGYGGQGGSGPGSAASAAAAS

DGEGGRGRYGQQGSGGYGQRGSGSGEASASAAAASS

GGGPGGAGYGGDGYGGQGGSGPGGAAASAAS

GGRGGGRYGQQGFGGFGQGGSETLGAGAAAAAASS

GEGSGGAGYGGIGRSGPGGAASAAA

GGRGAGGRYGPEGSGGYGQGGRGSGLAGAAASS

GVGQGGAGYGGDDYGGQGGSGPVSAASAAAAS

DEQGGDGRYGQQGSGGYGQGGSGSGGASASAAAASS

GEGPGGAGYGGDGYGGQGGSGPGGAAASAAS

GGRGGGRYGQQGFGGFGQGGSETGGAGAAAAAASS

GEGPGGAGYGGIGGSGPGGAASAAAAS

GGRGAGGRYGPEGSGGYGQGGRGSGGASAAASS

GVGPGGAGYGGDGYGGQGGSGPGSAASAAAAS

DGQGGRGRYGQQGSGGYGQGGSGSGGASASAAAASS

GEGPGGAGYGGDGYGGQGGSGPVGAAASAAS

GGRGGGRYGQQGFGGFGQGGSETGGAGAAAAAASS

GEGPGGAGYGGIGGSGPGGAASAAAAS

GGRGAGGRYGPEGSGGYGQGGRGSGGAGAAASS

GVGPGGAGYGGDGYGGPGGSGPGSAASAAAAS

DGQGGRGRYGQQGSGGYGQGGSGSGGASASAAAASS

GEGPGGAGYGGDGYGGQGGSGPGGAAASAAS

GGRGGGRYGQQGFGGFGQGGSETGGAGAAAAAASS

GEGPGGAGYGGIEGSGPGGAASAAAAS

GGRGAGGRYGPEGSGGYGQGGRGSGGAGAAASS

GVGPGGAGYGGDGYGGQGGSGPGSAASAAAAS

DGQGGRGRYGQQGSGGYGQGGSGSGGASASAAAASS

GEGPGGAGYGGDGYGGQGGSGPGGAAASAAS

GGRGGGRYGQQGFGGFGQGGSETGGAGAAAAAASS

GEGPGGAGYGGIEGSGPGGAASAAAASS

GRGARGRYGPEGSGGYGQGGRGSGGAGAAASS

GVGPGGAGYGGDGYGGPGGSGPGSAASAAAAS

DGQGGRGRYGQQGLGGYGQGGSGSGGASASAAAS

LSGEGPGGTGYGGDGYDGQVGSGPAGTAASAAS

GGRGGGRYGQQGFGGFGQGGSETGGAGAAAAAASS

GEGPVGAGYGGIEGSGPGGAASAAAAS

GGRGARGRYGPEGSGGYGQGGRGSGGASAAASS

GVGPGGAGYGGDGYGGQGGSGPGSAASAAAAS

DGQGGRGRYGQQGSGGYGQGGSGSGGASASAAAASS

GEGPGGTGYGGDGYGGQGGSGPGGAAAS

GGRGGGRYGLQGFGGFGQGGSETGGAGAAAAAASS

GEGPGGAGYGGIEGSGPGGAASAAAASS

RRGARGRYGPEGSGGYGQGGRGSGGASAAASS

GVGPGGAGYGGDGYGGQGGSGPGSAASAAAAS

DGQGGRGRYGQQGSGGYGQGGSGSGGASASAAAASS

GEGPGGAGYGGDGYVGQGGSGPGGAAASAAS

GGRGGGRYGQHGFGGFGQGGSETGGAGAAAAAASS

GEGPGGAGYGGIGGSGPGGAASAAAAS

GGRGAGGRYGPEGSGGYGQGGRGSGGASAAASS

GVGPGGAGYGGDGYGGQGGSGPGSAASAAAAS

DGQGGRGRYGQQGSGGYGQLGSGSGGASASAAAA

TSGEGPGGTGYGGDGYGGQGGSGPGGEASAASAS

GGQGGGGGYGQQGAGDYDQGGSGSGGSGAAAAAASS

GEGPGGARYGGDGYAVQGGSGQDGIASAAASAS

GIGGPGGLRGSkeiikkiivhrrvgsasdaeasvieenGYGGQGGYGAGYDGQGGSAPGGEAAAAS

GGRGGGGRYSSQGAGRYGEGGSGSRGAGAAASAASSS

GTGGTGYSGQGVSGPGGAASAAAAAS

GGRGGRGRYNAEGAGGYGEGRNESGGTGGFGSGSDGYGEQGESGGSGAAAAA

GSAGHGPQGAGDYGQGGSGSGGNEAAAASSTAASVASRLSSPAALSRVSSAVSVFLDDDLDYPVAFTNAFDNVVSGITLSNSDISGCELLVQSLMEVLSAVLGTAYGLNANSSVDIVRSVVNRFDY

>Aarg_MaSp3a

MAWITRLPLLVLVALCTQSIIVHGQDSHPWSDVRTTESFMKNFVECIRQSSYFNTDDIESIRDLSDTMIQSLNGMTAIGKTSHQMLQALNMGYAAGVAELVNSDGFYVQEKRNAIREAMRNSLLQTTGVVNESFINEMDSLMQMFSQINVLNEDSGGNGASAASSASASNVPGIGQSLGPQGQGSSSVSVSSTSVGGLQQRPVGSGSYEYPLSVNSIGGSGYGPGGSGTGGSGAAAAAASS

GGGTGSPGYGGQGGYGPGGQAAAAAAS

DGQGGTGGGRYGPQGAGGYGQGGYGPGGSGAAAAAASS

GGGSGSPGYGPGGTAAAAAAS

NGQGGTGGGRYGPQGTGGYGQGGYGPGGSGAAAAAASS

DGGTGGPGYGGQGGYGPGGTATAAAAS

DGQGGTGGGRYGPQGAGGYGQGGYGPGGSGAAAAAASS

GGGTGSPGYGGQGGYGPGGTAAAAAAS

DGQGGTGGGRYGPQGAGGYGQGGSGSGGSGAAAAAASSS

GGTGSPGYGGQGGYGPGGVRYGPQGAGGYDQGGIGTGSGLGAASAAASS

GAGQGTIGYGGEGIGGYGPGGGDAAAAASS

GQGGGRGDRYGPQGYGGYGQGGRGPGESGAAASAASA

GEGTGSPGYGGQGGYGAGGAAAAAAAS

GGQGGGGRYGPQGAGGYGQGGIGTGSGPGAAAAAASS

GAGTGSPGYGGQGGYGPGGTAAAASAS

DGQGGTGGGRYGPQGAGGYGQGRSGSGGSGAAAAAASS

GAGTGSPGYEGQGGYGPGGTAAAAAAS

DGQGGTGGGRYGPQGAGGYGQGGYGPGESEAAAAAASS

GAGPGYGGRGRkeiikkiivrrkggsqydteafeiegnnYGAQGGSGSGPGSSAAAASSS

TGPGSAGYGGQGGSGPGGEAAAAAS

GGQGGRGRYGSQGAGGYGQGGPGYGSGAAAAGASS

GTGDDGYGGQGGSGPGGAAAAAASS

GGQGGRGRYGSQGAGGYGQGGPGSGSGAAAAAASS

GTGDDGYGGQGGSGPGGASAAAASS

GGQGGRGRYGPQGAGGYGQGGPGSGSGAAAATASS

GTGDDGYGGQGGSGPGGAAAAAASS

GGQGGRGRYGPQGAGGYGQGGPGSGSGAAAAAASS

GTGEGDDGYGGQGGSGPGGAAAAAS

GGQGGRGRYGPQGAGGYGQGGPGSGSGAAAAAASS

GTGDDGYGGQGGSGPGGAAAAAASS

GGQGGRGRYGSQGAGGYGQGGPGSGSGAAAAAASS

GTGDDGYGGQGGSGPGGAAAAAASS

GGQGGRGRYGSQGAGGYGQGGPGSGSGAAAAAASS

GTGDDGYGGQGGSGSGGAAAAAASS

GGQGGRGRYGSQGAGGYGQGGPGSGSGAAAAAASS

GTGDDGYGGQGGSGPGGAAAAAASS

GGQGGRGRYGSQGAGGYGQGGPGSGSGAAAAAASS

GTGDDGYGGQGGSGSGGAAAAAASS

GGQGGRGGYGSQGAGGYGQGGPGSGSGAAAAAASS

GTGDDGYGGQGGSGSGGAAAAAASS

GGQGGRGRYGPQGAGGYGQGGPGSGSGAAAAAASS

GTGDDGYGGQGGSGPGGAAAAAASS

GGQGGRGRYGPQGAGGYGQGGPGSGSGAAAAAASS

GTGDDGYGGQGGSGPGGAAAAAASS

GGQGGRGRYGPQGAGGYGQGGPGSGSGAAAAAASS

GTGDDGYGGQGGSGPGGASAAAASS

GGQGGRGRYGPQGAGGYGQGGPGSGSGAAAAAASS

GTGDDGYGGQGGSGPGGASAAAASS

GGQGGRGRYGPQGAGGYGQGGPGSGSGAAAAAASS

GTGDDGYGGQGGSGPGGAAAAASS

GGQGGRGRYGPQGAGGYGQGGPGSGSGAAAAAASS

GTGDDGYGGQGGSGPGGAAAAAASS

GGQGGRGRYGPQGAGGYGQGGPGSGSGAAAAAASS

GTGDDGYGGQGGSGPGGAAAAAASS

GGQGGRGRYGPQGAGGYGQGGPGSGSGAAAAAASS

GTGDDGYGGQGGSGPGGAAAAAASS

GGQGGRGRYGPQGAGGYGQGGPGSGSGAAAAAASS

GTGDDGYGGQGGSGPGGAAAAAASS

GGQGGRGRYGPQGAGGYGQGGPGSGSGAAAAAASS

GTGDDGYGGQGGSGPGGAAAAAASS

GGQGGRGRYGPQGAGGYGQGGPGSGSGAAAAAASS

GTGDDGYGGQGGSGSGGAAAAAASS

GGQGGRGRYGSQGAGGYGQGGPGSGSGAAAAAASS

GTGDDGYGGQGGSGSGGSAAAAASS

GGQGGRGRYGSQGAGGYGQGGPGSGSGAAAAAASS

GTGDDGYGGQGGSGPGGAAAAAASS

GGQGGRGRYGPQGAGGYGQGGPGSGSGAAAAAASS

GTGDDGYGGQGGSGPGGAAAAAASS

GGQGGRGRYG?QGAGGYGQGGPGSGSGAAAAAASS

GTGDDGYGGQGGSGPGGAAAAAASS

GGQGGRGRYGPQGAGGYGQGGPGSGSGAAAAAASS

GTGDDGYGGQGGSGPGGAAAAAASS

GGQGGRGRYGPQGAGAYGQGGPGSGAGAAAAAASS

GTGDDGYGGQGGSGPGGASAAAASS

GGQGGRGRYGPQGAGGYGQGGPGSGSGAAGAAASS

GTGDNGYGGQGGSGPGGAAAAAASS

GGQGGRGRYGPQGAGGYGQGGPGSGSGAAAAAASS

GTGDDGYGGQGGSGPGEAAAAAASS

GRQGGRGRYGPQGAGGYGQGGPGSGSGAAASS

GTGDDGYGGQGGSVAGGAAAAAASSAGQGGRGRYGSQGAGGYGQGGNGRGGNEAAAATASSTAALVANRLSSPSSLSRVSSAVSVFLDDDLEYPVAFSNAFDNVVSGITLSNSDISGCELLVQSLMEVLSAVLGTAYGLNANSSVDIVRSVVNRFD

>Aarg_MaSp3b

MAWITRLPLLVLVALCTQSIIVHGQDSHPWSDVRTTESFMKNFVECIRQSSYFNTDDIESIRDLSDTMIQSLNGMTAIGKTSHQMLQALNMGYAAGVAELVNSDGFYVQEKRNAIREAMRNSLIQTTGVVNESFMNEMDKLMQMFSQINVLNEDSGGNGVSAASSASASNVPGIGQSLGPQGQGSSSVSVSSTSVGGLQQRPVGSGSYEYSLSVNSIGGSGYGQGGYGPGGSGAAAVAASSGGGSGSPGYGGQVGYGPGGTAAAAAASDGQGGTGGGRYGPQGAGGYGQGGYGPGGSGAAAASS

GGGTGSPGYGGQGGYGPGGTAAAAAAS

DGQGGTGGGKYGPQGAGGYGQGGYGPSGSGAAAAAASS

GGGTGSPGYGPGGSAAAAAAS

DGQGGTGGGRYGPQGAGGYGQGGSGQDGTAAAAASSS

GGTGSPGYGGQGGYGPGGTGGVRYGPQGAGGYGQGGIGTGSGLGAAAAAASS

GAGPGTIGYGGEGIGGYGQGGADAAAAA

LGGQGGGRGGRYGPQGYGGYGQGGYGPGGSGAAAAAASS

GEGTGRSGYGEQGGSGTDSAAAAAAAA

GGGGGRYGPQGAGGYGQGGRGPGGSGAAAAAASA

GEVTGSNGYGGQGGYGPGGAAAAAAAS

GGQGGGGRYGSQGAGGYGQGGIGTGSGPGAAAAAASS

GAGKGSLGYGGQGGYGSDGTAAAAAAS

DGQGGTGGGRYGSQGAGGYGQRGSGPGGSGAAAAA

DSSGAGTGSPGYGGQGGYGPGGTAAAAAAS

DGLVGTGGGRYGPQGAGGYVQGGYGPGGSGAAAAAASS

GAGPGYGERGRkeiikkiivrrkggsqydteafeiegnnYGPQGGSGSGSGSSAAAASSS

TGSGSAGYGEQGESGPGGAAAAAASS

GGQGGRGRYGPQGAGGYGQGGPGYGSGAAAAAASS

GTGDDGYGGQGGSGPGGAAAAAASS

GGQGGRGRYGPQGAGGYGQGGPGSGSGAAAAAASS

GTGDDGYGGQGGSGPGGAAAAAASS

GGQGGRGRYGPQGAGGYGQGGPGSGSGAAAAAASS

GTGDDGYGGQGGPGGAAAAAASS

GGQGGRGRYGPQGAGGYGQGGPGSGSGAAASS

GTGDDGYGGQGGSGPGGAAAAAASS

GGQGGRGRYGPQGAGGYGQGGPGSGSGAAAAAASS

GTGDDGYGGQGGPGG-AAAAAAAS

GGQGGRGRYGPQGAGGYGQGGPGSGSGAAAAAASS

GTGDDGYGGQGGPGGAAAAAASS

GGQGGRGRYGPQGAGGYGQGGPGSGSGAAAAAASS

GTGDDGYGGQGGPGGAAAAAAAS

GGQGGRGRYGPQGAGGYGQGGPGSGSGAAAAAASS

GTGDDGYGGQGGPGGAAAAAAAS

GGQGGRGRYGPQGAGGYGQGGPGSGSGAAAAAASS

GTGDDGYGGQGGPGGAAAAAAAS

GGQGGRGRYGPQGAGGYGQGGPGSGSGAAAAAASS

GTGDDGYGGQGGPGGAAAAAAAS

GGQGGRGRYGPQGAGGYGQGGPGSGSGAAAAAASS

GTGDDGYGGQGGPGGAAAAAAAS

GGQGGRGRYGPQGAGGYGQGGPGSGSGAAAAAASS

GTGDDGYGGQGGPGGAAAAAAAS

GGQGGRGRYGPQGAGGYGQGGPGSGSGAAAAAASS

GTGDDGYGGQGGSGPGGAAAAAASS

GGQGGRGRYGPQGAGGYGQGGPGSGSGAAAAAASS

GTGDDGYGGQGGPGGAAAAAAAS

GGQGGRGRYGPQGAGGYGQGGPGSGSGAAAAAASS

GTGDDGYGGQGGPGGAAAAAAAS

GGQGGRGRYGPQGAGGYGQGGPGSGSGAAAAAASS

GTGDDGYGGQGGPGGAAAAAAAS

GGQGGRGRYGPQGAGGYGQGGPGSGSGAAAAAASS

GTGDDGYGGQGGSG?GGAAAAAASS

GGQGGRGRYGPQGAGGYGQGGPGSGSGAAAAAASS

GTGDDGYGGQGGPGGAAAAAAAS

GGQGGRGRYGPQGAGGYGQGGPGSGSGAAAAAASS

GTGDDGYGGQGGPGGAAAAAAAS

GGQGGRGRYGTQGAGGYGQGGPGSGSGAAAAAASS

GTGDDGYGGQGGPGGAAAAAAAS

GGQGGRGRYGPEGAGRYGQGGRGSGSGAAASS

GTGDDGYGGQGGPGGAAAAAAAS

GGQGGRGRYGPQGAGGYGQGGPGSGSGAAAAAASS

GTGDDGYGGQGGSGPGGAAAAAASS

GGQGGRGRYGPQGAGGYGQGAPGSGSGAAAAAASS

GTGDDGYGGQGGPGGAAAAAAAS

GGQGRRGRYGPQGAGGYGQGGPGSGSGAAAAAASS

GTGDDGYGGQGGPGGAAAAAAAS

GGQGGRGRYGPQGAGGYGQGGPGSGSGAAAAAASS

GTGDDGYGGQGGPGGAAAAAAAS

GGQGGRGRYGPQGAGGYGQGGPGSGSGAAAAAASS

GTGDDGYGGQGGSGSGGAAAAAASS

GGQGGRGRYGSQGAGGYGQGGSGRGGNGAAAATASSTAALVANRLSSPSSLSRVSSAVSVFLDDDLEYPVAFSNAFDNVVSGITLSNSDISGCELLVQSLMEVLSAVLGTAYGLNANSSVDIVRSVVNRYDY

>Atri_MaSp3a

MAWIARLPLLVLVALCTQSIVVNGLDRHPWQDTGTTELFMENFVECIRQSSYFNNEDIESIRDLAETLIQSLNGMQAKGKTSHQVLQALNMGYAAGVAELVNSDGTNLQEKRNAVREAMKNSLLQATGEVNESFMNEMDKLMQMFSQINALKGDSGGYGAGAESYASSASASNIQGIGQNLGSQGQGLSSVSVSSASVGGLPQGPVGSGSYGYSLSVNSLGGSPSGYGGQYASGTGVGQGRIGTGAAGGAAAATASSGASLNGNGLGYGGIGGYGFDGASAAVAVGSGGQGGYGPGNLYGQGGSGAGASASSGEGTGGAGYGLDGNGGYGGSGSAAATAASASGGQGGDGRYGPQGTSGYGLGGIGSGGAGAAAATASGEGPGGNGYGGQGGPAASAAAAAAS

GERGGNGRYGPQGTSGYGLGGIGSGGAGAAAAAAS

GEGPGGNGYGGQGGSAASAAAAAAS

GERGGNGRYGPQGTSGYGLRGIGSGGAGAAAAA

GEVPGGAGYGGQGGEAEAAAAASTS

ESGENGALPRSRefikkiivhrrlgsssnaeasvieeneyGPQAAGRYGIGGSGSGTAASAASS

GNGLLGANNGGYGYGGQGGSGAAAASS

GRGGDGRYGPRGVGAYGQGGSGLGAAAAAASS

GEGPGGAGYGGQGGSGAAAAAASS

GEGPGGAGYGRDGYGRDGYGGQGGSGAAAAAASS

GEGPVGAGYGRDGYGRGGYGRDGYGGQGGSGAAAAAASS

GEGPGGAGYGRDGYGGQGGSGDAAAAAAS

GGEGRDGRYGRRGSGSDGAGAAAAAASS

GEGPGGAGYGGQGGSGAAAAAASS

GEGPGGAGYGRDGYGRGGYGRDGLGGQGGSGAAAAAASS

GEGTGGAGYGRDGYGRGGYGRGGYGRDGYGGQDGTGAAAAAASS

GEGPGGAGYGRDGYGRGGYGRDGLGGQGGSGAAAAAASS

GEGPGGAGYGGQGGSGAAAAAASS

GEGPGGAGYGRDGYGRDGLGGQGGSGAAAAAASS

GEGPGGAGYGGQGGSGAAAAAASS

GEGPGGAGYGRDGYGGQGGSGDAAAAAAS

GGRGRDGRYGRRGSGSDGAGAAAAAASS

GEGPGGAGYGGQGGSGAAAAAASS

GEGPGGAGYGRDGYGRGGYGRDGYGRGGYGRDGLGGQGGSGAAAAAASS

GEGPGGAGYGRDGYGGQGGSGAAAAAASS

GEGPGGTGYGRDGYGRDGLGGQGGSGAAAAAASS

GEGPGGAGYGGQGGSGAAAAASSS

GEGPGGAGYGRDGYGRGGYGRDGLGGQGGSGAAAAAASS

GEGPGGAGYGGQGGSGAAAAAASS

GEGPGGAGYGRDGYGRDGLGGQGGSGAAAAAASS

GEGPGGAGYGGQGGSGAAAAASS

GEGPGGAGYGRDGYGGQGGSGDAAAAAAS

GGRGRDGRYGRRGSGSDGAGAAAAAASS

GGAGYGGQGGSGAAAAAASS

GEGPGGAGYGRDGYGRGGYGRGGYGRDGLGGQGGSGAAAAAASS

GEGPGGAGYGGQGGSGAAAAAASS

GEGPGGAGYGRDGYGRGGYGRDGLGGQGGSGAAAAAASS

GEGPGGAGYGGQGGSGAAAAASSS

GEGPGGAGYGRDGYGRGGYGRDGLGGQGGSGAAAAAASS

GEGPGGAGYGGQGGSGAAAAAASS

GEGPGGAGYGRGGYGGQGGSGDAAAAAAS

GGRGRDGRYGRRGSGSDGAGAAAAAASS

GGAGYGGQGLSGAAAAAASS

GEGPGGAGYGRDGYGRGGYGRGGYGRDGLGGQGGSGAAAAAASS

GEGPGGAGYGGQGVSGAAAAAASS

GEGPGGAGYGRDGYGRGGYGRDGLGGQGGSGAAAAAASS

GEGPGGAGYGGQGGSGAAAAAASS

GEGPGGAGYGRDGYGRGGYGRDGLGGQGGSGAAAAAASS

GEGPGGAGYGGQGGSGAAAAAASS

GEGPGGAGYGRDGYGRGGYGRDGLGGQGGSGAAAAAASS

GEGPGGAGYGRGGYGRDGYGGQGGSGAAAAAASS

GEGPGGAGYGRDGYGGQGGSGDAAAAASS

GGGGGDGSYGRRGSGSDGAGAAAAAASS

GGAGYGGQGGSGAAAAAASS

GEGPGGAGYGRDGLGGQGGSGAAAAAASS

GEGPGGAGYGGQGGSGAAAAAASS

GEGPRGAGYGRDGYGRGGYGRDGYGEQGGSGAAAAAASS

GEGPGGAGYGGQGGSGAAAAAASS

GEGPGGAGYGRDGYGRGGYGRDGLGEQGGSGAAAAAASS

GEGPGGAGYGGQGGSGAAAAAASS

GEGPGGAGYGRDGYGRGGYGRDGLGGQGGSGAAAAAASS

GEGPGGAGYGGQGGSGAAAAAASS

GEGPGGAGYGRGGYGRDGYGGQGGSGAAAAAASS

GEGPGGAGYGRDGYGGQGESGDAAAAASS

GERGGDGSYGRRGSGSDGAGAVAAAASS

GEGPGGAGYGGQGGSGAAAAAASS

GEGPGGAGYGRDGYGRGGYGRGGYGRGGYGRDGLGGQGGSGAAAAAASS

GEGPGGAGYGGQGGSGAAAAAASS

GEGPGGAGYGRDGYGRGGYGRDGYGGQGGSGAAAAAASS

GEGPGGAGYGGQGGSGAAAAAASS

GEGPRGAGYGRDGYGRGGYGRDGYGGQGGSGAAAAAASS

GEGPGGAGYGGQGGSGAAAAAASS

GEGPRGAGYGRDGYGRGGCGRDGYGRDGYGGQGGSGAAAAAASS

GEGPGGAGYGRDGYGRDGYGGQGGSGDAAAAASS

GGQRQLWSEQGSGSDGAGAAAAAASS

GEGPGGAGYGGQGGSGAAAAAASS

GEGPGGAGYGRDGYGRGGYGRDGLGGQGGSGAAAAAASS

GEGPGGAGYGGQGGSGAAAAAASS

GEGPGGAGYGGQGGSGAAVAAASS

GEGPGGAGYGRDGYGGQGGSGAAAAAAASS

GRGGDGRYASGGSGSDGAAAASASSTAATVASRLSSPTSLSRVSSAVSIFLDDDLDYPEAFSNAFDNVVSGITLANSDISGCELLVQSLMEVLCAVMGTAYGLNANSSVDIVRNVVNRYDY

>Atri_MaSp3b

MTQIIFIRKHRSDKMRRRNIEPPASYHEIYDIKGWKIDRKINQSRRTKTTMTWIARLPLLVLVTLCIQSIIVHGLDSHPWQDASTTGLFMENFVQYIRQSSYFNSDDIDSIKVLADTLIQSLNGMQAKGKTSHQMLQALNLGYAAGVAELVNSDGTNLQEKRNAIREAMKNSLLQATGEVNESFMNEMDKLMQMFSQVNALNEDSGGYGAGAESYASSASASNIQGIGQNLGSQGQGSSSVSVSSASVGGLPQGPVGSGSYGFSLSVNSLGGSPNGYGGQYASGTGVELGRLGTGAAGGAAAATAS

GGASLNGNGFGYGGIGGYGLDGASAAVAVGSGGQRGYGQGIGFSQRGSGPGAAAAAASS

GEGPGGTGYGGNGYGGQGGSGAAAAAAAS

GGRGGDGRYGPQGAGSYGPGGSGSGAAAAAASS

GEGPSGAVYGGQGGSGAAAAVAASS

GRGGDGRYGSLGAGSYGPGGSGSGSAAAAASS

GEGQSGTGYGRNGYGGQDGSGAAAAAAAS

GGRGGEGRYGPQGAGSYGPGGNGSGAAAAAASS

GEGPSGAGYGGNGYGGQGGSGAATAAAAS

GGRGGDGRYGPQGAGSYGPGGSGSGAAAAAVSS

GEGESGAGYGENGYGLEDGSASATASGFGGSGGLRRSkevikkiivhrrlganGVDYDGQGGSRSDAAAATS

GGRGGDGRYGLRGVGGYGPEGSGSGAAAAAASS

GEGPGGAGYGRDGYGGQGGSGAAAAVASS

GEGPGGAGYGGDRYGGQGGSGAAAAAAAS

GGLEGDGRYGLRGVVGYGPGGSGSGAAAAAASS

GEGPGGAGYGRDRYGGEGGSEAAASS

GEGPGGAGYGGQGGSGASAAAASS

GEGGRGGYGRYGPRGIGSNGAGAAAAA

GEGPGGAGYGGQGGSGAAAASASS

GKGGRGGYGRYGPSGSGSNGAGAAAAAASS

GEGPGGAGYGRDGYGGQGGSEAAAAAASS

GEGPGGAGYGGQGGSGASAAAASS

GEGGRGGYGRYGPRGIGSNGAGAAAAAS

EGPGGAGYGGQGGSGASAAAASS

GEGGRGGYGRYGPSGSGSNGAGAAAAAASS

GEGPGGAGYGGDGYGGQGGSGAAAAAAAS

GGREGDGRYGLRGVVGYGPGGSGAGAAAAAASS

GEGPGGAGYGRDGYGGQGGSEAAAAAASS

GEGPGGAGYGGQGGSGASAAAASS

GEGGRGGYGRYGPRGIGSNGAGAAAAA

GEGPGGAGYGGQGGSGASAAAASS

GEGGRGGYGRYGPRGSGSNGAGAAAAAASS

GEGPGGAGYGGTKWGTGGSGASAAAASS

GEGGRGGYGRYGAGAAAAAASS

GEGPGGAGYGRDGYGGQGGSEAAAAAASS

GEGPGGAGYGGQGGSGASAAAASS

GEGGRGGYGRYGPRGSGSNGAGAAAAA

GEGPGGAGYGGQGGSGASAAAASS

GEGGRGGYGRYGPSGSGSNGAGAAAAAASS

GEGPGGAGYGGDGYGGQGGSGAAAAAAAS

GGREGDGRYGLRGVVGYGPGGSGAGAAAAAASS

GEGPGGAGYGRDGYGGQGGSEAAAAAASS

GEGPGGAGYGGQGGSGASAAAASS

GEGGRGGYGRYGPRGIGSNGAGAAAAA

GEGPGGAGYGGQGGSGASAAAASS

GEGGRGGYGRYGPSGSGSNGAGAAAAAASS

GEGPGGAGYGGDGYGGQGGSGAAAAAASS

GGREGDGRYGLRGVVGYGPGGSGAGAAAAAASS

GEGPGGAGYGRDGYGGQGGSEAAAAAASS

GEGPGGAGYGGQGGSGASAAAASS

GEGGRGGYGRYGPRGIGSNGAGAAAAA

GEGPGGAGYGGQGGSGASAAAASS

GEGGRGGYGRYGPSGSGSNGAGAAAAAASS

GEGPGGAGYGGDGYGGQGGSGAAAAAAAS

GGREGDGRYGLRGVVGYGPGGSGAGAAAAAASS

GEGPGGAGYGRDGYGGQGGSEAAAAAASS

GEGPGGAGYGGQGGSGASAAAASS

GEGGRGGYGRYGPRGIGSNGAGAAAAA

GEGPGGAGYGGQGGSGASAAAASS

GEGGRGGYGRYGPSGSGSNGAGAAAAAASS

GEGPGGAGYGGDGYGGQGGSGDAAAAAAS

GGREGDGRYGLRGVVGYGPGGSGAGAAAAAASS

GEGPGGAGYGRDGYGGQGGSEAAAAAASS

GEGPGGAGYGGQGGSGASAAAASS

GEGGRGGYGRYGPRGIGSNGAGAAAAA

GEGPGGAGYGGQGGSGASAAAASS

GEGGRGGYGRYGPSGSGSNGAGAAAAAASS

GEGPGGAGYGGDGYGGQGGSGAAAAAASS

GGREGDGRYGIRGVVGYGPGGSGAGAAAAAASS

GEGPGGAGYGRDGYGGQGGSEAAAAAASS

GEGPGGAGYGGQGGSGASAAAASS

GEGGRGGYGRYGPRGSGSNGAGAAAAA

GEGPGGAGYGGQGGSGASAAAASS

GEGGRGGYGRYGPRGIGSNGAGAAAAAASS

GEGPGGAGYGGDGYGGQGGSGAAAAAASS

GEGPGGAGYGGQGGSGASAAAASS

GEGGRGGYGRYGPRGSGSNGAGAAAAA

GEGPGGAGYGGQGGSGASAAAASS

GEGGRGGYGRYGPSGSGSNGAGAAAAAASS

GEGPGGAGYGGDGYGGQGGSGAAAAAASS

GGREGDGRYGIRGVVGYGPGGSGAGAAAAAASS

GEGPGGAGYGRDGYGGQGGSEAAAAAASS

GEGPGGAGYGGQGGSGASAAAASS

GEGGRGGYGRYGPRGSGSNGAGAAAAA

GEGPGGAGYGGQGGSGASAAAASS

GEGGRGGYGRYGPRGIGSNGAGAAAAAASS

GEGPGGAGYGGDGYGGQGGSGAAAAAASS

GGREGDGRYGLRGVVGYGPGGSGAGAAAAAASS

GEGPGGAGYGRDGYGGQGGSEAAAAAASS

GEGPGGAGYGGQGGSGASAAAASS

GEGGRGGYGRYGPRGSGSNGAGAAAAA

GEGPGGAGYGGQGGSGASAAAASS

GEGGRGGYGRYGPRGIGSNGAGAAAAAASS

GEGPGGAGYGGDGYGGQGGSGAAAAASSS

GGREGDGRYGLRGVVGYGPGGSGAGAAAAA

GSSGEGPGGAGYGRDGYGGQGGSEAAAAAASS

GEGPGGAGYGGQGGSGASAAAASS

GEGGRGGYGRYGPRGSGSNGAGAAAAA

GEGPGGAGYGGQGGSGASAAEASSGEGGRGGYGRYGPRGIGSNGAGAAAAAASS

GEGPGGAGYGRDGYGGQGGSGAAAAAAASS

GRGGDGRYGPGGSGSDGAAAASASSTAATVASRLSSPTSLSRISSAVSIFLDDDLDYPEAFSNAFDNVVSGITLANSDISGCELLVQSLMEVLCAVMGTAYGLNANSSVDMIRSVVNRYDY
